# Supplementary material for: Assessing the quality of bee honey on the basis of melissopalynology as well as chemical analysis
Source: PLoS One. 2023 Aug 8;18(8):e0289702. doi: 10.1371/journal.pone.0289702 (PMC10409281; doi:10.1371/journal.pone.0289702)
Supplement: Attachment — Submitted filename: PONE-D-22-31247_reviewer.pdf [file pone.0289702.s002.pdf]

PLOS ONE

Assessing the Quality of Bee Honey on the basis of melissopalynology as well chemical analysis: a geobotanic approach

--Manuscript Draft--

**Manuscript Number:**	PONE-D-22-31247
**Article Type:**	Research Article
**Full Title:**	Assessing the Quality of Bee Honey on the basis of melissopalynology as well chemical analysis: a geobotanic approach
**Short Title:**	Assessing the Quality of Bee Honey on the basis of melissopalynology as well chemical analysis
**Corresponding Author:**	Ahmadreza Mehrabian, PhD.<br>Shahid Beheshti University<br>Tehran, IRAN, ISLAMIC REPUBLIC OF
**Keywords:**	Melissopalynology, Botanical origin, Scanning Electron Microscopy (SEM), Iran, Pollen, palynology
**Abstract:**	Melissopalynological analysis and chemical composition of honey provide us with useful and valuable information about the botanical and geographical origin of honey. The data in question is important for authentication as well as quality testing of honey, so it is considered as a key method in honey regulation Here, we used melissopalynology and chemical analysis to evaluate different honey samples from two main Iranian hubs of honey. Sampling was carried out at two important poles of Iranian honey production, the central Alborz region, and the mountain ecosystems of Zagros in the years 2020-2021. Therefore, 52 samples from Alborz (N. Iran) as well as 42 samples from the Zagrosian ecosystems (western Iran) belonging to different ecologically habitats were collected. In addition, samples were taken at 7 altitudinal levels between 0-3500 m a.s.l. Furthermore in this study, various chemical analyzes such as the effect of antioxidant activity, the amount of phenolic compounds, the pH, and moisture content of honey samples were evaluated. Our results showed that all honey samples were classified as polyfloral honey. Based on our findings, 57 honey samples (61%) contained the standard amount of pollen . A total of 42 plant families and 55 genera were identified in the studied samples, with the highest presence of Asteraceae, Fabaceae, Rosaceae, Apiaceae, and Apocynaceae. Finally, the antioxidant activity rate was 19% -to 98%, phenolic compounds from 0.08 to -0.51 ppm, pH from 1.90 to -5.21, and humidity from 13% -to 18.40%.
**Order of Authors:**	zahra shakoori, masterS degree
	Ahmadreza Mehrabian, PhD.
	Dariush Minai
	Farid Salmanpour, Masters degree
	Farzaneh Khajoei Nasab
**Additional Information:**	
**Question**	**Response**
**Financial Disclosure**<br><br>Enter a financial disclosure statement that describes the sources of funding for the work included in this submission. Review the submission guidelines for detailed requirements. View published research articles from *PLOS ONE* for specific examples.	The authors received no specific funding for this work.

Powered by Editorial Manager® and ProduXion Manager® from Aries Systems Corporation

This statement is required for submission and **will appear in the published article** if the submission is accepted. Please make sure it is accurate.<br><br>**Unfunded studies**<br>Enter: *The author(s) received no specific funding for this work.*<br><br>**Funded studies**<br>Enter a statement with the following details:<br>• Initials of the authors who received each award<br>• Grant numbers awarded to each author<br>• The full name of each funder<br>• URL of each funder website<br>• Did the sponsors or funders play any role in the study design, data collection and analysis, decision to publish, or preparation of the manuscript?<br>• **NO** - Include this sentence at the end of your statement: *The funders had no role in study design, data collection and analysis, decision to publish, or preparation of the manuscript.*<br>• **YES** - Specify the role(s) played.<br><br>* typeset	
**Competing Interests**<br><br>Use the instructions below to enter a competing interest statement for this submission. On behalf of all authors, disclose any competing interests that could be perceived to bias this work—acknowledging all financial support and any other relevant financial or non-financial competing interests.<br><br>This statement is required for submission and **will appear in the published article** if the submission is accepted. Please make sure it is accurate and that any funding sources listed in your Funding Information later in the submission form are also declared in your Financial Disclosure statement.<br><br>View published research articles from *PLOS ONE* for specific examples.	The authors have declared that no competing interests exist.

**NO authors have competing interests**<br><br>Enter: *The authors have declared that no competing interests exist.*<br><br>**Authors with competing interests**<br><br>Enter competing interest details beginning with this statement:<br><br>*I have read the journal's policy and the authors of this manuscript have the following competing interests: [insert competing interests here]*<br><br>* typeset	
**Ethics Statement**<br><br>Enter an ethics statement for this submission. This statement is required if the study involved:<br><br>• Human participants<br>• Human specimens or tissue<br>• Vertebrate animals or cephalopods<br>• Vertebrate embryos or tissues<br>• Field research<br><br>Write "N/A" if the submission does not require an ethics statement.<br><br>General guidance is provided below. Consult the submission guidelines for detailed instructions. **Make sure that all information entered here is included in the Methods section of the manuscript.**	We have only collected honey samples in this study.

**Format for specific study types**<br><br>**Human Subject Research (involving human participants and/or tissue)**<br>• Give the name of the institutional review board or ethics committee that approved the study<br>• Include the approval number and/or a statement indicating approval of this research<br>• Indicate the form of consent obtained (written/oral) or the reason that consent was not obtained (e.g. the data were analyzed anonymously)<br><br>**Animal Research (involving vertebrate animals, embryos or tissues)**<br>• Provide the name of the Institutional Animal Care and Use Committee (IACUC) or other relevant ethics board that reviewed the study protocol, and indicate whether they approved this research or granted a formal waiver of ethical approval<br>• Include an approval number if one was obtained<br>• If the study involved *non-human primates*, add *additional details* about animal welfare and steps taken to ameliorate suffering<br>• If anesthesia, euthanasia, or any kind of animal sacrifice is part of the study, include briefly which substances and/or methods were applied<br><br>**Field Research**<br><br>Include the following details if this study involves the collection of plant, animal, or other materials from a natural setting:<br>• Field permit number<br>• Name of the institution or relevant body that granted permission	
**Data Availability**<br><br>Authors are required to make all data underlying the findings described fully available, without restriction, and from the time of publication. PLOS allows rare exceptions to address legal and ethical concerns. See the PLOS Data Policy and FAQ for detailed information.	Yes - all data are fully available without restriction

Powered by Editorial Manager® and ProduXion Manager® from Aries Systems Corporation

A Data Availability Statement describing where the data can be found is required at submission. Your answers to this question constitute the Data Availability Statement and **will be published in the article**, if accepted.<br><br>**Important:** Stating 'data available on request from the author' is not sufficient. If your data are only available upon request, select 'No' for the first question and explain your exceptional situation in the text box.<br><br>Do the authors confirm that all data underlying the findings described in their manuscript are fully available without restriction?	
**Describe where the data may be found in full sentences. If you are copying our sample text, replace any instances of XXX with the appropriate details.**<br><br>• If the data are **held or will be held in a public repository**, include URLs, accession numbers or DOIs. If this information will only be available after acceptance, indicate this by ticking the box below. For example: *All XXX files are available from the XXX database (accession number(s) XXX, XXX.).*<br>• If the data are all contained **within the manuscript and/or Supporting Information files**, enter the following: *All relevant data are within the manuscript and its Supporting Information files.*<br>• If neither of these applies but you are able to provide **details of access elsewhere**, with or without limitations, please do so. For example:<br><br>*Data cannot be shared publicly because of [XXX]. Data are available from the XXX Institutional Data Access / Ethics Committee (contact via XXX) for researchers who meet the criteria for access to confidential data.*<br><br>*The data underlying the results presented in the study are available from (include the name of the third party*	The data obtained in this study are placed in the figshare repository and its link is mentioned at the end of the manuscript, before the references.

Powered by Editorial Manager® and ProduXion Manager® from Aries Systems Corporation

and contact information or URL).<br>• This text is appropriate if the data are owned by a third party and authors do not have permission to share the data.<br><br>* typeset	
Additional data availability information:	Tick here if the URLs/accession numbers/DOIs will be available only after acceptance of the manuscript for publication so that we can ensure their inclusion before publication.

Powered by Editorial Manager® and ProduXion Manager® from Aries Systems Corporation

نسخه خطی Click here to access/download;Manuscript;Manuscript.docx

Assessing the Quality of Bee Honey on the basis of melissopalynology as well chemical analysis: a geobotanic approach

Zahra Shakoori[1], Ahmadreza Mehrabian[*1], Dariush Minai[1], Farid Salmanpour[2], Farzaneh Khajoei Nasab[1]

1. Department of Plant Science and Biotechnology, Faculty of Science and Biotechnology, Shahid Beheshti University, Tehran, Iran, Email: zahra_shakoori.eco@yahoo.com, https://orcid.org/0000-0003-3057-8981 a_mehrabian@sbu.ac.ir d_mtehrani@sbu.ac.ir Farzaneh.khajoei@yahoo.com
2. Department of Biodiversity and Ecosystem Management, Research Institute of Environmental Sciences, Shahid Beheshti University, Tehran, Iran, Email: farid.salmanpour@yahoo.com, https://orcid.org/0000-0002-1517-9061

Abstract

Melissopalynological analysis and chemical composition of honey provide us with useful and valuable information about the botanical and geographical origin of honey. The data in question is important for authentication as well as quality testing of honey, so it is considered as a key method in honey regulation Here, we used melissopalynology and chemical analysis to evaluate different honey samples from two main Iranian hubs of honey. Sampling was carried out at two important poles of Iranian honey production, the central Alborz region, and the mountain ecosystems of Zagros in the years 2020-2021. Therefore, 52 samples from Alborz (N. Iran) as well as 42 samples from the Zagrosian ecosystems (western Iran) belonging to different ecologically habitats were collected. In addition, samples were taken at 7 altitudinal levels between 0-3500 m a.s.l. Furthermore in this study, various chemical analyzes such as the effect of antioxidant activity, the amount of phenolic compounds, the pH, and moisture content of honey samples were evaluated. Our results showed that all honey samples were classified as polyfloral honey. Based on our findings, 57 honey samples (61%) contained the standard amount of pollen . A total of 42 plant families and 55 genera were identified in the studied samples, with the highest presence of Asteraceae, Fabaceae, Rosaceae, Apiaceae, and Apocynaceae. Finally, the antioxidant activity rate was 19% -to 98%, phenolic compounds from 0.08 to -0.51 ppm, pH from 1.90 to -5.21, and humidity from 13% -to 18.40%.

Keywords: Melissopalynology, Botanical origin, Scanning Electron Microscopy (SEM), Iran, Pollen, palynology.

1. Introduction

Honey is a precious natural product processed by bees from the nectar of flowers, and is stored in the hive. Honey production in an area depends mainly on the vegetation and climate of thate region, as the flowering season and nectar production can vary for a species in different regions (Zimmerman, 1988). With the developing the of the honey market through the world identification of botanical origin as well as its authenticity has received a lot of attention in international societies (Kaskonine & Venskutonis, 2010). [illegible]ke honey and poor-quality honey are major problems in the global honey trade. Honey, which is composed, of many stable elements as well as pollen grains, collected by bees along with nectar. Melissopalynology as an effective method as well as key criteria has been widely used to determine the purity, geographical and botanical origins of natural honey (Maurizo 1975, ; Alvarez-Suarez 2017). Louveaux ([illegible]0, 1978) first founded this method. However, several laboratory procedures have been developed to study of pollen grains in honey latter (Bryant & Jones 2001). Melissopalynology studies evaluated pollen grains in honey (Jones and Bryant 1996 & Louveaux et al. 1970 & Corvucci et al. 2015 & Menad et al.2020). R[illegible]nt analyzes represent valuable data on the botanical and geographical origins of honey. Also, a major authentication method is honey sorting (Shakoori et al.2022). Furthermore, the botanaical and geographical origin of honey is important to consumers and influences the quality of the product (Alnaqdy et al. 2005; Basualdo et al. 2007; Bogdanov et al. 2008). In addition, the botanical origin of honey influences the physical, chemical, organoleptic and bioactive properties of honey (Kortesniemi et al. 2018) which is considered as a valuable indicator of quality and authenticity of honey. Furthermore, the quality of honey mainly depends on the nectariferous plant taxa that honey bees use in their diet . Accordingly, different melliferous plant species have different characteristics as well as different applications (e.g. drugs, nutrients, etc.)(Ozturk and Ercan 2010, Tucak et al. 2000,).

Iran was ranked 9th i[illegible]e world or honey production from 1993 to 2018 (FAOSTAT, 2018). High climate variability , topographic complexity (Stöklin, 1968; Zohary, 1973), the geographic isolation of the Iranian highlands (Noroozi et al., 20[illegible]) as well as soil diversity (Dewan & Famouri, 1963) established different phytogeographical units in Iran (Takhtajan, 1986; Zahran, 2010), which made it the center of a endemism of plant taxa in SW Asia (Klein, 1972; Hedge & Wendelbo, 1978), the center of endemism of Irano-Turanian (Takhtajan, 1986; Klein, 1981 &

1992; Leonard, 1991), as well as an important center of plant diversity around the world (Davis & Heywood, 1994; Barthlott, 1996 & 1999; Kier et al., 2005). These ecological conditions have led to the formation of valuable habitats for beekeeping in the country, which makes high diversity of honey production in Iran.

The Alborz together with the Zagros Mountains in Iran have created transition zones that have lead to high diversity and endemism in this region (Klein, 1972, Mehrabian et al. 2021). Influenced by elevation diversity in this area , various vegetation types have been formed (Fayaz et al. 2015, Ghelich Nia 2017), which have created suitable habitats for beekeeping and honey production (Nazarian et al. 1997, Razaghi et al. 2006). Today, the melissopalynology method has been used by a number of researchers in Iran, including: the melissopalynology of some honeys in Tehran Province (Nazarian et al. 1997), the melissopalynology of honey in Karaj, N. Iran (Sanei Shariat Panahi, 1974), melissopalynology of some honeys of Khorasan, N.E Iran (Ghoraishi & Memariani 2000), assessment the pollen grains of honey in Isfahan Province, C. Iran (Faghih et al. 2004), the melissopalynology of honey in some zones of Mazandaran Province (Razaghi et al. 2006), melissopalynology of honey in Arasbaran, N.W Iran (Ahmad Abad et al. 2017), and origin evaluation of some Iranian honeys (Khansaritoreh et al. 2021).

In addition to nutrients, honey contains bioactive substances that can have a positive effect on the human body (Rao et al.2016). The health properties of honey are due to the presence of phenolic compounds, including flavonoids, phenolic acids and their esters, as well as organic acids, free amino acids, vitamins, carotenoid derivatives, enzymes and biological elements (Kędzierska-Matysek et al. 2021). These compounds have antioxidant properties (Cianciosi et al.2018). Oxidation processes can be dealt with using antioxidant agents. Antioxidants can prevent many diseases or even reduce their symptoms (Cianciosi et al.2018). Honey can be a complementary source of antioxidants in a balanced diet (Alvarez-Suarez et al.2013). antioxidative of honey are attributed to the presence of phenolic compounds (Al-Mamary et al., 2002, Al Kiyumi et al.2021). Moisture level of bee honey is very important for its stability against fermentation and granulation. Low moisture protects honey from microbiological activity and therefore can be preserved for a longer period of time. (Cantarelli et al., 2008, Buba et al., 2013).

The aim of the study was to evaluate different honey samples originated from different ecological regions of Iran based on melissopalynological and chemical analysis. In addition, the

study tries to analyze the mentioned data in relation to geographical factors. Therefore, conducting a comparative study in different regions while comparing honey produced analyzes the relationship between pollen factors and some important chemical factors of honey with ecological factors.

2. Study area

2.1. Physical geography

Iran as a prominent area of Iranian Plateau in southwestern Asia situated in between 24°–40° N longitude and 44°–64° E latitude, As well a limited zone of the orogenic belt (Zagros, Alborz, and other mountain chains), covers the Asian block along with Arabian-African unity (Berberian and King, 1981). The prominent geomorphological zones of Iran including Alborz, Zagros, Kopet Dagh, as well several interior mountain chains. The Zagros mountain chain extended from northwest to southeast (Hokme, 2007). This geomorphological formation has created an natural wall between the Iranian plateau (northeast) and the Persian Gulf and Mesopotamian (southwest) basins. Also, the Alborz mountain chain as an active, arcuate fold-and-thrust belt (Stöcklin, 1974, 1977) as well a prominent sector of the Alpine-Himalayan geological formation. Shaped as an east-west, softly sinuous crescent in northern Iran, it forms a natural barrier between the Caspian Sea and the central Iranian plateau (Stöcklin, 1974). These natural massifs surrounding the central plateau and lead to diverse precipitation patterns in the mentioned area. In addition, Iran covers Mediterranean (western, northwestern Iran), temperate (northern Iran), and tropical (southern coast zones of the Persian Gulf and the Gulf of Oman) macro-bioclimates; however, its climatic zones consist of 35.5% hyper-arid, 29.2% arid, and 20.1% semi-arid regions. Additionally, precipitation patterns represent an average of about 250 mm annually, somewhat less than one third of the average rainfall in the world (860 mm) (Amiri and Eslamian, 2010). Most of our honey samples were collected from Alborz and Zagros sections, which have the highest honey production in Iran, which includes more than half of Iran's provinces, and most of the collected samples were close to forested and protected areas (Figure 1).

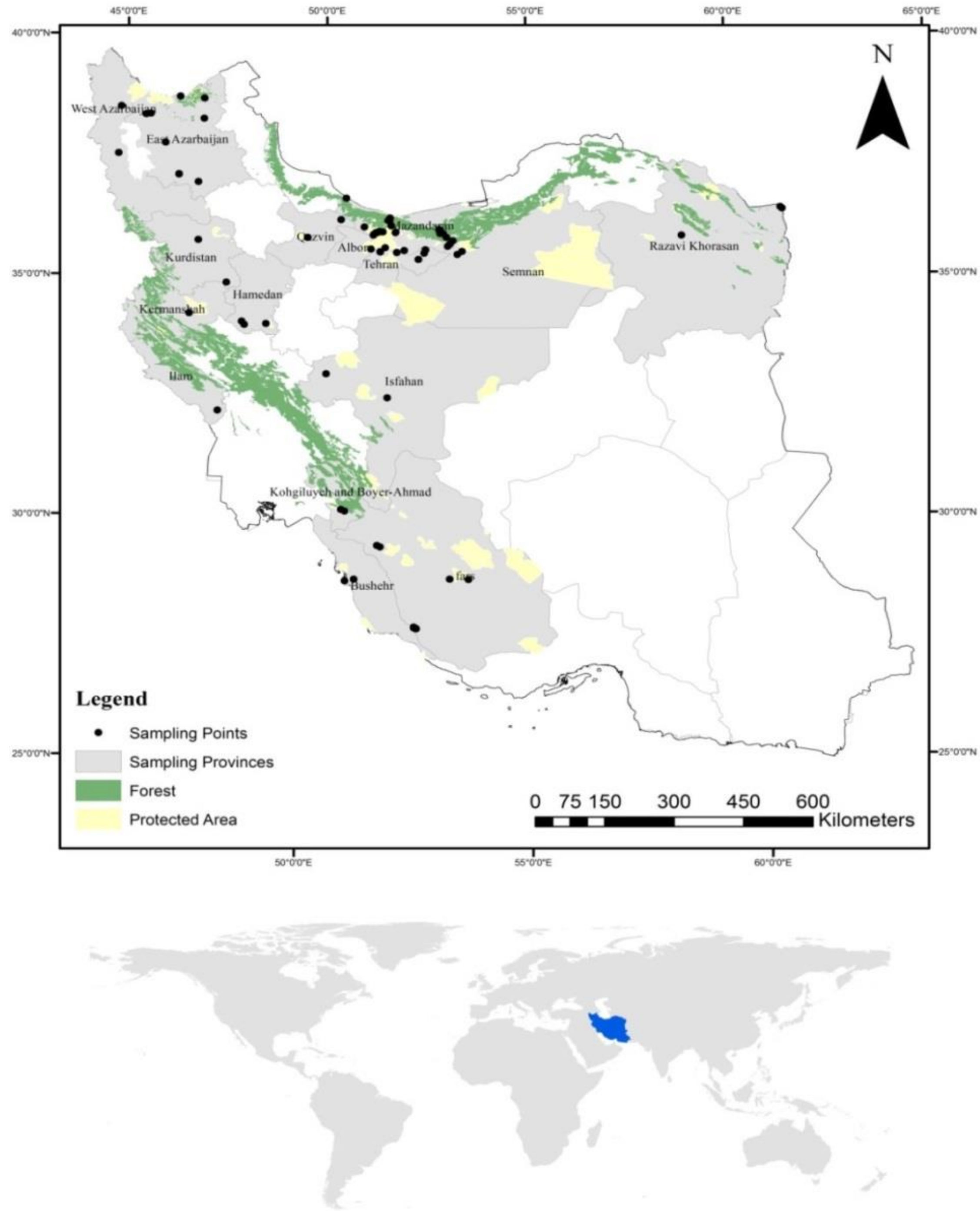

Figure. 1. Study area and sample collection points

2.2. Geobotany and Plant Ecology

The high forests of the Alborz, which are located only on the northern slopes (the Caspian Sea side), are dominated by deciduous Quercus macranthera, which usually reaches an altitude of 2400 meters in Alborz exceptionally and only in small protected pockets up to 2850 meters (Klein and Lacoste 1989; Naqinezhad and Esmailpoor 2017). In terms of plant geography, these forests belong to the Euro-Siberian region (Zohary 1973), and according to a bioclimatic classification, this region belongs to the Mediterranean pluviseasonal-oceanic biological climate using the Rivas-Martinez approach (Djamali et al. 2011). The southern slopes are mostly treeless, resulting in longer continental climates and stronger radiation during the long dry summer, although meteorological station data show that winter rainfall is still around ca. 550 mm> 2500 m a.s.l. In addition to plant species, Alborz high forests (Hyrkanian forests) host valuable animal species such as Iranian leopards(Farhadinia et al.2022). Zagros is the largest mountain range in Iran, which stretches from northeastern Iraq to southern Iran and its length is about 1300 km and width is about 250 km. This mountain range with an area of 394,000 square kilometers covers about a quarter of Iran's area (Noroozi et al. 2020). It has high peaks that reach sub-branches in different parts, but mostly in the central and southern Zagros. It has a continental climate and a Mediterranean rainy regime with cold and wet winters and hot and dry summers. Our database lists 3642 vascular plant species from this mountain range. The Zagros is located completely within the Iran-Turanian biogeographical region and the center of Iran-Anatolia biodiversity. More than 70% of the species are composed of Iranian-Turanian elements. The major vegetation types of the Zagros are oak woodlands, Amygdalus-Pistacia shrublands, montane steppe shrublands, wetlands, chasmophytic habitats, subalpine, alpine and subnival zones. (Noroozi et al. 2020). The vast expanse of the Zagros, its diverse climate and topography, and its high altitude range have led to the development of very different ecosystems and vegetation types. The main source of Zagros rainfall is the western systems carrying moisture from the Mediterranean and the Black Sea (Alijani and Harman 1985). The north-south extension of the Zagros

Range creates a strong rain shadow effect with much less annual rainfall on the eastern slopes than their western counterparts (Alijani et al. 2008). According to modern bioclimatic classifications (DJamali et al. 2011), Zagros belongs to different bioclimatic zones. Much of the western Zagros lies beneath the seasonal Mediterranean continental zone, characterized by high

annual rainfall in winter and early spring, which is in favor of deciduous oak forests. This mountain range shows the climate of the arid Mediterranean continents in its northeastern parts and the conditions of the drier Mediterranean continents in its southeastern parts (Djamali et al. 2011).

3. Materials and methods

3.1. Field studies

This study is based on direct collection of honey samples from hives in the area from May to October during 2020-2021. We selected sites for sampling that included different geographical and botanical areas (Al Naggar and El-Sofany 2018). Using a data collection form, the geographical coordinates of the hives were recorded by a Garmin Map 64s GPS device, [illegible] amount of height, the type of honey and the claims of beekeepers. Based on the diversity of ecological conditions, bee colonies were identified and samples were taken from the hives. Based on the diversity of ecological conditions, bee colonies were identified and samples were taken from the hives. From each colony, 1 sample of 300 g of unrefined and non-clay honey was prepared for pollen extraction and chemical analysis such as pH, antioxidant, total phenol, humidity. The samples were placed in sterile sealed containers and stored in a cool place until they reached the laboratory (Ponnuchamy et al. 2014).

3.2. Laboratory studies

Laboratory studies were performed in two parts:

Melissopalynology based on scanning electron microscopy (SEM)

These studies are based on the method (Louveaux et al. 1978). The melissopalynological method recommended by the International Commission on Bee Botany (ICBB) (Louveaux et al.1978; Von Der Ohe et al., 2004). First, 10 grams of honey in [illegible] 20 mL of distilled water was dissolved on a hot plate at a temperature of less than 40°C. After this step, honey wax samples were passed through a cloth strainer to remove large particles of honey wax, then the liquid was poured into centrifugal tubes with conical ends and a capacity of 30 mL. After several laboratory centrifuges (2500-3000 rpm, Model TDL-4 centrifuge) at room temperature (21 to 25°C), pollen grains were extracted from honey. In the first round, the samples were centrifuged for 10 minutes (about 2500 rpm), and the supernatant was carefully discarded to prevent pollen loss (Ponnuchamy et al. 2014). The remaining precipitate was mixed again with about 10 mL of

distilled water, but this time the samples were centrifuged for 5 minutes. In our study, we repeated this process 4 to 7 times, depending on the amount of sugar in the samples, so that the sugar in the honey was completely removed. But after the last centrifuge, we poured the final remaining liquid into the watch glass and placed it on the hot plate so that the water evaporated completely and the pollen grains remained at the bottom of the container. During the process, great care must be taken to prevent any contamination from external pollen, which is from previous honey products (use of disposable appliances is recommended as much as possible) or pollen grains in the air (close windows and limit exposure).

We placed the prepared pollen grains on special bases and after covering the samples with gold metal, we placed them inside the housing of HITACHI SU3500 scanning electron microscope (SEM) machine made in Japan, and depending on the size of the sample, using this microscope, it is possible to shoot surfaces with a magnification of 10 to 500,000 times and with a resolution of less than 1 to 20 nanometers. By analyzing the information obtained from electron microscopy, we identified and counted the pollen grains and then obtained the plant origin of honeys.

- *Identification and counting*

Using scanning electron microscopy images, pollen grains obtained from honey sediment were counted, counting at least 300 pollen grains is necessary to estimate the relative abundance of pollen types and 500 to 1000 pollen grains are necessary to determine the relative abundance (Behm et al. 1996). We also used pollen atlases and websites to identify pollen, identification of pollen grains was done according to the morphology of pollen grains. Pollen species should only be referred to as botanical species or species if they are reliably identified at the genus or species level, which is rare (Von Der Ohe et al. 2004).

3.2.2. Laboratory studies and chemical analysis of honey

All the reagents for biochemical studies were obtained from Merck Chemical Company. DPPH was obtained from Sigma chemicals.

In this part of the study, some chemical analysis of honey such as antioxidant , total phenol, pH and humidity content were investigated. For all chemical analysis honey was diluted 1/1 (w/v) by distilled water.

- *Analysis of antioxidant by DPPH free radical scavenging activity*

At this stage, the DPPH method was used to analyze all 94 samples of honey (Chua et al.2013), which dissolved a concentration of 0.1 mM of DPPH in methanol and a purple solution was obtained , 100 µl of DPPH was mixed with 10 µl of diluted honey and incubated 30 minutes in dark place. The absorption was recorded at 520 nm by the JENUS V-1100 spectrophotometer. Ascorbic acid (20 mg/ ml) was used as positive control. The antioxidant activity was calculated from the following formula.

$$\text{DPPH scavenging activity (\%)} = A_{\text{control}} - A_{\text{sample}} / A_{\text{control}} \times 100$$

- *Analysis of total phenol*

In this experiment, 1% iron chloride solution $FeCl_3$ 1% was used to measure the amount of total phenol in honey(Athipornchai and Klangmanee, 2021), Phenol with a concentration of 0.09% (mg / kg) was used as a standard sample. 0.1 ml of $FeCl_3$ was mixed with 0.1 ml of diluted honey and the absorbance was recorded at 590 nm. Phenol solution (0.09 %) was used as a standard.

- *Measuring pH and the moisture of honey*

For pH determination, 1 g of honey was mixed with 1 ml of distilled water and the pH of each sample was measured by a pH Meter model JENWAY 3505 and the moisture content of pure honey was determined with a refractometer.

4. Results

4.1. Pollen analysis

In this study, after analyzing pollen grains of 94 honey samples, 42 plant families were identified, that in total, Asteraceae families with 38%, Fabaceae with 13.5%, Rosaceae with 6%, Apocynaceae & Apiaceae with 5% each showed the highest presence (Figure 2) and 55 plant genus (Table 1) were identified.

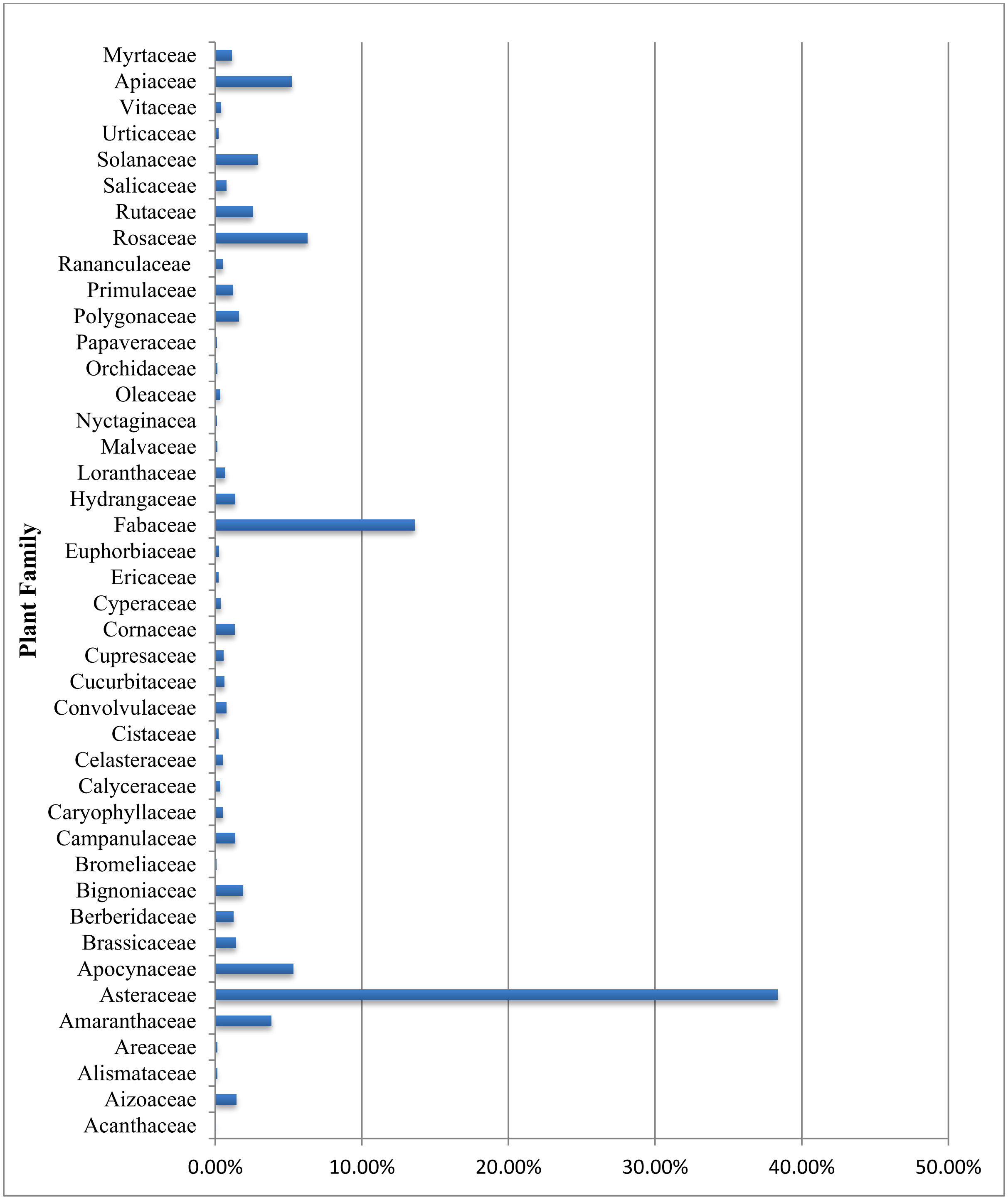

Figure. 2. Percentage of presence of each plant family that was identified through analysis of pollen of 94 honey samples

Table 1. Plant genus identified from pollen analysis of 94 honey samples

Family	Genus
**Amaranthaceae**	*Amaranthus sp.*
	Atriplex type
**Apocynaceae**	*Catharanthus type*
**Apiaceae**	*Apiaceae type*
	Apiaceae type
	Torilis
**Asteraceae**	*Acanthaceae type*
	Achillea sp.
	Artemisia sp.
	Carduus type
	Carthamus type
	Centaurea type
	Cirsium arvense
	Crepis
	Doronicum sp.
	Helichrysum sp.
	Hieracium sp.
	Inula sp.
	Senecio sp.
**Bignoniaceae**	*Bigononiaceae type 1*
	Bignoniaceae type 2
**Brassicaceae**	*Brassicaceae type*
**Caryophyllaceae**	*Caryphyllaceae type*
**Cistaceae**	*Cistus sp.*
**Convolvulaceae**	*Convolvulus arvensis*
**Cornaceae**	*Cornus sanguinea*
**Cucurbitaceae**	*Cucurbit asp.*
**Cyperaceae**	*Carex sp.*

**Fabaceae**	*Astragalus type*
	Robinia pseudacacia
	Hedysarum sp.
	Onobrychis cornuta
	Vicia sp.
**Hydrangeaceae**	*Philadelphus*
**Myrtaceae**	*Eucalyptus calmadonensis*
**Oleaceae**	*Olea europaea*
**Polygonaceae**	*Fagopyrum*
	Polygonum sp.
	Rumex sp.
**Ranunculaceae**	*Adonis sp.*
	Clematis sp.
	Ranunculus sp.
**Rosaceae**	*Alchemilla sp.*
	Cotoneaster sp.
	Sorbaria
	Prunus domestica
	Rosa cania
	Sorbaria sp.
	Spiraea sp.
**Rutaceae**	*Rutaceae type*
	Poncirus
**Salicaceae**	*Populus nigra*
**Solanaceae**	*Solanum spp.*
**Urticaceae**	*Parietaria judaica*
	Urtica dioica

Pollen grains extracted from honey samples were identified based on pollen references. Quantitative evaluation of pollen grains in each of the honey samples was performed according to the Louveaux standard. The collected samples were in the altitude ranges of 12 to 3062 meters above sea level. The samples were collected in 7 altitude intervals, which in terms of vegetation type changes and the location of beekeepers in accordance with the season, more than 73% of the samples were in the altitude range of 1000-3000 meters above sea level (Figure 3).

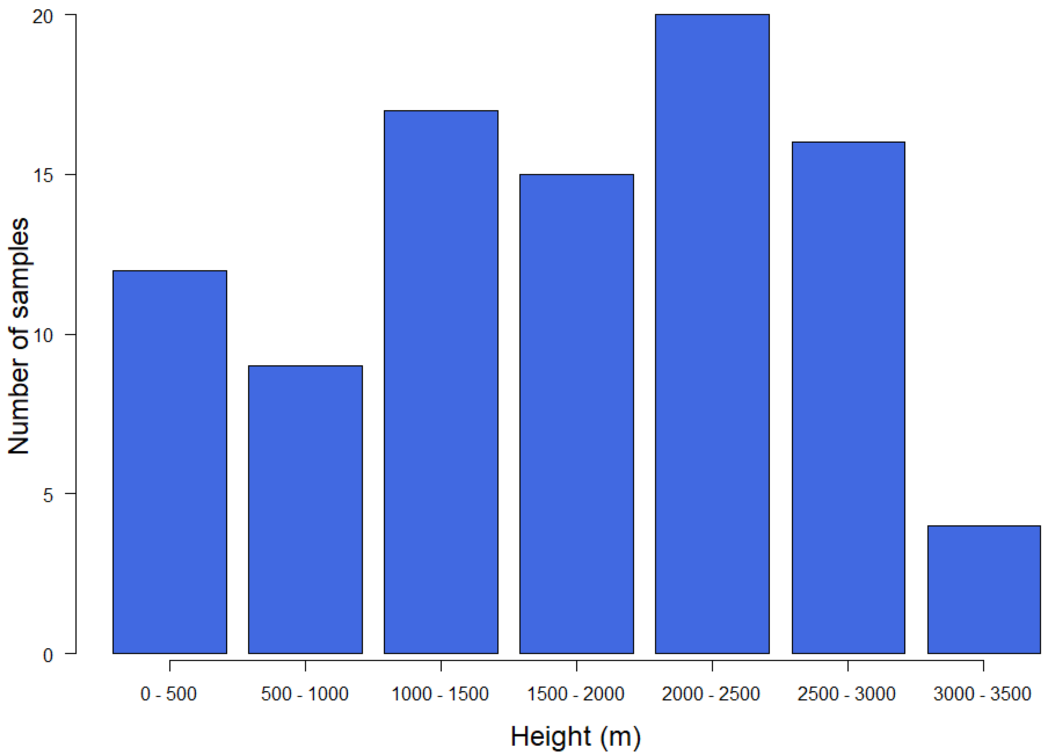

Figure. 3. Number of sampling performed at different altitude intervals

4.2. Physicochemical analysis of honeys

According to the resul[illegible]btained from chemical analysis of honey samples, the amount of antioxidants in the samples was the lowest of 19% and the highest of 98%. According to (Figure 4), more than 36% of the total samples had antioxidants between 40% and 60%.

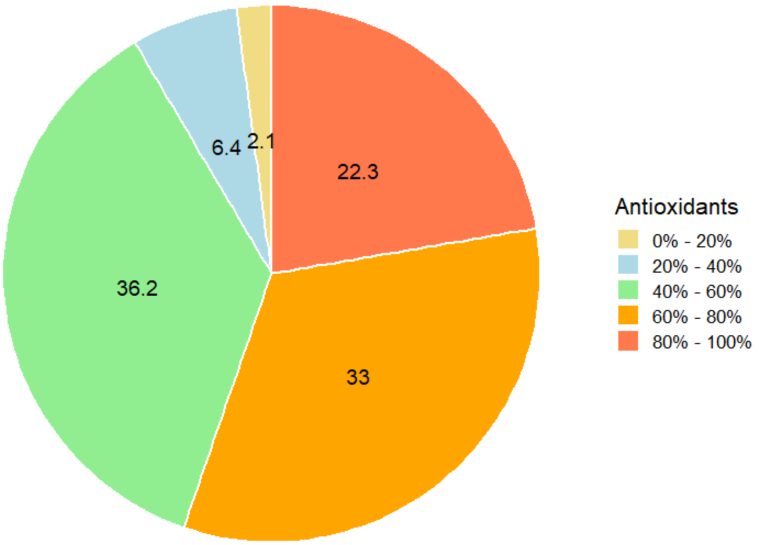

Figure. 4. Antioxidant abundance ratio of honey samples

The total phenol content of honey samples was between 0.08 to 0.59 micrograms. Differences in phenolic content in honey samples may be due to different geographical origins or other factors such as collection season and storage conditions. Also in this section, the pH of each honey sample was examined.

The lowest pH was 1.9 and the highest pH was 5.2. In the information obtained from measuring the moisture content of each honey sample, it was determined that the maximum moisture content was 18.8% and the minimum was 13%. We compared the humidity of honey samples with the humidity of the areas from which the samples were collected, which shows that the humidity of the environment affected the humidity of honey samples (Figure5).

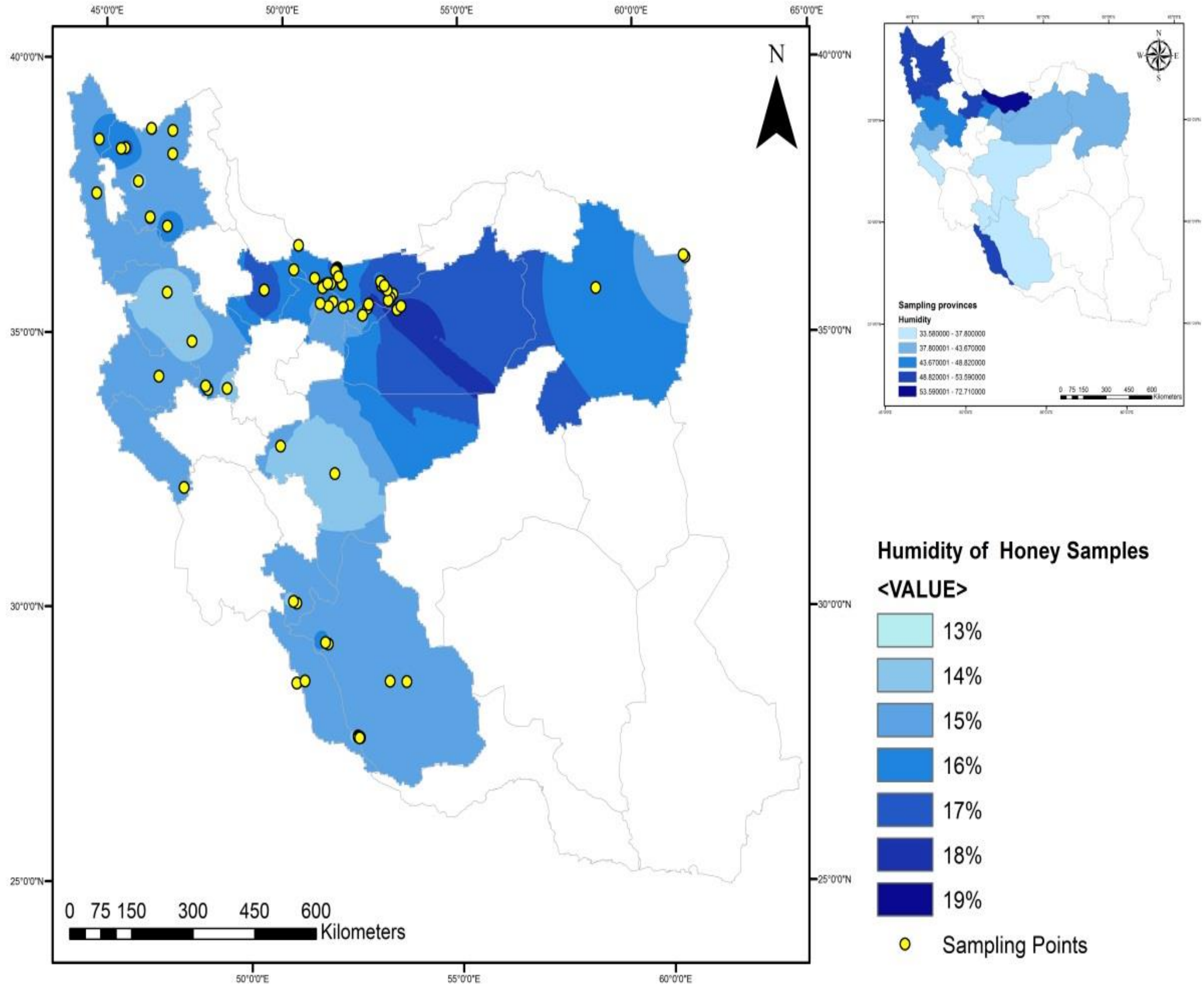

Figure. 5. Comparison of moisture content of honey samples with moisture content of the collection site

All honey samples were grouped with chemical factors obtained from them, which our plot (Figure 6) shows that the factors, pH and total phenol were positively correlated for grouping and factors, humidity and antioxidants for grouping were negatively correlated they had.

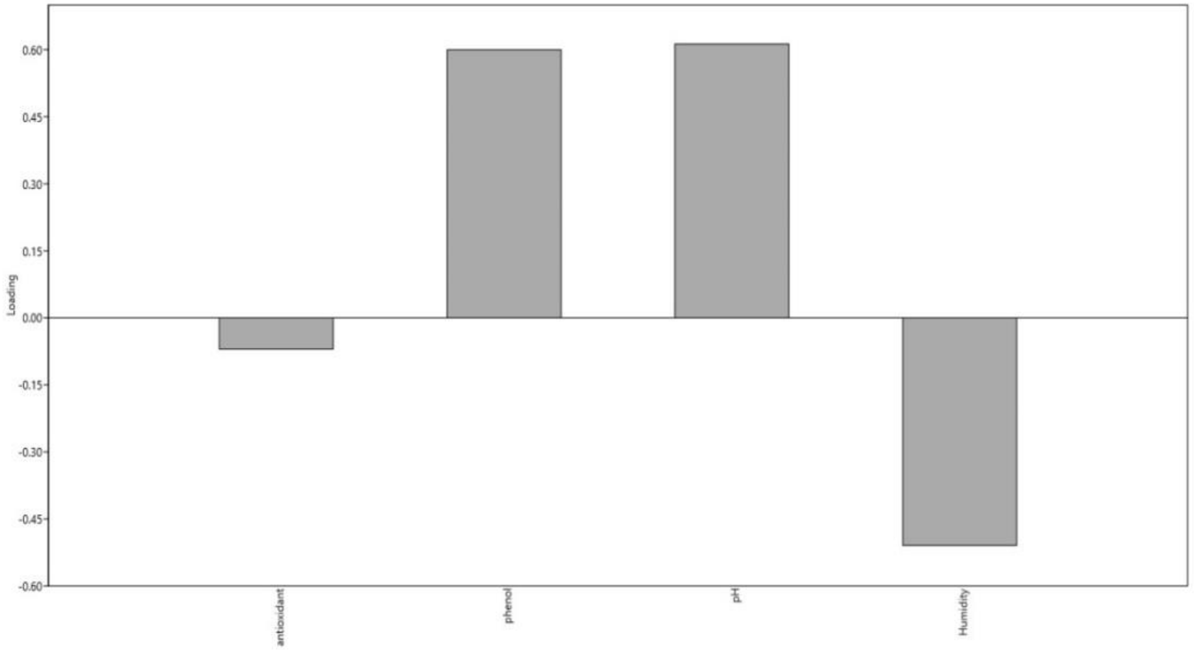

Figure. 6. Plot related to the grouping of total honey samples using chemical factors

Honey samples collected in this study were from different geographical areas, Based on the number of beekeepers present in each region, the number of different honey samples was collected from some areas, 2 samples of honey and some areas, more than 2 samples of honey were collected. The plot obtained (Figure 7) shows that in order to group the samples with their chemical composition, even the samples collected from a geographical area do not have the same chemical composition.

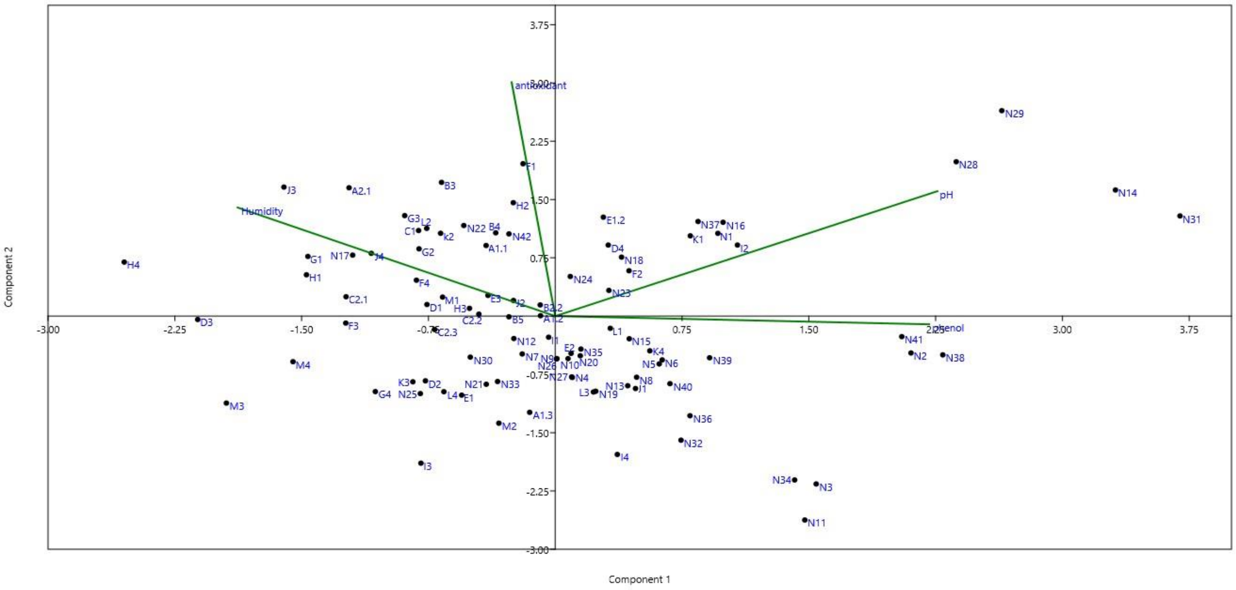

Figure. 7. Grouping the samples with their chemical compounds to show the difference in the amount of chemical compounds between the samples according to their geographical areas

Based on the plant families obtained from each honey sample, we performed a cluster analysis (Figure 8) that shows how close or distant the honey samples were in terms of the composition of plant taxa .

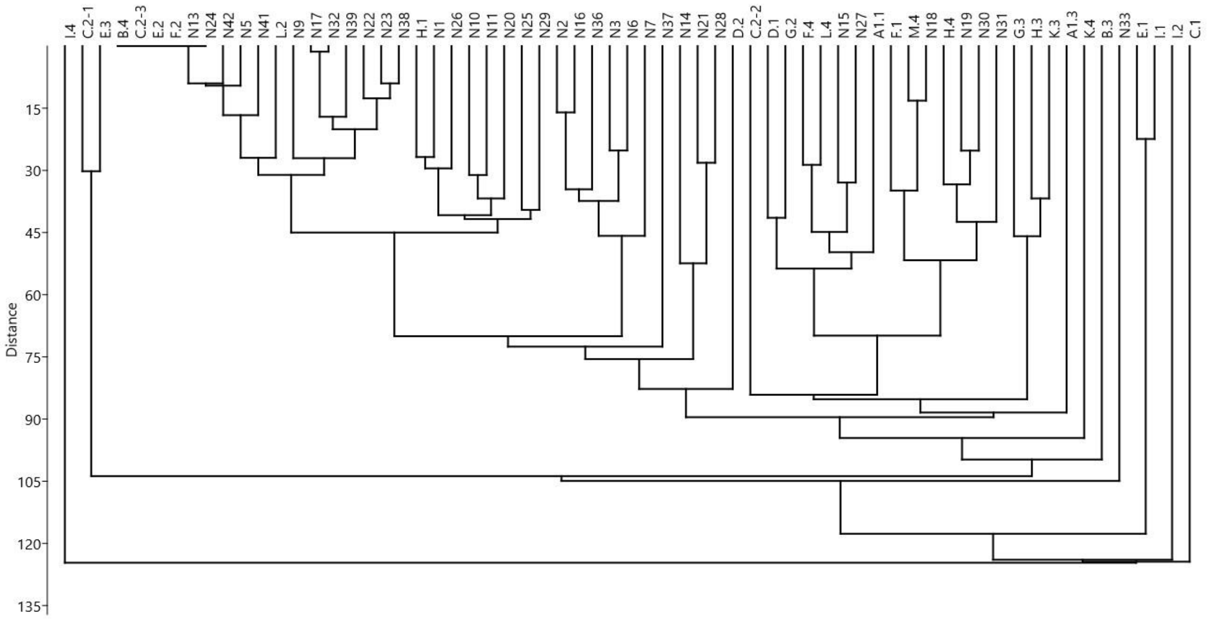

Figure. 8. Cluster analysis to find out how close or distant honey samples were in terms of plant taxon composition

5. Discussion

In this study, 94 samples of honey collected belong to the main honey producing regions in Iran. In the results of this study, 42 plant families and 57 genera of these families were identified. Due to the difficulty of identifying pollen grains extracted from honey at the genus or species level (Von Der Ohe et al.2004), we were able to identify up to the genus level in our studies.

The highest density of pollen grains in honey samples belong to the Asteraceae, Fabaceae, Rosaceae, Apiaceae, Apocynaceae (Figure 9 a,b,c,d). The mentioned frequency showing high similararity to the following studies: Karimi et al. (2016) in Fars province and Razzaqi et al. (2006) in Mazandaran province. However the Asteraceae, Fabaceae, Rosaceae, Apiaceae, Brassicaceae, Papilionaceae, Lamiaceae, showed the highest presence respectiveley, In Rašić et al.(2018).

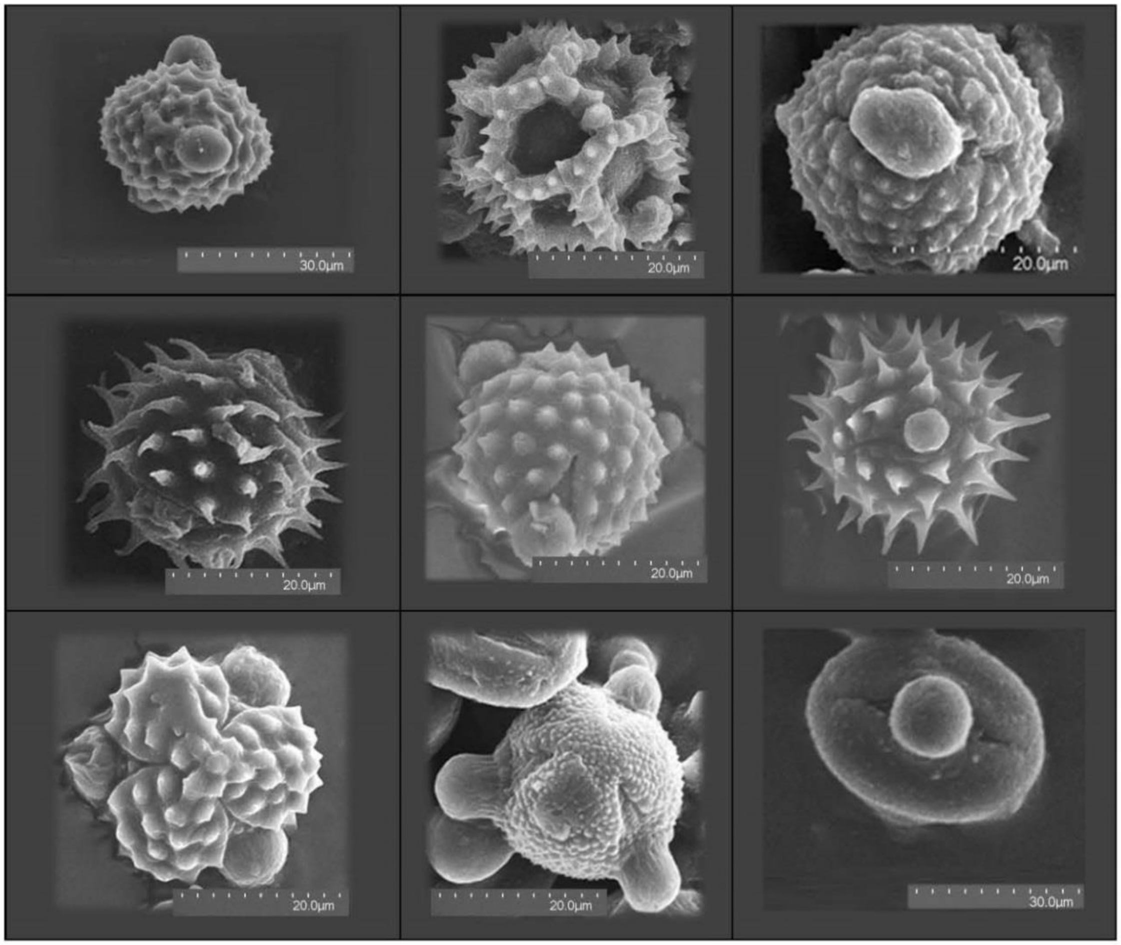

Figure. 9a. Some pollen images of the Asteraceae family

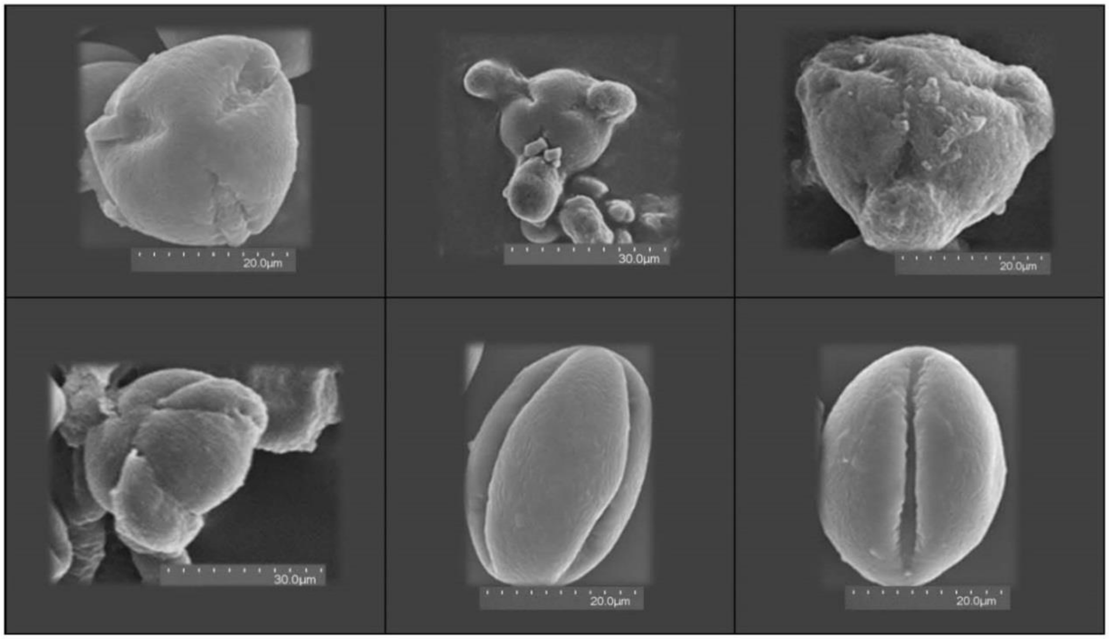

Figure. 9b. Some pollen images of the Rosaceae family

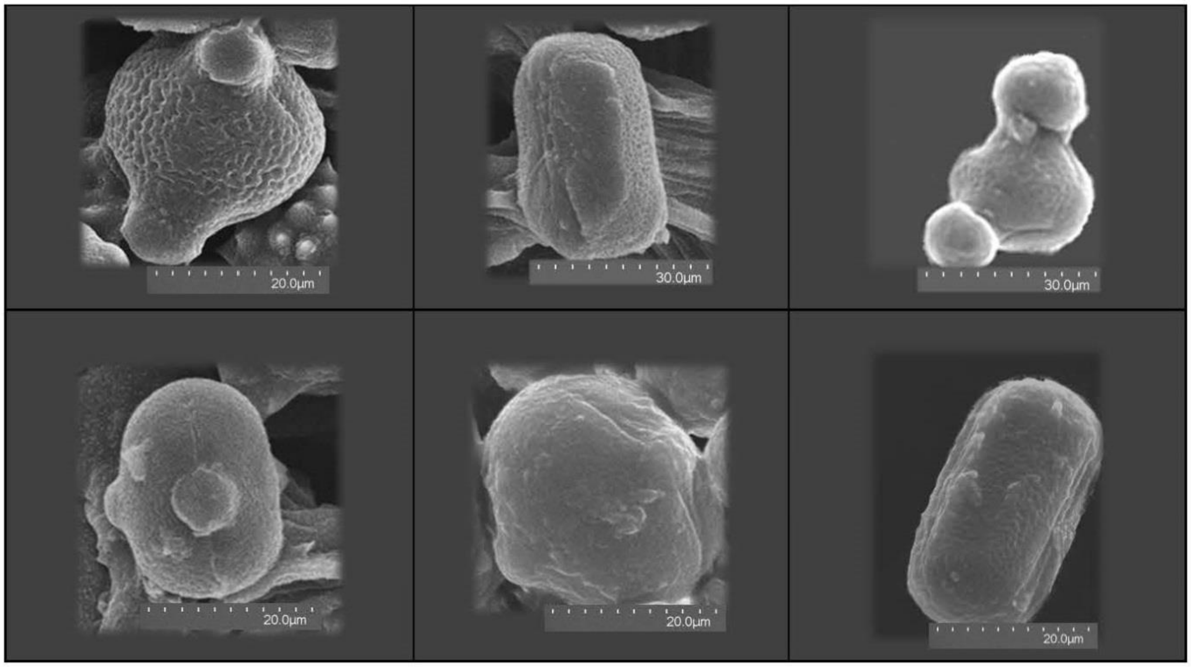

Figure. 9c. Some pollen images of the Fabaceae family

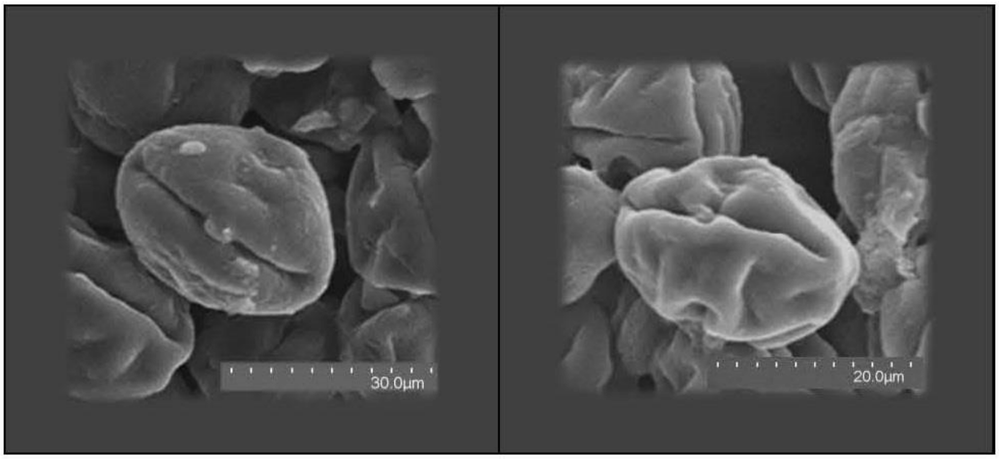

Figure. 9d. some pollen images of the Apocynaceae family

Moreover The Asteraceae, Fabaceae and Rosaceae were the most present, in the Stonia (Puusepp and Koff 2014) Croatia Our results showing all the studied samples are classified as Polyfloral honey. The results of our studies show that according to palinology criteria, about 30% of the honey samples studied are counterfeit, but the rest of the honey is of good quality.

The chemical, physical, antioxidant, and healthy composition of honey varies depending on the flower source, soil type, geological roots, and climatic conditions. [illegible]ACS (2001). Both ancient and modern civilizations use honey as a medicine for various diseases (Berhanu et al.2022). It is also a delicious food that is mostly made of different sugars and compounds such as proteins, organic acids, amino acids, nutrients, minerals, carotenoids and aromatic substances. It is rich in flavonoids and phenolic acids, which show a wide range of biological effects and act as a natural antioxidant (karabagias, et al. 2014; Manyi-Loh 2011). The results of our chemical analysis showed that the chemical composition of honey samples collected from different ecological areas are different from each other (Kuldasheva et al.2022). Chemical experiments also showed that about 3/5 of the samples showed more than 60% of the effect of antioxidant activity. We compared the results of antioxidants with increasing height, which seems to have a positive response to increasing height and up to 3000 meters, the trend line shows an upward trend, but after 3000 meters, antioxidants follow a completely opposite trend. The highest effect of antioxidant activity was recorded in the altitude range of 2500-3000 meters, which showed an average of 88.71% (Figure 10).

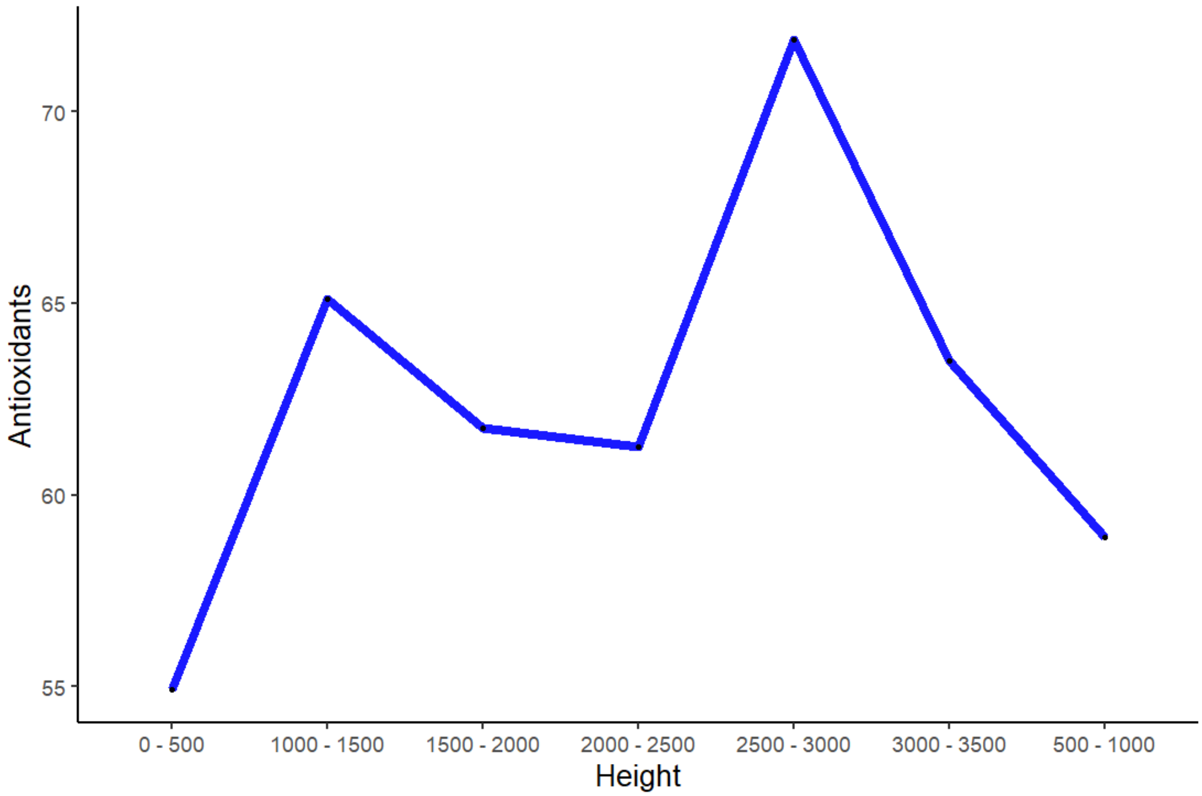

Figure. 10. Comparison of antioxidants with altitude

Variation in the antioxidant potential of honey depends on topographic and botanical differences (Martos et al. 2000), (Yao et al. 2003). The antioxidant activity of honey is mainly due to phenolic and flavonoid compounds. Thus, a significant variation in antioxidant activity is found among different types of honey worldwide (Beretta et al., 2005; Aljadi and Kamaruddin, 2004; Nayik and Nanda, 2016). An important group of compounds that affect the appearance and functional properties of honey are phenolic acids, which have nutritional value and therapeutic properties (Alvarez-Suarez et al., 2010). The type and concentration of phenolic compounds in different honeys are different from each other (Saxena et al. 2010).

The amount of phenolic compounds is influenced by various factors such as plant type, geographical origin and climatic conditions of the honey production site (Escuredo et al. 2012). Determining the amount of phenolic compounds in honey is a suitable factor to evaluate the quality and therapeutic potential of honey (Ouchemoukh et al. 2007). In the results of our analyzes, the samples showed phenolic compounds of 0.08-0.51 µg. Honey is recognized as a

treatment option for the treatment of acute and chronic wounds, and a wide range of in vitro and in vivo research studies have been performed in this field (Subrahmanyam, 1998; Cooper et al. 2002; Johnson et al. 2005). Low pH of honey (0.4 - 5.3) helps wound healing (Efem, 1988; Molan, 1999), which is done by reducing the high alkaline environment in non healing wounds to a more acidic healing environment. The global standard for pH of honey is 3.9 (3.2 – 4.5). Based on the results of our studies, honey samples showed a pH 1.90 - 5.21 and 48% of the total samples are in the pH range of 2.5 - 3.5 (Figure 11).

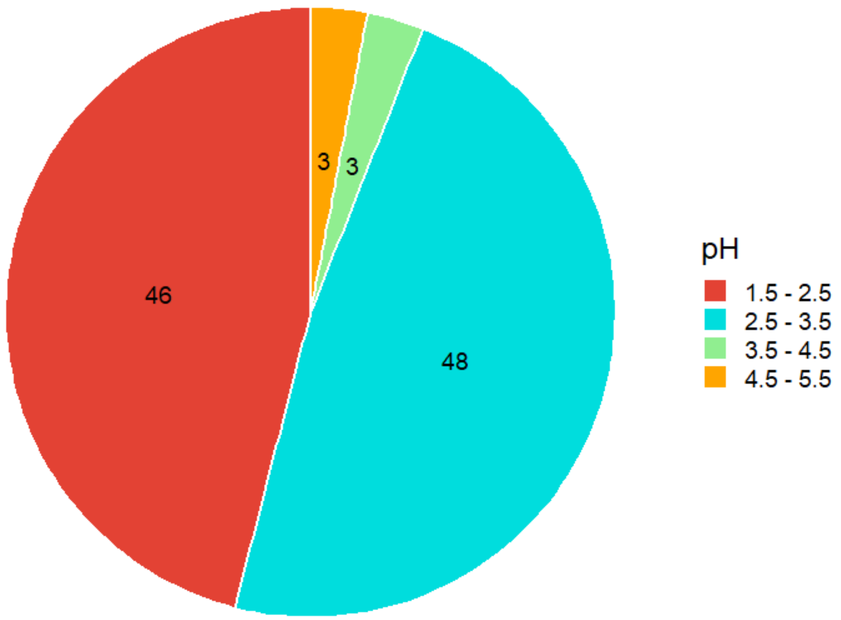

Figure. 11. pH abundance ratio of the samples

And pH values between 3.4 and 6.1 indicate the freshness of honey samples (Khalil et al. 2012; Selvaraju et al. 2019). Moisture content is considered as an important criterion for measuring the quality of honey. Honey with less moisture has a longer shelf life (Fredes and Montenegro, 2006). The moisture content of honey depends on various factors such as harvest time, climatic factors of the geographical area and storage temperature (Nanda et al., 2003; Finola et al., 2007). In this study, all honey samples showed a low moisture content (13% -18.80%), which corresponded to the values (<20 <) set by the Codex standard for honey (Almentarius Codex, 1987). The parameter of quality and uniqueness of honey has been studied by various researchers, this quality and credibility of honey is important for both buyers and producers.

One of the important concerns of honey quality is to guarantee the authenticity of honey according to legal requirements. This authenticity is achieved by recognizing the plant origin, geography and chemical analysis (da Silva et al.2016), (Machado et al.2018) Nutritional and healing properties of honey from the very beginning make it an important component of traditional medicine (Bogdanov 1997; Subrahmanyam 1998). The natural antioxidants in honey are valuable against heart disease, cancer, inflammation, coronary heart disease, aging, tumors, gastrointestinal tract and wound healing (de Rodríguez et al. 2004), (Kečkeš et al.2013) . In relation to health, honey, bee pollen, propolis, royal jelly, beeswax and bee venom have all been used in traditional and modern medicine (Easton-Calabria et al. 2019; Kocot et al. 2018). Researchers have identified the bioactive properties of honey, propolis and royal jelly, indicating compounds with antimicrobial, anti-inflammatory, antioxidant, anti-tumor and anti-cancer activities (Pasupuleti et al. 2017; Kocot et al. 2018; Easton Calabria et al. 2019). Honey is used to treat stomach ulcers and injuries, increase oral health, fight stomach disorders, and liver and pancreatic diseases, as well as to promote cardiovascular health (Pasupuleti et al. 2017; Easton-Calabria et al. 2019) .

6. Conclusion

Melissopalynology is a useful and complementary tool along with chemical assays to measure the authenticity, fraud and differentiation between different types of honey.In addition to being a useful tool in the food industry and therapeutic debates, melissopalynology is even more important in the conservation debate. Conducting more extensive studies in the field of melissopalynology in the future to identify the plant species used by bees is of mutual importance in the conservation debate, plant species conservation protects bees and bee conservation leads to plant species conservation. In the field of conservation, more and more ongoing research is needed to better understand the behavior and role of bees and plants. On the other hand, seasonal changes (including changes in temperature, natural light, precipitation) and changes in vegetation type can not only have a significant impact on the behavior of bees but also the behavior of other animals (Salmanpour et al.2021).

7. Recommendation

It is recommended that future studies on pollen of honey produced by farmed bees be compared to honey produced by wild bees to help better understand the role of melissopalynology in the protection of bees and plant species and the protection of ecosystems. Bee populations are threatened by human activities. Climate change, invasive species, monocultures that have less variety in flower resources, and the use of pesticides have a negative effect on bees. On the other hand, seasonal changes (including changes in temperature, natural light, precipitation) and changes in vegetation type can not only have a significant impact on the behavior of bees but also the behavior of other animals (Salmanpour et al. 2021). An understanding of ecosystem processes and the importance of the presence of bees and plants to preserve the planet raises the concern that biodiversity loss is directly affecting ecosystem performance.

Funding

This research received no external funding.

Acknowledgments

Many thanks to Mr. Saeed Javadi

Many thanks to Mr. Mehdi Kia Hairati

Many thanks to Mr. Mohsen Yasari

Many thanks to Mr. Erfan Rajabi

Many thanks to Mr. Mohammad Reza Kashfi

CRediT authorship contribution statement

Zahra Shakoori: Conceptualization, Principal Investigation, Formal analysis, Writing - Original Draft, Sample collection, Software .

Ahmadreza Mehrabian: Conceptualization, Formal analysis, Writing - Reviewing and editing.

Dariush Minai: Conceptualization, Formal analysis, Writing - Reviewing and editing.

Farid Salmanpour: Conceptualization, Formal analysis, Writing - Reviewing and editing, Sample collection, Software.

Farzaneh Khajoei Nasab: Conceptualization, editing, Software.

Conflicts of Interest

The authors confirm that they have no conflicts of interest with respect to the work described in this manuscript.

DATA AVAILABILITY STATEMENT

https://figshare.com/s/d21d14ac03da862023bd

References

1. Alijani, B., O'Brien, J. and Yarnal, B., 2008. Spatial analysis of precipitation intensity and concentration in Iran. *Theoretical and Applied Climatology*, *94*(1), pp.107-124. https://doi.org/10.1007/s00704-007-0344-y
2. Alijani, B. and Harman, J.R., 1985. Synoptic climatology of precipitation in Iran. *Annals of the Association of American Geographers*, *75*(3), pp.404-416. https://doi.org/10.1111/j.1467-8306.1985.tb00075.x
3. Aljadi, A.M. and Kamaruddin, M.Y., 2004. Evaluation of the phenolic contents and antioxidant capacities of two Malaysian floral honeys. *Food chemistry*, *85*(4), pp.513-518. https://doi.org/10.1016/S0308-8146(02)00596-4
4. Al Kiyumi, E.H., Al Rashdi, B.S., Al Alawi, A.R., Al Balushi, A.A., Al Hooti, S.N., Al Hosni, S.I., Dhanalekshmi, U.M. and Khan, S.A., 2021. Quantification of bioactive components and evaluation of antioxidative potential of different floral origin honey from arid regions of Oman. *Biocatalysis and Agricultural Biotechnology*, *33*, p.102007.
5. Al-Mamary, M., Al-Meeri, A. and Al-Habori, M., 2002. Antioxidant activities and total phenolics of different types of honey. *Nutrition research*, *22*(9), pp.1041-1047.
6. Al Naggar, Y. and El-Sofany, A., 2018. Characterization of Apis mellifera Honey of Different Botanical and Geographical Origins in Egypt. http://dx.doi.org/10.5455/egysebz.20180523104927
7. Alnaqdy, A., Al-Jabri, A., Al Mahrooqi, Z., Nzeako, B. and Nsanze, H., 2005. Inhibition effect of honey on the adherence of Salmonella to intestinal epithelial cells in vitro. *International journal of food microbiology*, *103*(3), pp.347-351. https://doi.org/10.1016/j.ijfoodmicro.2004.11.042
8. Alvarez-Suarez, J.M. ed., 2017. *Bee products-chemical and biological properties*. Springer. https://doi.org/10.1007/978-3-319-59689-1

9. Alvarez-Suarez, M., J., Giampieri, F., & Battino, M.(2013). Honey as a source of dietary antioxidants: structures, bioavailability and evidence of protective effects against human chronic diseases. Current medicinal chemistry, 20(5), pp.621-638. https://doi.org/10.2174/092986713804999358
10. Alvarez-Suarez, J.M., Tulipani, S., Romandini, S., Bertoli, E. and Battino, M., 2010. Contribution of honey in nutrition and human health: a review. *Mediterranean Journal of Nutrition and Metabolism*, *3*(1), pp.15-23. https://doi.org/10.1007/s12349-009-0051-6
11. Amiri, M.J. and Eslamian, S.S., 2010. Investigation of climate change in Iran. *Journal of Environmental Science and Technology*, *3*(4), pp.208-216.
12. Andrade, P.B., Amaral, M.T., Isabel, P., Carvalho, J.C., Seabra, R.M. and da Cunha, A.P., 1999. Physicochemical attributes and pollen spectrum of Portuguese heather honeys. *Food Chemistry*, *66*(4), pp.503-510. https://doi.org/10.1016/S0308-8146(99)00100-4
13. Barthlott, W., Biedinger, N., Braun, G., Feig, F., Kier, G. and Mutke, J., 1999. Terminological and methodological aspects of the mapping and analysis of global biodiversity. *Acta botanica fennica*, *162*(0), pp.103-110.
14. Barthlott, W., Neinhuis, C., Jetter, R., Bourauel, T. and Riederer, M., 1996. Waterlily, poppy, or sycamore: on the systematic position of Nelumbo. *Flora*, *191*(2), pp.169-174. https://doi.org/10.1016/S0367-2530(17)30709-0
15. Basualdo, C., Sgroy, V., Finola, M.S. and Marioli, J.M., 2007. Comparison of the antibacterial activity of honey from different provenance against bacteria usually isolated from skin wounds. *Veterinary microbiology*, *124*(3-4), pp.375-381. https://doi.org/10.1016/j.vetmic.2007.04.039
16. Behm, F., von der Ohe, K. and Henrich, W., 1996. Zuverlässigkeit der Pollen analyse von Honig. Bestimmung Der Pollenhäufigkeit, Dtsch. Lebensm, 92: 183-187.
17. Belay, A., Haki, G.D., Birringer, M., Borck, H., Addi, A., Baye, K. and Melaku, S., 2017. Rheology and botanical origin of Ethiopian monofloral honey. *LWT*, *75*, pp.393-401. https://doi.org/10.1016/j.lwt.2016.09.021
18. Berberian, M. (1983). The southern Caspian: a compressional depression floored by a trapped, modified oceanic crust. *Canadian Journal of Earth Sciences*, *20*(2), 163-183. https://doi.org/10.1139/e83-015

19. Berberian, M. and King, G.C.P., 1981. Towards a paleogeography and tectonic evolution of Iran. *Canadian journal of earth sciences*, *18*(2), pp.210-265. https://doi.org/10.1139/e81-019
20. Beretta, G., Granata, P., Ferrero, M., Orioli, M. and Facino, R.M., 2005. Standardization of antioxidant properties of honey by a combination of spectrophotometric/fluorimetric assays and chemometrics. *Analytica Chimica Acta*, *533*(2), pp.185-191. https://doi.org/10.1016/j.aca.2004.11.010
21. Berhanu, S., Tadesse, D.M. and Jorge, A., 2022. Physico-chemical properties of Ethiopian Apis mellifera Honey: Review. *J Agric Sc Food Technol*, *8*(1), pp.038-044. https://dx.doi.org/10.17352/2455-815X.000143
22. Bogdanov, S., Jurendic, T., Sieber, R. and Gallmann, P., 2008. Honey for nutrition and health: a review. *Journal of the American college of Nutrition*, *27*(6), pp.677-689. https://doi.org/10.1080/07315724.2008.10719745
23. Bogdanov, S. and Martin, P., 2002. Honey authenticity. *Mitteilungen aus Lebensmitteluntersuchung und Hygiene*, *93*(3), pp.232-254.
24. Bogdanov, S., 1997. Nature and origin of the antibacterial substances in honey. *LWT-Food Science and Technology*, *30*(7), pp.748-753. https://doi.org/10.1006/fstl.1997.0259
25. Bryant Jr, V.M. and Jones, G.D., 2001. The r-values of honey: Pollen coefficients. *Palynology*, *25*(1), pp.11-28. https://doi.org/10.1080/01916122.2001.9989554
26. Buba, F., Gidado, A. and Shugaba, A., 2013. Analysis of biochemical composition of honey samples from North-East Nigeria. *Biochem Anal Biochem*, *2*(3), p.139.
27. Cantarelli, M.A., Pellerano, R.G., Marchevsky, E.J. and Camiña, J.M., 2008. Quality of honey from Argentina: Study of chemical composittion and trace elements. *The Journal of Argentine Chemical Society*, *96*(1-2), pp.33-41.
28. Christy, E.M.L. and Anna, M.C., 2011. An overview of honey: Therapeutic properties and contribution in nutrition and human health. *African Journal of Microbiology Research*, *5*(8), pp.844-852. https://doi.org/10.5897/AJMR10.008

29. Chua, L. S., Rahaman, N. L., Adnan, N. A., & Eddie Tan, T. T. (2013). Antioxidant Activity of Three Honey Samples in relation with Their Biochemical Components. *Journal of analytical methods in chemistry*, *2013*, 313798. https://doi.org/10.1155/2013/313798
30. Cianciosi, D., Forbes-Hernández, T.Y., Afrin, S., Gasparrini, M., Reboredo-Rodriguez, P., Manna, P.P., Zhang, J., Bravo Lamas, L., Martínez Flórez, S., Agudo Toyos, P. and Quiles, J.L., 2018. Phenolic compounds in honey and their associated health benefits: A review. *Molecules*, *23*(9), p.2322. https://doi.org/10.3390/molecules23092322
31. Cooper, R.A., Molan, P.C. and Harding, K.G., 2002. The sensitivity to honey of Gram-positive cocci of clinical significance isolated from wounds. *Journal of Applied microbiology*, *93*(5), pp.857-863. https://doi.org/10.1046/j.1365-2672.2002.01761.x
32. Corvucci, F., Nobili, L., Melucci, D. and Grillenzoni, F.V., 2015. The discrimination of honey origin using melissopalynology and Raman spectroscopy techniques coupled with multivariate analysis. *Food chemistry*, *169*, pp.297-304. https://doi.org/10.1016/j.foodchem.2014.07.122
33. da Silva, P.M., Gauche, C., Gonzaga, L.V., Costa, A.C.O. and Fett, R., 2016. Honey: Chemical composition, stability and authenticity. *Food chemistry*, *196*, pp.309-323. https://doi.org/10.1016/j.foodchem.2015.09.051
34. de Rodríguez, G.O., de Ferrer, B.S., Ferrer, A. and Rodríguez, B., 2004. Characterization of honey produced in Venezuela. *Food Chemistry*, *84*(4), pp.499-502. https://doi.org/10.1016/S0308-8146(02)00517-4
35. Djamali, M., Akhani, H., Khoshravesh, R., Andrieu-Ponel, V., Ponel, P. and Brewer, S., 2011. Application of the global bioclimatic classification to Iran: implications for understanding the modern vegetation and biogeography. *Ecologia mediterranea*, *37*(1), pp.91-114. https://www.persee.fr/doc/ecmed_0153-8756_2011_num_37_1_1350
36. Easton-Calabria, A., Demary, K.C. and Oner, N.J., 2019. Beyond pollination: honey bees (Apis mellifera) as zootherapy keystone species. *Frontiers in Ecology and Evolution*, *6*, p.161. https://doi.org/10.3389/fevo.2018.00161
37. Efem, S.E.E., 1988. Clinical observations on the wound healing properties of honey. *British journal of Surgery*, *75*(7), pp.679-681. https://doi.org/10.1002/bjs.1800750718

38. El-Senduny, F.F., Hegazi, N.M., Abd Elghani, G.E. and Farag, M.A., 2021. Manuka honey, a unique mono-floral honey. A comprehensive review of its bioactives, metabolism, action mechanisms, and therapeutic merits. *Food Bioscience*, *42*, p.101038. https://doi.org/10.1016/j.fbio.2021.101038
39. Escuredo, O., Silva, L.R., Valentão, P., Seijo, M.C. and Andrade, P.B., 2012. Assessing Rubus honey value: Pollen and phenolic compounds content and antibacterial capacity. *Food Chemistry*, *130*(3), pp.671-678. https://doi.org/10.1016/j.foodchem.2011.07.107
40. Fagih, A.R., Ebadi, R. & Nazarian, H. 2004. Pollen study of flowering plants used by bees in the regions Khansar and Faridan of Isfahan province. Journal of Iran Agriculture Science 35(2): 265-283. https://eurekamag.com/research/004/024/004024250.php
41. Farhadinia, M.S., Soofi, M., Rosen, T., Moghadas, P., Hobeali, K., Behnoud, P., Amanov, A., Dieterich, T., Hojamuradov, H., Hudaikuliev, N., Karryeva, s., Kaczensky, P., Mengliev, s., Muhashov, A., Nurmuhambetov, Z., Pestov, M., Potaeva, A., Terentyev, V., Salmanpour, F., Tizrouyan, H., Alinezhad, H., Jafari, B., Ommatmohammadi, M., Rabie, K., Veyisov, A., Linnell, J., 2022. Status of Persian leopards in northern Iran and Central Asia. CATnews Special. https://www.researchgate.net/publication/362889852
42. Finola, M.S., Lasagno, M.C. and Marioli, J.M., 2007. Microbiological and chemical characterization of honeys from central Argentina. *Food chemistry*, *100*(4), pp.1649-1653. https://doi.org/10.1016/j.foodchem.2005.12.046
43. Fredes, C. and Montenegro, G., 2006. Heavy metal and other trace elements contents in honey bee in Chile. Cien. Inv. Agr.(in English) 33 (1): 50-58. *International Journal of Agriculture and Natural Resources*, *33*(1), pp.50-58. https://revistaschilenas.uchile.cl/handle/2250/10.7764/rcia.v33i1.328
44. García, N.L., 2018. The current situation on the international honey market. *Bee World*, *95*(3), pp.89-94. https://doi.org/10.1080/0005772X.2018.1483814
45. Hailu, D. and Belay, A., 2020. Melissopalynology and antioxidant properties used to differentiate Schefflera abyssinica and polyfloral honey. *Plos one*, *15*(10), p.e0240868. https://doi.org/10.1371/journal.pone.0240868

46. Hedge, I. C., & Wendelbo, P. (1978). Patterns of distribution and endemism in Iran. *Notes Roy. Bot. Gard. Edinburgh*, *36*(2), 441-464. https://www.scopus.com/record/display.uri?eid=2-s2.0-0006003564&origin=inward&txGid=08c1677fbb653bcda02974e2f9bd74ed
47. Jones, G.D. and Bryant Jr, V.M., 1996. New Frontiers in Palynology. *Palynology: Principles and Applications, edited by J. Jansonius and DC McGregor*, pp.933-38. https://scholar.google.com/scholar?hl=en&as_sdt=0%2C5&q=New+Frontiers+in+Palynology.+Palynology%3A+Principles+and+Applications%2C+edited+by+J.+Jansonius+and+DC+McGregor.&btnG=
48. Johnson, D.W., van Eps, C., Mudge, D.W., Wiggins, K.J., Armstrong, K., Hawley, C.M., Campbell, S.B., Isbel, N.M., Nimmo, G.R. and Gibbs, H., 2005. Randomized, controlled trial of topical exit-site application of honey (Medihoney) versus mupirocin for the prevention of catheter-associated infections in hemodialysis patients. *Journal of the American Society of Nephrology*, *16*(5), pp.1456-1462. https://doi.org/10.1681/ASN.2004110997
49. Karabagias, I.K., Badeka, A., Kontakos, S., Karabournioti, S. and Kontominas, M.G., 2014. Characterisation and classification of Greek pine honeys according to their geographical origin based on volatiles, physicochemical parameters and chemometrics. *Food chemistry*, *146*, pp.548-557. https://doi.org/10.1016/j.foodchem.2013.09.105
50. Karimi, A., Nazarian, H., Jafari, E. and Hatami, A., 2016. Identification and determine the distribution of plants used by bees in Galehdar watershed (Fars province). *Natural Ecosystems of Iran*, *7*(3), pp.13-27. https://neijournal.nour.iau.ir/mobile/article_527674.html?lang=en
51. Kaškonienė, V. and Venskutonis, P.R., 2010. Floral markers in honey of various botanical and geographic origins: a review. *Comprehensive reviews in food science and food safety*, *9*(6), pp.620-634. https://doi.org/10.1111/j.1541-4337.2010.00130.x
52. Kečkeš, S., Gašić, U., Veličković, T.Ć., Milojković-Opsenica, D., Natić, M. and Tešić, Ž., 2013. The determination of phenolic profiles of Serbian unifloral honeys using ultra-high-performance liquid chromatography/high resolution accurate mass spectrometry. *Food Chemistry*, *138*(1), pp.32-40.

https://doi.org/10.1016/j.foodchem.2012.10.025

53. Kędzierska-Matysek, M., Stryjecka, M., Teter, A., Skałecki, P., Domaradzki, P. and Florek, M., 2021. Relationships between the content of phenolic compounds and the antioxidant activity of polish honey varieties as a tool for botanical discrimination. *Molecules*, *26*(6), p.1810. https://doi.org/10.3390/molecules26061810

54. Khalil, M.I., Moniruzzaman, M., Boukraâ, L., Benhanifia, M., Islam, M.A., Islam, M.N., Sulaiman, S.A. and Gan, S.H., 2012. Physicochemical and antioxidant properties of Algerian honey. *Molecules*, *17*(9), pp.11199-11215. https://doi.org/10.3390/molecules170911199

55. Khansaritoreh, E., Salmaki, Y., Azirani, T.A., Henareh, F., Alizadeh, K., Ramezani, E., Zarre, S., Beckh, G. and Behling, H., 2021. The sources and quality of Iranian honey. *Heliyon*, *7*(4), p.e06651. https://doi.org/10.1016/j.heliyon.2021.e06651

56. Kier, G., Mutke, J., Dinerstein, E., Ricketts, T.H., Küper, W., Kreft, H. and Barthlott, W., 2005. Global patterns of plant diversity and floristic knowledge. *Journal of Biogeography*, *32*(7), pp.1107-1116. https://doi.org/10.1111/j.1365-2699.2005.01272.x

57. Klein, J.C. and Lacoste, A., 1989. The oak groves of Quercus macranthera F. and M. in the Alborz mountain (Iran) and the eastern extremities of the Great and Little Caucasus. *Ecologia Mediterranea (France)*. https://scholar.google.com/scholar?hl=en&as_sdt=0%2C5&q=%5BThe+oak+groves+of+Quercus+macranthera+F.+and+M.+in+the+Alborz+mountain+%28Iran%29+and+the+eastern+extremities+of+the+Great+and+Little+Caucasus&btnG=

58. Klein, B. and Leffler, K.B., 1981. The role of market forces in assuring contractual performance. *Journal of political Economy*, *89*(4), pp.615-641. https://doi.org/10.1086/260996

59. Kocot, J., Kiełczykowska, M., Luchowska-Kocot, D., Kurzepa, J. and Musik, I., 2018. Antioxidant potential of propolis, bee pollen, and royal jelly: possible medical application. *Oxidative medicine and cellular longevity*, *2018*. https://doi.org/10.1155/2018/7074209

60. Kortesniemi, M., Rosenvald, S., Laaksonen, O., Vanag, A., Ollikka, T., Vene, K. and Yang, B., 2018. Sensory and chemical profiles of Finnish honeys of different botanical origins and consumer preferences. *Food Chemistry*, *246*, pp.351-359. https://doi.org/10.1016/j.foodchem.2017.10.069
61. Kuldasheva, F., Madartov, B., Nurmurodova, N. and Eshdavlatov, O., 2022. STUDY OF ORGANOLEPTIC, PHYSICAL AND CHEMICAL INDICATORS OF HONEY IN TASHKENT REGION. *Agro Inform*, (1), pp.43-51. https://cyberleninka.ru/article/n/study-of-organoleptic-physical-and-chemical-indicators-of-honey-in-tashkent-region
62. Louveaux, J., Maurizio, A., & Vorwohl, G. (1978). Methods of melissopalynology. Bee world, 59(4), 139-157. https://doi.org/10.1080/0005772X.1978.11097714
63. Louveaux, J. and Maurizio, A., G. vorwohl. 1970. Methods of melissopalynology. *Bee world*, *51*, pp.25-138. https://doi.org/10.1080/0005772X.1970.11097312
64. Machado De-Melo, A.A., Almeida-Muradian, L.B.D., Sancho, M.T. and Pascual-Maté, A., 2018. Composition and properties of Apis mellifera honey: A review. *Journal of apicultural research*, *57*(1), pp.5-37. https://doi.org/10.1080/00218839.2017.1338444
65. Manzanares, A.B., García, Z.H., Galdón, B.R., Rodríguez-Rodríguez, E.M. and Romero, C.D., 2017. Physicochemical characteristics and pollen spectrum of monofloral honeys from Tenerife, Spain. *Food chemistry*, *228*, pp.441-446. https://doi.org/10.1016/j.foodchem.2017.01.150
66. Martos, I., Ferreres, F., Yao, L., D'Arcy, B., Caffin, N. and Tomás-Barberán, F.A., 2000. Flavonoids in monospecific Eucalyptus honeys from Australia. *Journal of Agricultural and Food Chemistry*, *48*(10), pp.4744-4748. https://doi.org/10.1021/jf000277i
67. Maurizio, A., 1975. Microscopy of honey. *Honey: A Comprehensive Survey. E. Crane, ed.* https://scholar.google.com/scholar?hl=en&as_sdt=0%2C5&q=Maurizio%2C+A.+1975.+Microscopy+of+honey.+%E2%80%93+In%3A+Honey%3A+&btnG=
68. Mehrabian, A. and Rad, M.A., 2021. Distribution patterns and priorities for conservation of Iranian Endemic Monocots: determining the Areas of Endemism (AOEs). *Journal of Wildlife and Biodiversity*, *5*(2), pp.69-87. https://doi.org/10.22120/jwb.2020.136616.1188

69. Memariani, F. & Ghoraishi Alhossein, J. 2000. Pollen study of some samples of honey in Khorasan province. Pajohesh va Sazandegi 49: 76-83.

70. Menad, H., Ben-Naoum, F. and Amine, A., 2020. A Hybrid Grey Wolves Optimizer and Convolutional Neural Network for Pollen Grain Recognition. *International Journal of Swarm Intelligence Research (IJSIR)*, *11*(3), pp.49-71. https://doi.org/10.4018/IJSIR.2020070104

71. Molan, P., 2001. Why honey is effective as a medicine: 2. The scientific explanation of its effects. *Bee world*, *82*(1), pp.22-40. https://doi.org/10.1080/0005772X.2001.11099498

72. Mureșan, C.I., Cornea-Cipcigan, M., Suharoschi, R., Erler, S. and Mărgăoan, R., 2022. Honey botanical origin and honey-specific protein pattern: Characterization of some European honeys. *LWT*, *154*, p.112883. https://doi.org/10.1016/j.lwt.2021.112883

73. Naqinezhad, A. and Esmailpoor, A., 2017. Flora and vegetation of rocky outcrops/cliffs near the Hyrcanian forest timberline in the Mazandaran mountains, northern Iran. *Nordic Journal of Botany*, *35*(4), pp.449-466. https://doi.org/10.1111/njb.01384

74. Nanda, V., Sarkar, B.C., Sharma, H.K. and Bawa, A.S., 2003. Physico-chemical properties and estimation of mineral content in honey produced from different plants in Northern India. *Journal of Food Composition and Analysis*, *16*(5), pp.613-619. https://doi.org/10.1016/S0889-1575(03)00062-0

75. Nayik, G.A. and Nanda, V., 2016. Application of response surface methodology to study the combined effect of temperature, time and pH on antioxidant activity of cherry (Prunus avium) honey. *Polish Journal of Food and Nutrition Sciences*, *66*(4), pp.287-293. https://doi.org/10.1515/pjfns-2015-0055

76. Nazarian, H., Sanei Shariat Panahi, M. & tahmasebi, G.H. 1997. Determination of Identification of plants used by bees in Tehran province. The second Conference on Honey bee. Iran Animal Science Research Institute.

77. Noroozi, J. ed., 2020. *Plant biogeography and vegetation of high mountains of Central and South-West Asia*. Springer International Publishing. https://doi.org/10.1007/978-3-030-45212-4

78. Noroozi, J., Pauli, H., Grabherr, G. and Breckle, S.W., 2011. The subnival–nival vascular plant species of Iran: a unique high-mountain flora and its threat from climate warming. *Biodiversity and Conservation*, *20*(6), pp.1319-1338. https://doi.org/10.1007/s10531-011-0029-9
79. Olivieri, C., Marota, I., Rollo, F. and Luciani, S., 2012. Tracking plant, fungal, and bacterial DNA in honey specimens. *Journal of Forensic Sciences*, *57*(1), pp.222-227. https://doi.org/10.1111/j.1556-4029.2011.01964.x
80. Ouchemoukh, S., Louaileche, H. and Schweitzer, P., 2007. Physicochemical characteristics and pollen spectrum of some Algerian honeys. *Food control*, *18*(1), pp.52-58. https://doi.org/10.1016/j.foodcont.2005.08.007
81. Ozturk, F.; Erkan, C. Bee plants of Van Lake Basin (Turkey). *Int. J. Bot.* **2010**, *6*, 101–106. https://www.cabdirect.org/cabdirect/abstract/20103306252
82. Pasupuleti, V.R., Sammugam, L., Ramesh, N. and Gan, S.H., 2017. Honey, propolis, and royal jelly: a comprehensive review of their biological actions and health benefits. *Oxidative medicine and cellular longevity*, *2017*. https://doi.org/10.1155/2017/1259510
83. Ponnuchamy, R., Bonhomme, V., Prasad, S., Das, L., Patel, P., Gaucherel, C., Pragasam, A. and Anupama, K., 2014. Honey pollen: using melissopalynology to understand foraging preferences of bees in tropical South India. *PLoS One*, *9*(7), p.e101618. https://doi.org/10.1371/journal.pone.0101618
84. Puusepp, L. and Koff, T., 2014. Pollen analysis of honey from the Baltic region, Estonia. *Grana*, *53*(1), pp.54-61. https://doi.org/10.1080/00173134.2013.850532
85. Rao, P.V., Krishnan, K.T., Salleh, N. and Gan, S.H., 2016. Biological and therapeutic effects of honey produced by honey bees and stingless bees: a comparative review. *Revista Brasileira de Farmacognosia*, *26*, pp.657-664. https://doi.org/10.1016/j.bjp.2016.01.012
86. Rašić, S., Štefanić, E., Antunović, S., Jović, J. and Kristek, S., 2018. Pollen analysis of honey from north-eastern Croatia. *Poljoprivreda*, *24*(2), pp.43-49. https://doi.org/10.18047/poljo.24.2.6
87. Razzaghy Kamroudi Mehdi, Sanei Shariat Panahi Mohammad, Nazarian Hassan, Qelichnia Hassan. Identification of pollens in honeys of Nourrud watershed of Mazandaran. Research

and construction. 2006 [cited 2022May14]; 19 (3 (p. 72) on livestock and aquaculture): 74-83.
88. Ronsisvalle, S., Lissandrello, E., Fuochi, V., Petronio Petronio, G., Straquadanio, C., Crascì, L., Panico, A., Milito, M., Cova, A.M., Tempera, G. and Furneri, P.M., 2019. Antioxidant and antimicrobial properties of Casteanea sativa Miller chestnut honey produced on Mount Etna (Sicily). *Natural product research*, *33*(6), pp.843-850. https://doi.org/10.1080/14786419.2017.1413568
89. Safi Ahmad Abad, M., Shirvany, A., Panahi, P. (2017). 'Comparison of pollen grain composition and quality of honey obtained from forest and rangeland sites in Arasbaran region', *Iranian Journal of Forest and Poplar Research*, 25(2), pp. 342-353. doi: 10.22092/ijfpr.2017.111783 https://dx.doi.org/10.22092/ijfpr.2017.111783
90. Salmanpour, F., Shakoori, Z., Kia, M. and Ghomi, S., 2021. Investigating the Factors Affecting Leopard (Panthera pardus tulliana) Conflict with Domestic Livestock Compared to other Large Carnivores in Nowshahr Hyrcanian Forests. *Journal of Animal Environment*, *13*(2), pp.1-8. doi: 10.22034/AEJ.2021.135105 https://www.aejournal.ir/article_135105.html?lang=en
91. Sanei Shariat Panahi, M. & Said Abadi, V. 1974. Nectriferous plants in Karaj based on palynology. Journal of Agricultural Faculty, 2-3: 35-47.
92. Saxena, S., Gautam, S. and Sharma, A., 2010. Physical, biochemical and antioxidant properties of some Indian honeys. *Food chemistry*, *118*(2), pp.391-397. https://doi.org/10.1016/j.foodchem.2009.05.001
93. Selvaraju, K., Vikram, P., Soon, J.M., Krishnan, K.T. and Mohammed, A., 2019. Melissopalynological, physicochemical and antioxidant properties of honey from West Coast of Malaysia. *Journal of food science and technology*, *56*(5), pp.2508-2521. https://doi.org/10.1007/s13197-019-03728-3
94. Shakoori, Z., Mehrabian, A., Salmanpour, F., 2022. Evaluation of plant origin of honey based on pollen evidence in some prominent honey-producing zones in Iran, 47th Apimondia Congress (0355), pp-124Ref.0355. https://www.researchgate.net/publication/363134174
95. Stocklin, J., 1977. Structural correlation of the Alpine ranges between Iran and Central Asia.https://pascal-

francis.inist.fr/vibad/index.php?action=getRecordDetail&idt=PASCALGEODEBRGM7720435256

96. Stöcklin, J., 1974. Northern iran: Alborz mountains. *Geological Society, London, Special Publications*, *4*(1), pp.213-234. https://doi.org/10.1144/GSL.SP.2005.004.01.12

97. Subrahmanyam, M., 1998. A prospective randomised clinical and histological study of superficial burn wound healing with honey and silver sulfadiazine. *Burns*, *24*(2), pp.157-161. https://doi.org/10.1016/S0305-4179(97)00113-7

98. Tucak, Z.; Tucak, A.; Puškadija, Z.; Tucak, M. Nutritious healing composition of some kinds of honey in Eastern Croatia. *Agriculture* **2000**, *6*, 129–132.

99. Vaghefi, S.A., Keykhai, M., Jahanbakhshi, F., Sheikholeslami, J., Ahmadi, A., Yang, H. and Abbaspour, K.C., 2019. The future of extreme climate in Iran. *Scientific reports*, *9*(1), pp.1-11. https://doi.org/10.1038/s41598-018-38071-8

100. Von Der Ohe, W., Oddo, L.P., Piana, M.L., Morlot, M. and Martin, P., 2004. Harmonized methods of melissopalynology. *Apidologie*, *35*(Suppl. 1), pp.S18-S25. DOI: 10.1051/apido:2004050 https://www.apidologie.org/articles/apido/pdf/2004/06/MHS07.pdf

101. Yao, L., Datta, N., Tomás-Barberán, F.A., Ferreres, F., Martos, I. and Singanusong, R., 2003. Flavonoids, phenolic acids and abscisic acid in Australian and New Zealand Leptospermum honeys. *Food Chemistry*, *81*(2), pp.159-168. https://doi.org/10.1016/S0308-8146(02)00388-6

Books:

102. Davis SD, Heywood VH, Hamilton AC. Centres of plant diversity. Natural History. 1994;111(1):1-0.

103. Dewan, M.L. and Famouri, J., 1964. *The soils of Iran*. Food and Agriculture Organization of the United Nations.

104. Ghelich Nia, H. 2017. Vegetation types of Mazandaran. Research Institute of Forests and Rangelands.

105. Fayaz, M. 2015. Vegetation types of Tehran. Research Institute of Forests and Rangelands.

106. Stöcklin, J., 1968. Salt deposits of the Middle East.

107. Takhtajan, A. 1986: Floristic regions of the world. - Berkeley: University of California Press.Volume 7, Issue 4, April 2021, e06651.

108. Zahran, M.A., 2010. *Climate-Vegetation:: Afro-Asian Mediterranean and Red Sea Coastal Lands* (Vol. 4). Springer Science & Business Media.

109. Zimmerman, M.I.C.H.A.E.L., 1988. Nectar production, flowering phenology, and strategies for pollination. *Plant reproductive ecology: patterns and strategies*, *41*, pp.157-178.

110. Zohary, M., 1973. *Geobotanical foundations of the Middle East*. Fischer.

Thesis:

111. Dewan ML, Famouri J. *The Soils of Iran, FAO, Rome* (Doctoral dissertation, Thesis presented to the)1963.

112. Klein R. *The new politics of the National Health Service* (Doctoral dissertation, Texas Tech University),1972.

Website:

113. UNESCO (United Nations Educational Scientific and Cultural Organization). 2019. https://www.whc.unesco. org/en/list/1584. Hyrcanian Forests.

114. FAOSTAT, 2018. Production of Natural Honey in World. http://www.fao.org/faostat/en/#data/QL/visualize

نسخه خطی
Click here to access/download;Manuscript;Manuscript.pdf

Assessing the Quality of Bee Honey on the basis of melissopalynology as well chemical analysis: a geobotanic approach

Zahra Shakoori[1], Ahmadreza Mehrabian[*1], Dariush Minai[1], Farid Salmanpour[2], Farzaneh Khajoei Nasab[1]

1. Department of Plant Science and Biotechnology, Faculty of Science and Biotechnology, Shahid Beheshti University, Tehran, Iran, Email: zahra_shakoori.eco@yahoo.com, https://orcid.org/0000-0003-3057-8981
   a_mehrabian@sbu.ac.ir
   d_mtehrani@sbu.ac.ir
   Farzaneh.khajoei@yahoo.com
2. Department of Biodiversity and Ecosystem Management, Research Institute of Environmental Sciences, Shahid Beheshti University, Tehran, Iran, Email: farid.salmanpour@yahoo.com, https://orcid.org/0000-0002-1517-9061

Abstract

Melissopalynological analysis and chemical composition of honey provide us with useful and valuable information about the botanical and geographical origin of honey. The data in question is important for authentication as well as quality testing of honey, so it is considered as a key method in honey regulation Here, we used melissopalynology and chemical analysis to evaluate different honey samples from two main Iranian hubs of honey. Sampling was carried out at two important poles of Iranian honey production, the central Alborz region, and the mountain ecosystems of Zagros in the years 2020-2021. Therefore, 52 samples from Alborz (N. Iran) as well as 42 samples from the Zagrosian ecosystems (western Iran) belonging to different ecologically habitats were collected. In addition, samples were taken at 7 altitudinal levels between 0-3500 m a.s.l. Furthermore in this study, various chemical analyzes such as the effect of antioxidant activity, the amount of phenolic compounds, the pH, and moisture content of honey samples were evaluated. Our results showed that all honey samples were classified as polyfloral honey. Based on our findings, 57 honey samples (61%) contained the standard amount of pollen . A total of 42 plant families and 55 genera were identified in the studied samples, with the highest presence of Asteraceae, Fabaceae, Rosaceae, Apiaceae, and Apocynaceae. Finally, the antioxidant activity rate was 19% -to 98%, phenolic compounds from 0.08 to -0.51 ppm, pH from 1.90 to -5.21, and humidity from 13% -to 18.40%.

Keywords: Melissopalynology, Botanical origin, Scanning Electron Microscopy (SEM), Iran, Pollen, palynology.

1. Introduction

Honey is a precious natural product processed by bees from the nectar of flowers, and is stored in the hive. Honey production in an area depends mainly on the vegetation and climate of thate region, as the flowering season and nectar production can vary for a species in different regions (Zimmerman, 1988). With the developing the of the honey market through the world identification of botanical origin as well as its authenticity has received a lot of attention in international societies (Kaskonine & Venskutonis, 2010). Fake honey and poor-quality honey are major problems in the global honey trade. Honey, which is composed, of many stable elements as well as pollen grains, collected by bees along with nectar. Melissopalynology as an effective method as well as key criteria has been widely used to determine the purity, geographical and botanical origins of natural honey (Maurizo 1975, ; Alvarez-Suarez 2017). Louveaux (1970, 1978) first founded this method. However, several laboratory procedures have been developed to study of pollen grains in honey latter (Bryant & Jones 2001). Melissopalynology studies evaluated pollen grains in honey (Jones and Bryant 1996 & Louveaux et al. 1970 & Corvucci et al. 2015 & Menad et al.2020). Recent analyzes represent valuable data on the botanical and geographical origins of honey. Also, a major authentication method is honey sorting (Shakoori et al.2022). Furthermore, the botanaical and geographical origin of honey is important to consumers and influences the quality of the product (Alnaqdy et al. 2005; Basualdo et al. 2007; Bogdanov et al. 2008). In addition, the botanical origin of honey influences the physical, chemical, organoleptic and bioactive properties of honey (Kortesniemi et al. 2018) which is considered as a valuable indicator of quality and authenticity of honey. Furthermore, the quality of honey mainly depends on the nectariferous plant taxa that honey bees use in their diet . Accordingly, different melliferous plant species have different characteristics as well as different applications (e.g. drugs, nutrients, etc.)(Ozturk and Ercan 2010, Tucak et al. 2000,).

Iran was ranked 9th in the world or honey production from 1993 to 2018 (FAOSTAT, 2018). High climate variability , topographic complexity (Stöklin, 1968; Zohary, 1973), the geographic isolation of the Iranian highlands (Noroozi et al., 2011) as well as soil diversity (Dewan & Famouri, 1963) established different phytogeographical units in Iran (Takhtajan, 1986; Zahran, 2010), which made it the center of a endemism of plant taxa in SW Asia (Klein, 1972; Hedge & Wendelbo, 1978), the center of endemism of Irano-Turanian (Takhtajan, 1986; Klein, 1981 &

1992; Leonard, 1991), as well as an important center of plant diversity around the world (Davis & Heywood, 1994; Barthlott, 1996 & 1999; Kier et al., 2005). These ecological conditions have led to the formation of valuable habitats for beekeeping in the country, which makes high diversity of honey production in Iran.

The Alborz together with the Zagros Mountains in Iran have created transition zones that have lead to high diversity and endemism in this region (Klein, 1972, Mehrabian et al. 2021). Influenced by elevation diversity in this area , various vegetation types have been formed (Fayaz et al. 2015, Ghelich Nia 2017), which have created suitable habitats for beekeeping and honey production (Nazarian et al. 1997, Razaghi et al. 2006). Today, the melissopalynology method has been used by a number of researchers in Iran, including: the melissopalynology of some honeys in Tehran Province (Nazarian et al. 1997), the melissopalynology of honey in Karaj, N. Iran (Sanei Shariat Panahi, 1974), melissopalynology of some honeys of Khorasan, N.E Iran (Ghoraishi & Memariani 2000), assessment the pollen grains of honey in Isfahan Province, C. Iran (Faghih et al. 2004), the melissopalynology of honey in some zones of Mazandaran Province (Razaghi et al. 2006), melissopalynology of honey in Arasbaran, N.W Iran (Ahmad Abad et al. 2017), and origin evaluation of some Iranian honeys (Khansaritoreh et al. 2021).

In addition to nutrients, honey contains bioactive substances that can have a positive effect on the human body (Rao et al.2016). The health properties of honey are due to the presence of phenolic compounds, including flavonoids, phenolic acids and their esters, as well as organic acids, free amino acids, vitamins, carotenoid derivatives, enzymes and biological elements (Kędzierska-Matysek et al. 2021). These compounds have antioxidant properties (Cianciosi et al.2018). Oxidation processes can be dealt with using antioxidant agents. Antioxidants can prevent many diseases or even reduce their symptoms (Cianciosi et al.2018). Honey can be a complementary source of antioxidants in a balanced diet (Alvarez-Suarez et al.2013). antioxidative of honey are attributed to the presence of phenolic compounds (Al-Mamary et al., 2002, Al Kiyumi et al.2021). Moisture level of bee honey is very important for its stability against fermentation and granulation. Low moisture protects honey from microbiological activity and therefore can be preserved for a longer period of time. (Cantarelli et al., 2008, Buba et al., 2013).

The aim of the study was to evaluate different honey samples originated from different ecological regions of Iran based on melissopalynological and chemical analysis. In addition, the

study tries to analyze the mentioned data in relation to geographical factors. Therefore, conducting a comparative study in different regions while comparing honey produced analyzes the relationship between pollen factors and some important chemical factors of honey with ecological factors.

2. Study area

2.1. Physical geography

Iran as a prominent area of Iranian Plateau in southwestern Asia situated in between 24°–40° N longitude and 44°–64° E latitude, As well a limited zone of the orogenic belt (Zagros, Alborz, and other mountain chains), covers the Asian block along with Arabian-African unity (Berberian and King, 1981). The prominent geomorphological zones of Iran including Alborz, Zagros, Kopet Dagh, as well several interior mountain chains. The Zagros mountain chain extended from northwest to southeast (Hokme, 2007). This geomorphological formation has created an natural wall between the Iranian plateau (northeast) and the Persian Gulf and Mesopotamian (southwest) basins. Also, the Alborz mountain chain as an active, arcuate fold-and-thrust belt (Stöcklin, 1974, 1977) as well a prominent sector of the Alpine-Himalayan geological formation. Shaped as an east-west, softly sinuous crescent in northern Iran, it forms a natural barrier between the Caspian Sea and the central Iranian plateau (Stöcklin, 1974). These natural massifs surrounding the central plateau and lead to diverse precipitation patterns in the mentioned area. In addition, Iran covers Mediterranean (western, northwestern Iran), temperate (northern Iran), and tropical (southern coast zones of the Persian Gulf and the Gulf of Oman) macro-bioclimates; however, its climatic zones consist of 35.5% hyper-arid, 29.2% arid, and 20.1% semi-arid regions. Additionally, precipitation patterns represent an average of about 250 mm annually, somewhat less than one third of the average rainfall in the world (860 mm) (Amiri and Eslamian, 2010). Most of our honey samples were collected from Alborz and Zagros sections, which have the highest honey production in Iran, which includes more than half of Iran's provinces, and most of the collected samples were close to forested and protected areas (Figure 1).

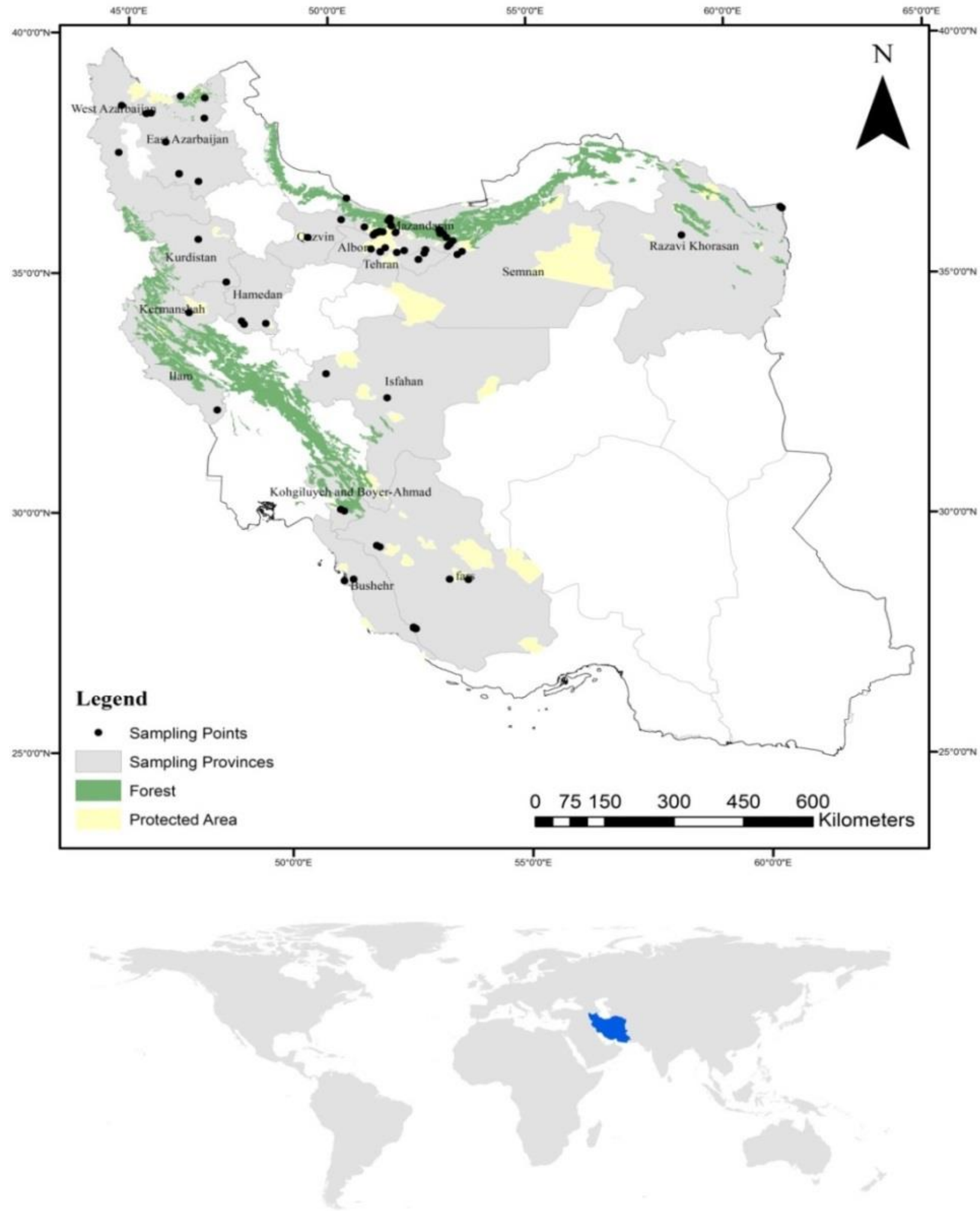

Figure. 1. Study area and sample collection points

2.2. Geobotany and Plant Ecology

The high forests of the Alborz, which are located only on the northern slopes (the Caspian Sea side), are dominated by deciduous Quercus macranthera, which usually reaches an altitude of 2400 meters in Alborz exceptionally and only in small protected pockets up to 2850 meters (Klein and Lacoste 1989; Naqinezhad and Esmailpoor 2017). In terms of plant geography, these forests belong to the Euro-Siberian region (Zohary 1973), and according to a bioclimatic classification, this region belongs to the Mediterranean pluviseasonal-oceanic biological climate using the Rivas-Martinez approach (Djamali et al. 2011). The southern slopes are mostly treeless, resulting in longer continental climates and stronger radiation during the long dry summer, although meteorological station data show that winter rainfall is still around ca. 550 mm> 2500 m a.s.l. In addition to plant species, Alborz high forests (Hyrkanian forests) host valuable animal species such as Iranian leopards(Farhadinia et al.2022). Zagros is the largest mountain range in Iran, which stretches from northeastern Iraq to southern Iran and its length is about 1300 km and width is about 250 km. This mountain range with an area of 394,000 square kilometers covers about a quarter of Iran's area (Noroozi et al. 2020). It has high peaks that reach sub-branches in different parts, but mostly in the central and southern Zagros. It has a continental climate and a Mediterranean rainy regime with cold and wet winters and hot and dry summers. Our database lists 3642 vascular plant species from this mountain range. The Zagros is located completely within the Iran-Turanian biogeographical region and the center of Iran-Anatolia biodiversity. More than 70% of the species are composed of Iranian-Turanian elements. The major vegetation types of the Zagros are oak woodlands, Amygdalus-Pistacia shrublands, montane steppe shrublands, wetlands, chasmophytic habitats, subalpine, alpine and subnival zones. (Noroozi et al. 2020). The vast expanse of the Zagros, its diverse climate and topography, and its high altitude range have led to the development of very different ecosystems and vegetation types. The main source of Zagros rainfall is the western systems carrying moisture from the Mediterranean and the Black Sea (Alijani and Harman 1985). The north-south extension of the Zagros

Range creates a strong rain shadow effect with much less annual rainfall on the eastern slopes than their western counterparts (Alijani et al. 2008). According to modern bioclimatic classifications (DJamali et al. 2011), Zagros belongs to different bioclimatic zones. Much of the western Zagros lies beneath the seasonal Mediterranean continental zone, characterized by high

annual rainfall in winter and early spring, which is in favor of deciduous oak forests. This mountain range shows the climate of the arid Mediterranean continents in its northeastern parts and the conditions of the drier Mediterranean continents in its southeastern parts (Djamali et al. 2011).

3. Materials and methods

3.1. Field studies

This study is based on direct collection of honey samples from hives in the area from May to October during 2020-2021. We selected sites for sampling that included different geographical and botanical areas (Al Naggar and El-Sofany 2018). Using a data collection form, the geographical coordinates of the hives were recorded by a Garmin Map 64s GPS device, the amount of height, the type of honey and the claims of beekeepers. Based on the diversity of ecological conditions, bee colonies were identified and samples were taken from the hives. Based on the diversity of ecological conditions, bee colonies were identified and samples were taken from the hives. From each colony, 1 sample of 300 g of unrefined and non-clay honey was prepared for pollen extraction and chemical analysis such as pH, antioxidant, total phenol, humidity. The samples were placed in sterile sealed containers and stored in a cool place until they reached the laboratory (Ponnuchamy et al. 2014).

3.2. Laboratory studies

Laboratory studies were performed in two parts:

Melissopalynology based on scanning electron microscopy (SEM)

These studies are based on the method (Louveaux et al. 1978). The melissopalynological method recommended by the International Commission on Bee Botany (ICBB) (Louveaux et al.1978; Von Der Ohe et al., 2004). First, 10 grams of honey in about 20 mL of distilled water was dissolved on a hot plate at a temperature of less than 40°C. After this step, honey wax samples were passed through a cloth strainer to remove large particles of honey wax, then the liquid was poured into centrifugal tubes with conical ends and a capacity of 30 mL. After several laboratory centrifuges (2500-3000 rpm, Model TDL-4 centrifuge) at room temperature (21 to 25°C), pollen grains were extracted from honey. In the first round, the samples were centrifuged for 10 minutes (about 2500 rpm), and the supernatant was carefully discarded to prevent pollen loss (Ponnuchamy et al. 2014). The remaining precipitate was mixed again with about 10 mL of

distilled water, but this time the samples were centrifuged for 5 minutes. In our study, we repeated this process 4 to 7 times, depending on the amount of sugar in the samples, so that the sugar in the honey was completely removed. But after the last centrifuge, we poured the final remaining liquid into the watch glass and placed it on the hot plate so that the water evaporated completely and the pollen grains remained at the bottom of the container. During the process, great care must be taken to prevent any contamination from external pollen, which is from previous honey products (use of disposable appliances is recommended as much as possible) or pollen grains in the air (close windows and limit exposure).
We placed the prepared pollen grains on special bases and after covering the samples with gold metal, we placed them inside the housing of HITACHI SU3500 scanning electron microscope (SEM) machine made in Japan, and depending on the size of the sample, using this microscope, it is possible to shoot surfaces with a magnification of 10 to 500,000 times and with a resolution of less than 1 to 20 nanometers. By analyzing the information obtained from electron microscopy, we identified and counted the pollen grains and then obtained the plant origin of honeys.

- *Identification and counting*

Using scanning electron microscopy images, pollen grains obtained from honey sediment were counted, counting at least 300 pollen grains is necessary to estimate the relative abundance of pollen types and 500 to 1000 pollen grains are necessary to determine the relative abundance (Behm et al. 1996). We also used pollen atlases and websites to identify pollen, identification of pollen grains was done according to the morphology of pollen grains. Pollen species should only be referred to as botanical species or species if they are reliably identified at the genus or species level, which is rare (Von Der Ohe et al. 2004).

3.2.2. Laboratory studies and chemical analysis of honey

All the reagents for biochemical studies were obtained from Merck Chemical Company. DPPH was obtained from Sigma chemicals.
In this part of the study, some chemical analysis of honey such as antioxidant , total phenol, pH and humidity content were investigated. For all chemical analysis honey was diluted 1/1 (w/v) by distilled water.

- *Analysis of antioxidant by DPPH free radical scavenging activity*

At this stage, the DPPH method was used to analyze all 94 samples of honey (Chua et al.2013), which dissolved a concentration of 0.1 mM of DPPH in methanol and a purple solution was obtained , 100 µl of DPPH was mixed with 10 µl of diluted honey and incubated 30 minutes in dark place. The absorption was recorded at 520 nm by the JENUS V-1100 spectrophotometer. Ascorbic acid (20 mg/ ml) was used as positive control. The antioxidant activity was calculated from the following formula.

DPPH scavenging activity (%) = $A_{control} - A_{sample} / A_{control} \times 100$

- *Analysis of total phenol*

In this experiment, 1% iron chloride solution $FeCl_3$ 1% was used to measure the amount of total phenol in honey(Athipornchai and Klangmanee, 2021), Phenol with a concentration of 0.09% (mg / kg) was used as a standard sample. 0.1 ml of $FeCl_3$ was mixed with 0.1 ml of diluted honey and the absorbance was recorded at 590 nm. Phenol solution (0.09 %) was used as a standard.

- *Measuring pH and the moisture of honey*

For pH determination, 1 g of honey was mixed with 1 ml of distilled water and the pH of each sample was measured by a pH Meter model JENWAY 3505 and the moisture content of pure honey was determined with a refractometer.

4. Results

4.1. Pollen analysis

In this study, after analyzing pollen grains of 94 honey samples, 42 plant families were identified, that in total, Asteraceae families with 38%, Fabaceae with 13.5%, Rosaceae with 6%, Apocynaceae & Apiaceae with 5% each showed the highest presence (Figure 2) and 55 plant genus (Table 1) were identified.

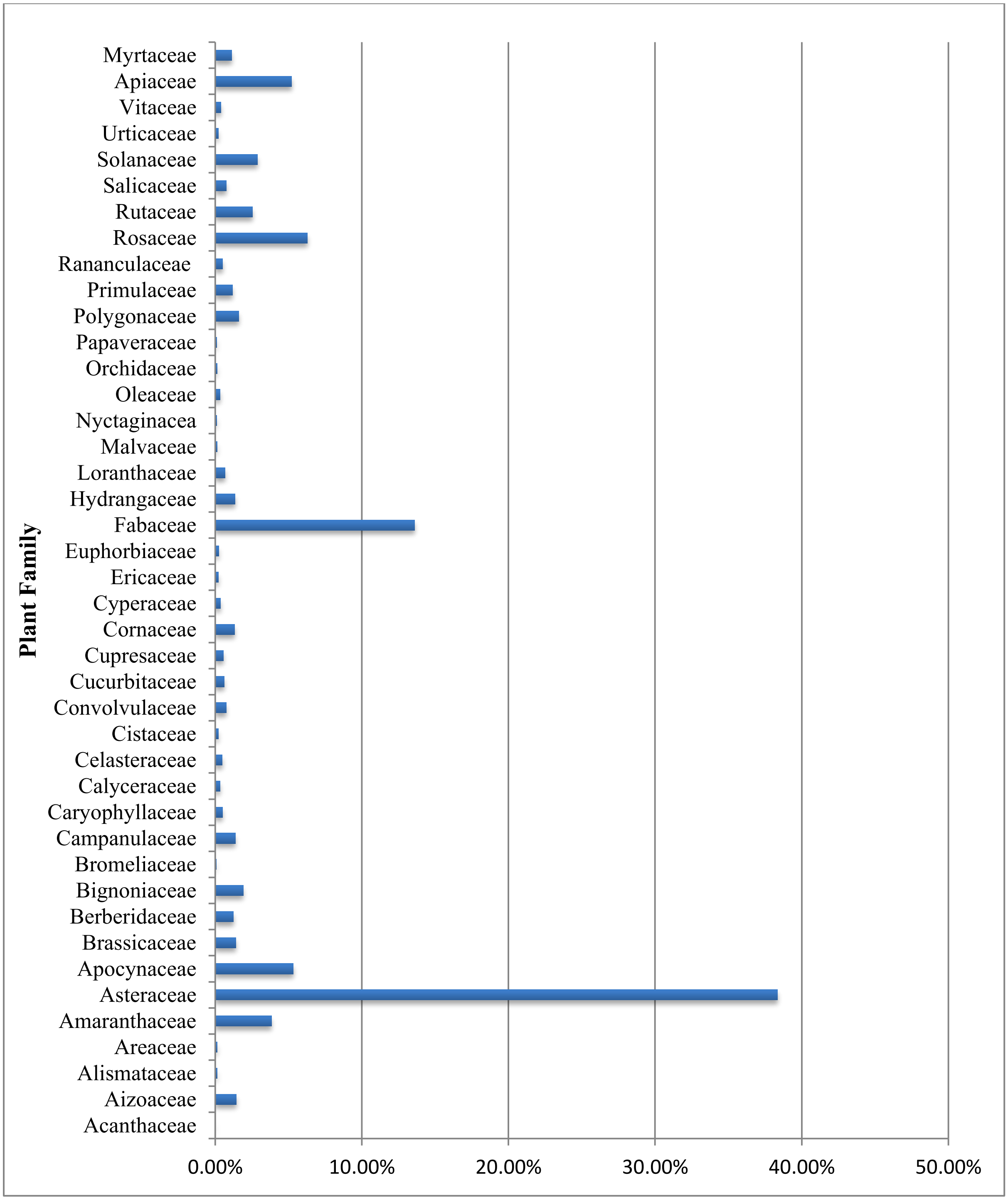

Figure. 2. Percentage of presence of each plant family that was identified through analysis of pollen of 94 honey samples

Table 1. Plant genus identified from pollen analysis of 94 honey samples

Family	Genus
**Amaranthaceae**	*Amaranthus sp.*
	Atriplex type
**Apocynaceae**	*Catharanthus type*
**Apiaceae**	*Apiaceae type*
	Apiaceae type
	Torilis
**Asteraceae**	*Acanthaceae type*
	Achillea sp.
	Artemisia sp.
	Carduus type
	Carthamus type
	Centaurea type
	Cirsium arvense
	Crepis
	Doronicum sp.
	Helichrysum sp.
	Hieracium sp.
	Inula sp.
	Senecio sp.
**Bignoniaceae**	*Bigononiaceae type 1*
	Bignoniaceae type 2
**Brassicaceae**	*Brassicaceae type*
**Caryophyllaceae**	*Caryphyllaceae type*
**Cistaceae**	*Cistus sp.*
**Convolvulaceae**	*Convolvulus arvensis*
**Cornaceae**	*Cornus sanguinea*
**Cucurbitaceae**	*Cucurbit asp.*
**Cyperaceae**	*Carex sp.*

**Fabaceae**	*Astragalus type*
	Robinia pseudacacia
	Hedysarum sp.
	Onobrychis cornuta
	Vicia sp.
**Hydrangeaceae**	*Philadelphus*
**Myrtaceae**	*Eucalyptus calmadonensis*
**Oleaceae**	*Olea europaea*
**Polygonaceae**	*Fagopyrum*
	Polygonum sp.
	Rumex sp.
**Ranunculaceae**	*Adonis sp.*
	Clematis sp.
	Ranunculus sp.
**Rosaceae**	*Alchemilla sp.*
	Cotoneaster sp.
	Sorbaria
	Prunus domestica
	Rosa cania
	Sorbaria sp.
	Spiraea sp.
**Rutaceae**	*Rutaceae type*
	Poncirus
**Salicaceae**	*Populus nigra*
**Solanaceae**	*Solanum spp.*
**Urticaceae**	*Parietaria judaica*
	Urtica dioica

Pollen grains extracted from honey samples were identified based on pollen references. Quantitative evaluation of pollen grains in each of the honey samples was performed according to the Louveaux standard. The collected samples were in the altitude ranges of 12 to 3062 meters above sea level. The samples were collected in 7 altitude intervals, which in terms of vegetation type changes and the location of beekeepers in accordance with the season, more than 73% of the samples were in the altitude range of 1000-3000 meters above sea level (Figure 3).

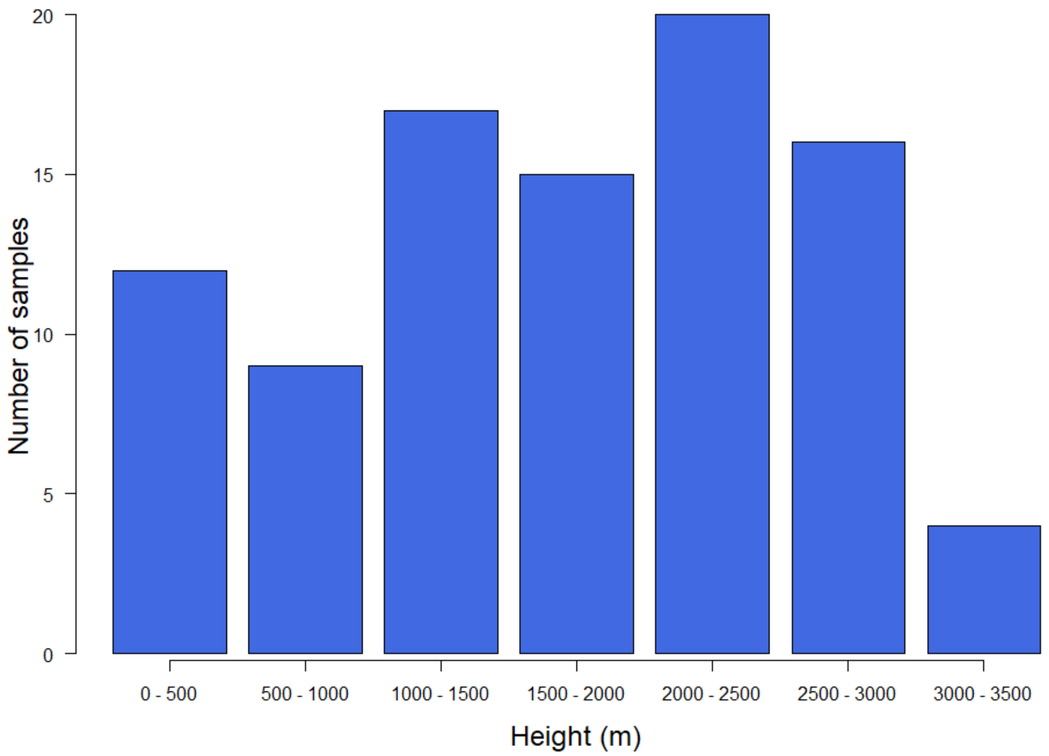

Figure. 3. Number of sampling performed at different altitude intervals

4.2. Physicochemical analysis of honeys

According to the results obtained from chemical analysis of honey samples, the amount of antioxidants in the samples was the lowest of 19% and the highest of 98%. According to (Figure 4), more than 36% of the total samples had antioxidants between 40% and 60%.

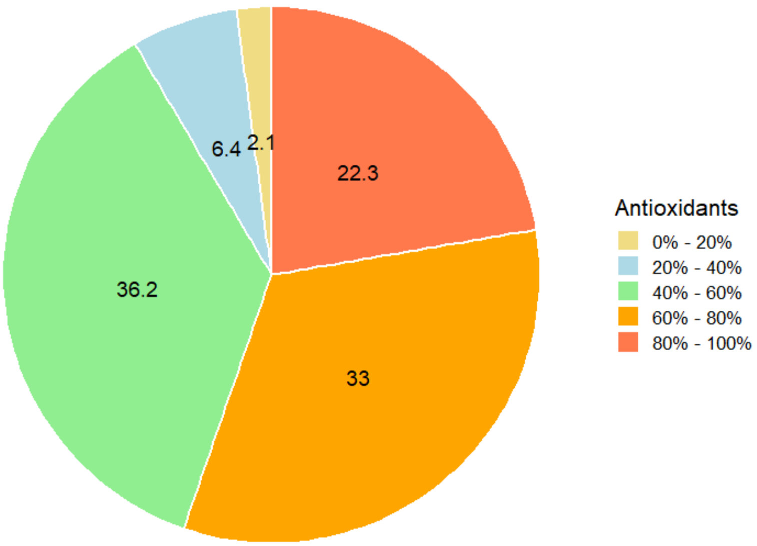

Figure. 4. Antioxidant abundance ratio of honey samples

The total phenol content of honey samples was between 0.08 to 0.59 micrograms. Differences in phenolic content in honey samples may be due to different geographical origins or other factors such as collection season and storage conditions. Also in this section, the pH of each honey sample was examined.

The lowest pH was 1.9 and the highest pH was 5.2. In the information obtained from measuring the moisture content of each honey sample, it was determined that the maximum moisture content was 18.8% and the minimum was 13%. We compared the humidity of honey samples with the humidity of the areas from which the samples were collected, which shows that the humidity of the environment affected the humidity of honey samples (Figure5).

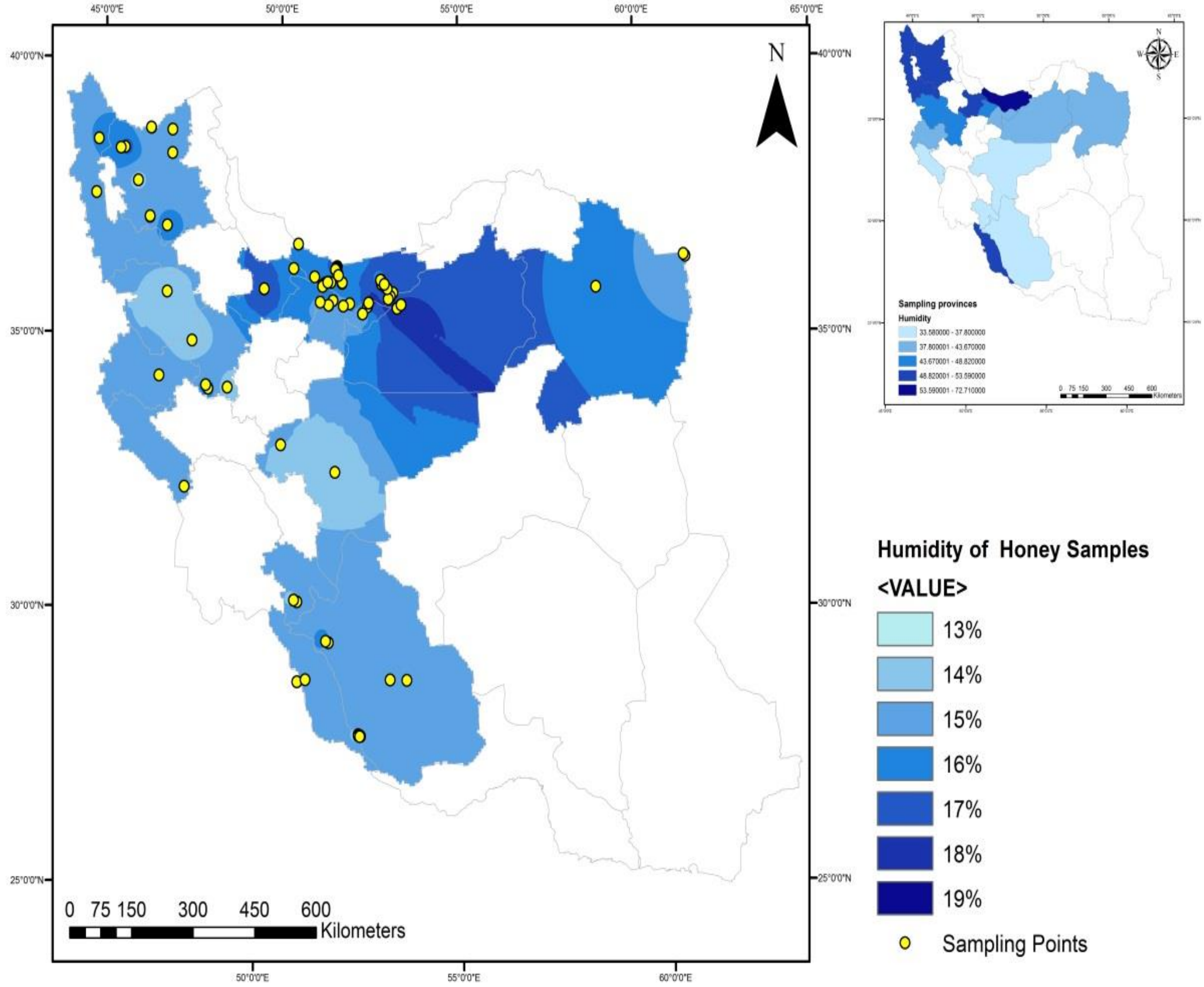

Figure. 5. Comparison of moisture content of honey samples with moisture content of the collection site

All honey samples were grouped with chemical factors obtained from them, which our plot (Figure 6) shows that the factors, pH and total phenol were positively correlated for grouping and factors, humidity and antioxidants for grouping were negatively correlated they had.

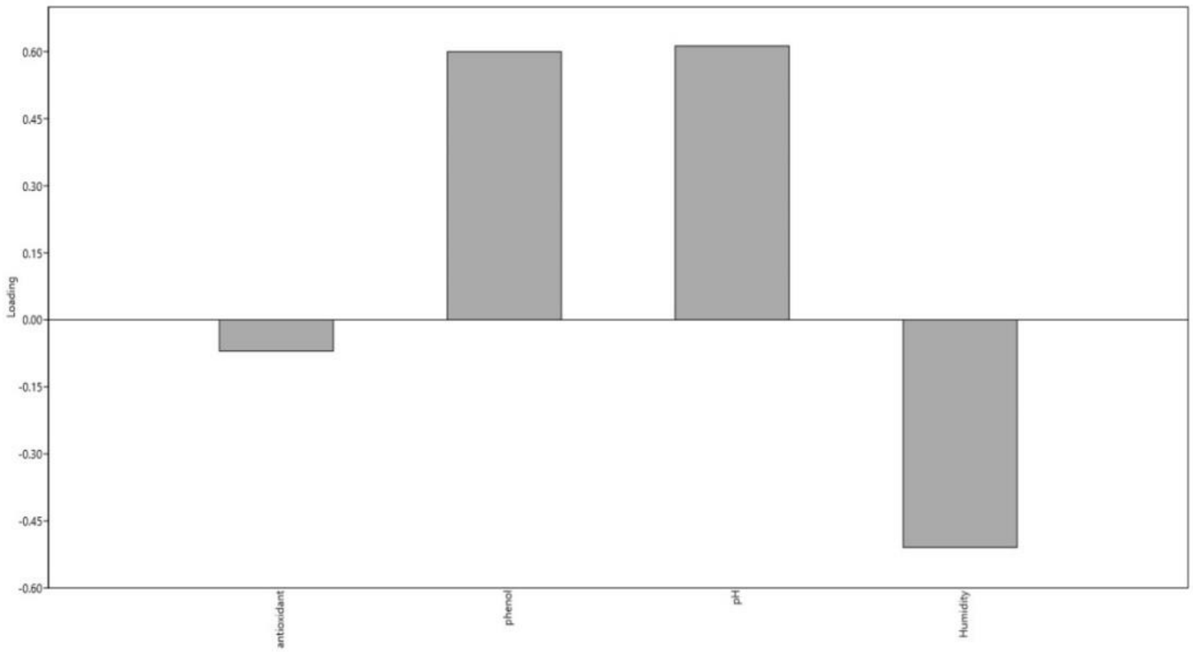

Figure. 6. Plot related to the grouping of total honey samples using chemical factors

Honey samples collected in this study were from different geographical areas, Based on the number of beekeepers present in each region, the number of different honey samples was collected from some areas, 2 samples of honey and some areas, more than 2 samples of honey were collected. The plot obtained (Figure 7) shows that in order to group the samples with their chemical composition, even the samples collected from a geographical area do not have the same chemical composition.

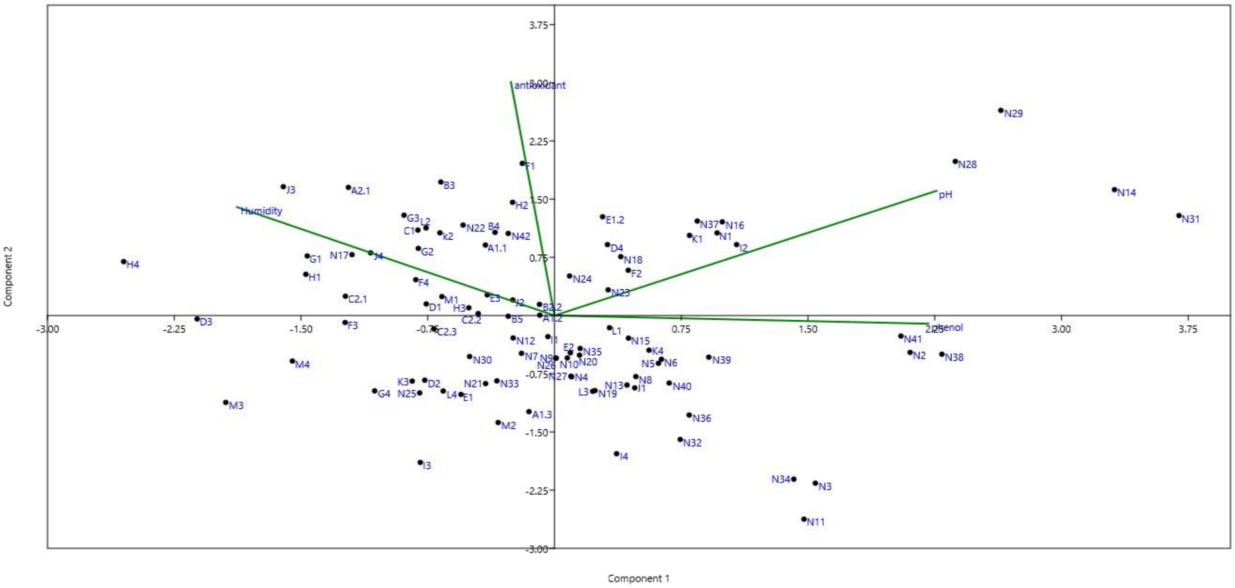

Figure. 7. Grouping the samples with their chemical compounds to show the difference in the amount of chemical compounds between the samples according to their geographical areas

Based on the plant families obtained from each honey sample, we performed a cluster analysis (Figure 8) that shows how close or distant the honey samples were in terms of the composition of plant taxa .

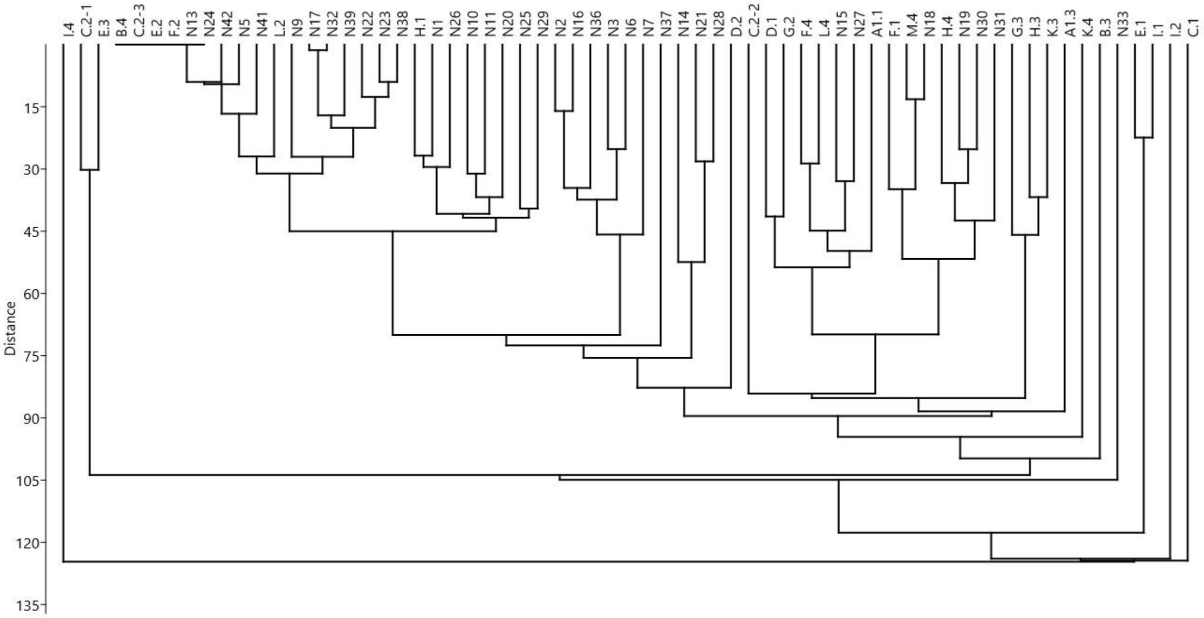

Figure. 8. Cluster analysis to find out how close or distant honey samples were in terms of plant taxon composition

5. Discussion

In this study, 94 samples of honey collected belong to the main honey producing regions in Iran. In the results of this study, 42 plant families and 57 genera of these families were identified. Due to the difficulty of identifying pollen grains extracted from honey at the genus or species level (Von Der Ohe et al.2004), we were able to identify up to the genus level in our studies.

The highest density of pollen grains in honey samples belong to the Asteraceae, Fabaceae, Rosaceae, Apiaceae, Apocynaceae (Figure 9 a,b,c,d). The mentioned frequency showing high similararity to the following studies: Karimi et al. (2016) in Fars province and Razzaqi et al. (2006) in Mazandaran province. However the Asteraceae, Fabaceae, Rosaceae, Apiaceae, Brassicaceae, Papilionaceae, Lamiaceae, showed the highest presence respectiveley, In Rašić et al.(2018).

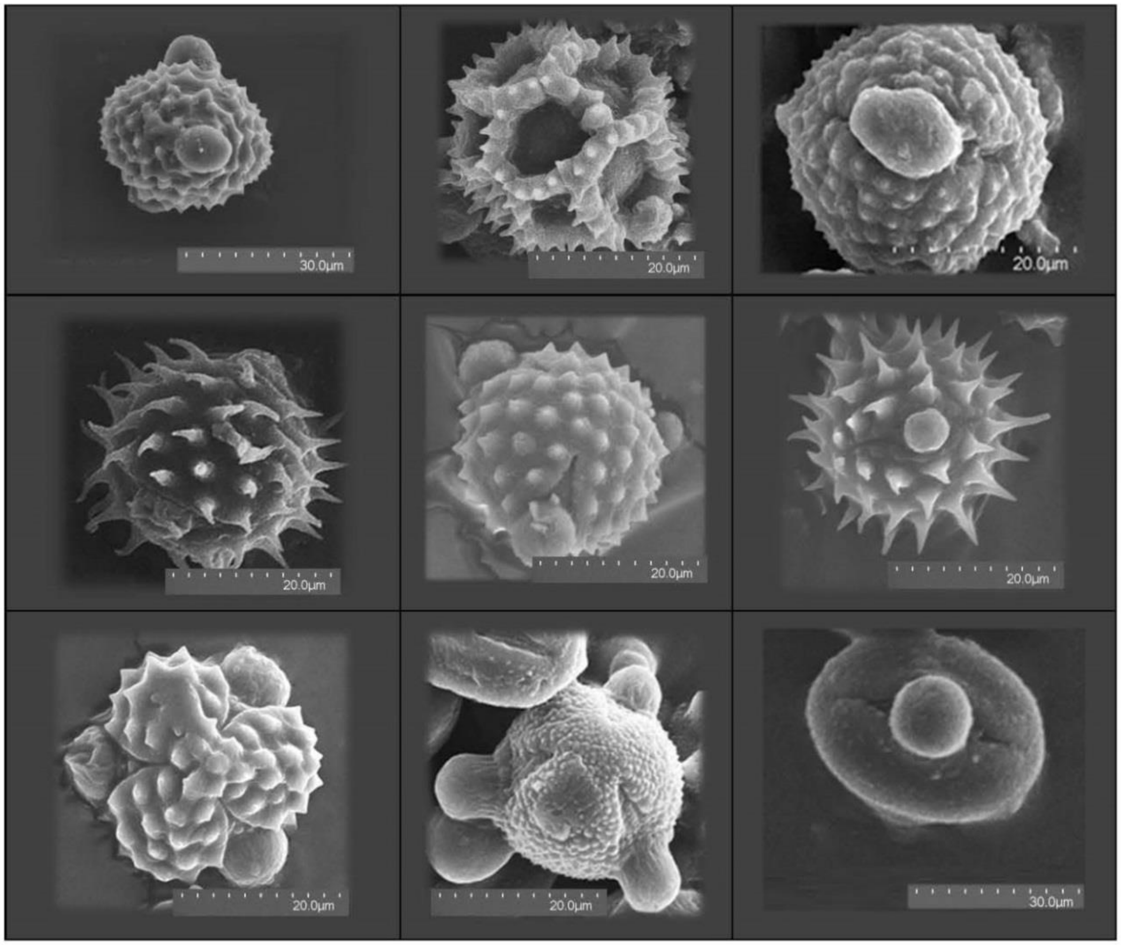

Figure. 9a. Some pollen images of the Asteraceae family

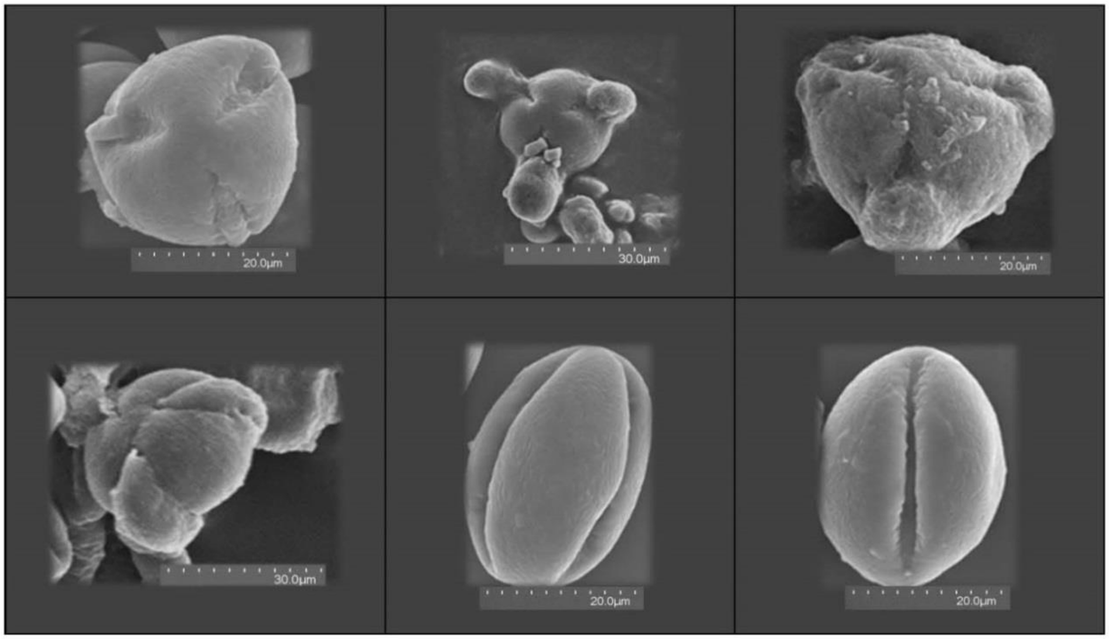

Figure. 9b. Some pollen images of the Rosaceae family

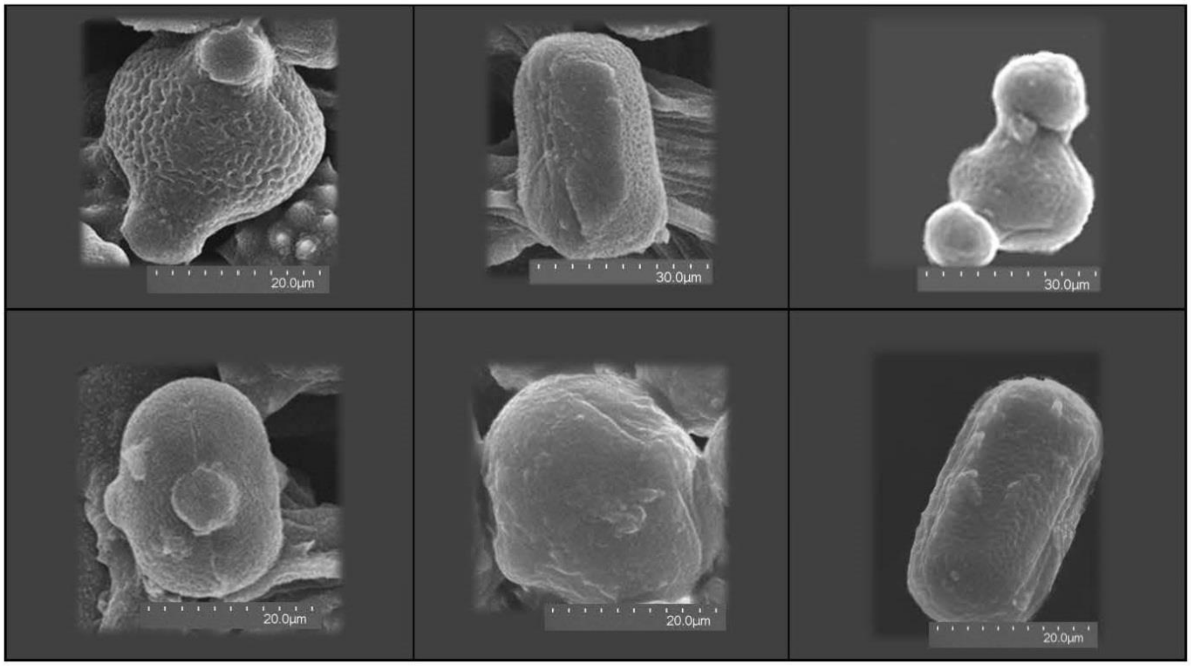

Figure. 9c. Some pollen images of the Fabaceae family

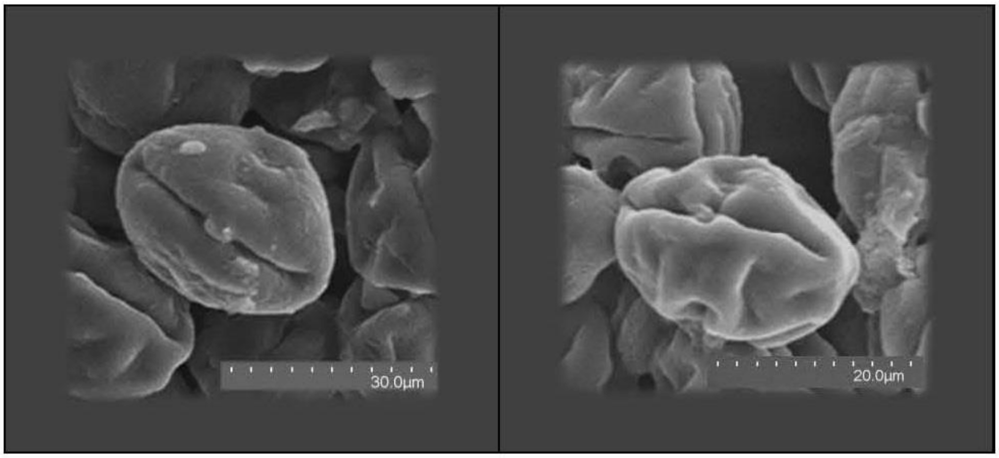

Figure. 9d. some pollen images of the Apocynaceae family

Moreover The Asteraceae, Fabaceae and Rosaceae were the most present, in the Stonia (Puusepp and Koff 2014) Croatia Our results showing all the studied samples are classified as Polyfloral honey. The results of our studies show that according to palinology criteria, about 30% of the honey samples studied are counterfeit, but the rest of the honey is of good quality.

The chemical, physical, antioxidant, and healthy composition of honey varies depending on the flower source, soil type, geological roots, and climatic conditions. CACS (2001). Both ancient and modern civilizations use honey as a medicine for various diseases (Berhanu et al.2022). It is also a delicious food that is mostly made of different sugars and compounds such as proteins, organic acids, amino acids, nutrients, minerals, carotenoids and aromatic substances. It is rich in flavonoids and phenolic acids, which show a wide range of biological effects and act as a natural antioxidant (karabagias, et al. 2014; Manyi-Loh 2011). The results of our chemical analysis showed that the chemical composition of honey samples collected from different ecological areas are different from each other (Kuldasheva et al.2022). Chemical experiments also showed that about 3/5 of the samples showed more than 60% of the effect of antioxidant activity. We compared the results of antioxidants with increasing height, which seems to have a positive response to increasing height and up to 3000 meters, the trend line shows an upward trend, but after 3000 meters, antioxidants follow a completely opposite trend. The highest effect of antioxidant activity was recorded in the altitude range of 2500-3000 meters, which showed an average of 88.71% (Figure 10).

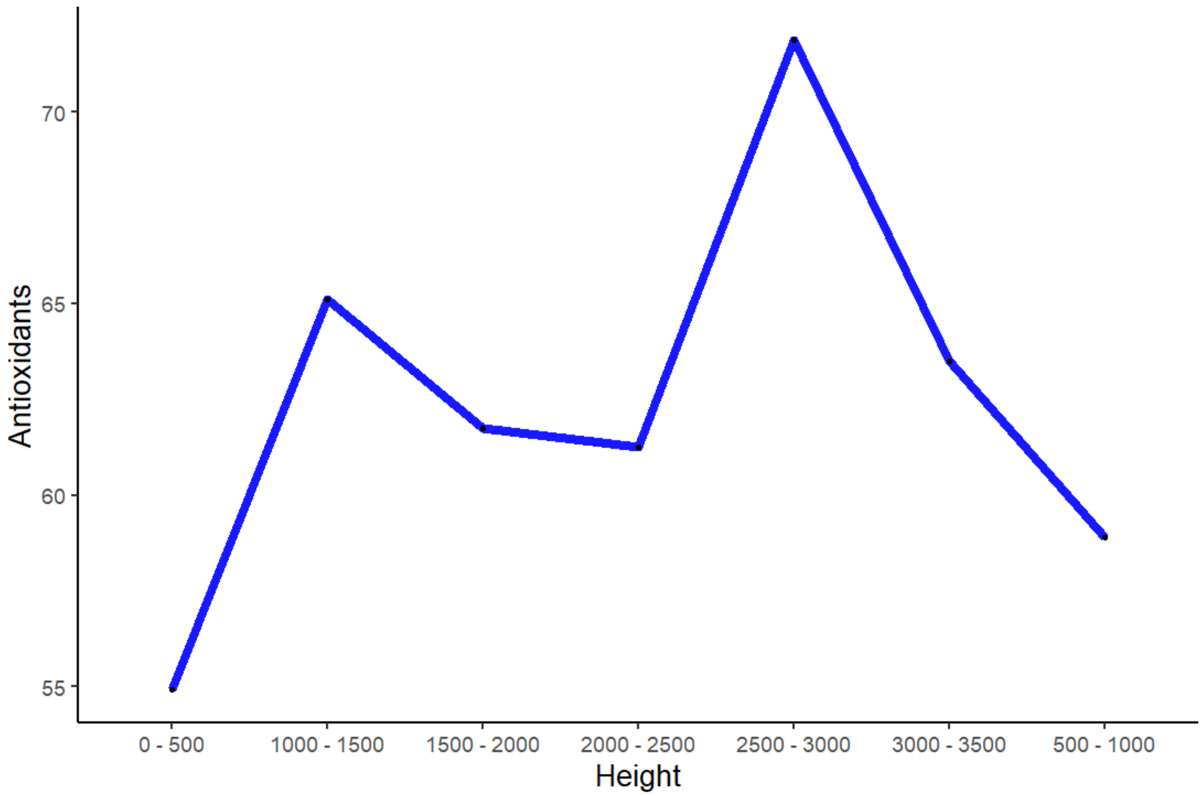

Figure. 10. Comparison of antioxidants with altitude

Variation in the antioxidant potential of honey depends on topographic and botanical differences (Martos et al. 2000), (Yao et al. 2003). The antioxidant activity of honey is mainly due to phenolic and flavonoid compounds. Thus, a significant variation in antioxidant activity is found among different types of honey worldwide (Beretta et al., 2005; Aljadi and Kamaruddin, 2004; Nayik and Nanda, 2016). An important group of compounds that affect the appearance and functional properties of honey are phenolic acids, which have nutritional value and therapeutic properties (Alvarez-Suarez et al., 2010). The type and concentration of phenolic compounds in different honeys are different from each other (Saxena et al. 2010).

The amount of phenolic compounds is influenced by various factors such as plant type, geographical origin and climatic conditions of the honey production site (Escuredo et al. 2012). Determining the amount of phenolic compounds in honey is a suitable factor to evaluate the quality and therapeutic potential of honey (Ouchemoukh et al. 2007). In the results of our analyzes, the samples showed phenolic compounds of 0.08-0.51 μg. Honey is recognized as a

treatment option for the treatment of acute and chronic wounds, and a wide range of in vitro and in vivo research studies have been performed in this field (Subrahmanyam, 1998; Cooper et al. 2002; Johnson et al. 2005). Low pH of honey (0.4 - 5.3) helps wound healing (Efem, 1988; Molan, 1999), which is done by reducing the high alkaline environment in non healing wounds to a more acidic healing environment. The global standard for pH of honey is 3.9 (3.2 – 4.5). Based on the results of our studies, honey samples showed a pH 1.90 - 5.21 and 48% of the total samples are in the pH range of 2.5 - 3.5 (Figure 11).

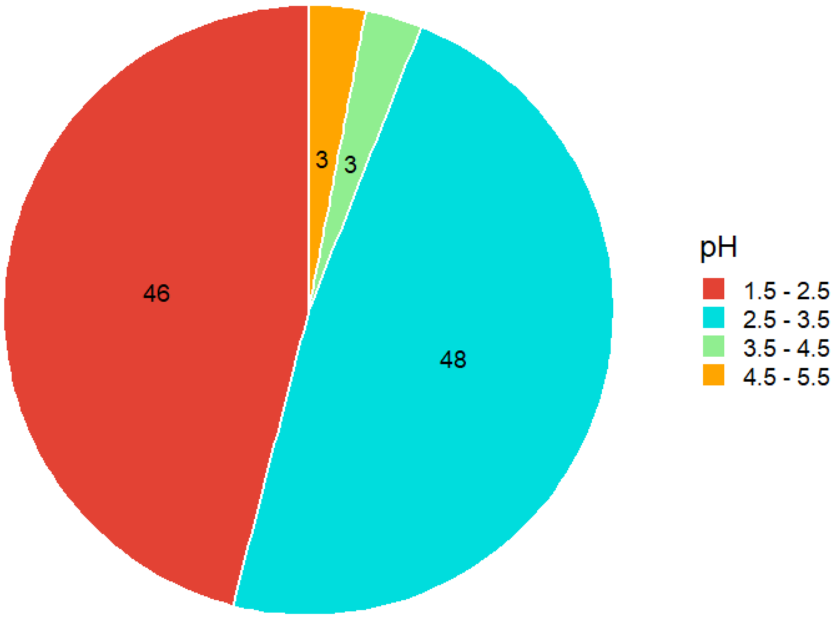

Figure. 11. pH abundance ratio of the samples

And pH values between 3.4 and 6.1 indicate the freshness of honey samples (Khalil et al. 2012; Selvaraju et al. 2019). Moisture content is considered as an important criterion for measuring the quality of honey. Honey with less moisture has a longer shelf life (Fredes and Montenegro, 2006). The moisture content of honey depends on various factors such as harvest time, climatic factors of the geographical area and storage temperature (Nanda et al., 2003; Finola et al., 2007). In this study, all honey samples showed a low moisture content (13% -18.80%), which corresponded to the values (<20 <) set by the Codex standard for honey (Almentarius Codex, 1987). The parameter of quality and uniqueness of honey has been studied by various researchers, this quality and credibility of honey is important for both buyers and producers.

One of the important concerns of honey quality is to guarantee the authenticity of honey according to legal requirements. This authenticity is achieved by recognizing the plant origin, geography and chemical analysis (da Silva et al.2016), (Machado et al.2018) Nutritional and healing properties of honey from the very beginning make it an important component of traditional medicine (Bogdanov 1997; Subrahmanyam 1998). The natural antioxidants in honey are valuable against heart disease, cancer, inflammation, coronary heart disease, aging, tumors, gastrointestinal tract and wound healing (de Rodríguez et al. 2004), (Kečkeš et al.2013) . In relation to health, honey, bee pollen, propolis, royal jelly, beeswax and bee venom have all been used in traditional and modern medicine (Easton-Calabria et al. 2019; Kocot et al. 2018). Researchers have identified the bioactive properties of honey, propolis and royal jelly, indicating compounds with antimicrobial, anti-inflammatory, antioxidant, anti-tumor and anti-cancer activities (Pasupuleti et al. 2017; Kocot et al. 2018; Easton Calabria et al. 2019). Honey is used to treat stomach ulcers and injuries, increase oral health, fight stomach disorders, and liver and pancreatic diseases, as well as to promote cardiovascular health (Pasupuleti et al. 2017; Easton-Calabria et al. 2019) .

6. Conclusion

Melissopalynology is a useful and complementary tool along with chemical assays to measure the authenticity, fraud and differentiation between different types of honey.In addition to being a useful tool in the food industry and therapeutic debates, melissopalynology is even more important in the conservation debate. Conducting more extensive studies in the field of melissopalynology in the future to identify the plant species used by bees is of mutual importance in the conservation debate, plant species conservation protects bees and bee conservation leads to plant species conservation. In the field of conservation, more and more ongoing research is needed to better understand the behavior and role of bees and plants. On the other hand, seasonal changes (including changes in temperature, natural light, precipitation) and changes in vegetation type can not only have a significant impact on the behavior of bees but also the behavior of other animals (Salmanpour et al.2021).

7. Recommendation

It is recommended that future studies on pollen of honey produced by farmed bees be compared to honey produced by wild bees to help better understand the role of melissopalynology in the protection of bees and plant species and the protection of ecosystems. Bee populations are threatened by human activities. Climate change, invasive species, monocultures that have less variety in flower resources, and the use of pesticides have a negative effect on bees. On the other hand, seasonal changes (including changes in temperature, natural light, precipitation) and changes in vegetation type can not only have a significant impact on the behavior of bees but also the behavior of other animals (Salmanpour et al. 2021). An understanding of ecosystem processes and the importance of the presence of bees and plants to preserve the planet raises the concern that biodiversity loss is directly affecting ecosystem performance.

Funding

This research received no external funding.

Acknowledgments

Many thanks to Mr. Saeed Javadi

Many thanks to Mr. Mehdi Kia Hairati

Many thanks to Mr. Mohsen Yasari

Many thanks to Mr. Erfan Rajabi

Many thanks to Mr. Mohammad Reza Kashfi

CRediT authorship contribution statement

Zahra Shakoori: Conceptualization, Principal Investigation, Formal analysis, Writing - Original Draft, Sample collection, Software .

Ahmadreza Mehrabian: Conceptualization, Formal analysis, Writing - Reviewing and editing.

Dariush Minai: Conceptualization, Formal analysis, Writing - Reviewing and editing.

Farid Salmanpour: Conceptualization, Formal analysis, Writing - Reviewing and editing, Sample collection, Software.

Farzaneh Khajoei Nasab: Conceptualization, editing, Software.

Conflicts of Interest

The authors confirm that they have no conflicts of interest with respect to the work described in this manuscript.

DATA AVAILABILITY STATEMENT

https://figshare.com/s/d21d14ac03da862023bd

References

1. Alijani, B., O'Brien, J. and Yarnal, B., 2008. Spatial analysis of precipitation intensity and concentration in Iran. *Theoretical and Applied Climatology*, *94*(1), pp.107-124. https://doi.org/10.1007/s00704-007-0344-y
2. Alijani, B. and Harman, J.R., 1985. Synoptic climatology of precipitation in Iran. *Annals of the Association of American Geographers*, *75*(3), pp.404-416. https://doi.org/10.1111/j.1467-8306.1985.tb00075.x
3. Aljadi, A.M. and Kamaruddin, M.Y., 2004. Evaluation of the phenolic contents and antioxidant capacities of two Malaysian floral honeys. *Food chemistry*, *85*(4), pp.513-518. https://doi.org/10.1016/S0308-8146(02)00596-4
4. Al Kiyumi, E.H., Al Rashdi, B.S., Al Alawi, A.R., Al Balushi, A.A., Al Hooti, S.N., Al Hosni, S.I., Dhanalekshmi, U.M. and Khan, S.A., 2021. Quantification of bioactive components and evaluation of antioxidative potential of different floral origin honey from arid regions of Oman. *Biocatalysis and Agricultural Biotechnology*, *33*, p.102007.
5. Al-Mamary, M., Al-Meeri, A. and Al-Habori, M., 2002. Antioxidant activities and total phenolics of different types of honey. *Nutrition research*, *22*(9), pp.1041-1047.
6. Al Naggar, Y. and El-Sofany, A., 2018. Characterization of Apis mellifera Honey of Different Botanical and Geographical Origins in Egypt. http://dx.doi.org/10.5455/egysebz.20180523104927
7. Alnaqdy, A., Al-Jabri, A., Al Mahrooqi, Z., Nzeako, B. and Nsanze, H., 2005. Inhibition effect of honey on the adherence of Salmonella to intestinal epithelial cells in vitro. *International journal of food microbiology*, *103*(3), pp.347-351. https://doi.org/10.1016/j.ijfoodmicro.2004.11.042
8. Alvarez-Suarez, J.M. ed., 2017. *Bee products-chemical and biological properties*. Springer. https://doi.org/10.1007/978-3-319-59689-1

9. Alvarez-Suarez, M., J., Giampieri, F., & Battino, M.(2013). Honey as a source of dietary antioxidants: structures, bioavailability and evidence of protective effects against human chronic diseases. Current medicinal chemistry, 20(5), pp.621-638. https://doi.org/10.2174/0929867113804999358

10. Alvarez-Suarez, J.M., Tulipani, S., Romandini, S., Bertoli, E. and Battino, M., 2010. Contribution of honey in nutrition and human health: a review. *Mediterranean Journal of Nutrition and Metabolism*, *3*(1), pp.15-23. https://doi.org/10.1007/s12349-009-0051-6

11. Amiri, M.J. and Eslamian, S.S., 2010. Investigation of climate change in Iran. *Journal of Environmental Science and Technology*, *3*(4), pp.208-216.

12. Andrade, P.B., Amaral, M.T., Isabel, P., Carvalho, J.C., Seabra, R.M. and da Cunha, A.P., 1999. Physicochemical attributes and pollen spectrum of Portuguese heather honeys. *Food Chemistry*, *66*(4), pp.503-510. https://doi.org/10.1016/S0308-8146(99)00100-4

13. Barthlott, W., Biedinger, N., Braun, G., Feig, F., Kier, G. and Mutke, J., 1999. Terminological and methodological aspects of the mapping and analysis of global biodiversity. *Acta botanica fennica*, *162*(0), pp.103-110.

14. Barthlott, W., Neinhuis, C., Jetter, R., Bourauel, T. and Riederer, M., 1996. Waterlily, poppy, or sycamore: on the systematic position of Nelumbo. *Flora*, *191*(2), pp.169-174. https://doi.org/10.1016/S0367-2530(17)30709-0

15. Basualdo, C., Sgroy, V., Finola, M.S. and Marioli, J.M., 2007. Comparison of the antibacterial activity of honey from different provenance against bacteria usually isolated from skin wounds. *Veterinary microbiology*, *124*(3-4), pp.375-381. https://doi.org/10.1016/j.vetmic.2007.04.039

16. Behm, F., von der Ohe, K. and Henrich, W., 1996. Zuverlässigkeit der Pollen analyse von Honig. Bestimmung Der Pollenhäufigkeit, Dtsch. Lebensm, 92: 183-187.

17. Belay, A., Haki, G.D., Birringer, M., Borck, H., Addi, A., Baye, K. and Melaku, S., 2017. Rheology and botanical origin of Ethiopian monofloral honey. *LWT*, *75*, pp.393-401. https://doi.org/10.1016/j.lwt.2016.09.021

18. Berberian, M. (1983). The southern Caspian: a compressional depression floored by a trapped, modified oceanic crust. *Canadian Journal of Earth Sciences*, *20*(2), 163-183. https://doi.org/10.1139/e83-015

19. Berberian, M. and King, G.C.P., 1981. Towards a paleogeography and tectonic evolution of Iran. *Canadian journal of earth sciences*, *18*(2), pp.210-265. https://doi.org/10.1139/e81-019
20. Beretta, G., Granata, P., Ferrero, M., Orioli, M. and Facino, R.M., 2005. Standardization of antioxidant properties of honey by a combination of spectrophotometric/fluorimetric assays and chemometrics. *Analytica Chimica Acta*, *533*(2), pp.185-191. https://doi.org/10.1016/j.aca.2004.11.010
21. Berhanu, S., Tadesse, D.M. and Jorge, A., 2022. Physico-chemical properties of Ethiopian Apis mellifera Honey: Review. *J Agric Sc Food Technol*, *8*(1), pp.038-044. https://dx.doi.org/10.17352/2455-815X.000143
22. Bogdanov, S., Jurendic, T., Sieber, R. and Gallmann, P., 2008. Honey for nutrition and health: a review. *Journal of the American college of Nutrition*, *27*(6), pp.677-689. https://doi.org/10.1080/07315724.2008.10719745
23. Bogdanov, S. and Martin, P., 2002. Honey authenticity. *Mitteilungen aus Lebensmitteluntersuchung und Hygiene*, *93*(3), pp.232-254.
24. Bogdanov, S., 1997. Nature and origin of the antibacterial substances in honey. *LWT-Food Science and Technology*, *30*(7), pp.748-753. https://doi.org/10.1006/fstl.1997.0259
25. Bryant Jr, V.M. and Jones, G.D., 2001. The r-values of honey: Pollen coefficients. *Palynology*, *25*(1), pp.11-28. https://doi.org/10.1080/01916122.2001.9989554
26. Buba, F., Gidado, A. and Shugaba, A., 2013. Analysis of biochemical composition of honey samples from North-East Nigeria. *Biochem Anal Biochem*, *2*(3), p.139.
27. Cantarelli, M.A., Pellerano, R.G., Marchevsky, E.J. and Camiña, J.M., 2008. Quality of honey from Argentina: Study of chemical composittion and trace elements. *The Journal of Argentine Chemical Society*, *96*(1-2), pp.33-41.
28. Christy, E.M.L. and Anna, M.C., 2011. An overview of honey: Therapeutic properties and contribution in nutrition and human health. *African Journal of Microbiology Research*, *5*(8), pp.844-852. https://doi.org/10.5897/AJMR10.008

29. Chua, L. S., Rahaman, N. L., Adnan, N. A., & Eddie Tan, T. T. (2013). Antioxidant Activity of Three Honey Samples in relation with Their Biochemical Components. *Journal of analytical methods in chemistry*, *2013*, 313798. https://doi.org/10.1155/2013/313798
30. Cianciosi, D., Forbes-Hernández, T.Y., Afrin, S., Gasparrini, M., Reboredo-Rodriguez, P., Manna, P.P., Zhang, J., Bravo Lamas, L., Martínez Flórez, S., Agudo Toyos, P. and Quiles, J.L., 2018. Phenolic compounds in honey and their associated health benefits: A review. *Molecules*, *23*(9), p.2322. https://doi.org/10.3390/molecules23092322
31. Cooper, R.A., Molan, P.C. and Harding, K.G., 2002. The sensitivity to honey of Gram-positive cocci of clinical significance isolated from wounds. *Journal of Applied microbiology*, *93*(5), pp.857-863. https://doi.org/10.1046/j.1365-2672.2002.01761.x
32. Corvucci, F., Nobili, L., Melucci, D. and Grillenzoni, F.V., 2015. The discrimination of honey origin using melissopalynology and Raman spectroscopy techniques coupled with multivariate analysis. *Food chemistry*, *169*, pp.297-304. https://doi.org/10.1016/j.foodchem.2014.07.122
33. da Silva, P.M., Gauche, C., Gonzaga, L.V., Costa, A.C.O. and Fett, R., 2016. Honey: Chemical composition, stability and authenticity. *Food chemistry*, *196*, pp.309-323. https://doi.org/10.1016/j.foodchem.2015.09.051
34. de Rodríguez, G.O., de Ferrer, B.S., Ferrer, A. and Rodríguez, B., 2004. Characterization of honey produced in Venezuela. *Food Chemistry*, *84*(4), pp.499-502. https://doi.org/10.1016/S0308-8146(02)00517-4
35. Djamali, M., Akhani, H., Khoshravesh, R., Andrieu-Ponel, V., Ponel, P. and Brewer, S., 2011. Application of the global bioclimatic classification to Iran: implications for understanding the modern vegetation and biogeography. *Ecologia mediterranea*, *37*(1), pp.91-114. https://www.persee.fr/doc/ecmed_0153-8756_2011_num_37_1_1350
36. Easton-Calabria, A., Demary, K.C. and Oner, N.J., 2019. Beyond pollination: honey bees (Apis mellifera) as zootherapy keystone species. *Frontiers in Ecology and Evolution*, *6*, p.161. https://doi.org/10.3389/fevo.2018.00161
37. Efem, S.E.E., 1988. Clinical observations on the wound healing properties of honey. *British journal of Surgery*, *75*(7), pp.679-681. https://doi.org/10.1002/bjs.1800750718

38. El-Senduny, F.F., Hegazi, N.M., Abd Elghani, G.E. and Farag, M.A., 2021. Manuka honey, a unique mono-floral honey. A comprehensive review of its bioactives, metabolism, action mechanisms, and therapeutic merits. *Food Bioscience*, *42*, p.101038. https://doi.org/10.1016/j.fbio.2021.101038

39. Escuredo, O., Silva, L.R., Valentão, P., Seijo, M.C. and Andrade, P.B., 2012. Assessing Rubus honey value: Pollen and phenolic compounds content and antibacterial capacity. *Food Chemistry*, *130*(3), pp.671-678. https://doi.org/10.1016/j.foodchem.2011.07.107

40. Fagih, A.R., Ebadi, R. & Nazarian, H. 2004. Pollen study of flowering plants used by bees in the regions Khansar and Faridan of Isfahan province. Journal of Iran Agriculture Science 35(2): 265-283. https://eurekamag.com/research/004/024/004024250.php

41. Farhadinia, M.S., Soofi, M., Rosen, T., Moghadas, P., Hobeali, K., Behnoud, P., Amanov, A., Dieterich, T., Hojamuradov, H., Hudaikuliev, N., Karryeva, s., Kaczensky, P., Mengliev, s., Muhashov, A., Nurmuhambetov, Z., Pestov, M., Potaeva, A., Terentyev, V., Salmanpour, F., Tizrouyan, H., Alinezhad, H., Jafari, B., Ommatmohammadi, M., Rabie, K., Veyisov, A., Linnell, J., 2022. Status of Persian leopards in northern Iran and Central Asia. CATnews Special. https://www.researchgate.net/publication/362889852

42. Finola, M.S., Lasagno, M.C. and Marioli, J.M., 2007. Microbiological and chemical characterization of honeys from central Argentina. *Food chemistry*, *100*(4), pp.1649-1653. https://doi.org/10.1016/j.foodchem.2005.12.046

43. Fredes, C. and Montenegro, G., 2006. Heavy metal and other trace elements contents in honey bee in Chile. Cien. Inv. Agr.(in English) 33 (1): 50-58. *International Journal of Agriculture and Natural Resources*, *33*(1), pp.50-58. https://revistaschilenas.uchile.cl/handle/2250/10.7764/rcia.v33i1.328

44. García, N.L., 2018. The current situation on the international honey market. *Bee World*, *95*(3), pp.89-94. https://doi.org/10.1080/0005772X.2018.1483814

45. Hailu, D. and Belay, A., 2020. Melissopalynology and antioxidant properties used to differentiate Schefflera abyssinica and polyfloral honey. *Plos one*, *15*(10), p.e0240868. https://doi.org/10.1371/journal.pone.0240868

46. Hedge, I. C., & Wendelbo, P. (1978). Patterns of distribution and endemism in Iran. *Notes Roy. Bot. Gard. Edinburgh*, *36*(2), 441-464. https://www.scopus.com/record/display.uri?eid=2-s2.0-0006003564&origin=inward&txGid=08c1677fbb653bcda02974e2f9bd74ed
47. Jones, G.D. and Bryant Jr, V.M., 1996. New Frontiers in Palynology. *Palynology: Principles and Applications, edited by J. Jansonius and DC McGregor*, pp.933-38. https://scholar.google.com/scholar?hl=en&as_sdt=0%2C5&q=New+Frontiers+in+Palynology.+Palynology%3A+Principles+and+Applications%2C+edited+by+J.+Jansonius+and+DC+McGregor.&btnG=
48. Johnson, D.W., van Eps, C., Mudge, D.W., Wiggins, K.J., Armstrong, K., Hawley, C.M., Campbell, S.B., Isbel, N.M., Nimmo, G.R. and Gibbs, H., 2005. Randomized, controlled trial of topical exit-site application of honey (Medihoney) versus mupirocin for the prevention of catheter-associated infections in hemodialysis patients. *Journal of the American Society of Nephrology*, *16*(5), pp.1456-1462. https://doi.org/10.1681/ASN.2004110997
49. Karabagias, I.K., Badeka, A., Kontakos, S., Karabournioti, S. and Kontominas, M.G., 2014. Characterisation and classification of Greek pine honeys according to their geographical origin based on volatiles, physicochemical parameters and chemometrics. *Food chemistry*, *146*, pp.548-557. https://doi.org/10.1016/j.foodchem.2013.09.105
50. Karimi, A., Nazarian, H., Jafari, E. and Hatami, A., 2016. Identification and determine the distribution of plants used by bees in Galehdar watershed (Fars province). *Natural Ecosystems of Iran*, *7*(3), pp.13-27. https://neijournal.nour.iau.ir/mobile/article_527674.html?lang=en
51. Kaškonienė, V. and Venskutonis, P.R., 2010. Floral markers in honey of various botanical and geographic origins: a review. *Comprehensive reviews in food science and food safety*, *9*(6), pp.620-634. https://doi.org/10.1111/j.1541-4337.2010.00130.x
52. Kečkeš, S., Gašić, U., Veličković, T.Ć., Milojković-Opsenica, D., Natić, M. and Tešić, Ž., 2013. The determination of phenolic profiles of Serbian unifloral honeys using ultra-high-performance liquid chromatography/high resolution accurate mass spectrometry. *Food Chemistry*, *138*(1), pp.32-40.

https://doi.org/10.1016/j.foodchem.2012.10.025

53. Kędzierska-Matysek, M., Stryjecka, M., Teter, A., Skałecki, P., Domaradzki, P. and Florek, M., 2021. Relationships between the content of phenolic compounds and the antioxidant activity of polish honey varieties as a tool for botanical discrimination. *Molecules*, *26*(6), p.1810. https://doi.org/10.3390/molecules26061810

54. Khalil, M.I., Moniruzzaman, M., Boukraâ, L., Benhanifia, M., Islam, M.A., Islam, M.N., Sulaiman, S.A. and Gan, S.H., 2012. Physicochemical and antioxidant properties of Algerian honey. *Molecules*, *17*(9), pp.11199-11215. https://doi.org/10.3390/molecules170911199

55. Khansaritoreh, E., Salmaki, Y., Azirani, T.A., Henareh, F., Alizadeh, K., Ramezani, E., Zarre, S., Beckh, G. and Behling, H., 2021. The sources and quality of Iranian honey. *Heliyon*, *7*(4), p.e06651. https://doi.org/10.1016/j.heliyon.2021.e06651

56. Kier, G., Mutke, J., Dinerstein, E., Ricketts, T.H., Küper, W., Kreft, H. and Barthlott, W., 2005. Global patterns of plant diversity and floristic knowledge. *Journal of Biogeography*, *32*(7), pp.1107-1116. https://doi.org/10.1111/j.1365-2699.2005.01272.x

57. Klein, J.C. and Lacoste, A., 1989. The oak groves of Quercus macranthera F. and M. in the Alborz mountain (Iran) and the eastern extremities of the Great and Little Caucasus. *Ecologia Mediterranea (France)*. https://scholar.google.com/scholar?hl=en&as_sdt=0%2C5&q=%5BThe+oak+groves+of+Quercus+macranthera+F.+and+M.+in+the+Alborz+mountain+%28Iran%29+and+the+eastern+extremities+of+the+Great+and+Little+Caucasus&btnG=

58. Klein, B. and Leffler, K.B., 1981. The role of market forces in assuring contractual performance. *Journal of political Economy*, *89*(4), pp.615-641. https://doi.org/10.1086/260996

59. Kocot, J., Kiełczykowska, M., Luchowska-Kocot, D., Kurzepa, J. and Musik, I., 2018. Antioxidant potential of propolis, bee pollen, and royal jelly: possible medical application. *Oxidative medicine and cellular longevity*, *2018*. https://doi.org/10.1155/2018/7074209

60. Kortesniemi, M., Rosenvald, S., Laaksonen, O., Vanag, A., Ollikka, T., Vene, K. and Yang, B., 2018. Sensory and chemical profiles of Finnish honeys of different botanical origins and consumer preferences. *Food Chemistry*, *246*, pp.351-359. https://doi.org/10.1016/j.foodchem.2017.10.069
61. Kuldasheva, F., Madartov, B., Nurmurodova, N. and Eshdavlatov, O., 2022. STUDY OF ORGANOLEPTIC, PHYSICAL AND CHEMICAL INDICATORS OF HONEY IN TASHKENT REGION. *Agro Inform*, (1), pp.43-51. https://cyberleninka.ru/article/n/study-of-organoleptic-physical-and-chemical-indicators-of-honey-in-tashkent-region
62. Louveaux, J., Maurizio, A., & Vorwohl, G. (1978). Methods of melissopalynology. Bee world, 59(4), 139-157. https://doi.org/10.1080/0005772X.1978.11097714
63. Louveaux, J. and Maurizio, A., G. vorwohl. 1970. Methods of melissopalynology. *Bee world*, *51*, pp.25-138. https://doi.org/10.1080/0005772X.1970.11097312
64. Machado De-Melo, A.A., Almeida-Muradian, L.B.D., Sancho, M.T. and Pascual-Maté, A., 2018. Composition and properties of Apis mellifera honey: A review. *Journal of apicultural research*, *57*(1), pp.5-37. https://doi.org/10.1080/00218839.2017.1338444
65. Manzanares, A.B., García, Z.H., Galdón, B.R., Rodríguez-Rodríguez, E.M. and Romero, C.D., 2017. Physicochemical characteristics and pollen spectrum of monofloral honeys from Tenerife, Spain. *Food chemistry*, *228*, pp.441-446. https://doi.org/10.1016/j.foodchem.2017.01.150
66. Martos, I., Ferreres, F., Yao, L., D'Arcy, B., Caffin, N. and Tomás-Barberán, F.A., 2000. Flavonoids in monospecific Eucalyptus honeys from Australia. *Journal of Agricultural and Food Chemistry*, *48*(10), pp.4744-4748. https://doi.org/10.1021/jf000277i
67. Maurizio, A., 1975. Microscopy of honey. *Honey: A Comprehensive Survey. E. Crane, ed.* https://scholar.google.com/scholar?hl=en&as_sdt=0%2C5&q=Maurizio%2C+A.+1975.+Microscopy+of+honey.+%E2%80%93+In%3A+Honey%3A+&btnG=
68. Mehrabian, A. and Rad, M.A., 2021. Distribution patterns and priorities for conservation of Iranian Endemic Monocots: determining the Areas of Endemism (AOEs). *Journal of Wildlife and Biodiversity*, *5*(2), pp.69-87. https://doi.org/10.22120/jwb.2020.136616.1188

69. Memariani, F. & Ghoraishi Alhossein, J. 2000. Pollen study of some samples of honey in Khorasan province. Pajohesh va Sazandegi 49: 76-83.
70. Menad, H., Ben-Naoum, F. and Amine, A., 2020. A Hybrid Grey Wolves Optimizer and Convolutional Neural Network for Pollen Grain Recognition. *International Journal of Swarm Intelligence Research (IJSIR)*, *11*(3), pp.49-71. https://doi.org/10.4018/IJSIR.2020070104
71. Molan, P., 2001. Why honey is effective as a medicine: 2. The scientific explanation of its effects. *Bee world*, *82*(1), pp.22-40. https://doi.org/10.1080/0005772X.2001.11099498
72. Mureșan, C.I., Cornea-Cipcigan, M., Suharoschi, R., Erler, S. and Mărgăoan, R., 2022. Honey botanical origin and honey-specific protein pattern: Characterization of some European honeys. *LWT*, *154*, p.112883. https://doi.org/10.1016/j.lwt.2021.112883
73. Naqinezhad, A. and Esmailpoor, A., 2017. Flora and vegetation of rocky outcrops/cliffs near the Hyrcanian forest timberline in the Mazandaran mountains, northern Iran. *Nordic Journal of Botany*, *35*(4), pp.449-466. https://doi.org/10.1111/njb.01384
74. Nanda, V., Sarkar, B.C., Sharma, H.K. and Bawa, A.S., 2003. Physico-chemical properties and estimation of mineral content in honey produced from different plants in Northern India. *Journal of Food Composition and Analysis*, *16*(5), pp.613-619. https://doi.org/10.1016/S0889-1575(03)00062-0
75. Nayik, G.A. and Nanda, V., 2016. Application of response surface methodology to study the combined effect of temperature, time and pH on antioxidant activity of cherry (Prunus avium) honey. *Polish Journal of Food and Nutrition Sciences*, *66*(4), pp.287-293. https://doi.org/10.1515/pjfns-2015-0055
76. Nazarian, H., Sanei Shariat Panahi, M. & tahmasebi, G.H. 1997. Determination of Identification of plants used by bees in Tehran province. The second Conference on Honey bee. Iran Animal Science Research Institute.
77. Noroozi, J. ed., 2020. *Plant biogeography and vegetation of high mountains of Central and South-West Asia*. Springer International Publishing. https://doi.org/10.1007/978-3-030-45212-4

78. Noroozi, J., Pauli, H., Grabherr, G. and Breckle, S.W., 2011. The subnival–nival vascular plant species of Iran: a unique high-mountain flora and its threat from climate warming. *Biodiversity and Conservation*, *20*(6), pp.1319-1338. https://doi.org/10.1007/s10531-011-0029-9
79. Olivieri, C., Marota, I., Rollo, F. and Luciani, S., 2012. Tracking plant, fungal, and bacterial DNA in honey specimens. *Journal of Forensic Sciences*, *57*(1), pp.222-227. https://doi.org/10.1111/j.1556-4029.2011.01964.x
80. Ouchemoukh, S., Louaileche, H. and Schweitzer, P., 2007. Physicochemical characteristics and pollen spectrum of some Algerian honeys. *Food control*, *18*(1), pp.52-58. https://doi.org/10.1016/j.foodcont.2005.08.007
81. Ozturk, F.; Erkan, C. Bee plants of Van Lake Basin (Turkey). *Int. J. Bot.* **2010**, *6*, 101–106. https://www.cabdirect.org/cabdirect/abstract/20103306252
82. Pasupuleti, V.R., Sammugam, L., Ramesh, N. and Gan, S.H., 2017. Honey, propolis, and royal jelly: a comprehensive review of their biological actions and health benefits. *Oxidative medicine and cellular longevity*, *2017*. https://doi.org/10.1155/2017/1259510
83. Ponnuchamy, R., Bonhomme, V., Prasad, S., Das, L., Patel, P., Gaucherel, C., Pragasam, A. and Anupama, K., 2014. Honey pollen: using melissopalynology to understand foraging preferences of bees in tropical South India. *PLoS One*, *9*(7), p.e101618. https://doi.org/10.1371/journal.pone.0101618
84. Puusepp, L. and Koff, T., 2014. Pollen analysis of honey from the Baltic region, Estonia. *Grana*, *53*(1), pp.54-61. https://doi.org/10.1080/00173134.2013.850532
85. Rao, P.V., Krishnan, K.T., Salleh, N. and Gan, S.H., 2016. Biological and therapeutic effects of honey produced by honey bees and stingless bees: a comparative review. *Revista Brasileira de Farmacognosia*, *26*, pp.657-664. https://doi.org/10.1016/j.bjp.2016.01.012
86. Rašić, S., Štefanić, E., Antunović, S., Jović, J. and Kristek, S., 2018. Pollen analysis of honey from north-eastern Croatia. *Poljoprivreda*, *24*(2), pp.43-49. https://doi.org/10.18047/poljo.24.2.6
87. Razzaghy Kamroudi Mehdi, Sanei Shariat Panahi Mohammad, Nazarian Hassan, Qelichnia Hassan. Identification of pollens in honeys of Nourrud watershed of Mazandaran. Research

and construction. 2006 [cited 2022May14]; 19 (3 (p. 72) on livestock and aquaculture): 74-83.

88. Ronsisvalle, S., Lissandrello, E., Fuochi, V., Petronio Petronio, G., Straquadanio, C., Crascì, L., Panico, A., Milito, M., Cova, A.M., Tempera, G. and Furneri, P.M., 2019. Antioxidant and antimicrobial properties of Casteanea sativa Miller chestnut honey produced on Mount Etna (Sicily). *Natural product research*, *33*(6), pp.843-850. https://doi.org/10.1080/14786419.2017.1413568
89. Safi Ahmad Abad, M., Shirvany, A., Panahi, P. (2017). 'Comparison of pollen grain composition and quality of honey obtained from forest and rangeland sites in Arasbaran region', *Iranian Journal of Forest and Poplar Research*, 25(2), pp. 342-353. doi: 10.22092/ijfpr.2017.111783 https://dx.doi.org/10.22092/ijfpr.2017.111783
90. Salmanpour, F., Shakoori, Z., Kia, M. and Ghomi, S., 2021. Investigating the Factors Affecting Leopard (Panthera pardus tulliana) Conflict with Domestic Livestock Compared to other Large Carnivores in Nowshahr Hyrcanian Forests. *Journal of Animal Environment*, *13*(2), pp.1-8. doi: 10.22034/AEJ.2021.135105 https://www.aejournal.ir/article_135105.html?lang=en
91. Sanei Shariat Panahi, M. & Said Abadi, V. 1974. Nectriferous plants in Karaj based on palynology. Journal of Agricultural Faculty, 2-3: 35-47.
92. Saxena, S., Gautam, S. and Sharma, A., 2010. Physical, biochemical and antioxidant properties of some Indian honeys. *Food chemistry*, *118*(2), pp.391-397. https://doi.org/10.1016/j.foodchem.2009.05.001
93. Selvaraju, K., Vikram, P., Soon, J.M., Krishnan, K.T. and Mohammed, A., 2019. Melissopalynological, physicochemical and antioxidant properties of honey from West Coast of Malaysia. *Journal of food science and technology*, *56*(5), pp.2508-2521. https://doi.org/10.1007/s13197-019-03728-3
94. Shakoori, Z., Mehrabian, A., Salmanpour, F., 2022. Evaluation of plant origin of honey based on pollen evidence in some prominent honey-producing zones in Iran, 47th Apimondia Congress (0355), pp-124Ref.0355. https://www.researchgate.net/publication/363134174
95. Stocklin, J., 1977. Structural correlation of the Alpine ranges between Iran and Central Asia.https://pascal-

francis.inist.fr/vibad/index.php?action=getRecordDetail&idt=PASCALGEODEBRGM7720435256

96. Stöcklin, J., 1974. Northern iran: Alborz mountains. *Geological Society, London, Special Publications*, *4*(1), pp.213-234. https://doi.org/10.1144/GSL.SP.2005.004.01.12

97. Subrahmanyam, M., 1998. A prospective randomised clinical and histological study of superficial burn wound healing with honey and silver sulfadiazine. *Burns*, *24*(2), pp.157-161. https://doi.org/10.1016/S0305-4179(97)00113-7

98. Tucak, Z.; Tucak, A.; Puškadija, Z.; Tucak, M. Nutritious healing composition of some kinds of honey in Eastern Croatia. *Agriculture* **2000**, *6*, 129–132.

99. Vaghefi, S.A., Keykhai, M., Jahanbakhshi, F., Sheikholeslami, J., Ahmadi, A., Yang, H. and Abbaspour, K.C., 2019. The future of extreme climate in Iran. *Scientific reports*, *9*(1), pp.1-11. https://doi.org/10.1038/s41598-018-38071-8

100. Von Der Ohe, W., Oddo, L.P., Piana, M.L., Morlot, M. and Martin, P., 2004. Harmonized methods of melissopalynology. *Apidologie*, *35*(Suppl. 1), pp.S18-S25. DOI: 10.1051/apido:2004050 https://www.apidologie.org/articles/apido/pdf/2004/06/MHS07.pdf

101. Yao, L., Datta, N., Tomás-Barberán, F.A., Ferreres, F., Martos, I. and Singanusong, R., 2003. Flavonoids, phenolic acids and abscisic acid in Australian and New Zealand Leptospermum honeys. *Food Chemistry*, *81*(2), pp.159-168. https://doi.org/10.1016/S0308-8146(02)00388-6

Books:

102. Davis SD, Heywood VH, Hamilton AC. Centres of plant diversity. Natural History. 1994;111(1):1-0.

103. Dewan, M.L. and Famouri, J., 1964. *The soils of Iran*. Food and Agriculture Organization of the United Nations.

104. Ghelich Nia, H. 2017. Vegetation types of Mazandaran. Research Institute of Forests and Rangelands.

105. Fayaz, M. 2015. Vegetation types of Tehran. Research Institute of Forests and Rangelands.

106. Stöcklin, J., 1968. Salt deposits of the Middle East.

107. Takhtajan, A. 1986: Floristic regions of the world. - Berkeley: University of California Press.Volume 7, Issue 4, April 2021, e06651.

108. Zahran, M.A., 2010. *Climate-Vegetation:: Afro-Asian Mediterranean and Red Sea Coastal Lands* (Vol. 4). Springer Science & Business Media.

109. Zimmerman, M.I.C.H.A.E.L., 1988. Nectar production, flowering phenology, and strategies for pollination. *Plant reproductive ecology: patterns and strategies*, *41*, pp.157-178.

110. Zohary, M., 1973. *Geobotanical foundations of the Middle East*. Fischer.

Thesis:

111. Dewan ML, Famouri J. *The Soils of Iran, FAO, Rome* (Doctoral dissertation, Thesis presented to the)1963.

112. Klein R. *The new politics of the National Health Service* (Doctoral dissertation, Texas Tech University),1972.

Website:

113. UNESCO (United Nations Educational Scientific and Cultural Organization). 2019. https://www.whc.unesco. org/en/list/1584. Hyrcanian Forests.

114. FAOSTAT, 2018. Production of Natural Honey in World. http://www.fao.org/faostat/en/#data/QL/visualize
