# Supplementary material for: Assessing the quality of bee honey on the basis of melissopalynology as well as chemical analysis
Source: PLoS One. 2023 Aug 8;18(8):e0289702. doi: 10.1371/journal.pone.0289702 (PMC10409281; doi:10.1371/journal.pone.0289702)
Supplement: Attachment — Submitted filename: PONE-D-22-31247_R1-H.pdf [file pone.0289702.s005.pdf]

PLOS ONE

Assessing the Quality of Bee Honey on the basis of melissopalynology as well chemical analysis

--Manuscript Draft--

**Manuscript Number:**	PONE-D-22-31247R1
**Article Type:**	Research Article
**Full Title:**	Assessing the Quality of Bee Honey on the basis of melissopalynology as well chemical analysis
**Short Title:**	Assessing the Quality of Bee Honey on the basis of melissopalynology as well chemical analysis
**Corresponding Author:**	Ahmadreza Mehrabian, PhD.<br>Shahid Beheshti University<br>Tehran, IRAN, ISLAMIC REPUBLIC OF
**Keywords:**	Melissopalynology, Botanical origin, Scanning Electron Microscopy (SEM), Iran, Pollen, palynology
**Abstract:**	Melissopalynological analysis and chemical composition of honey provide us with useful and valuable information about the botanical and geographical origin of honey. The data in question is very important for authentication as well as for testing the quality of honey, so this is considered the main method in honey regulation here, we have used chemical analysis and melissopalynology to evaluate different honey samples from two main Iranian hubs of honey. Sampling was carried out on two important poles in Iran's honey production, the central Alborz region, and the mountainous Zagros ecosystems in the years 2020 to 2021. Therefore, 52 samples from Alborz (Northern Iran), as well as 42 samples from the Zagrosian ecosystems (western Iran) belonging to different ecological habitats, were collected. In addition, samples were taken at 7 altitudes from 0 to 3500 m a.s.l. Furthermore, in this study, various chemical analyses such as the effect of antioxidant activity, the amount of total phenolic content, pH, and moisture content of honey samples were evaluated. Our results showed that all honey samples were classified as polyfloral honey. Based on our findings, 57 honey samples (61%) contained the standard amount of pollen. A total of 42 plant families and 55 genera were identified in the studied samples, with the highest presence of Asteraceae, Fabaceae, Rosaceae, Apocynaceae, and Apiaceae. Finally, an antioxidant activity ratio of 19% to 98%, total phenolic content from 0.08 to 0.51 ppm, pH from 1.90 to 5.21, and moisture content from 13% to 18.40%.
**Order of Authors:**	zahra shakoori, masterS degree
	Ahmadreza Mehrabian, PhD.
	Dariush Minai
	Farid Salmanpour, Masters degree
	Farzaneh Khajoei Nasab
**Response to Reviewers:**	Dear Dr. Daniel de Paiva Silva<br><br>Thank you for providing us with the opportunity to submit a revised draft of the manuscript PONE-D-22-31247 entitled " Assessing the Quality of Bee Honey on the basis of melissopalynology as well chemical analysis ". We appreciate the time and effort that you and the reviewers have dedicated to providing valuable feedback on our manuscript. We are grateful to the reviewers for their insightful comments on the paper. We have been able to incorporate changes to reflect all the suggestions provided and all changes are highlighted in the manuscript text file.<br><br>Please see below a point-by-point response to the reviewers’ comments and concerns.<br><br>Best wishes<br>Ahmadreza Mehrabian

Powered by Editorial Manager® and ProduXion Manager® from Aries Systems Corporation

Dear Editor
Comments to the Author:
After this first review round, all three reviewers raised significant issues that prevent the publication of your manuscript at this time. Still, the reviewers indicated that the manuscript has a potential to be published in PLoS One after improvements are considered.
Response: We appreciate the positive comments of the reviewers and have done our best to incorporate all comments, with specific responses below.
If applicable, we recommend that you deposit your laboratory protocols in protocols.io to enhance the reproducibility of your results. Protocols.io assigns your protocol its own identifier (DOI) so that it can be cited independently in the future.
Response: We saved our data in the figshare repository and placed the link in the text of the manuscript.
DATA AVAILABILITY STATEMENT
DOI: 10.6084/m9.figshare.22586863
https://figshare.com/s/949e02f3163f39991d4e
Journal requirements:
1. Please ensure that your manuscript meets PLOS ONE's style requirements, including those for file naming. The PLOS ONE style templates can be found at https://journals.plos.org/plosone/s/file?id=wjVg/PLOSOne_formatting_sample_main_body.pdf and https://journals.plos.org/plosone/s/file?id=ba62/PLOSOne_formatting_sample_title_authors_affiliations.pdf.
Response: We prepared the manuscript with PLOS ONE style requirements, including file naming.
2. We noticed you have some minor occurrence of overlapping text with the following previous publication(s), which needs to be addressed:
Plant Biogeography and Vegetation of High Mountains of Central and South-West Asia - https://link.springer.com/book/10.1007/978-3-030-45212-4, and
A bioclimatic characterization of high elevation habitats in the Alborz mountains of Iran - https://doi.org/10.1007/s00035-018-0202-9.
In your revision ensure you cite all your sources (including your own works), and quote or rephrase any duplicated text outside the methods section. Further consideration is dependent on these concerns being addressed.
Response: We added "A bioclimatic characterization of high elevation habitats in the Alborz mountains of Iran - https://doi.org/10.1007/s00035-018-0202-9" in our study area section:
The Alborz mountain range is located at 36° N from the Caspian Sea up to a height of 5671 meters (Noroozi et al.2018).

3. In your Methods section, please provide additional information regarding the permits you obtained for the work. Please ensure you have included the full name of the authority that approved the field site access and, if no permits were required, a brief statement explaining why.
Response: In the above study, we did not need a permit because the honey samples were provided to us voluntarily by the beekeepers, and because there was no need to enter protected areas or national parks, we did not need to obtain a permit.
Statement: In the above study, the beekeepers provided us with honey samples voluntarily and with full consent, and we did not need to obtain a permit due to not entering protected areas or national parks.

4. Please update your submission to use the PLOS LaTeX template. The template and more information on our requirements for LaTeX submissions can be found at http://journals.plos.org/plosone/s/latex.
Response: Corrections were made.
5. We note that you have stated that you will provide repository information for your

data at acceptance. Should your manuscript be accepted for publication, we will hold it until you provide the relevant accession numbers or DOIs necessary to access your data. If you wish to make changes to your Data Availability statement, please describe these changes in your cover letter and we will update your Data Availability statement to reflect the information you provide.
Response: We saved our laboratory protocols and data in the figshare repository and placed the link in the text of the manuscript.
DATA AVAILABILITY STATEMENT
DOI: 10.6084/m9.figshare.22586863
https://figshare.com/s/949e02f3163f39991d4e

6. PLOS requires an ORCID iD for the corresponding author in Editorial Manager on papers submitted after December 6th, 2016. Please ensure that you have an ORCID iD and that it is validated in Editorial Manager. To do this, go to 'Update my Information' (in the upper left-hand corner of the main menu), and click on the Fetch/Validate link next to the ORCID field. This will take you to the ORCID site and allow you to create a new iD or authenticate a pre-existing iD in Editorial Manager. Please see the following video for instructions on linking an ORCID iD to your Editorial Manager account: https://www.youtube.com/watch?v=_xcclfuvtxQ.
Response: The ORCID of each author was declared.
Zahra Shakoori: https://orcid.org/0000-0003-3057-8981
Ahmadreza Mehrabian: https://orcid.org/0000-0001-6633-3092
Dariush Minai: https://orcid.org/0000-0003-3589-7324
Farid Salmanpour: https://orcid.org/0000-0002-1517-9061
Farzaneh Khajoei Nasab: https://orcid.org/0000-0002-2325-9555

Dear Prof. Ing. Jaromir Lachman, Reviewer #1:
To the article, I have next comments and recommendations:
• In the article, there are many typing errors, which should be carefully checked and corrected, e.g., in Abstract: you should use 0.08 to 0.05 ppm or 0.08-0.05 and so on throughout the text. Like wise L. 47:…climate of the region…; L. 61:…botanical…;, Table 1: Bignoniaceae L. 98: instead antioxidative should be used antioxidant activity or antioxidant capacity and similar in the all article.
Response: We tried to carefully check and correct typographical errors. For example, we used 0.08 to 0.05 pp throughout the text. Instead of antioxidant, we used antioxidant activity in the article. Other mentioned points such as amendment in L. 46:...climate of the region...; L. 58:…botanical…;, Table 1: Bignoniaceae done.
• Latin names of plants should be in Italics through all text and References, e.g., L. 140: Quercus macranthera, etc.
Response: The Latin names of the plants were written in italics in all texts and references.
• All applied equipment and chemicals should be carefully completed with the name of the producer, town and country (state) – check and complete the missing date especially in section Laboratory studies.
Response: The name of the manufacturer, city and country (state) of all equipment and chemicals used were carefully entered with the name of the manufacturer, especially in the laboratory studies section.
-L.212: Hot plate model HP100 made by Mtops company in South Korea
-L.216: Centrifuge model TDL-4 made by Jenius China
-L.247: Merck Chemical Company in Germany.
-L.248: 2,2-Diphenyl-1-picrylhydrazyl (DPPH) was obtained from Sigma American Chemical.
-258: JENUS V-1100 spectrophotometer Made by DLAB China

•In the centrifugation convert rpm to g values and correct centrifugation instead centrifuge(s), where it is appropriate.
Response: In the centrifuge, we converted revolutions per minute to g values and corrected centrifugation instead of centrifuge where appropriate.

•L. 637: 1% ferric chloride…

Response: The item mentioned in L. 637 was not found, but it was found in line 264 and corrected.

•Unify Apocynaceae & Apiaceae or Apocynaceae and Apiaceae, similarly throughout the text.
Response: Apocynaceae and Apiaceae are similarly united throughout the text.

•L. 294 and elsewhere: total phenolic content or total phenolics.
Response: Throughout the text, the phenol compounds and total phenol were changed to total phenolic content.

•Some sentences are completely confusing and should be corrected, e.g. L. 336-337:…in the Estonia (Puusepp and Koff 2014). Croatia (?). Our results show…
Response: Confusing sentences were completely corrected throughout the text.

•The main problem is that the citation system should be substantially redone according to system used in PLOS One, i.e., using brackets like [1, [2,[3, etc. and hence the references they will not be in alphabetical order.
Response: In this version, we have adjusted the entire manuscript according to PLOS One guidelines.

•According to this system you should change the order of references. In References you should apply Italics in Latin names of plants (Prunus avium, Schefflera abyssinica) and bees (Apis mellifera), use the full titles of Journals with capitals of nouns and adjectives, like Food Chemistry, Nutrition Research, International Journal of Food Microbiology, Current Medicinal Chemistry, LWT-Food Science and Technology, etc., do not use Italics in titles of journals (not mixture). Do not use capitals in the name of article – should be e.g., Study of organoleptic, physical, and chemical indicators of honey in Tashkent region. Do not use pp. and p. in references, by the way some articles in new volumes use an article number (one number) instead of pages.
Response: We modified and prepared all the references according to the instructions of the journal and all the mentioned points were applied.

•Books, Theses and Website should be implemented along journals referenced with their citation number.
Here you are some examples:
1. Hailu D, Belay A. Melissopalynology and antioxidant properties used to differentiate Schefflera abyssinica and polyfloral honey. PLOS One. 2020;15(10): e0240868.https://doi.org/10.1371/journal.pone.0240868
2. Alvarez-Suarez MJ., Giampieri F, Battino M. Honey as a source of dietary antioxidants: structures, bioavailability and evidence of protective effects against human chronic diseases. Current Medicinal Chemistry. 2013; 20(5), 621-638. https://doi.org/10.2174/092986713804999358
3. Berberian M.The southern Caspian: a compressional depression floored by a trapped, modified oceanic crust. Canadian Journal of Earth Sciences. 1983; 20(2): 163-183. https://doi.org/10.1139/e83-015
4. Chua LS, Rahaman NL, Adnan NA, Eddie Tan, TT. Antioxidant activity of three honey samples in relation with their biochemical components. Journal of Analytical Methods in Chemistry. 2013: 313798. https://doi.org/10.1155/2013/313798
5. El-Senduny FF, Hegazi NM, Abd Elghani GE, Farag MA. Manuka honey, a unique mono-floral honey. A comprehensive review of its bioactives. metabolism, action mechanisms, and therapeutic merits. Food Bioscience. 2021; 42:101038. https://doi.org/10.1016/j.fbio.2021.101038
6. Hedge IC, Wendelbo P. Patterns of distribution and endemism in Iran. Notes from the Royal Botanic Garden, Edinburgh. 1978; 36(2): 441-464. https://www.scopus.com/record/display.uri?eid=2-s2.0-0006003564&origin=inward&txGid=08c1677fbb653bcda02974e2f9bd74ed
7. Kędzierska-Matysek M, Stryjecka M, Teter A, Skałecki P, Domaradzki P, Florek M. 2021. Relationships between the content of phenolic compounds and the antioxidant activity of Polish honey varieties as a tool for botanical discrimination. Molecules. 2021; 26(6): 1810. https://doi.org/10.3390/molecules26061
Response: We have made the mentioned corrections completely.

Dear Reviewer #2:

1.Authors stated in line 105 that “the study tries to analyze the mentioned data in

	relation to geographical factors." and they try to analyze this aspect in the section 5. Discussion. However, for drawing conclusions if chemical parameters of honey depend on geographical factors, the proper statistical analysis is needed.<br>Response: This section has been completely revised.<br><br>2.The sentence "We compared the humidity of honey samples with the humidity of the areas from which the samples were collected, which shows that the humidity of the environment affected the humidity of honey samples" must be proven by the statistical analysis.<br>Response: We removed the map related to the effect of ambient humidity on the humidity of honey samples and using the minitab software, we got regression fitted line plots statistical model.<br>The regression result between the average annual relative humidity of the region (independent variable) and the humidity of the honey sample (dependent variable) showed that there is a significant relationship (p value 0.000) between them, so that the humidity of honey samples is a function of the humidity of the environment.<br><br>Fig 5. Comparison of moisture content of honey samples with moisture content of the collection site<br><br>Fig 5. Regression related to the effect of environmental humidity on the moisture of honey samples<br><br>3.On Figure. 6 the font size on the axis descriptions must be increased for greater readability. It is also no clear for me, what is the Loading. Do Authors mean coefficient of correlation?<br>Response: In this section, more detailed reforms were needed, and these reforms were carried out.<br>Yes, loading means the same coefficient of correlation that was modified in the figure.<br><br>4.The sentence in lines 299-302 should be corrected. It is completely incomprehensible.<br>Response: In this section, more detailed reforms were needed, and these reforms were carried out.<br><br>5.Figure 8 – Authors should inform where the reader can find the explanation of codes of samples. On this picture Authors depict results of cluster analysis but I can't find in the text how these results are used, or which conclusions were drawn base on them.<br>Response: We saved our data in the figshare repository and placed the link in the text of the manuscript, codes can be found in it.<br>DOI: 10.6084/m9.figshare.22586863<br>https://figshare.com/s/949e02f3163f39991d4e<br>In addition, this section was modified in the text of the manuscript and some information was included in the text of the manuscript.<br><br>6.Text must be linguistically and editorially corrected.<br>Response: The text was linguistically and editorially corrected.<br><br>Dear Dr. Haya i. Aljohar, Reviewer #3<br>-Introduction<br>-The introduction consists of many staccato sentences, forcing the author to use an infinite number of linking phrases, such as: as well as , Furthermore, in addition, Therefore etc (Rewrite the entire introduction in proper English).<br>Response: We rewrote the entire introduction in proper English.<br><br>oLine 49 remove(the)<br>Response: It was corrected.

oLine 51 use counterfeit instead of fake
Response: Counterfeit replaced fake.

oLine 54 use and instead of as well as and change has to have.
Response: It was corrected

oLine 56 (Louveaux (1970, 1978)) Add reference
Response: Line59: Reference added (Louveaux et al. 1970; 1978)

oLine 59- 65 Rewrite this paragraph (There are many separate sentences that must be linked together and rewritten in proper English).
Response: We have rewritten this paragraph in proper English.

oLine 73 (SW Asia) It was mentioned here for the first time, so it must be written without abbreviation.
Response: Line 78: Southwest Asia is written instead of SW Asia

oRewrite the paragraph from line 69-78.
Response: The text of the manuscript was completely rewritten and revised

-Study area
-Rewrite in a proper English
Response: All the text of the manuscript was rewritten in proper English.

-Field studies Change to sample collection
Response: Field studies Changed to sample collection

oLine 179 What is the meaning of amount of hight.
Response: We mean the same height.
Line 197: The amount of hight was changed to height

-Laboratory studies

oIt is better to put a paragraph for the chemicals and reagents used and another paragraph for the devices and tools used in the experiment separately.
Response: We have completely revised this paragraph and tried to separate the content as much as possible in the same paragraph to make it clearer.

oRewrite in a proper English.
Response: All the text of the manuscript was rewritten in proper English.

oLine 192 Then, remove large …. By passed through
Response: corrections were made

oLine 223 DPPH abbreviation for what? It was mentioned here for the first time, so it must be written without abbreviation.
Response: Line 248: DPPH was written in full.
2,2-Diphenyl-1-picrylhydrazyl (DPPH) was obtained from Sigma American Chemical Company.

-Line 237(1%) Repeated twice in the sentence why?
Response: $FeCl_3$ 1% is the abbreviation of 1% ferric chloride solution, that's why 1% is repeated twice in the sentence.
Line 264: The sentence was modified in this way: In this experiment, 1% ferric chloride solution ($FeCl_3$ 1%)...

-Results

oRewrite scientifically in a proper English
Response: It was scientifically rewritten in appropriate English

oAdd a table containing honey samples names, symbols, and geographical areas
Response: We have added the requested information and stored it in the figshare

Powered by Editorial Manager® and ProduXion Manager® from Aries Systems Corporation

	repository as backup information.<br><br>DATA AVAILABILITY STATEMENT<br>DOI: 10.6084/m9.figshare.22586863<br>https://figshare.com/s/949e02f3163f39991d4e<br><br><br>oLine 273 Was in the range instead of was the lowest.<br>Response: Line 339: It was corrected.<br><br>oFigure 6 Not clear<br>Response: Response: In this section, more detailed reforms were needed, and these reforms were carried out.<br><br><br><br>oLine 299-304 This part is incomprehensible and needs reformulation<br>Response: Line 424 – 427: The paragraph has been completely revised.<br>The PCA did not result in any significant differences between all honey samples studied (Fig. 9). Considering that the honey samples studied were collected from different geographical areas, samples from the same area were not placed next to each other in the plot. This shows that honey samples from different locations in the same area have different chemical compositions.<br><br>-Unify the method of writing references within the article<br>Response: Unify the references in the article were prepared as based on the journal's guidelines.<br><br>-Line 348 Rephrasing<br>Response: Rewriting done
**Additional Information:**	
**Question**	**Response**
**Financial Disclosure**<br><br>Enter a financial disclosure statement that describes the sources of funding for the work included in this submission. Review the submission guidelines for detailed requirements. View published research articles from *PLOS ONE* for specific examples.<br><br>This statement is required for submission and **will appear in the published article** if the submission is accepted. Please make sure it is accurate.	The authors received no specific funding for this work.

**Unfunded studies**<br>Enter: *The author(s) received no specific funding for this work.*<br><br>**Funded studies**<br>Enter a statement with the following details:<br>• Initials of the authors who received each award<br>• Grant numbers awarded to each author<br>• The full name of each funder<br>• URL of each funder website<br>• Did the sponsors or funders play any role in the study design, data collection and analysis, decision to publish, or preparation of the manuscript?<br>• **NO** - Include this sentence at the end of your statement: *The funders had no role in study design, data collection and analysis, decision to publish, or preparation of the manuscript.*<br>• **YES** - Specify the role(s) played.<br><br>* typeset	
**Competing Interests**<br><br>Use the instructions below to enter a competing interest statement for this submission. On behalf of all authors, disclose any competing interests that could be perceived to bias this work—acknowledging all financial support and any other relevant financial or non-financial competing interests.<br><br>This statement is required for submission and **will appear in the published article** if the submission is accepted. Please make sure it is accurate and that any funding sources listed in your Funding Information later in the submission form are also declared in your Financial Disclosure statement.<br><br>View published research articles from *PLOS ONE* for specific examples.	The authors have declared that no competing interests exist.

**NO authors have competing interests**<br><br>Enter: *The authors have declared that no competing interests exist.*<br><br>**Authors with competing interests**<br><br>Enter competing interest details beginning with this statement:<br><br>*I have read the journal's policy and the authors of this manuscript have the following competing interests: [insert competing interests here]*<br><br>* typeset	
**Ethics Statement**<br><br>Enter an ethics statement for this submission. This statement is required if the study involved:<br><br>• Human participants<br>• Human specimens or tissue<br>• Vertebrate animals or cephalopods<br>• Vertebrate embryos or tissues<br>• Field research<br><br>Write "N/A" if the submission does not require an ethics statement.<br><br>General guidance is provided below. Consult the submission guidelines for detailed instructions. **Make sure that all information entered here is included in the Methods section of the manuscript.**	We have only collected honey samples in this study.

**Format for specific study types**<br><br>**Human Subject Research (involving human participants and/or tissue)**<br>• Give the name of the institutional review board or ethics committee that approved the study<br>• Include the approval number and/or a statement indicating approval of this research<br>• Indicate the form of consent obtained (written/oral) or the reason that consent was not obtained (e.g. the data were analyzed anonymously)<br><br>**Animal Research (involving vertebrate animals, embryos or tissues)**<br>• Provide the name of the Institutional Animal Care and Use Committee (IACUC) or other relevant ethics board that reviewed the study protocol, and indicate whether they approved this research or granted a formal waiver of ethical approval<br>• Include an approval number if one was obtained<br>• If the study involved *non-human primates*, add *additional details* about animal welfare and steps taken to ameliorate suffering<br>• If anesthesia, euthanasia, or any kind of animal sacrifice is part of the study, include briefly which substances and/or methods were applied<br><br>**Field Research**<br><br>Include the following details if this study involves the collection of plant, animal, or other materials from a natural setting:<br>• Field permit number<br>• Name of the institution or relevant body that granted permission	
**Data Availability**<br><br>Authors are required to make all data underlying the findings described fully available, without restriction, and from the time of publication. PLOS allows rare exceptions to address legal and ethical concerns. See the PLOS Data Policy and FAQ for detailed information.	Yes - all data are fully available without restriction

A Data Availability Statement describing where the data can be found is required at submission. Your answers to this question constitute the Data Availability Statement and **will be published in the article**, if accepted.<br><br>**Important:** Stating 'data available on request from the author' is not sufficient. If your data are only available upon request, select 'No' for the first question and explain your exceptional situation in the text box.<br><br>Do the authors confirm that all data underlying the findings described in their manuscript are fully available without restriction?	
**Describe where the data may be found in full sentences. If you are copying our sample text, replace any instances of XXX with the appropriate details.**<br><br>• If the data are **held or will be held in a public repository**, include URLs, accession numbers or DOIs. If this information will only be available after acceptance, indicate this by ticking the box below. For example: *All XXX files are available from the XXX database (accession number(s) XXX, XXX.*).<br>• If the data are all contained **within the manuscript and/or Supporting Information files**, enter the following: *All relevant data are within the manuscript and its Supporting Information files.*<br>• If neither of these applies but you are able to provide **details of access elsewhere**, with or without limitations, please do so. For example:<br><br>*Data cannot be shared publicly because of [XXX]. Data are available from the XXX Institutional Data Access / Ethics Committee (contact via XXX) for researchers who meet the criteria for access to confidential data.*<br><br>*The data underlying the results presented in the study are available from (include the name of the third party*	The data obtained in this study are placed in the figshare repository and its link is mentioned at the end of the manuscript, before the references.<br>DATA AVAILABILITY STATEMENT<br>DOI: 10.6084/m9.figshare.22586863<br>https://figshare.com/s/949e02f3163f39991d4e

and contact information or URL).<br>• This text is appropriate if the data are owned by a third party and authors do not have permission to share the data.<br><br>* typeset	
Additional data availability information:	Tick here if the URLs/accession numbers/DOIs will be available only after acceptance of the manuscript for publication so that we can ensure their inclusion before publication.

Powered by Editorial Manager® and ProduXion Manager® from Aries Systems Corporation

Manuscript

Click here to access/download;Manuscript;Manuscript without track change.docx

Assessing the Quality of Bee Honey on the Basis of Melissopalynology as well as Chemical Analysis

Zahra Shakoori[1], Ahmadreza Mehrabian[*1], Dariush Minai[1], Farid Salmanpour[2], Farzaneh Khajoei Nasab[1]

[1] Department of Plant Science and Biotechnology, Faculty of Science and Biotechnology, Shahid Beheshti University, Tehran, Iran, Email:
zahra_shakoori.eco@yahoo.com, https://orcid.org/0000-0003-3057-8981
a_mehrabian@sbu.ac.ir, https://orcid.org/0000-0001-6633-3092
d_mtehrani@sbu.ac.ir, https://orcid.org/0000-0003-3589-7324
Farzaneh.khajoei@yahoo.com, https://orcid.org/0000-0002-2325-9555

[2] Department of Biodiversity and Ecosystem Management, Research Institute of Environmental Sciences, Shahid Beheshti University, Tehran, Iran, Email: farid.salmanpour@yahoo.com, https://orcid.org/0000-0002-1517-9061

* Corresponding author: E-mail: a_mehrabian@sbu.ac.ir

Abstract

Melissopalynological analysis and chemical composition of honey provid[illegible] with useful and valuable information about the botanical and geographical origin of honey. The data in question is very important for authentication as well as for testing the quality of honey, so this is considered the main method in honey regulation here, we have used chemical analysis and melissopalynology to evaluate different honey samples from two main Iranian hubs of honey. Sampling was carried out on two important poles in Iran's honey production, the central Alborz region, and the mountainous Zagros ecosystems in the years 2020 to 2021. Therefore, 52 samples from Alborz (Northern Iran), as well as 42 samples from the Zagrosian ecosystems (western Iran) belonging to different ecological habitats, were collected. In addition, samples were taken at 7 altitudes from 0 to 3500 m a.s.l. Furthermore, in this study, various chemical analyses such as the effect of antioxidant activity, the amount of total phenolic content, pH, and moisture content of honey samples were evaluated. Our results showed that all honey samples were classified as polyfloral honey. Based on our findings, 57 honey samples (61%) contained the standard amount of pollen. A total of 42 plant families and 55 genera were identified in the studied samples, with the highest presence of Asteraceae, Fabaceae, Rosaceae, Apocynaceae, and Apiaceae. Finally, an antioxidant activity ratio of 19% to 98%, total phenolic content from 0.08 to 0.51 ppm, pH from 1.90 to 5.21, and moisture content from 13% to 18.40%.

Introduction

Honey is a precious natural product that is processed by bees from the nectar of flowers and stored in the beehives. Honey production in an area depends mainly on the vegetation and climate of the region, as the flowering season and nectar production can be different for a species in different regions [94]. With the growth of the worldwide honey market, the identification of the botanical origin as well as its authenticity has received much attention in the international society [47]. Counterfeit honey and poor-quality honey are major problems in the global honey trade. Honey, which consists of many stable elements as well as pollen grains, is collected by bees along with nectar. Melissopalynology as an effective method, as well as key criteria, has been widely used to determine the purity, geographical, and botanical origin of natural honey [63, 6]. Louveaux 1970, 1978 was the first to establish this method [59, 60]. However, several laboratory procedures have been developed to study of pollen grains in honey latter [19]. Melissopalynology studies evaluated pollen grains in honey [43, 60, 25, [illegible] Pollen analysis provides valuable data on the botanical and geographical origins of honey and is a key method for honey authentication [86]. The botanical and geographical origins of honey is important to the consumers and influences the product quality and physical, chemical, organoleptic, and bioactive properties of honey, which is considered as a valuable indicator of honey quality and authenticity [5, 12, 17, 57]. Furthermore, the quality of honey is largely dependent_on the nectariferous plant species that honey bees use in their diet. Accordingly, different melliferous plant species have different characteristics and applications (eg, drugs, nutrients, etc.) [73, 90].

Iran was ranked 9th in the world for honey production from 1993 to 2018 [35]. High climate variability, topographic complexity [87, 95], the geographical isolation of the highlands of Iran [71] as well as soil diversity [29] established different phytogeographical units in Iran [89, 93],

making it the center of a endemism for plant taxa in Southwest Asia [55, 41], the center of endemism of Irano-Turanian region [89, 53, 54], and an important center of plant diversity in the world [10, 11, 27, 52]. These ecological conditions have led to the formation of valuable beekeeping environments in the country, making honey production in Iran very diverse.

The Alborz and Zagros Mountains in Iran have created transition zones that have lead to a high diversity and endemism in this region [55, 64]. Influenced by the high-altitude diversity in this area, a variety of vegetation has been formed [36, 39], creating suitable habitats for beekeeping and honey production [69, 79]. Today, several researchers in Iran have used melissopalynology research methods, including melissopalynology study of some honey in Tehran Province [69] melissopalynology in Karaj, northern Iran [83], melissopalynology of some honey from Khorasan, N.E Iran [65], evaluation of honey pollen grains in Isfahan Province, C. Iran [33], the melissopalynology of honey in some areas of Mazandaran Province [79], melissopalynology of honey in Arasbaran, northwestern Iran [80], and origin evaluation of some Iranian honeys [51].

In addition to nutrients, honey also contains bioactive substances that can have a positive effect on the human body [77]. The healthful properties of honey are due to the presence of total phenolic content, including flavonoids, phenolic acids and their esters, as well as organic acids, free amino acids, vitamins, carotenoid derivatives, enzymes and biological elements [49]. These compounds have antioxidant activity properties [23]. Oxidation processes can be dealt with using antioxidant activity agents. Antioxidant activities can prevent many diseases or even reduce their symptoms [23]. Honey can be a complementary source of antioxidant activities in a balanced diet [7]. The antioxidant activity of honey is due to the presence of total phenolic content [3,2]. The moisture content of bee honey is very important for its stability against fermentation and

granulation. Low humidity protects honey from microbial activity and thus can be stored longer [20, 21].

The aim of the study was to evaluate different honey samples from different ecological regions of Iran based on chemical and melissopalynological analyses. In addition, the study tries to analyze the mentioned data in relation to geographical factors. Therefore, conducting a comparative study in different regions while comparing honey produced analyzes the relationship between pollen factors and some important chemical factors of honey with ecological factors.

Materials and methods

Study area

Iran is a prominent area of the Iranian Plateau in southwestern Asia situated between 24° to 40° N latitude and 44° to 64° E longitude, as well as a limited area of the orogenic belt (Zagros, Alborz, and other mountain chains. It covers the Asian block along with Arabian-African unity [14]. The main geomorphological areas of Iran, include Alborz, Zagros, Kopet Dagh, as well as several interior mountain ranges.

The Zagros Mountains extend from the northwest to the southeast. The Alborz Mountains are located at 36° N latitude from the Caspian Sea with an elevation of 5671 meters [70]. In addition to plant species, Alborz high forests (Hyrkanian forests) host valuable animal species such as Iranian leopards [34]. Most of our honey samples were collected from the areas of Alborz and Zagros, which have the highest honey production in Iran, including more than half of Iran's provinces, and most of the collected samples were close to forests and protected areas (Fig 1).

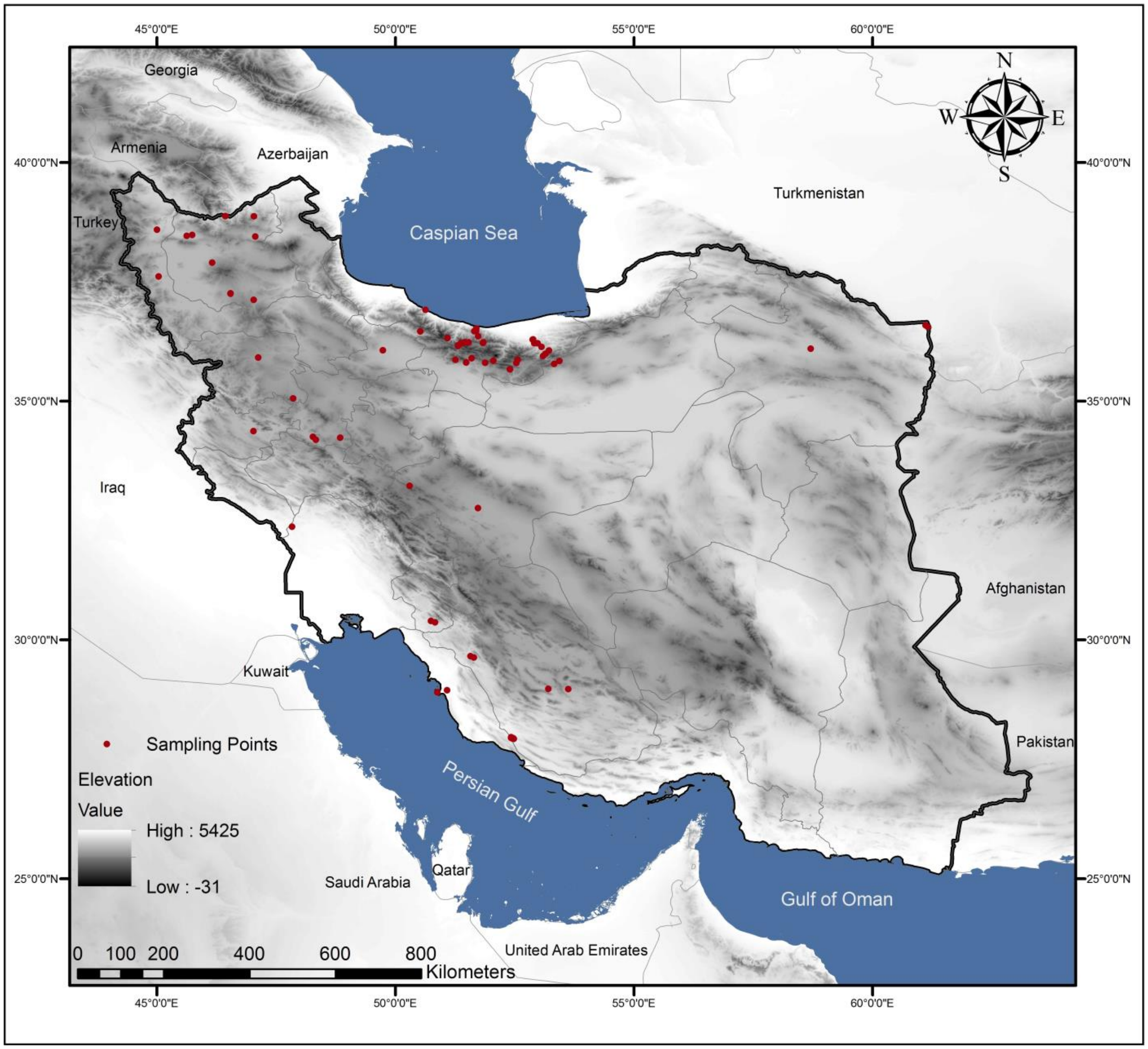

Fig 1. Study area and sample collection po[illegible]

Sample collection

This study is based on a direct collection of honey samples from beehive[illegible] the region from May to October 2020-2021. We selected sampling sites covering different geographical and botanical areas [4]. Using a data collection form, the geographical coordinates of the hives were recorded by a Garmin Map 64s GPS device, height, the type of honey and the claims of

beekeepers. Based on the diversity of ecological conditions, bee colonies were identified and sampled from the hive. From each colony, 1 sample of 300 g of unrefined and non-cla[illegible] honey was prepared for pollen extraction and chemical analysis such as pH, antioxidant activity, total phenolic content, and moisture content. [illegible]ples were placed in a sterile sealed container and kept in a cool place until they reached the laboratory [75].

Laboratory studi[illegible]

Laboratory studies were performed in two parts:

Melissopalynology based on scanning electron microscopy (SEM)

Melissopalynology analyzes based on the method of Louveaux et al. 1978 has been do[illegible] [59]. The melissopalynological method was proposed by the International Commission on Bee Botany (ICBB) [59, 91]. First, 10 grams of honey in about 20 mL of distilled water was dissolved on a hot plate (model HP100 made by Mtops company in South Korea) at a temperature below 40°C. After this step[illegible] honey wax samples were passed through a cloth strainer to remove large honey wax particles , then the liquid was poured into 30 mL conical centrifuge tubes. After several centrifugation times in the laboratory (2500 to 3000 g, Centrifuge model TDL-4 made by Jenius China) at room temperature (21 to 25°C), pollen grains were extracted from the honey. In the first round, the samples were centrifuged for 10 min (approximately 2500 g) and the supernatant was carefully removed to avoid pollen loss [75]. The remaining precipitate was mixed again with about 10 mL of distilled water, but this time the samples were centrifuged for 5 mi[illegible] our study, we repeated this process 4 - 7 times depending on the amount of sugar in the sample to completely remove the sugar in the honey . But after the last centrifugation, we poured the last remaining liquid into the watch glass and placed it on the hot plate so that the water

evaporated completely and the pollen grains remained at the bottom of the container. During this process, extreme care should be taken to avoid any contamination from external pollen from previous honey products (disposable equipment is recommended whenever possible) or pollen grains in the air (close windows and limit exposure).

We placed the prepared pollen grains on special bases and, after coating the samples with gold metal, we placed them inside the housing of a Japanese-made HITACHI SU3500 scanning electron microscope (SEM). Depending on the size of the sample, using this microscope, it is possible to shoot surfaces with magnification from 10 to 500,000 times and resolutions below 1 to 20 nanometers. By analyzing the information obtained by[illegible]M, we identified and counted the pollen grains, and then obtained the botanical origin of the honey.

Identification and counting

Using scanning electron microscopy images[illegible]llen grains obtained from honey sediments were counted, Counting 500 to 1000 pollen grains are necessary to determine relative abundance [13]. We also used pollen atlases and websites for pollen identificatio[illegible]he identification of pollen grains was done according to the morphology of the pollen grains. Pollen species should only be referred to as botanical species or species if they can be reliably identified at the genus or species level, which is rare [91].

Laboratory studies and chemical analysis of honey

All the reagents for biochemical studies were obtained from Merck Chemical Company in Germany. 2,2-Diphenyl-1-picrylhydrazyl (DPPH) was obtained from Sigma American Chemical Company.

In this part of the study, several chemical analyses of honey such as antioxidant activity, total phenolic content, pH, and moisture content were investigated. For all chemical analyses, honey was diluted 1/1 (w/v) with distilled water.

Analysis of antioxidant activity by DPPH free radical scavenging activity

~~At this stage,~~ the DPPH method was used to analyze all 94 honey samples [22].We dissolved a concentration of 0.1 mM of DPPH in methanol, and a purple solution was obtained. Then, 100 µl of DPPH was mixed with 10 µl of honey diluted and was incubated for 30 min in the da[illegible] Absorption was recorded at 520 nm using a JENUS V-1100 spectrophotometer manufactured by DLAB Chin[illegible] Ascorbic acid (20 mg/ml) was used as a positive control. The antioxidant activity was calculated from the following formula.

$$DPPH\ scavenging\ activity\ (\%) = A_{control} - A_{sample} / A_{control} \times 100$$

Analysis of total phenolic content

In this experiment, 1% ferric chloride solution (1% $FeCl_3$) was used to measure the amount of total phenolic content of honey [9], phenol at a concentration of 0.09% (mg /kg) was used as the standard sample. 0.1 ml of $FeCl_3$ was mixed with 0.1 ml of diluted honey and the absorbance was recorded at 590 nm. Phenol solution (0.09%) was used as the standard

Measuring pH and the moisture of honey

To determine the pH, 1 g of honey was mixed with 1 ml of distilled water, and the pH of each sample was measured with a pH meter model JENWAY 3505 and the moisture content of pure

honey was determined with a refractomete[illegible]Using the Minitab software, we performed a regression between the mean annual relative humidity of the honey collection sites and the moisture content of the honey samples using the fitted line plots statistical model.

Data analysis

Principal component analysis (PC[illegible]

A principal component analysis (PCA) was performed to identify the main chemical factors to distinguish the honey samples studied. Chemical parameters (pH), chemical compounds (total phenolic content and antioxidant activity), as well as moisture content, were used for PCA analysis. Finally, data were analyzed using the PAST software version 3.21 [40].

Cluster analysis

We used clustering to determine the similarity of the honey samples studied based on the composition of the plant families contained within them. A variance-covariance matrix was generated for all the honey samples and their botanical families. The studied samples were clustered using the Euclidean distance and UPGMA (unweighted pair group method using arithmetic average) using the PAST software.

Results and Discussion

Pollen analysis

In this study, after analyzing pollen grains of 94 honey samples, a total of 42 plant families were identified. Among them, Asteraceae (38%), Fabaceae (13.5%), Rosaceae (6%), and

Apocynaceae and Apiaceae (5%) were the largest plant families (Fig 2). Fig 3 illustrated some pollen images of the Asteraceae, Fabaceae, Rosaceae, and Apocynaceae families (F[illegible]A, B, C, D). The mentioned results show strong similarity with the following studies: Karimi in Fars Province [46] and Razzaghy in Mazandaran Province [79]. However, Asteraceae, Fabaceae, Rosaceae, Apiaceae, Brassicaceae, Papilionaceae, and Lamiaceae, respectively, showed the highest presence, in the research of Rašić [78]. Furthermore, in studies by Puusepp and Koff in Estonia [76], plant species of the Asteraceae, Fabaceae, and Rosaceae families showed the strongest presence. In addition, 55 plant genera were identified (Table 1).

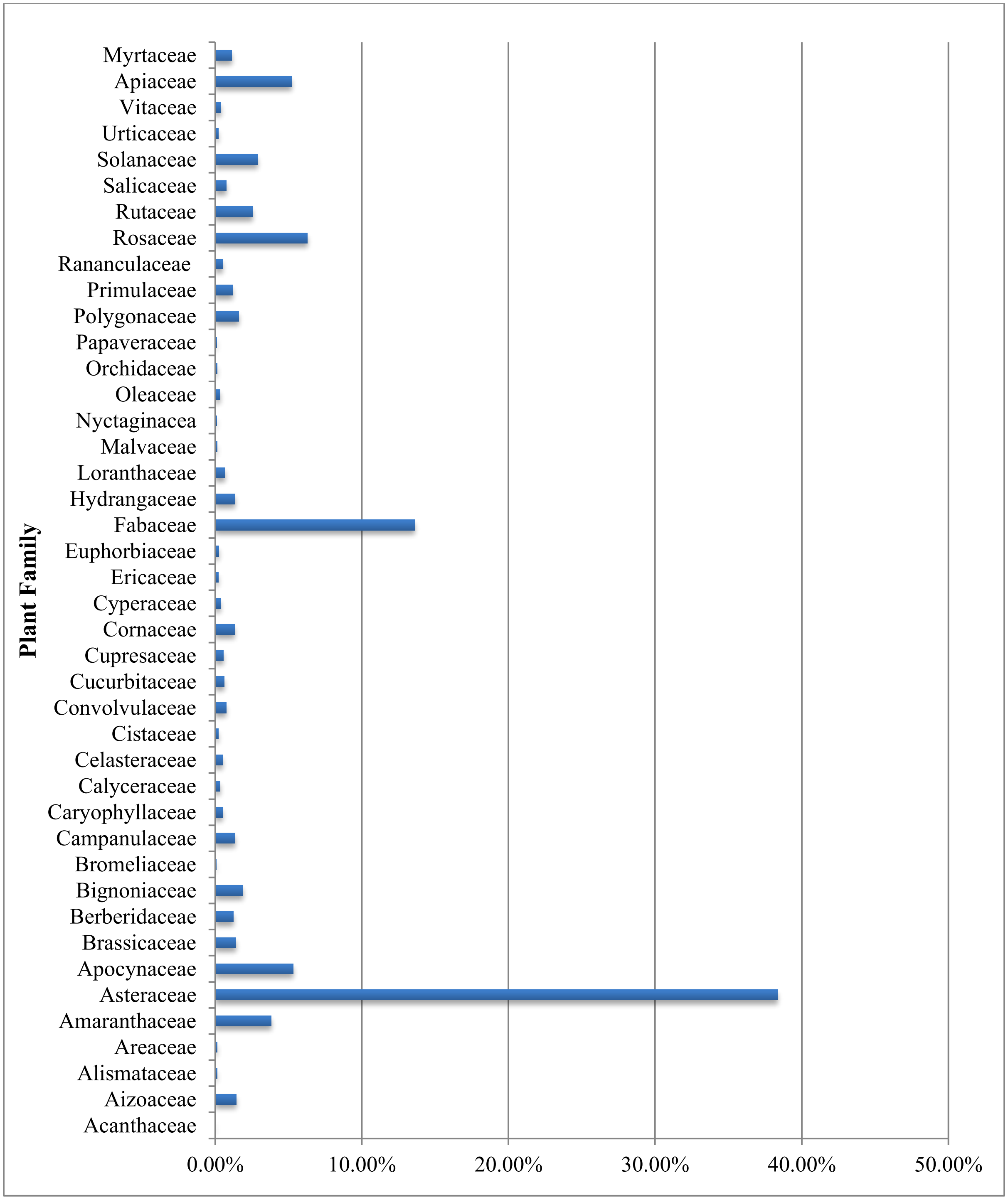

Fig 2. Percentage of presence of each plant family that was identified through analysis of pollen of 94 honey samples

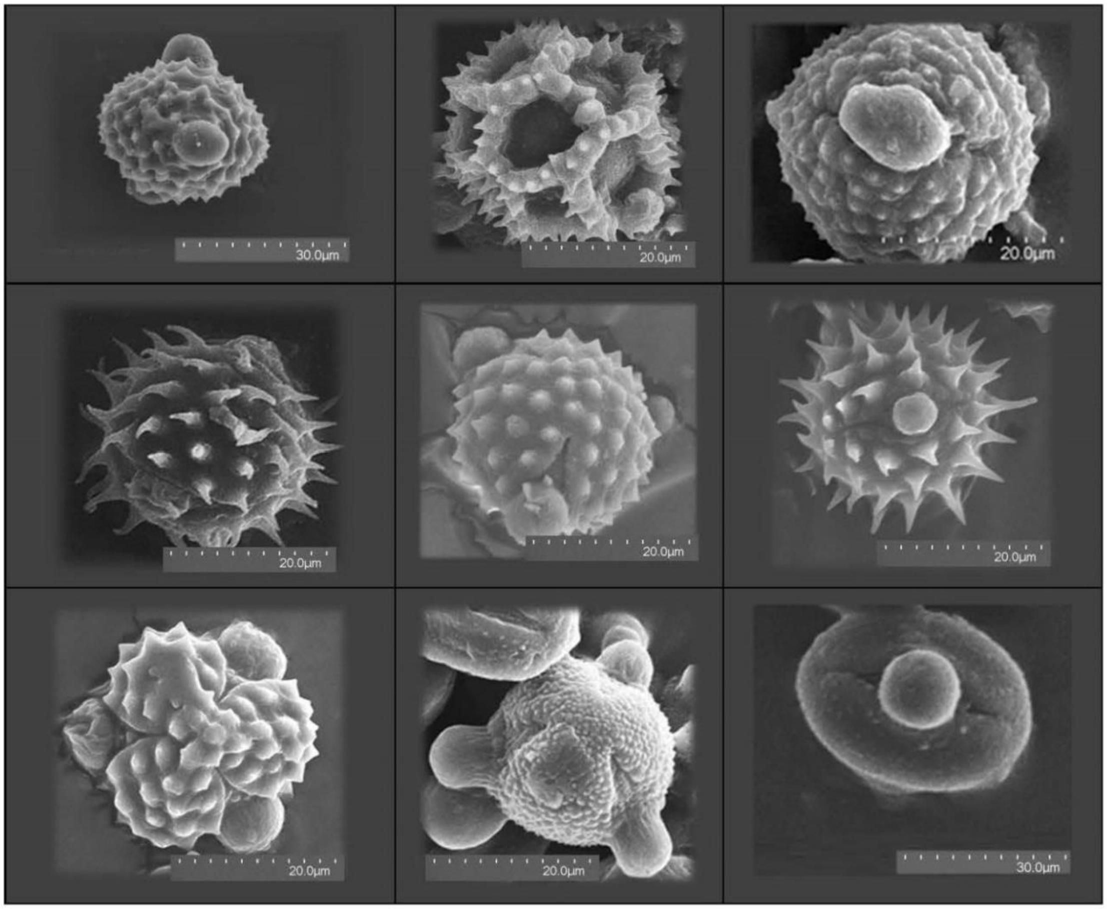

Fig 3A. Some pollen images of the Asteraceae family

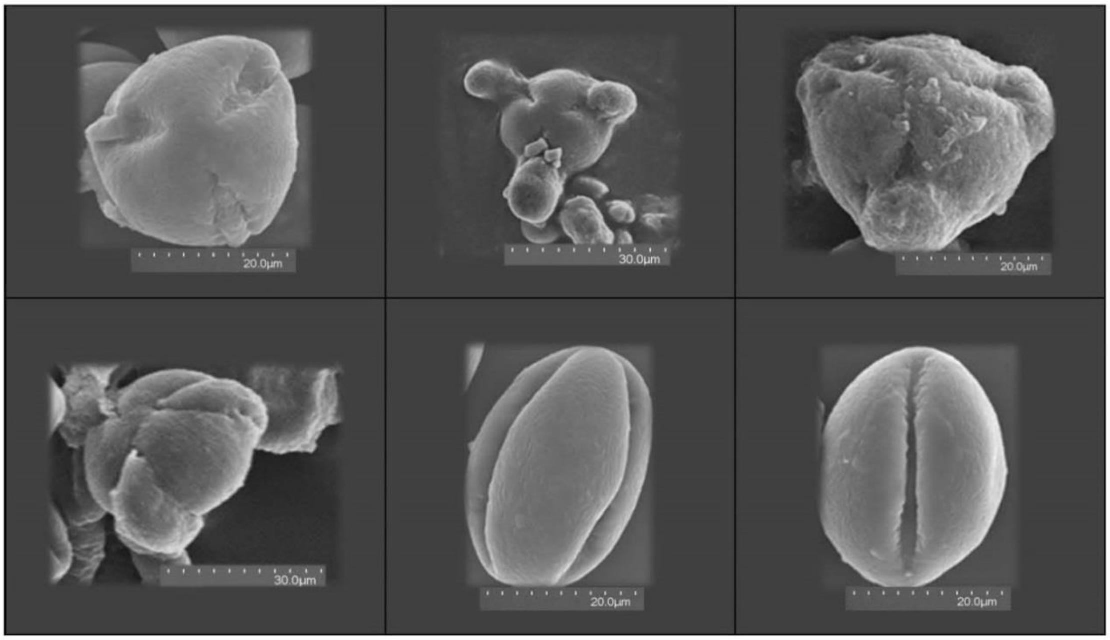

Fig 3B. Some pollen images of the Rosaceae family

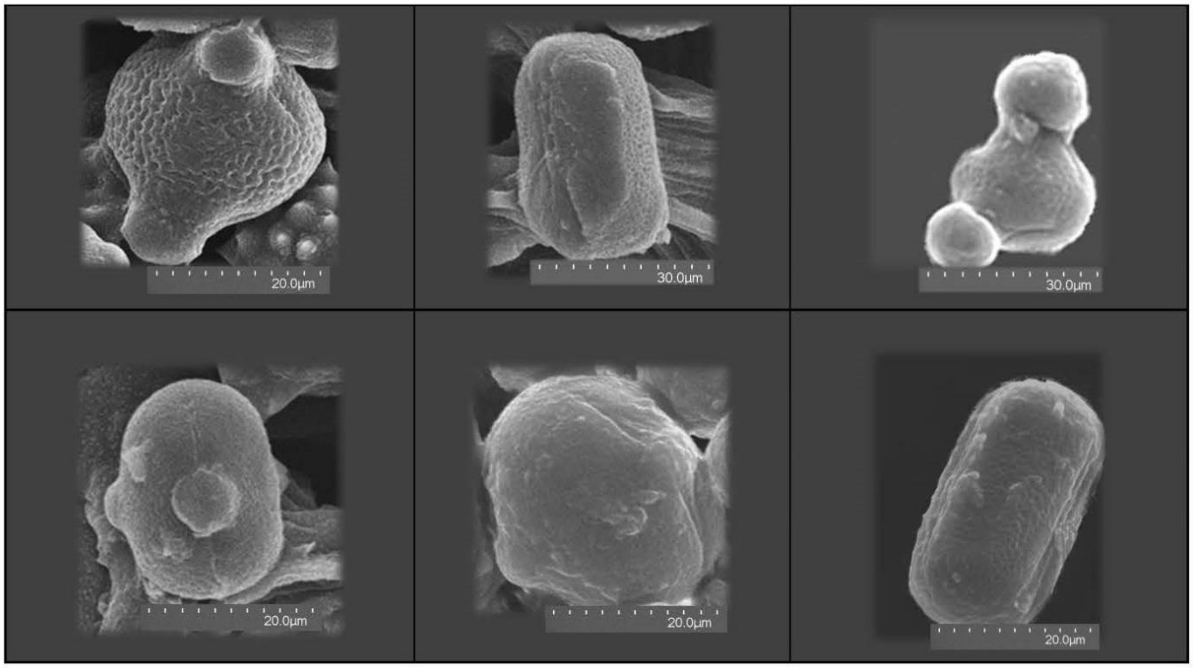

Fig 3C. Some pollen images of the Fabaceae family

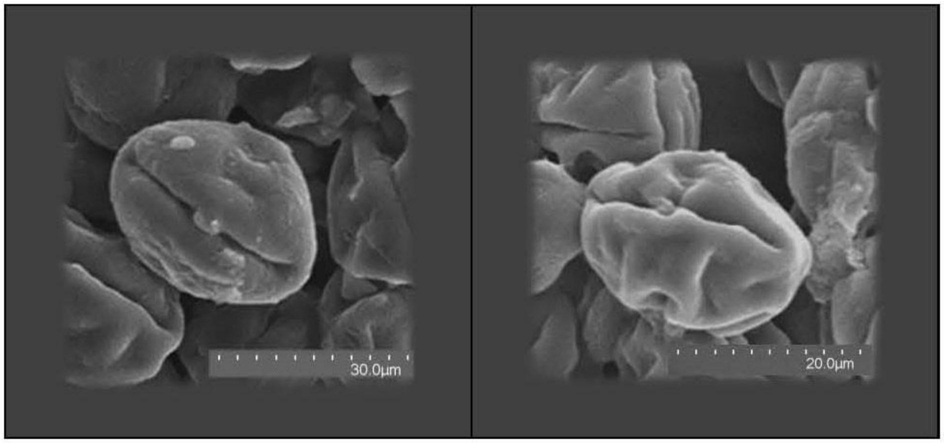

Fig 3D. some pollen images of the Apocynaceae family

Table 1. Pollen types of 94 honey samples in this study

Family	Pollen type
**Amaranthaceae**	*Amaranthus* sp.
	Atriplex type
**Apocynaceae**	*Catharanthus* type
**Apiaceae**	*Apiaceae* type
	Apiaceae type
	Torilis sp.
Acanthaceae	*Acanthaceae* type
	Achillea sp.
	Artemisia sp.
	Carduus type
	Carthamus type
**Asteraceae**	*Centaurea* type
	Cirsium arvense
	Crepis sp.
	Doronicum sp.
	Helichrysum sp.
	Hieracium sp.
	Inula sp.
	Senecio sp.
**Bignoniaceae**	*Bignoniaceae* type 1

	Bignoniaceae type 2
**Brassicaceae**	*Brassicaceae* type
**Caryophyllaceae**	*Caryophyllaceae* type
**Cistaceae**	*Cistus* sp.
**Convolvulaceae**	*Convolvulus arvensis*
**Cornaceae**	*Cornus sanguinea*
**Cucurbitaceae**	*Cucurbita* sp.
**Cyperaceae**	*Carex* sp.
**Fabaceae**	*Astragalus* type<br>*Robinia pseudacacia*<br>*Hedysarum* sp.<br>*Onobrychis cornuta*<br>*Vicia* sp.
**Hydrangeaceae**	*Philadelphus* sp.
**Myrtaceae**	*Eucalyptus calmadonensis*
**Oleaceae**	*Olea europaea*
**Polygonaceae**	*Fagopyrum* sp.<br>*Polygonum* sp.<br>*Rumex* sp.
**Ranunculaceae**	*Adonis* sp.<br>*Clematis* sp.<br>*Ranunculus* sp.
**Rosaceae**	*Alchemilla* sp.<br>*Cotoneaster* sp.<br>*Sorbaria* sp.<br>*Prunus domestica*<br>*Rosa cania*<br>*Sorbaria* sp.<br>*Spiraea* sp.
**Rutaceae**	*Rutaceae* type<br>*Poncirus* sp.

**Salicaceae**	*Populus nigra*
**Solanaceae**	*Solanum* spp.
**Urticaceae**	*Parietaria judaica*<br>*Urtica dioica*

Our results showed that all the studied samples are classified as polyfloral honey and based on the palynological criteria, about 30% of the studied honey samples were not of good quality, the rest good quality.

Pollen grains extracted from honey samples were identified based on pollen references. A quantitative assessment of pollen grains in each honey sample was performed according to the Louveaux standard. Samples were collected at altitudes ranging from 12 to 3062 meters above sea level. Samples were taken at seven altitudes, considering the variation of vegetation type and the location of beekeepers depending on the season. More than 73% of the samples were taken at altitudes between 1000 and 3000 meters above sea level (Fig 4).

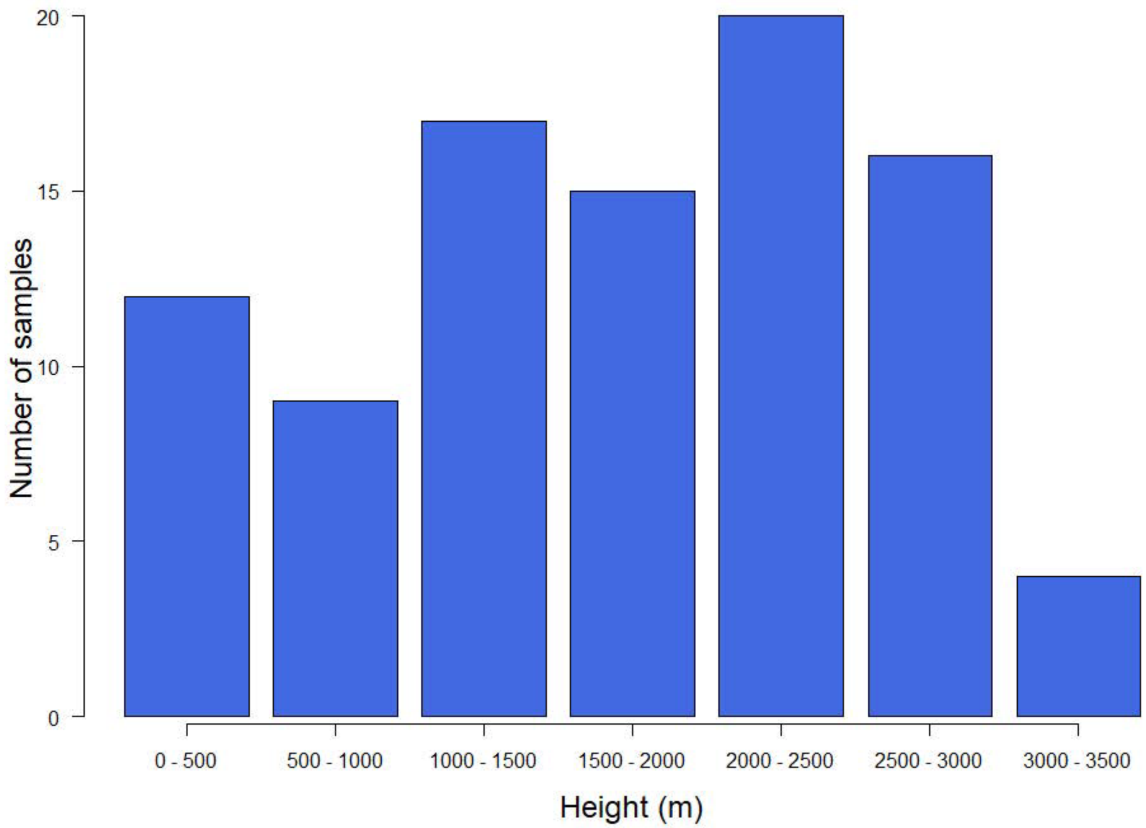

Fig 4. Number of samplings performed at different altitude intervals

Physicochemical analysis of honeys

According to the results of the chemical analysis of honey samples, the amount of antioxidant activities in the samples was as low as 19% and the highest as 98%. According to Fig 5, more than 36% of the total samples had antioxidant activities between 40% and 60%.

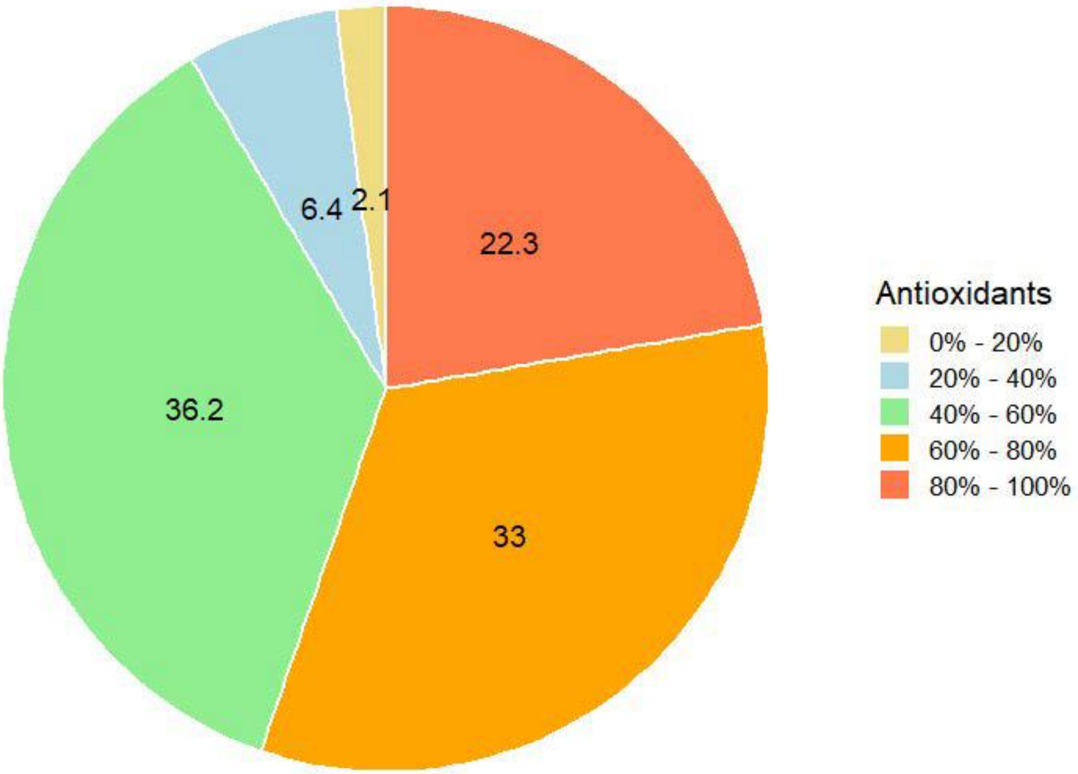

Fig5. The antioxidant activity abundance ratio of honey samples

The chemical, physical, antioxidant and health-promoting properties of honey vary depending on the flower source, soil type, geological root, and climatic conditions CACS (2001). Ancient and modern civilizations used honey as medicine for various ailments [16]. It is also a delicious food composed mainly of various sugars and compounds such as proteins, organic acids, amino acids, nutrients, minerals, carotenes and aromatic substances. It is rich in flavonoids and phenolic acids, exhibits many biological effects and acts as natural antioxidant activity [45]. The results of our chemical analysis showed that the chemical composition of honey samples collected from different ecological regions was different from each other [58]. Chemical experiments also showed that about 3/5 of the samples showed more than 60% of the effect of antioxidant activity.

We compared the results of antioxidant activities with increasing elevation, which seems to have a positive response to increasing height, and up to 3000 meters the trendline showed an upward trend, but after 3000 meters, the antioxidant activities followed the exact opposite trend. The highest efficiency of antioxidant activity was observed at altitudes between 2500 and 3000 meters, with an average of 88.71% (Figure 6). The potential variation of the antioxidant activity of honey depends on topographical and botanical differences [62, 92]. The antioxidant activity of honey is mainly due to phenolic and flavonoid compounds. Thus, a significant difference in antioxidant activity is observed between different types of honey worldwide [15, 1, 69].

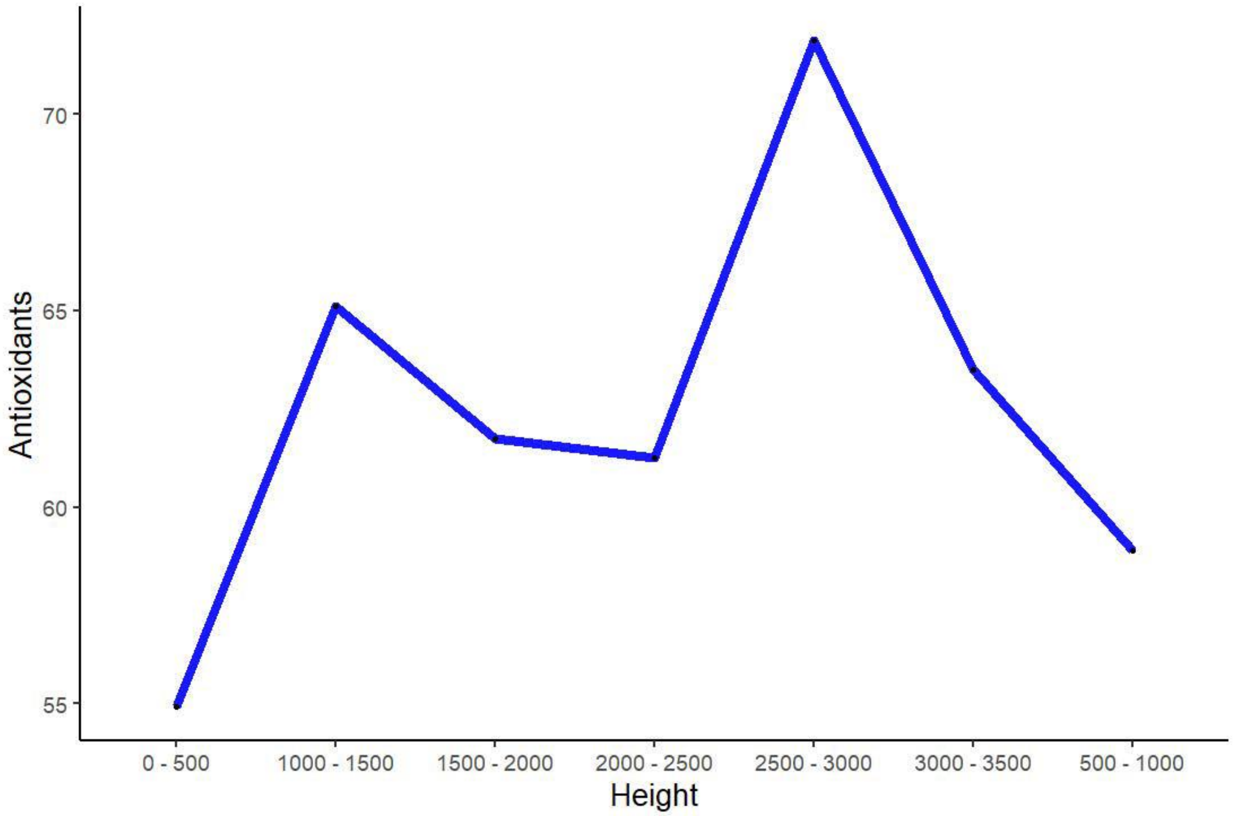

Fig 6. Comparison of antioxidant activities with altitude

The total phenolic content in the honey samples was between 0.08 and 0.59 micrograms. Differences in total phenolic content in honey samples may be due to different geographical

origins or other factors such as collection season and storage conditions. An important group of compounds affecting the appearance and functional properties of honey is phenolic acid, which has nutritional value and medicinal properties [8]. The type and concentration of total phenolic content in different kinds of honey are different from each other [84]. The amount of total phenolic content is influenced by various factors such as plant type, geographical origin, and climatic conditions of the honey production site [32]. Determination of total phenolic content in honey is a suitable factor to evaluate the quality and therapeutic potential of honey [72].

Also in this section, the pH of each honey sample was tested (Fig. 7). The lowest pH was 1.9 and the highest pH was 5.2, and 48% of the total samples were in the pH range from 2.5 to 3.5. The global standard for the pH of honey is 3.9 (3.2 to 4.5). The pH values between 3.4 and 6.1 indicate the freshness of honey samples [50, 85]. Honey is recognized as a therapeutic option for the treatment of acute and chronic wounds, and a variety of in vitro and in vivo research studies have been performed in this area [88, 24, 44]. The low pH of honey (0.4 to 5.3) promotes wound healing [31, 67] which is accomplished by reducing the highly alkaline environment in non-healing wounds to a more acidic healing environment.

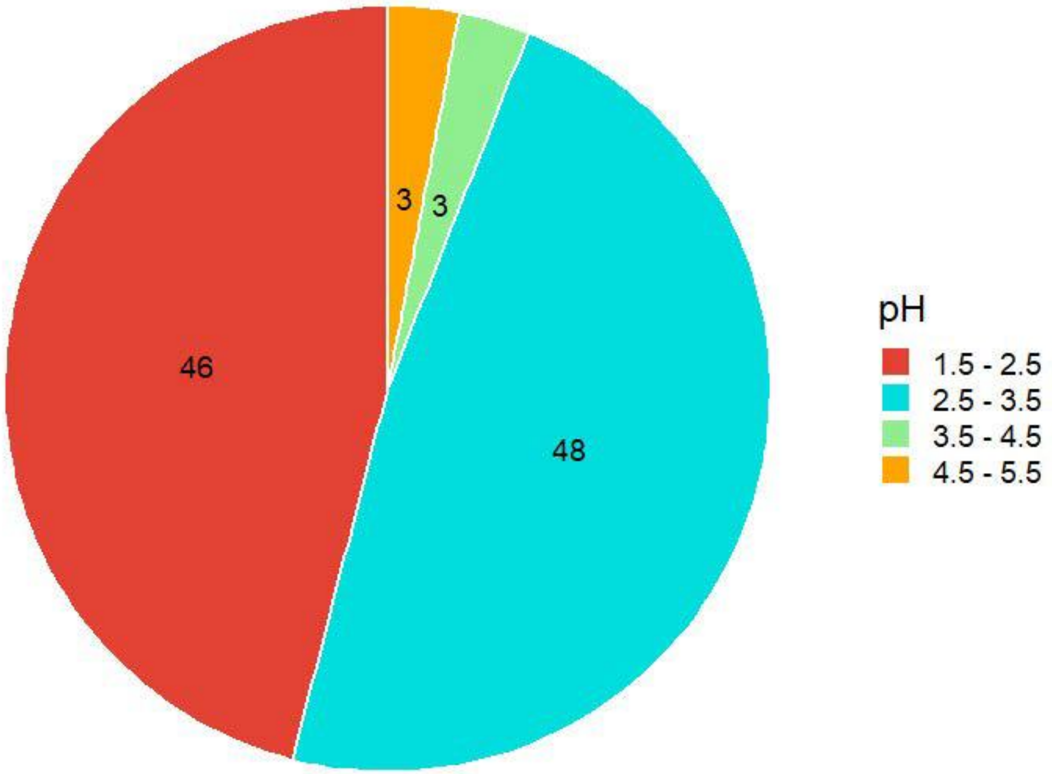

Fig 7. pH abundance ratio of the samples

In this study, all honey samples showed a low moisture content (13% to 18.80%), which corresponded to the values (< 20 <) set by the Codex standard for honey (Almentarius Codex, 1987). Moisture content is considered as an important criterion to evaluate the quality of honey. The less moisture in honey, the longer the shelf life [38]. The moisture content of honey depends on various factors such as the time of harvest, the climatic factors of the geographical area, and the storage temperature [68, 37]. The parameter of quality and uniqueness of honey has been studied by many researchers, and the quality and reliability of honey is very important to both buyers and producers.

Regression results between the average annual relative humidity of the area (independent variable) and the humidity of the honey sample (dependent variable) showed that there is a significant relationship between them (p-value 0.000), so the moisture content of the honey samples is a function of the environmental humidity (Fig 8).

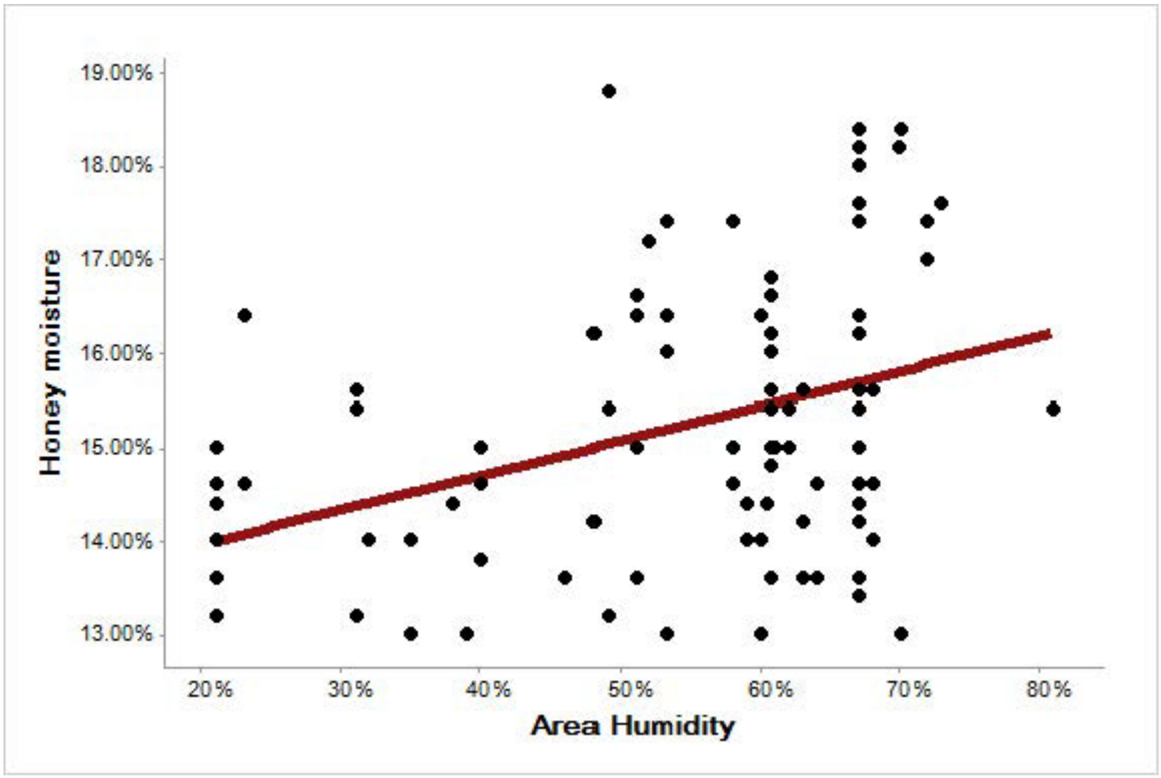

Fig 8. Regression related to the effect of environmental humidity on the moisture of honey samples

Principal component analysis (PCA)

For the PCA analysis, both the first and second axes accounted for 57.19% of the total variance (Table 2). The first principal component explained 29.8% of the variance, the main chemical variables of which were: total phenol content and pH (Table 2). The second axis explained 27. 39% of the variance and antioxidant activities and pH were the most significant variables (Table 2).

Table 2. Relative eigenvalue, percentage of variance, and vector values of principal component analysis (PCA) using total phenolic content, antioxidant activity, pH, and moisture content of the studied honey samples.

Name of variables	PC 1	PC 2	PC 3	PC 4
Eigenvalue	1.19215	1.09555	0.932069	0.780231
% variance	29.804	27.389	23.302	19.506
Antioxidant activities	-0.15449	**0.79953**	-0.2348	-0.53081
Total phenol content	**0.59267**	0.056052	0.69854	-0.39705
pH	**0.58965**	**0.47616**	-0.1825	0.62632
Moisture content	-0.52648	0.36179	0.65085	0.41026

The PCA did not result in any significant differences between all honey samples studied (Fig. 9). Considering that the honey samples studied were collected from different geographical areas, samples from the same area were not placed next to each other in the plot. This shows that honey samples from different locations in the same area have different chemical compositions.

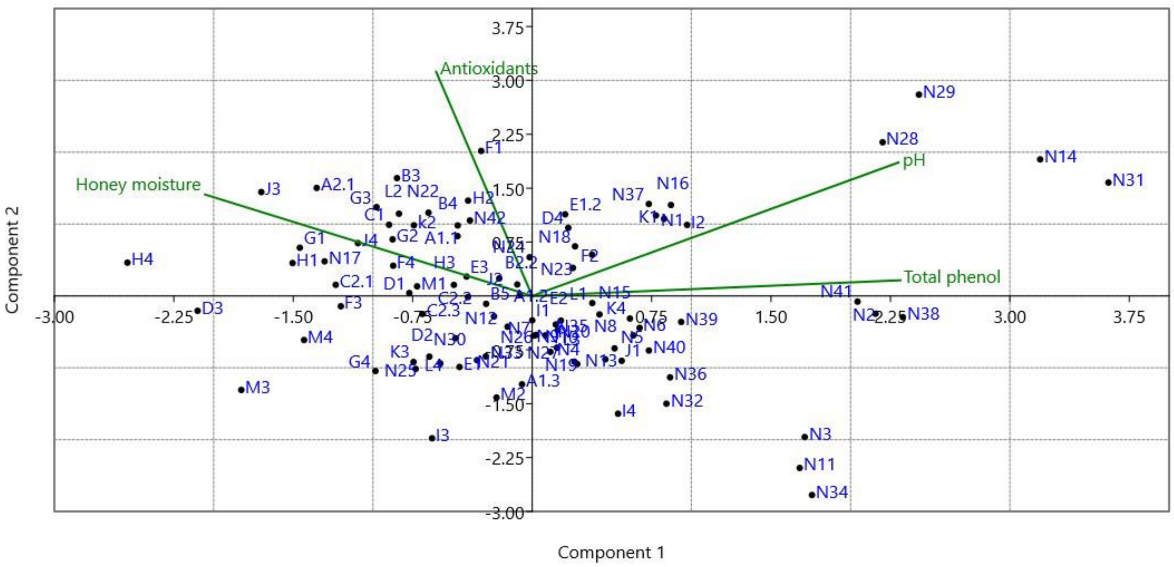

Fig 9. Principal component analysis (PCA) scatter plot of the studied honey samples based on total phenolic content, antioxidant activity, pH, and moisture content. Sample codes in Supplementary 1.

Cluster analysis

According to the cluster analysis of the plant family composition, the honey samples collected from a geographical location were located far from each other in the dendrogram and were not located in the same cluster, as shown in Fig 10.

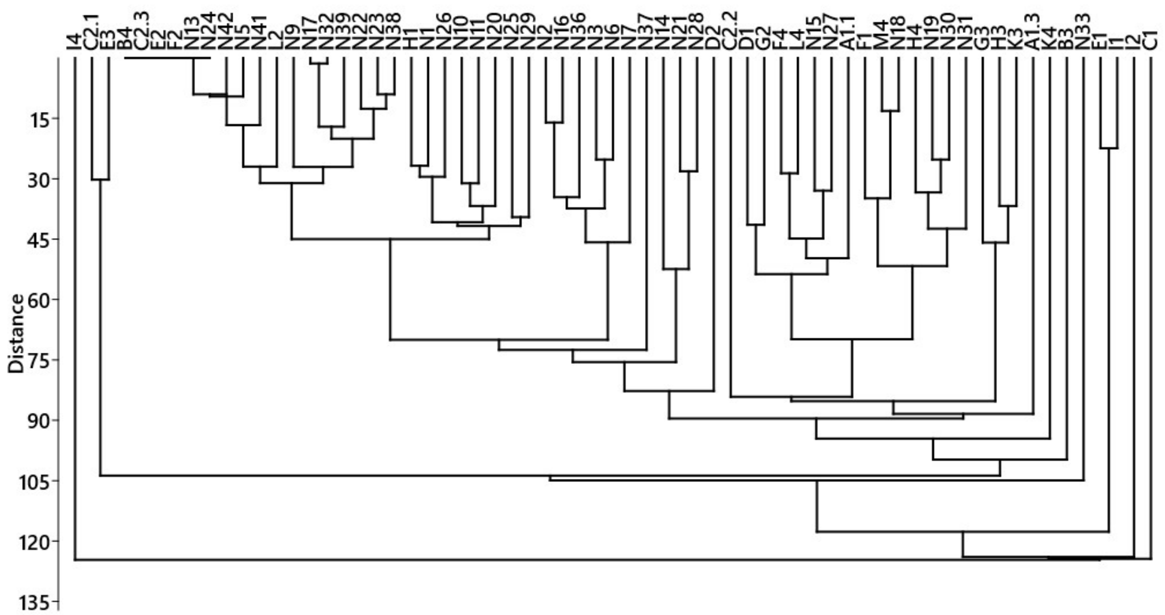

Fig 10. Dendrogram of the studied honey samples according to their botanical family composition and clustered by UPGMA (unweighted pair group method using Euclidean distance). Cophenetic correlation coefficient = 0.8565. Sample codes in Supplementary 1.

One of the important concerns of honey quality is to guarantee the authenticity of honey according to legal requirements. This authenticity is achieved by recognizing the plant's origin, geography, and chemical analysis [26, 61] Nutritional and healing properties of honey from the very beginning make it an important component of traditional medicine [18, 88]. The natural antioxidant activities in honey are valuable against heart disease, cancer, inflammation, coronary heart disease, aging, tumors, gastrointestinal tract, and wound healing [28, 48]. In relation to health, honey, bee pollen, propolis, royal jelly, beeswax, and bee venom have all been used in traditional and modern medicine [30, 56]. Researchers have identified the bioactive properties of honey, propolis, and royal jelly, indicating compounds with antimicrobial, anti-inflammatory, antioxidant activity, anti-tumor and anti-cancer activities [74, 56, 30] Honey is used to treat stomach ulcers and injuries, increase oral health, fight stomach disorders, and liver and pancreatic diseases, as well as to promote cardiovascular health [74, 30].

Conclusion

Melissopalynology is a useful and complementary tool along with chemical assays to measure the authenticity, fraud, and differentiation between different types of honey. In addition to being a useful tool in the food industry and therapeutic debates, melissopalynology is even more important in the conservation debate. Conducting more extensive studies in the field of melissopalynology in the future to identify the plant species used by bees is of mutual importance in the conservation debate, plant species conservation protects bees and bee conservation leads to plant species conservation. In the field of conservation, more and more ongoing research is needed to better understand the behavior and role of bees and plants. Bee populations are threatened by human activities. Climate change, invasive species, monocultures

that have less variety in flower resources, and the use of pesticides have a negative effect on bees. Considering that climate changes affect the feeding behavior of mammals, these changes can affect insects more [81]. On the other hand, seasonal changes (including changes in temperature, natural light, and precipitation) and changes in vegetation type can not only have a significant impact on the behavior of bees but also on the behavior of other animals [82]. It is recommended that future studies look at the impact of climate change on honey bees and on the pollen of honey produced by farmed bees be compared to honey produced by wild bees to help better understand the role of melissopalynology in the protection of bees and plant species and the protection of ecosystems.

Funding

This research received no external funding.

Acknowledgments

The authors thank anonymous reviewers for their valuable comments on an earlier draft. We are grateful to Mr. Saeed Javadi Anaghizi is thanked for his assistance in SEM work. We are profoundly grateful to Mr. Mehdi Kia Hairati, Mr. Mohsen Yasari, Mr. Erfan Rajabi, and Mr. Mohammad Reza Kashfi for their valuable help during the fieldwork study.

CRediT authorship contribution statement

Writing - Original Draft: Zahra Shakoori

Writing – review & editing: Zahra Shakoori, Ahmadreza Mehrabian, Dariush Minai, Farid Salmanpour, Farzaneh Khajoei Nasab

Conceptualization: Zahra Shakoori, Dariush Minai, Ahmadreza Mehrabian, Farid Salmanpour, Farzaneh Khajoei Nasab

Principal Investigation: Zahra Shakoori

Formal analysis: Zahra Shakoori, Dariush Minai, Ahmadreza Mehrabian, Farid Salmanpour

Sample collection: Zahra Shakoori, Farid Salmanpour

Software: Zahra Shakoori, Farid Salmanpour, Farzaneh Khajoei Nasab

Supervision: Ahmadreza Mehrabian

Methodology: Zahra Shakoori, Dariush Minai, Ahmadreza Mehrabian, Farid Salmanpour

Data curation: Zahra Shakoori

Conflicts of Interest

The authors confirm that they have no conflicts of interest with respect to the work described in this manuscript.

DATA AVAILABILITY STATEMENT

DOI: 10.6084/m9.figshare.22586863

https://figshare.com/s/949e02f3163f39991d4e

Statement

In the above study, the beekeepers provided us with honey samples voluntarily and with full consent, and we did not need to obtain a permit due to not entering protected areas or national parks.

References

1. Aljadi AM, Kamaruddin MY. Evaluation of the phenolic contents and antioxidant capacities of two Malaysian floral honeys. Food chemistry. 2004 May 1;85(4):513-8. https://doi.org/10.1016/S0308-8146(02)00596-4
2. Al Kiyumi EH, Al Rashdi BS, Al Alawi AR, Al Balushi AA, Al Hooti SN, Al Hosni SI, Dhanalekshmi UM, Khan SA. Quantification of bioactive components and evaluation of antioxidative potential of different floral origin honey from arid regions of Oman. Biocatalysis and Agricultural Biotechnology. 2021 May 1;33:102007.
3. Al-Mamary M, Al-Meeri A, Al-Habori M. Antioxidant activities and total phenolics of different types of honey. Nutrition research. 2002 Sep 1;22(9):1041-7. https://doi.org/10.1016/S0271-5317(02)00406-2
4. Al Naggar Y, El-Sofany A, Naiem E, Seif A. Characterization of *Apis mellifera* honey of different botanical and geographical origins in Egypt. The Egyptian Journal of Experimental Biology (Zoology). 2018;14(1):75-84. http://dx.doi.org/10.5455/egysebz.20180523104927
5. Alnaqdy A, Al-Jabri A, Al Mahrooqi Z, Nzeako B, Nsanze H. Inhibition effect of honey on the adherence of Salmonella to intestinal epithelial cells in vitro. International journal of food microbiology. 2005 Sep 15;103(3):347-51. https://doi.org/10.1016/j.ijfoodmicro.2004.11.042
6. Alvarez-Suarez JM, editor. Bee products-chemical and biological properties. Springer; 2017 Sep 5. https://doi.org/10.1007/978-3-319-59689-1
7. M Alvarez-Suarez J, Giampieri F, Battino M. Honey as a source of dietary antioxidants: structures, bioavailability and evidence of protective effects against human chronic diseases. Current medicinal chemistry. 2013 Feb 1;20(5):621-38. https://doi.org/10.2174/092986713804999358

8. Alvarez-Suarez JM, Tulipani S, Romandini S, Bertoli E, Battino M. Contribution of honey in nutrition and human health: a review. Mediterranean Journal of Nutrition and Metabolism. 2010 Jan 1;3(1):15-23. https://doi.org/10.1007/s12349-009-0051-6

9. Athipornchai A, Klangmanee K. A rapid determination of the effective antioxidant agents using their Fe (III) complexes. Arabian Journal of Chemistry. 2021 Mar 1;14(3):102986. https://doi.org/10.1016/j.arabjc.2021.102986

10. Barthlott W, Biedinger N, Braun G, Feig F, Kier G, Mutke J. Terminological and methodological aspects of the mapping and analysis of global biodiversity. Acta botanica fennica. 1999;162(0):103-10. https://www.researchgate.net/profile/Jens-Mutke/publication/215672839_Terminological_and_methodological_aspects_of_the_mapping_and_analysis_of_global_biodiversity/links/57c5338208aecd4514157c0b/Terminological-and-methodological-aspects-of-the-mapping-and-analysis-of-global-biodiversity.pdf

11. Barthlott W, Neinhuis C, Jetter R, Bourauel T, Riederer M. Waterlily, poppy, or sycamore: on the systematic position of Nelumbo. Flora. 1996 Apr 1;191(2):169-74. https://doi.org/10.1016/S0367-2530(17)30709-0

12. Basualdo C, Sgroy V, Finola MS, Marioli JM. Comparison of the antibacterial activity of honey from different provenance against bacteria usually isolated from skin wounds. Veterinary microbiology. 2007 Oct 6;124(3-4):375-81. https://doi.org/10.1016/j.vetmic.2007.04.039

13. Behm F, Von Der Ohe K, Henrich W. Zuverlässigkeit der pollenanalyse von honig: bestimmung der pollenhäufigkeit. Deutsche Lebensmittel-Rundschau. 1996;92(6):183-8. https://pascal-francis.inist.fr/vibad/index.php?action=getRecordDetail&idt=3123198

14. Berberian M, King GC. Towards a paleogeography and tectonic evolution of Iran: Reply. Canadian Journal of Earth Sciences. 1981 Nov 1;18(11):1764-6. https://doi.org/10.1139/e81-019

15. Beretta G, Granata P, Ferrero M, Orioli M, Facino RM. Standardization of antioxidant properties of honey by a combination of spectrophotometric/ fluorimetric assays and chemometrics. Analytica Chimica Acta. 2005 Mar 28;533(2):185-91. https://doi.org/10.1016/j.aca.2004.11.010

16. Berhanu S, Tadesse DM, Jorge A. Physico-chemical properties of Ethiopian *Apis mellifera* Honey: Review. J Agric Sc Food Technol. 2022;8(1):038-44. https://dx.doi.org/10.17352/2455-815X.000143

17. Bogdanov S, Jurendic T, Sieber R, Gallmann P. Honey for nutrition and health: a review. Journal of the American college of Nutrition. 2008 Dec 1;27(6):677-89. https://doi.org/10.1080/07315724.2008.10719745

18. Bogdanov S. Nature and origin of the antibacterial substances in honey. LWT-Food Science and Technology. 1997 Nov 1;30(7):748-53. https://doi.org/10.1006/fstl.1997.0259

19. Bryant Jr VM, Jones GD. The R-values of honey: pollen coefficients. Palynology. 2001 Jan 1;25(1):11-28. https://doi.org/10.1080/01916122.2001.9989554

20. Buba F, Gidado A, Shugaba A. Analysis of biochemical composition of honey samples from North-East Nigeria. Biochem Anal Biochem. 2013;2(3):139. DOI: 10.4172/2161-1009.1000139

21. Cantarelli MA, Pellerano RG, Marchevsky EJ, Camiña JM. Quality of honey from Argentina: Study of chemical composittion and trace elements. The Journal of Argentine Chemical Society. 2008 Dec;96(1-2):33-41. https://doi.org/10.5897/AJMR10.008

22. Chua LS, Rahaman NL, Adnan NA, Eddie Tan TT. Antioxidant activity of three honey samples in relation with their biochemical components. Journal of analytical methods in chemistry. 2013 Jan 1;2013. https://doi.org/10.1155/2013/313798

23. Cianciosi D, Forbes-Hernández TY, Afrin S, Gasparrini M, Reboredo-Rodriguez P, Manna PP, Zhang J, Bravo Lamas L, Martínez Flórez S, Agudo Toyos P, Quiles JL. Phenolic compounds in honey and their associated health benefits: A review. Molecules. 2018 Sep 11;23(9):2322. https://doi.org/10.3390/molecules23092322

24. Cooper RA, Molan PC, Harding KG. The sensitivity to honey of Gram-positive cocci of clinical significance isolated from wounds. Journal of Applied microbiology. 2002 Nov 1;93(5):857-63. https://doi.org/10.1046/j.1365-2672.2002.01761.x

25. Corvucci F, Nobili L, Melucci D, Grillenzoni FV. The discrimination of honey origin using melissopalynology and Raman spectroscopy techniques coupled with multivariate analysis. Food chemistry. 2015 Feb 15;169:297-304. https://doi.org/10.1016/j.foodchem.2014.07.122

26. da Silva PM, Gauche C, Gonzaga LV, Costa AC, Fett R. Honey: Chemical composition, stability and authenticity. Food chemistry. 2016 Apr 1;196:309-23. https://doi.org/10.1016/j.foodchem.2015.09.051

27. Davis SD, Heywood VH, Hamilton AC. Centres of plant diversity. Natural History. 1994;111(1):1-0. http://www.nativefishlab.net/library/textpdf/18340.pdf

28. de Rodríguez GO, de Ferrer BS, Ferrer A, Rodríguez B. Characterization of honey produced in Venezuela. Food Chemistry. 2004 Mar 1;84(4):499-502. https://doi.org/10.1016/S0308-8146(02)00517-4

29. Dewan ML, Famouri J. The soils of Iran. 1964. FAO, Rome.

30. Easton-Calabria A, Demary KC, Oner NJ. Beyond pollination: honey bees (Apis mellifera) as zootherapy keystone species. Frontiers in Ecology and Evolution. 2019 Feb 1;6:161. https://doi.org/10.3389/fevo.2018.00161

31. Efem SE. Clinical observations on the wound healing properties of honey. British journal of Surgery. 1988 Jul;75(7):679-81. https://doi.org/10.1002/bjs.1800750718

32. Escuredo O, Silva LR, Valentão P, Seijo MC, Andrade PB. Assessing Rubus honey value: Pollen and phenolic compounds content and antibacterial capacity. Food Chemistry. 2012 Feb 1;130(3):671-8.https://doi.org/10.1016/j.foodchem.2011.07.107

33. Faghihi AR, Ebadi R, Nazarian H. Study of pollen plants used by honey bees (Apis mellifera L.) in Khansar and Faridan regions of Isfahan province with sub stepic climate. Iranian Journal of Agricultural Sciences. 2004;35(2):265-83.https://eurekamag.com/research/004/024/004024250.php

34. Farhadinia, M.S., Soofi, M., Rosen, T., Moghadas, P., Hobeali, K., Behnoud, P., Amanov, A., Dieterich, T., Hojamuradov, H., Hudaikuliev, N., Karryeva, s., Kaczensky, P., Mengliev, s., Muhashov, A., Nurmuhambetov, Z., Pestov, M., Potaeva, A., Terentyev, V., Salmanpour, F., Tizrouyan, H., Alinezhad, H., Jafari, B., Ommatmohammadi, M., Rabie, K., Veyisov, A., Linnell, J., 2022. Status of Persian leopards in northern Iran and Central Asia. CATnews Special. https://www.researchgate.net/publication/362889852

35. FAOSTAT, 2018. Production of Natural Honey in World. http://www.fao.org/faostat/en/#data/QL/visualize

36. Fayaz, M. 2015. Vegetation types of Tehran. Research Institute of Forests and Rangelands.

37. Finola MS, Lasagno MC, Marioli JM. Microbiological and chemical characterization of honeys from central Argentina. Food chemistry. 2007 Jan 1;100(4):1649-53. https://doi.org/10.1016/j.foodchem.2005.12.046
38. Fredes C, Montenegro G. Heavy metal and other trace elements contents in honey bee in Chile. Cien. Inv. Agr.(in English) 33 (1): 50-58. International Journal of Agriculture and Natural Resources. 2006 Jan 1;33(1):50-8. https://revistaschilenas.uchile.cl/handle/2250/10.7764/rcia.v33i1.328
39. Ghelich Nia, H. 2017. Vegetation types of Mazandaran. Research Institute of Forests and Rangelands.

40. Hammer Ø, Harper DA, Ryan PD. PAST: Paleontological statistics software package for education and data analysis. Palaeontologia electronica. 2001 Jun 22;4(1):9. https://paleo.carleton.ca/2001_1/past/past.pdf
41. Hedge IC, Wendelbo P. Patterns of distribution and endemism in Iran. 1978. https://www.scopus.com/record/display.uri?eid=2-s2.0-0006003564&origin=inward&txGid=08c1677fbb653bcda02974e2f9bd74ed
42. Jones GD, Bryant Jr VM. New frontiers in Palynology. Palynology: Principles and Applications, edited by J. Jansonius and DC McGregor. 1996:933-38. https://scholar.google.com/scholar?hl=en&as_sdt=0%2C5&q=New+Frontiers+in+Palynology.+Palynology%3A+Principles+and+Applications%2C+edited+by+J.+Jansonius+and+DC+McGregor.&btnG=
43. Johnson DW, van Eps C, Mudge DW, Wiggins KJ, Armstrong K, Hawley CM, Campbell SB, Isbel NM, Nimmo GR, Gibbs H. Randomized, controlled trial of topical exit-site

application of honey (Medihoney) versus mupirocin for the prevention of catheter-associated infections in hemodialysis patients. Journal of the American Society of Nephrology. 2005 May 1;16(5):1456-62. https://doi.org/10.1681/ASN.2004110997

44. Karabagias IK, Badeka A, Kontakos S, Karabournioti S, Kontominas MG. Characterisation and classification of Greek pine honeys according to their geographical origin based on volatiles, physicochemical parameters and chemometrics. Food chemistry. 2014 Mar 1;146:548-57. https://doi.org/10.1016/j.foodchem.2013.09.105

45. Karimi A, Nazarian H, Jafari E, Hatami A. Identification and determine the distribution of plants used by bees in Galehdar watershed (Fars province). Natural Ecosystems of Iran. 2016 Nov 21;7(3):13-27. https://neijournal.nour.iau.ir/mobile/article_527674.html?lang=en

46. Kaškonienė V, Venskutonis PR. Floral markers in honey of various botanical and geographic origins: a review. Comprehensive reviews in food science and food safety. 2010 Nov;9(6):620-34. https://doi.org/10.1111/j.1541-4337.2010.00130.x

47. Kečkeš S, Gašić U, Veličković TĆ, Milojković-Opsenica D, Natić M, Tešić Ž. The determination of phenolic profiles of Serbian unifloral honeys using ultra-high-performance liquid chromatography/high resolution accurate mass spectrometry. Food Chemistry. 2013 May 1;138(1):32-40. https://doi.org/10.1016/j.foodchem.2012.10.025

48. Kędzierska-Matysek M, Stryjecka M, Teter A, Skałecki P, Domaradzki P, Florek M. Relationships between the content of phenolic compounds and the antioxidant activity of Polish honey varieties as a tool for botanical discrimination. Molecules. 2021 Mar 23;26(6):1810. https://doi.org/10.3390/molecules26061810

49. Khalil MI, Moniruzzaman M, Boukraâ L, Benhanifia M, Islam MA, Islam MN, Sulaiman SA, Gan SH. Physicochemical and antioxidant properties of Algerian honey. Molecules. 2012 Sep 20;17(9):11199-215. https://doi.org/10.3390/molecules170911199
50. Khansaritoreh E, Salmaki Y, Azirani TA, Henareh F, Alizadeh K, Ramezani E, Zarre S, Beckh G, Behling H. The sources and quality of Iranian honey. Heliyon. 2021 Apr 1;7(4):e06651. https://doi.org/10.1016/j.heliyon.2021.e06651
51. Kier G, Mutke J, Dinerstein E, Ricketts TH, Küper W, Kreft H, Barthlott W. Global patterns of plant diversity and floristic knowledge. Journal of Biogeography. 2005 Jul;32(7):1107-16. https://doi.org/10.1111/j.1365-2699.2005.01272.x
52. Klein JC, Lacoste A. The oak groves of Quercus macranthera F. and M. in the Alborz mountain (Iran) and the eastern extremities of the Great and Little Caucasus. Ecologia Mediterranea (France). 1989. https://scholar.google.com/scholar?hl=en&as_sdt=0%2C5&q=%5BThe+oak+groves+of+Quercus+macranthera+F.+and+M.+in+the+Alborz+mountain+%28Iran%29+and+the+eastern+extremities+of+the+Great+and+Little+Caucasus&btnG=
53. Klein B, Leffler KB. The role of market forces in assuring contractual performance. Journal of political Economy. 1981 Aug 1;89(4):615-41. https://doi.org/10.1086/260996
54. Klein R. The new politics of the National Health Service (Doctoral dissertation, Texas Tech University).
55. Kocot J, Kiełczykowska M, Luchowska-Kocot D, Kurzepa J, Musik I. Antioxidant potential of propolis, bee pollen, and royal jelly: possible medical application. Oxidative medicine and cellular longevity. 2018 May 2;2018. https://doi.org/10.1155/2018/7074209

56. Kortesniemi M, Rosenvald S, Laaksonen O, Vanag A, Ollikka T, Vene K, Yang B. Sensory and chemical profiles of Finnish honeys of different botanical origins and consumer preferences. Food Chemistry. 2018 Apr 25;246:351-9. https://doi.org/10.1016/j.foodchem.2017.10.069
57. Kuldasheva F, Madartov B, Nurmurodova N, Eshdavlatov O. STUDY OF ORGANOLEPTIC, PHYSICAL AND CHEMICAL INDICATORS OF HONEY IN TASHKENT REGION. Agro Inform. 2022(1):43-51.https://cyberleninka.ru/article/n/study-of-organoleptic-physical-and-chemical-indicators-of-honey-in-tashkent-region
58. Louveaux J, Maurizio A, Vorwohl G. Methods of melissopalynology. Bee world. 1978 Jan 1;59(4):139-57.https://doi.org/10.1080/0005772X.1978.11097714
59. Louveaux J, Maurizio A, Vorwohl G. Methods of melissopa-lynology. Bee World. 1970 51:25–138https://doi.org/10.1080/0005772X.1970.11097312
60. Machado De-Melo AA, Almeida-Muradian LB, Sancho MT, Pascual-Maté A. Composition and properties of Apis mellifera honey: A review. Journal of apicultural research. 2018 Jan 1;57(1):5-37.https://doi.org/10.1080/00218839.2017.1338444
61. Martos I, Ferreres F, Tomás-Barberán FA. Identification of flavonoid markers for the botanical origin of Eucalyptus honey. Journal of Agricultural and Food Chemistry. 2000 May 15;48(5):1498-502.https://doi.org/10.1021/jf000277i
62. Maurizio A. Microscopy of honey. Honey: A Comprehensive Survey. E. Crane, ed. 1975.https://scholar.google.com/scholar?hl=en&as_sdt=0%2C5&q=Maurizio%2C+A.+1975.+Microscopy+of+honey.+%E2%80%93+In%3A+Honey%3A+&btnG=

63. Mehrabian A, Rad MA. Distribution patterns and priorities for conservation of Iranian Endemic Monocots: determining the Areas of Endemism (AOEs). Journal of Wildlife and Biodiversity. 2021 Mar 30;5(2):69-87.https://doi.org/10.22120/jwb.2020.136616.1188
64. Memariani F. A pollen study of some honey samples in Khorasan province. Journal of Research and Construction. 2000;17(4):49-83.
65. Menad H, Ben-Naoum F, Amine A. A hybrid grey wolves optimizer and convolutional neural network for pollen grain recognition. International Journal of Swarm Intelligence Research (IJSIR). 2020 Jul 1;11(3):49-71.https://doi.org/10.4018/IJSIR.2020070104
66. Molan P. Why honey is effective as a medicine: 2. The scientific explanation of its effects. Bee world. 2001 Jan 1;82(1):22-40.https://doi.org/10.1080/0005772X.2001.11099498
67. Nanda V, Sarkar BC, Sharma HK, Bawa AS. Physico-chemical properties and estimation of mineral content in honey produced from different plants in Northern India. Journal of Food Composition and Analysis. 2003 Oct 1;16(5):613-9. https://doi.org/10.1016/S0889-1575(03)00062-0
68. Nayik GA, Nanda V. Application of response surface methodology to study the combined effect of temperature, time and pH on antioxidant activity of cherry (Prunus avium) honey. Polish Journal of Food and Nutrition Sciences. 2016 Dec 31;66(4):287-93. https://doi.org/10.1515/pjfns-2015-0055
69. Nazarian, H., Sanei Shariat Panahi, M. & tahmasebi, G.H. 1997. Determination of Identification of plants used by bees in Tehran province. The second Conference on Honey bee. Iran Animal Science Research Institute.

70. Noroozi J, Körner C. A bioclimatic characterization of high elevation habitats in the Alborz mountains of Iran. Alpine botany. 2018 Apr;128:1-1. https://doi.org/10.1007/s00035-018-0202-9

71. Noroozi J, Pauli H, Grabherr G, Breckle SW. The subnival–nival vascular plant species of Iran: a unique high-mountain flora and its threat from climate warming. Biodiversity and Conservation. 2011 Jun;20:1319-38.https://doi.org/10.1007/s10531-011-0029-9

72. Ouchemoukh S, Louaileche H, Schweitzer P. Physicochemical characteristics and pollen spectrum of some Algerian honeys. Food control. 2007 Jan 1;18(1):52-8. https://doi.org/10.1016/j.foodcont.2005.08.007

73. Ozturk F, Erkan C. Bee plants of Van Lake Basin (Turkey). International Journal of Botany. 2010;6(2):101-6. https://www.cabdirect.org/cabdirect/abstract/20103306252

74. Pasupuleti VR, Sammugam L, Ramesh N, Gan SH. Honey, propolis, and royal jelly: a comprehensive review of their biological actions and health benefits. Oxidative medicine and cellular longevity. 2017 Oct;2017. https://doi.org/10.1155/2017/1259510

75. Ponnuchamy R, Bonhomme V, Prasad S, Das L, Patel P, Gaucherel C, Pragasam A, Anupama K. Honey pollen: using melissopalynology to understand foraging preferences of bees in tropical South India. PLoS One. 2014 Jul 8;9(7):e101618. https://doi.org/10.1371/journal.pone.0101618

76. Puusepp L, Koff T. Pollen analysis of honey from the Baltic region, Estonia. Grana. 2014 Jan 2;53(1):54-61. https://doi.org/10.1080/00173134.2013.850532

77. Rao PV, Krishnan KT, Salleh N, Gan SH. Biological and therapeutic effects of honey produced by honey bees and stingless bees: a comparative review. Revista Brasileira de Farmacognosia. 2016 Sep;26:657-64.https://doi.org/10.1016/j.bjp.2016.01.012

78. Rašić S, Štefanić E, Antunović S, Jović J, Kristek S. Pollen analysis of honey from north-eastern Croatia. Poljoprivreda. 2018 Dec 19;24(2):43-9.https://doi.org/10.18047/poljo.24.2.6
79. Razzaghy Kamroudi M, Sanei Shariat Panahi M, Nazarian H, Qelichnia Hassan. Identification of pollens in honeys of Nourrud watershed of Mazandaran. Research and construction. 2006 [cited 2022May14]; 19 (3 (p. 72) on livestock and aquaculture): 74-83.
80. Safi Ahmad Abad M, Shirvany A, Panahi P. Comparison of pollen grain composition and quality of honey obtained from forest and rangeland sites in Arasbaran region. Iranian Journal of Forest and Poplar Research. 2017 Jun 22;25(2):342-53. https://dx.doi.org/10.22092/ijfpr.2017.111783
81. Salmanpour F, Shakoori Z, Kia M, Eshaghi R, Ghaderi M, Ghomi S, Kaveh R, Rabie K, Kiabi BH, Farhadinia MS. Mineral lick use by a community of large herbivores in northern Iran. Ecology and Evolution. 2023 Jan;13(1):e9731. https://doi.org/10.1002/ece3.9731
82. Salmanpour F, Shakoori Z, Kia M, Ghomi S. Investigating the Factors Affecting Leopard (Panthera pardus tulliana) Conflict with Domestic Livestock Compared to other Large Carnivores in Nowshahr Hyrcanian Forests. Journal of Animal Environment. 2021 Jul 23;13(2):1-8. https://www.aejournal.ir/article_135105.html?lang=en
83. Sanei Shariat Panahi, M, & Said Abadi, V. Nectriferous plants in Karaj based on palynology. Journal of Agricultural Faculty. 1974; 2-3: 35-47.
84. Saxena S, Gautam S, Sharma A. Physical, biochemical and antioxidant properties of some Indian honeys. Food chemistry. 2010 Jan 15;118(2):391-7. https://doi.org/10.1016/j.foodchem.2009.05.001
85. Selvaraju K, Vikram P, Soon JM, Krishnan KT, Mohammed A. Melissopalynological, physicochemical and antioxidant properties of honey from West Coast of Malaysia. Journal

of food science and technology. 2019 May 1;56:2508-21. https://doi.org/10.1007/s13197-019-03728-3

86. Shakoori Z, Mehrabian A, Salmanpour F. Evaluation of plant origin of honey based on pollen evidence in some prominent honey-producing zones in Iran, 47th Apimondia Congress (0355). 2022; pp-124Ref.0355. https://www.researchgate.net/publication/363134174

87. Stöcklin J. Salt deposits of the Middle East. Geological Society of America; 1968 Jan. https://books.google.com/books?hl=en&lr=&id=uwGmwnjjM5YC&oi=fnd&pg=PA157&dq=87.%09St%C3%B6cklin,+J.,+1968.+Salt+deposits+of+the+Middle+East.+&ots=QRqRrTvKhb&sig=BDdWzNrkq8YwaQG9lrqFcse05No#v=onepage&q&f=false

88. Subrahmanyam M. A prospective randomised clinical and histological study of superficial burn wound healing with honey and silver sulfadiazine. Burns. 1998 Mar 1;24(2):157-61. https://doi.org/10.1016/S0305-4179(97)00113-7

89. Takhtajan AL. Floristic regions of the world. Berkeley University of California Press. 54 B. Djavadi & S. M Ghaffari. 1986.

90. Tucak Z, Tucak A, Puskadija Z, Tucak M. Nutritions-healing structure of some kinds Honey in eastern Croatia. In8 th International Symposium Animal science days, Osijek 2000 (pp. 129-131). https://www.researchgate.net/profile/Martin-Verstegen/publication/40140722_Ileal_digestibility_of_amino_acids_in_pig_feeds_and_its_use_in_diet_formulations/links/53db7c780cf2631430cb5153/Ileal-digestibility-of-amino-acids-in-pig-feeds-and-its-use-in-diet-formulations.pdf?_sg%5B0%5D=started_experiment_milestone&origin=journalDetail&_rtd=e30%3D#page=129

91. Von Der Ohe W, Oddo LP, Piana ML, Morlot M, Martin P. Harmonized methods of melissopalynology. Apidologie. 2004;35(Suppl. 1):S18-25. DOI: 10.1051/apido:2004050 https://www.apidologie.org/articles/apido/pdf/2004/06/MHS07.pdf
92. Yao L, Datta N, Tomás-Barberán FA, Ferreres F, Martos I, Singanusong R. Flavonoids, phenolic acids and abscisic acid in Australian and New Zealand Leptospermum honeys. Food Chemistry. 2003 May 1;81(2):159-68. https://doi.org/10.1016/S0308-8146(02)00388-6
93. Zahran MA. Climate-Vegetation:: Afro-Asian Mediterranean and Red Sea Coastal Lands. Springer Science & Business Media; 2010 Mar 10. https://books.google.com/books?id=rRIBe5OyF5MC&printsec=frontcover#v=onepage&q&f=false
94. Zimmerman MI. Nectar production, flowering phenology, and strategies for pollination. Plant reproductive ecology: patterns and strategies. 1988 Jul 7;41:157-78. https://books.google.com/books?id=Yjv1woP0QtYC&printsec=frontcover#v=onepage&q&f=false
95. Zohary M. Geobotanical foundations of the Middle East. Fischer; 1973. https://ixtheo.de/Record/1154164403

Assessing the Quality of Bee Honey on the ~~basis~~ Basis of ~~melissopalynology~~ Melissopalynology as well as ~~c~~Chemical ~~analysis~~Analysis: ~~a A geobotanic Geobotanic approachApproach~~

Formatted: Strikethrough

Zahra Shakoori[1], Ahmadreza Mehrabian[*1], Dariush Minai[1], Farid Salmanpour[2], Farzaneh Khajoei Nasab[1]

1. Department of Plant Science and Biotechnology, Faculty of Science and Biotechnology, Shahid Beheshti University, Tehran, Iran, Email: zahra_shakoori.eco@yahoo.com, https://orcid.org/0000-0003-3057-8981 a_mehrabian@sbu.ac.ir, https://orcid.org/0000-0001-6633-3092 d_mtehrani@sbu.ac.ir, https://orcid.org/0000-0003-3589-7324 Farzaneh.khajoei@yahoo.com, https://orcid.org/0000-0002-2325-9555
2. Department of Biodiversity and Ecosystem Management, Research Institute of Environmental Sciences, Shahid Beheshti University, Tehran, Iran, Email: farid.salmanpour@yahoo.com, https://orcid.org/0000-0002-1517-9061

* Corresponding author

E-mail: testperson@university.ed (AM)

Abstract

Melissopalynological analysis and chemical composition of honey provide us with useful and valuable information about the botanical and geographical origin of honey. The data in question is very important for authentication as well as for testing the quality ~~testing~~ of honey, so ~~it~~ this is considered ~~as~~ a ~~key~~ the main method in honey regulation here, we have used chemical analysis and melissopalynology ~~and chemical analysis~~ to evaluate different honey samples from two main Iranian hubs of honey. Sampling was carried out ~~at~~ on two important poles in Iran's ~~of Iranian~~ honey production, the central Alborz region, and the mountainous Zagros ecosystems ~~mountain ecosystems of Zagros~~ in the years 2020 to 2021. Therefore, 52 samples from Alborz (Northern ~~N.~~ Iran), as well as 42 samples from the Zagrosian ecosystems (western Iran) belonging to different ecological~~ly~~ habitats, were collected. In addition, samples were taken at 7 altitudes from~~altitudinal levels between~~ 0 to 3500 m a.s.l. ~~Furthermore~~Furthermore, in this study, various chemical analyses ~~analyzes~~ such as the effect of antioxidant activity, the amount of total phenolic content, ~~the~~ pH, and moisture content of honey samples were evaluated. Our results showed that all honey samples were classified as polyfloral honey. Based on our findings, 57 honey samples (61%) contained the standard amount of pollen. A total of 42 plant families and 55 genera were identified in the studied samples, with the highest presence of Asteraceae, Fabaceae, Rosaceae, Apocynaceae, and Apiaceae. Finally, ~~the~~ an antioxidant activity ratioe ~~was~~ of 19% to 98%, total phenolic content from 0.08 to 0.51 ppm, pH–from 1.90 to 5.21, and moisture ~~humidity~~ ~~from~~content from 13% to 18.40%.

Formatted: Highlight
Formatted: Highlight
Formatted: Highlight
Formatted: Highlight
Formatted: Highlight
Formatted: Highlight
Formatted: Highlight
Formatted: Highlight
Formatted: Highlight
Formatted: Highlight
Formatted: Highlight
Formatted: Font color: Red
Formatted: Highlight
Formatted: Highlight
Formatted: Highlight
Formatted: Highlight
Formatted: Highlight
Formatted: Font color: Red
Formatted: Highlight
Formatted: Highlight
Formatted: Font color: Red
Formatted: Highlight

Formatted: Font color: Red
Formatted: Highlight
Formatted: Font color: Red
Formatted: Highlight
Formatted: Highlight
Formatted: Highlight
Formatted: Font color: Red
Formatted: Highlight
Formatted: Font color: Red

~~**Keywords:** Melissopalynology, Botanical origin, Scanning Electron Microscopy (SEM), Iran, Pollen, palynology.~~

Introduction

Honey is a precious natural product that is processed by bees from the nectar of flowers~~,~~ and ~~is~~ stored in the beehives~~hive~~. Honey production in an area depends mainly on the vegetation and climate of the region, as the flowering season and nectar production can be different ~~vary~~ for a species in different regions (Zimmerman, 1988). With the growth of the worldwide honey market~~de~~ ~~veloping the of the honey market through the world~~ the identification of the botanical origin as well as its authenticity has received ~~a lot of~~much attention in the international society ~~societies~~ (Kaskonine & Venskutonis, 2010). Counterfeit honey and poor-quality honey are major problems in the global honey trade. Honey, which consists ~~is composed, of manyof~~ of many stable elements as well as pollen grains, is collected by bees along with nectar. Melissopalynology as an effective method, as well as key criteria, has been widely used to determine the purity, geographical, and botanical origins of natural honey (Maurizo 1975~~, ;~~; Alvarez-Suarez 2017). Louveaux (1970, 1978) was the first ~~to founded this~~establish this method (Louveaux et al. 1970; 1978). However, several laboratory procedures have been developed to study of pollen grains in honey latter (Bryant & Jones 2001). Melissopalynology studies evaluated pollen grains in honey (Jones and Bryant 1996 & Louveaux et al. 1970 & Corvucci et al. 2015 & Menad et al.2020).

Pollen analysis provides valuable data on the botanical and geographical origins of honey and is a ~~major~~ key method ~~of~~ for honey authentication (Shakoori et al.2022).The ~~plant~~ botanical and geographical origins of honey is important ~~for~~ to the consumers and influences ~~affects~~ the product quality and~~,~~ physical, chemical, organoleptic, and bioactive properties of honey, which is

considered as a valuable indicator ~~for~~ of honey ~~the~~ quality and authenticity ~~of honey~~ (Alnaqdy et al. 2005; Basualdo et al. 2007; Bogdanov et al. 2008, Kortesniemi et al. 2018). Furthermore, the quality of ~~honey~~ honey is largely dependent ~~mainly depends~~ on the nectariferous plant species ~~taxa~~ that honey bees use in their diet. Accordingly, different melliferous plant species have different characteristics ~~as well~~ ~~as~~and ~~different~~ applications (eg. ~~e.g.~~ drugs, nutrients, ~~etc.)~~(etc.) (Ozturk and Ercan 2010, Tucak et al. 2000~~,)~~,).

Iran was ranked 9th in the world for honey production from 1993 to 2018 (FAOSTAT, 2018). High climate variability~~—~~, topographic complexity (Stöklin, 1968; Zohary, 1973), the geographical isolation of the ~~Iranian~~ highlands of Iran (Noroozi et al., 2011) as well as soil diversity (Dewan & Famouri, 1963) established different phytogeographical units in Iran (Takhtajan, 1986; Zahran, 2010), ~~which made~~making it the center of a endemism ~~of~~ for plant taxa in Southwest Asia (Klein, 1972; Hedge & Wendelbo, 1978), the center of endemism of Irano-Turanian region (Takhtajan, 1986; Klein, 1981 & 1992; Leonard, 1991), ~~as well~~ ~~as~~and an important center of plant diversity ~~around~~ in the world (Davis & Heywood, 1994; Barthlott, 1996 & 1999; Kier et al., 2005). These ecological conditions have led to the formation of valuable beekeeping environments~~habitats for beekeeping~~ in the country, making honey production in Iran very diverse~~which makes high diversity of honey production in Iran~~.

The Alborz ~~together~~ ~~with~~and ~~the~~ Zagros Mountains in Iran have created transition zones that have lead to a high diversity and endemism in this region (Klein, 1972, Mehrabian et al. 2021). Influenced by the high altitude~~elevation~~ diversity in this ~~area~~ ,area, a variety of~~various~~ vegetation ~~types have~~ has been formed (Fayaz et al. 2015, Ghelich Nia 2017), creating ~~which have created~~ suitable habitats for beekeeping and honey production (Nazarian et al. 1997, Razaghi et al. 2006). Today, several researchers in Iran have used melissopalynology research methods,

Formatted: Font color: Red, Highlight
Formatted: Highlight
Formatted: Font color: Red, Highlight
Formatted: Font color: Red
Formatted: Highlight
Formatted: Underline, Highlight
Formatted: Highlight
Formatted: Highlight
Formatted: Highlight
Formatted: Highlight
Formatted: Highlight
Formatted: Highlight
Formatted: Highlight
Formatted: Highlight
Formatted: Font color: Red, Highlight
Formatted: Highlight
Formatted: Highlight
Formatted: Highlight
Formatted: Highlight
Formatted: Highlight
Formatted: Highlight
Formatted: Highlight
Formatted: Highlight
Formatted: Highlight
Formatted: Highlight
Formatted: Highlight
Formatted: Highlight

including ~~the melissopalynology method has been used by a number of researchers in Iran, including:~~ ~~the~~ melissopalynology study of some honeys in Tehran Province (Nazarian et al. 1997), ~~the~~ melissopalynology ~~of honey~~ in Karaj, northern ~~N.~~ Iran (Sanei Shariat Panahi, 1974), melissopalynology of some honeys ~~of~~ from Khorasan, N.E Iran (Ghoraishi & Memariani 2000), evaluation of honey~~assessment the~~ pollen grains ~~of honey~~ in Isfahan Province, C. Iran (Faghih et al. 2004), the melissopalynology of honey in some ~~zones~~ areas of Mazandaran Province (Razaghi et al. 2006), melissopalynology of honey in Arasbaran, northwestern ~~N.W~~ Iran (Ahmad Abad et al. 2017), and origin evaluation of some Iranian honeys (Khansaritoreh et al. 2021).

In addition to nutrients, honey also contains bioactive substances that can have a positive effect on the human body (Rao et al.2016). The healthful ~~health~~ properties of honey are due to the presence of total phenolic content, including flavonoids, phenolic acids and their esters, as well as organic acids, free amino acids, vitamins, carotenoid derivatives, enzymes and biological elements (Kędzierska-Matysek et al. 2021). These compounds have antioxidant activity properties (Cianciosi et al.2018). Oxidation processes can be dealt with using antioxidant activity agents. Antioxidant activities can prevent many diseases or even reduce their symptoms (Cianciosi et al.2018). Honey can be a complementary source of antioxidant activities in a balanced diet (Alvarez-Suarez et al.2013). The antioxidant activity of honey ~~are~~ is ~~attributed~~ due to the presence of total phenolic content (Al-Mamary et al., 2002, Al Kiyumi et al.2021). The moisture ~~Moisture level of~~ content of bee honey is very important for its stability against fermentation and granulation. Low humidity ~~moisture~~ protects honey from microbial ~~microbiological~~ activity and thus can be stored longer ~~therefore can be preserved for a longer period of time.~~ (Cantarelli et al., 2008, Buba et al., 2013).

Formatted: Highlight
Formatted: Highlight
Formatted: Highlight
Formatted: Highlight
Formatted: Highlight
Formatted: Highlight
Formatted: Highlight
Formatted: Highlight
Formatted: Highlight
Formatted: Font color: Red
Formatted: Font color: Red
Formatted: Font color: Red
Formatted: Font color: Red
Formatted: Font color: Red
Formatted: Font color: Red
Formatted: Highlight
Formatted: Font color: Red
Formatted: Highlight
Formatted: Highlight
Formatted: Font color: Red
Formatted: Highlight
Formatted: Highlight
Formatted: Highlight
Formatted: Highlight

The aim of the study was to evaluate different honey samples ~~originated~~ from different ecological regions of Iran based on chemical and melissopalynological ~~and chemical~~ analyses~~analysis~~. In addition, the study tries to analyze the mentioned data in relation to geographical factors. Therefore, conducting a comparative study in different regions while comparing honey produced analyzes the relationship between pollen factors and some important chemical factors of honey with ecological factors.

Study area

~~Physical geography~~

~~Iran as a prominent area of Iranian Plateau in southwestern Asia situated in between 24° to 40° N longitude and 44° to 64° E latitude, As well a limited zone of the orogenic belt (Zagros, Alborz, and other mountain chains), covers the Asian block along with Arabian-African unity (Berberian and King, 1981). The prominent geomorphological zones of Iran including Alborz, Zagros, Kopet Dagh, as well several interior mountain chains. The Zagros mountain chain extended from northwest to southeast (Hokme, 2007). This geomorphological formation has created an natural wall between the Iranian plateau (northeast) and the Persian Gulf and Mesopotamian (southwest) basins. The Alborz mountain range is located at 36° N from the Caspian Sea up to a height of 5671 meters (Noroozi et al.2018). Also, the Alborz mountain chain as an active, arcuate fold and thrust belt (Stöcklin, 1974, 1977) as well a prominent sector of the Alpine-Himalayan geological formation. Shaped as an east-west, softly sinuous crescent in northern Iran, it forms a natural barrier between the Caspian Sea and the central Iranian plateau (Stöcklin, 1974). These natural massifs surrounding the central plateau and lead to diverse precipitation patterns in the mentioned area. In addition, Iran covers Mediterranean (western, northwestern Iran), temperate~~

Formatted: Highlight
Formatted: Highlight
Formatted: Highlight
Formatted: Font color: Red
Formatted: Font color: Red
Formatted: Highlight
Formatted: Highlight
Formatted: Highlight
Formatted: Highlight
Formatted: Highlight
Formatted: Highlight
Formatted: Font color: Red
Formatted: Font color: Red, Highlight
Formatted: Font color: Red
Formatted: Highlight
Formatted: Highlight
Formatted: Highlight
Formatted: Highlight
Formatted: Highlight

(northern Iran), and tropical (southern coast zones of the Persian Gulf and the Gulf of Oman) macro-bioclimates; however, its climatic zones consist of 35.5% hyper-arid, 29.2% arid, and 20.1% semi-arid regions. Additionally, precipitation patterns represent an average of about 250 mm annually, somewhat less than one third of the average rainfall in the world (860 mm) (Amiri and Eslamian, 2010). Most of our honey samples were collected from Alborz and Zagros sections, which have the highest honey production in Iran, which includes more than half of Iran's provinces, and most of the collected samples were close to forested and protected areas (Fig 1).

Iran is a prominent area of the Iranian Plateau in southwestern Asia situated between 24° to 40° N latitude and 44° to 64° E longitude, as well as a limited area of the orogenic belt (Zagros, Alborz, and other mountain chains. It covers the Asian block along with Arabian-African unity (Berberian and King, 1981). The main geomorphological areas of Iran, include Alborz, Zagros, Kopet Dagh, as well as several interior mountain ranges.

The Zagros Mountains extend from the northwest to the southeast (Hokme, 2007). The Alborz Mountains are located at 36° N latitude from the Caspian Sea with an elevation of 5671 meters (Noroozi et al.2018). In addition to plant species, Alborz high forests (Hyrkanian forests) host valuable animal species such as Iranian leopards (Farhadinia et al.2022). Most of our honey samples were collected from the areas of Alborz and Zagros, which have the highest honey production in Iran, including more than half of Iran's provinces, and most of the collected samples were close to forests and protected areas (Fig 1).

Formatted: Highlight

Formatted: Highlight

Formatted: Highlight

Formatted: Highlight

Formatted: Highlight

Formatted: Highlight

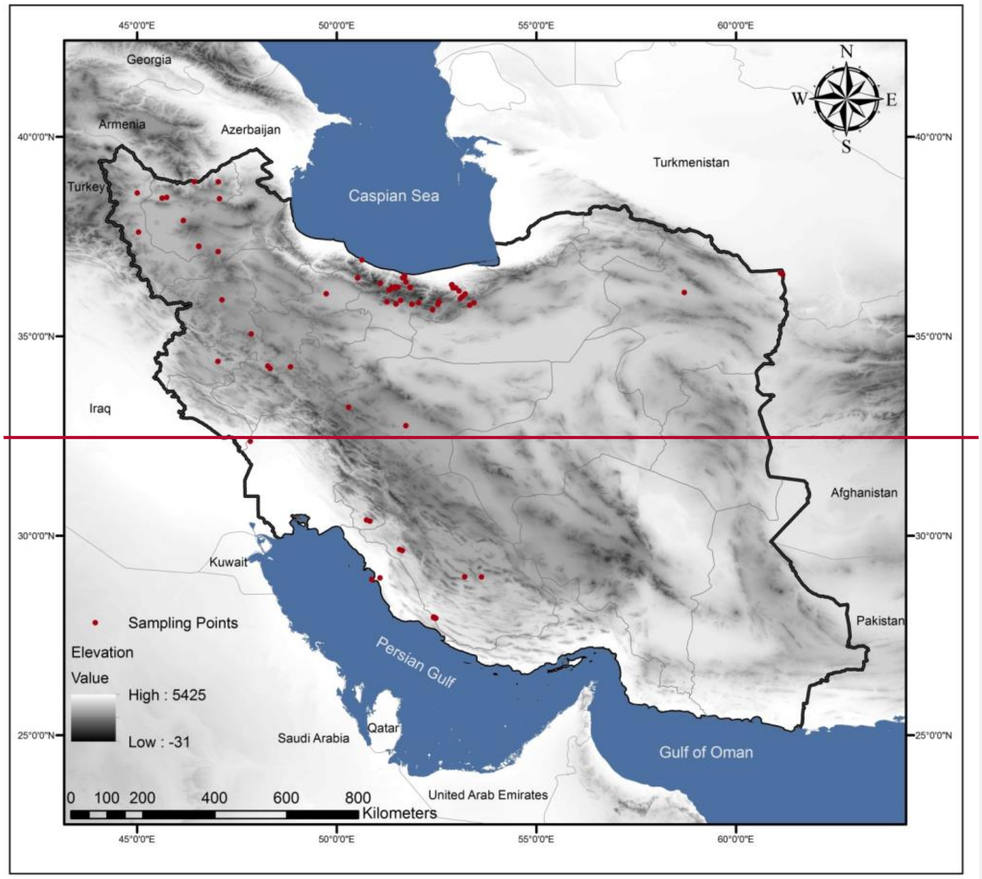

Fig 1. Study area and sample collection points

Geobotany and Plant Ecology

Formatted: Highlight

The high forests of the Alborz, which are located only on the northern slopes (the Caspian Sea side), are dominated by deciduous *Quercus macranthera*, which usually reaches an altitude of 2400 meters in Alborz exceptionally and only in small protected pockets up to 2850 meters

Formatted: Font color: Red

(Klein and Lacoste 1989; Naqinezhad and Esmailpoor 2017). In terms of plant geography, these forests belong to the Euro-Siberian region (Zohary 1973), and according to a bioclimatic classification, this region belongs to the Mediterranean pluviseasonal-oceanic biological climate using the Rivas-Martinez approach (Djamali et al. 2011). The southern slopes are mostly treeless, resulting in longer continental climates and stronger radiation during the long dry summer, although meteorological station data show that winter rainfall is still around ca. 550 mm> 2500 m a.s.l. In addition to plant species, Alborz high forests (Hyrkanian forests) host valuable animal species such as Iranian leopards (Farhadinia et al.2022). Zagros is the largest mountain range in Iran, which stretches from northeastern Iraq to southern Iran and its length is about 1300 km and width is about 250 km. This mountain range with an area of 394,000 square kilometers covers about a quarter of Iran's area (Noroozi et al. 2020). It has high peaks that reach sub-branches in different parts, but mostly in the central and southern Zagros. It has a continental climate and a Mediterranean rainy regime with cold and wet winters and hot and dry summers. Our database lists 3642 vascular plant species from this mountain range. The Zagros is located completely within the Iran-Turanian biogeographical region and the center of Iran-Anatolia biodiversity. More than 70% of the species are composed of Iranian-Turanian elements. The major vegetation types of the Zagros are oak woodlands, *Amygdalus-Pistacia* shrublands, montane steppe, shrublands, wetlands, chasmophytic habitats, subalpine, alpine and subnival zones (Noroozi et al. 2020). The vast expanse of the Zagros, its diverse climate and topography, and its high altitude range have led to the development of very different ecosystems and vegetation types. The main source of Zagros rainfall is the western systems carrying moisture from the Mediterranean and the Black Sea (Alijani and Harman 1985). The north-south extension of the Zagros Range creates a strong rain shadow effect with much less annual rainfall

Formatted: Highlight
Formatted: Highlight
Formatted: Highlight
Formatted: Highlight
Formatted: Highlight
Formatted: Highlight
Formatted: Highlight
Formatted: Highlight
Formatted: Highlight
Formatted: Highlight
Formatted: Highlight
Formatted: Highlight
Formatted: Highlight
Formatted: Font: Italic, Highlight
Formatted: Highlight
Formatted: Font: Italic, Highlight
Formatted: Highlight
Formatted: Highlight
Formatted: Highlight
Formatted: Highlight
Formatted: Highlight

~~on the eastern slopes than their western counterparts (Alijani et al. 2008). According to modern bioclimatic classifications (DJamali et al. 2011), Zagros belongs to different bioclimatic zones. Much of the western Zagros lies beneath the seasonal Mediterranean continental zone, characterized by high annual rainfall in winter and early spring, which is in favor of deciduous oak forests. This mountain range shows the climate of the arid Mediterranean continents in its northeastern parts and the conditions of the drier Mediterranean continents in its southeastern parts (Djamali et al. 2011).~~

Materials and methods

Sample collection

This study is based on a direct collection of honey samples from beehives ~~hives~~ in the ~~area~~ region from May to October ~~during~~ 2020-2021. We selected ~~sites for~~ sampling sites covering ~~that included~~ different geographical and botanical areas (Al Naggar and El-Sofany 2018). Using a data collection form, the geographical coordinates of the hives were recorded by a Garmin Map 64s GPS device, height, the type of honey and the claims of beekeepers. Based on the diversity of ecological conditions, bee colonies were identified and sampled ~~samples were taken~~ from the hives. ~~Based on the diversity of ecological conditions, bee colonies were identified and samples were taken from the hives~~. From each colony, 1 sample of 300 g of unrefined and non-clay honey was prepared for pollen extraction and chemical analysis such as pH, antioxidant activity, total phenolic content, and moisture content~~humidity~~. Samples ~~The samples~~ were placed in a sterile sealed ~~containers~~ ~~and~~container and kept ~~stored~~ in a cool place until they reached the laboratory (Ponnuchamy et al. 2014).

Laboratory studies

Formatted: Highlight

Formatted: Highlight

Formatted: Font color: Red

Formatted: Highlight

Formatted: Highlight

Formatted: Highlight

Formatted: Highlight

Formatted: Highlight

Formatted: Font color: Red

Formatted: Highlight

Formatted: Highlight

Formatted: Highlight

Formatted: Font color: Red

Formatted: Font color: Red

Formatted: Highlight

Formatted: Highlight

Formatted: Highlight

Formatted: Highlight

Formatted: Highlight

Laboratory studies were performed in two parts:

Melissopalynology based on scanning electron microscopy (SEM)

Melissopalynology analyzes based on the method of Louveaux et al. 1978 has been done (Louveaux et al. 1978). The melissopalynological method was proposed ~~recommended~~ by the International Commission on Bee Botany (ICBB) (Louveaux et al.1978; Von Der Ohe et al., 2004). First, 10 grams of honey in about 20 mL of distilled water was dissolved on a hot plate (model HP100 made by Mtops company in South Korea) at a temperature below ~~of less than~~ 40°C. After this step, the honey wax samples were passed through a cloth strainer to remove large honey wax particles ~~of~~ ~~honey wax~~, then the liquid was poured into 30 mL conical centrifuge tubes~~centrifugal tubes with conical ends and a capacity of 30 mL~~. After several centrifugation times in the laboratory ~~centrifuges~~ (2500 to 3000 ~~rpm~~g, Centrifuge model TDL-4 made by Jenius China) at room temperature (21 to 25ºC), pollen grains were extracted from the honey. In the first round, the samples were centrifuged for 10 min~~utes~~ (approximately ~~about~~ 2500 ~~rpm~~g), and the supernatant was carefully removed ~~discarded~~ to avoid ~~prevent~~ pollen loss (Ponnuchamy et al. 2014). The remaining precipitate was mixed again with about 10 mL of distilled water, but this time the samples were centrifuged for 5 min~~utes~~. In our study, we repeated this process 4 ~~to~~ – 7 times~~,~~ depending on the amount of sugar in the sample to completely remove the sugar in the honey ~~samples, so that the sugar in the honey was completely removed~~. But after the last centrifugation~~centrifuge~~, we poured the last ~~final~~ remaining liquid into the watch glass and placed it on the hot plate so that the water evaporated completely and the pollen grains remained at the bottom of the container. During thise process, extreme ~~great~~ care should ~~must~~ be taken to avoid ~~prevent~~ any contamination from external pollen~~, which is~~ from previous honey products (disposable equipment is recommended whenever possible~~use of~~

~~disposable appliances is recommended as much as possible)~~) or pollen grains in the air (close windows and limit exposure).

We placed the prepared pollen grains on special bases and, after coating ~~covering~~ the samples with gold metal, we placed them inside the housing of a Japanese-made HITACHI SU3500 scanning electron microscope (SEM) ~~machine made in Japan, .~~ ~~and depending~~ Depending on the size of the sample, using this microscope, it is possible to shoot surfaces with ~~a~~ magnification ~~of~~ from 10 to 500,000 times and ~~with a~~ resolutions ~~of less than~~below 1 to 20 nanometers. By analyzing the information obtained ~~from~~ by SEM~~electron microscopy~~, we identified and counted the pollen grains, and then obtained the ~~plant~~ botanical origin of the honey~~s~~.

Identification and counting

Using scanning electron microscopy images, pollen grains obtained from honey sediment~~s~~ were counted, Counting 500 to 1000 pollen grains are necessary to determine relative abundance (Behm et al. 1996). We also used pollen atlases and websites for pollen identification, the~~to identify pollen,~~ identification of pollen grains was done according to the morphology of the pollen grains. Pollen species should only be referred to as botanical species or species if they can be ~~are~~ reliably identified at the genus or species level, which is rare (Von Der Ohe et al. 2004).

Laboratory studies and chemical analysis of honey

All the reagents for biochemical studies were obtained from Merck Chemical Company in Germany. 2,2-Diphenyl-1-picrylhydrazyl (DPPH) was obtained from Sigma American Chemical Company.

In this part of the study, several some chemical analyses analysis of honey such as antioxidant activity, total phenolic content, pH, and moisture humidity content were investigated. For all chemical analysis honeyanalyses, honey was diluted 1/1 (w/v) by with distilled water.

Analysis of antioxidant activity by DPPH free radical scavenging activity

At this stage, the DPPH method was used to analyze all 94 samples of honey samples (Chua et al.2013),).whichWe dissolved a concentration of 0.1 mM of DPPH in methanol, and a purple solution was obtained., Then, 100 µl of DPPH was mixed with 10 µl of honey diluted honey and was incubated for 30 minutes in the dark place. The aAbsorption was recorded at 520 nm by theusing a JENUS V-1100 spectrophotometer Madespectrophotometer manufactured by DLAB China. Ascorbic acid (20 mg/-ml) was used as a positive control. The antioxidant activity was calculated from the following formula.

$$DPPH\ scavenging\ activity\ (\%) = A_{control} - A_{sample} / A_{control} \times 100$$

Analysis of total phenolic content

In this experiment, 1% ferric chloride solution (1% $FeCl_3$$FeCl_3$ 1%) was used to measure the amount of total phenolic content in of honey (Athipornchai and Klangmanee, 2021), Phenol phenol with at a concentration of 0.09% (mg /-kg) was used as a the standard sample. 0.1 ml of $FeCl_3$ was mixed with 0.1 ml of diluted honey and the absorbance was recorded at 590 nm. Phenol solution (0.09 %) was used as a the standard.

Measuring pH and the moisture of honey

Formatted: Highlight
Formatted: Highlight
Formatted: Font color: Red
Formatted: Font color: Red
Formatted: Highlight
Formatted: Highlight
Formatted: Highlight
Formatted: Font: 18 pt, Font color: Red
Formatted: Highlight
Formatted: Highlight
Formatted: Highlight
Formatted: Highlight
Formatted: Highlight
Formatted: Highlight
Formatted: Highlight
Formatted: Highlight
Formatted: Highlight
Formatted: Highlight
Formatted: Highlight
Formatted: Highlight
Formatted: Highlight
Formatted: Highlight
Formatted: Highlight
Formatted: Highlight
Formatted: Font color: Red, Highlight
Formatted: Font color: Red
Formatted: Font color: Red
Formatted: Highlight
Formatted: Subscript, Highlight
Formatted: Highlight
Formatted: Font color: Red
Formatted: Highlight
Formatted: Highlight
Formatted: Highlight
Formatted: Highlight
Formatted: Highlight
Formatted: Highlight
Formatted: Highlight

To determine the pH, ~~For pH determination,~~ 1 g of honey was mixed with 1 ml of distilled water, and the pH of each sample was measured ~~by~~ with a pH ~~Meter~~ meter model JENWAY 3505 and the moisture content of pure honey was determined with a refractometer. Using the Minitab software, we performed a regression between the mean annual relative humidity of the honey collection sites and the moisture content of the honey samples using the fitted line plots statistical model.

Formatted: Highlight
Formatted: Highlight
Formatted: Highlight
Formatted: Highlight
Formatted: Highlight
Formatted: Highlight
Formatted: Highlight
Formatted: Highlight
Formatted: Highlight

Data analysis

Formatted: Font: Bold, Highlight

Principal component analysis (PCA)

A principal component analysis (PCA) was performed to identify the main chemical factors to distinguish the honey samples studied. Chemical parameters (pH), chemical compounds (total phenolic content and antioxidant activity), as well as moisture content, were used for PCA analysis. Finally, data were analyzed using the PAST software version 3.21 (Hammer et al. 2001).

Formatted: Highlight

Formatted: Highlight

Cluster analysis

Formatted: Font: Bold, Highlight

We used clustering to determine the similarity of the honey samples studied based on the composition of the plant families contained within them. A variance-covariance matrix was generated for all the honey samples and their botanical families. The studied samples were clustered using the Euclidean distance and UPGMA (unweighted pair group method using arithmetic average) using the PAST software.

Formatted: Highlight

Results and Discussion

Pollen analysis

In this study, after analyzing pollen grains of 94 honey samples, a total of 42 plant families were identified~~—~~. Among them, Asteraceae (38%), Fabaceae (13.5%), Rosaceae (6%), and Apocynaceae and Apiaceae (5%) were the largest plant families (Fig 2). Fig 3 illustrated some pollen images of the Asteraceae, Fabaceae, Rosaceae, and Apocynaceae families (Fig 3A, B, C, D). The mentioned results show strong similarity with the following studies: Karimi et al. (2016) in Fars Province and Razzaqi et al. (2006) in Mazandaran Province. However, Asteraceae, Fabaceae, Rosaceae, Apiaceae, Brassicaceae, Papilionaceae, and Lamiaceae, respectively, showed the highest presence, in the research of Rašić et al. (2018). Furthermore, in studies by Puusepp and Koff in Estonia, plant species of the Asteraceae, Fabaceae, and Rosaceae families showed the strongest presence.~~that in total, Asteraceae families with 38%, Fabaceae with 13.5%, Rosaceae with 6%, Apocynaceae and Apiaceae with 5% each showed the highest presence (Fig 2) and 55 plant genus (Table 1) were identified.~~

Formatted: Highlight

Formatted: Highlight

Formatted: Highlight

Formatted: Highlight

Formatted: Highlight

Formatted: Highlight

Formatted: Highlight

Formatted: Font color: Red

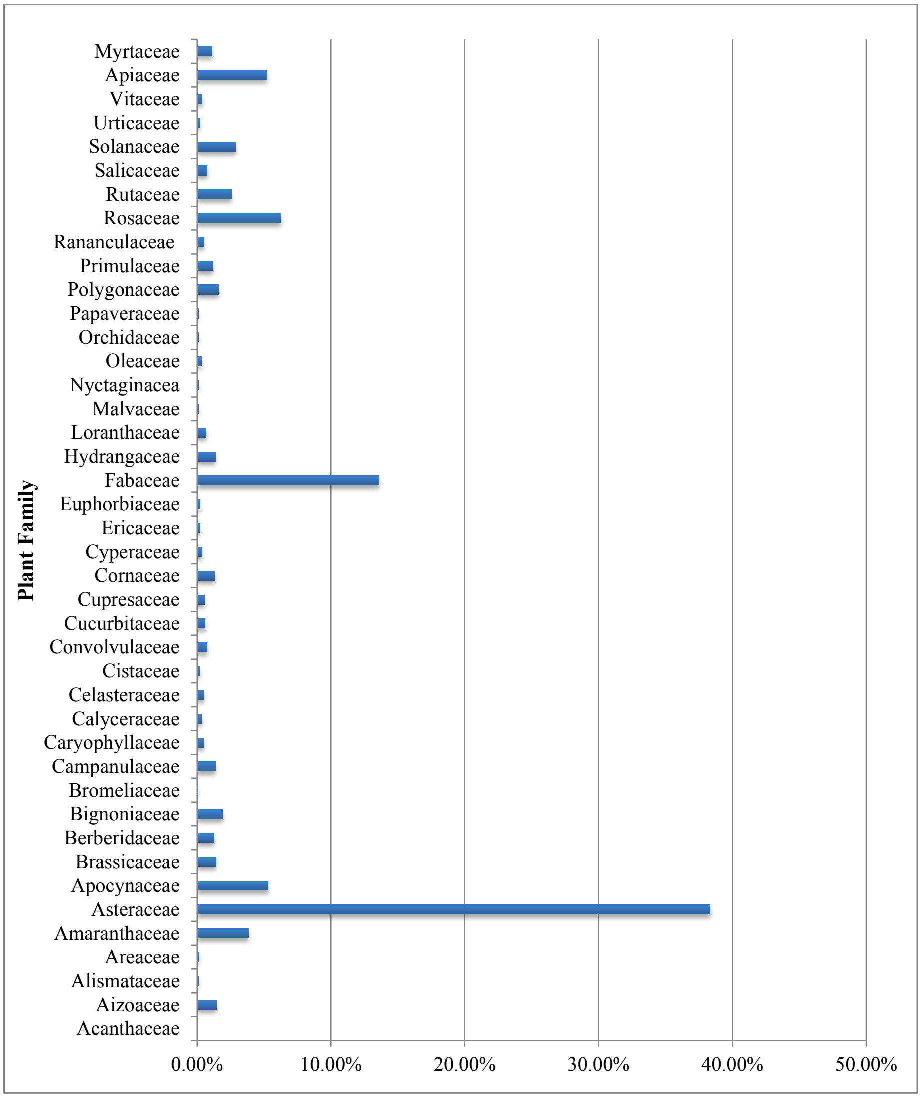

Fig 2. Percentage of presence of each plant family that was identified through analysis of pollen of 94 honey samples

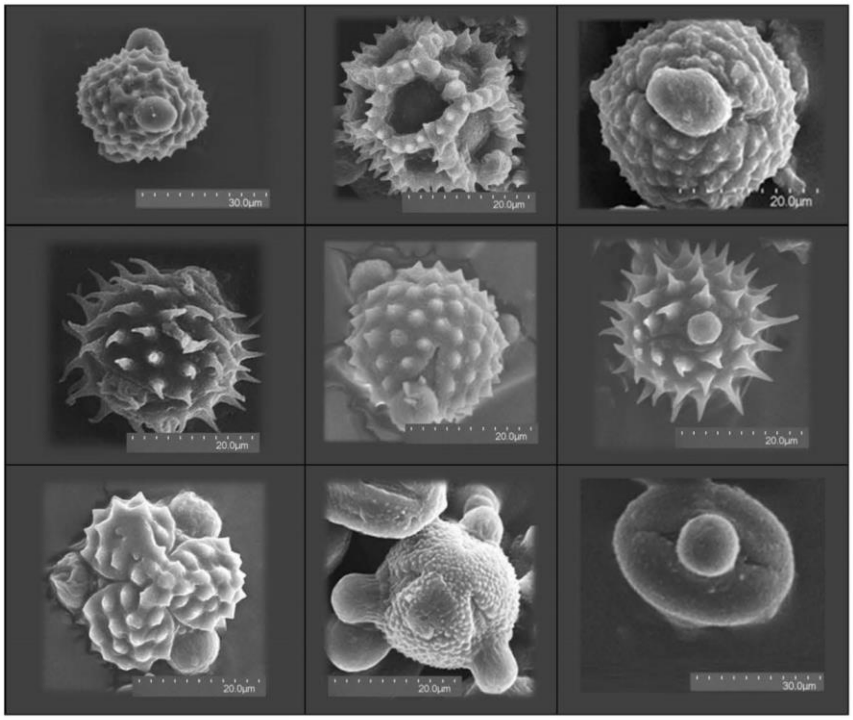

Fig 3A. Some pollen images of the Asteraceae family

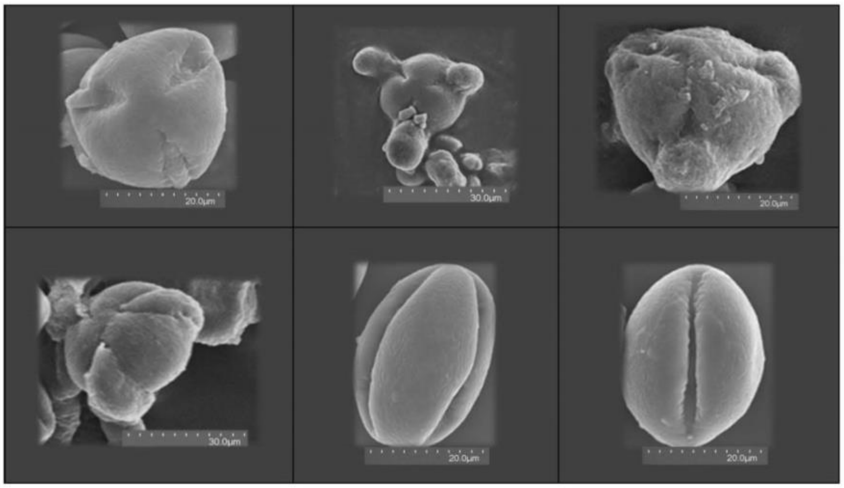

Fig 3B. Some pollen images of the Rosaceae family

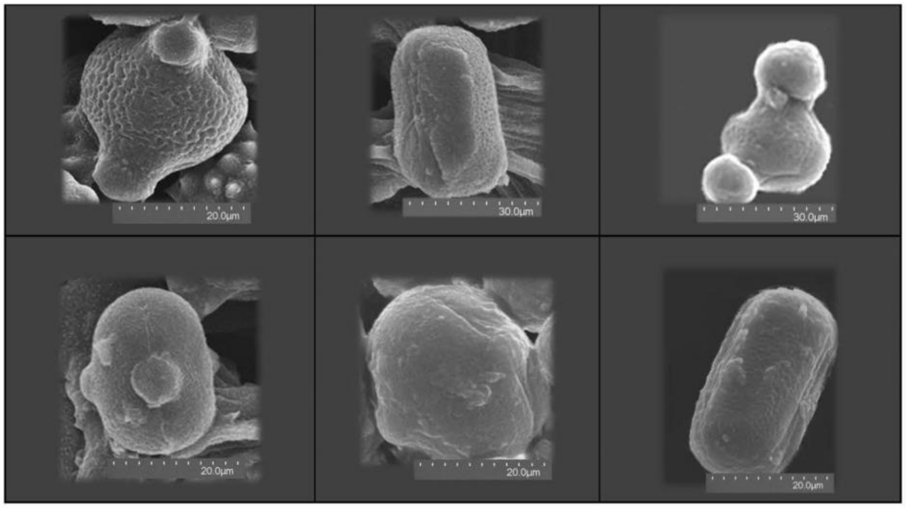

Fig 3C. Some pollen images of the Fabaceae family

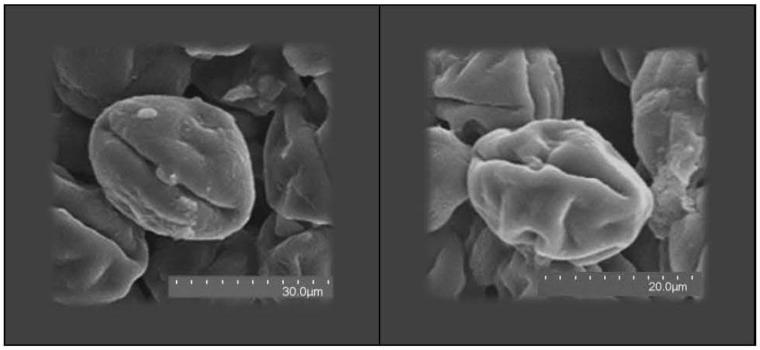

Fig 3D. some pollen images of the Apocynaceae family

In addition, 55 plant genera were identified (Table 1).

Table 1. ~~Plant genus identified from pollen analysis~~Pollen types of 94 honey samples in this study

**Family**	**Pollen type~~Genus~~**
**Amaranthaceae**	*Amaranthus* sp.
	Atriplex type
**Apocynaceae**	*Catharanthus* type
**Apiaceae**	*Apiaceae* type
	Apiaceae type
	Torilis sp.
Acanthaceae~~**Asteraceae**~~	*Acanthaceae* type
	Achillea sp.
	Artemisia sp.
	Carduus type
	Carthamus type

Formatted
Formatted: Justified, Space After: 0 pt, Line spacing: Double, Tab stops: 2.76", Left
Formatted: Highlight
Formatted: Highlight
Formatted: Font color: Text 1
Formatted Table
Formatted: Left, Space After: 0 pt, Line spacing: single
Formatted: Font color: Text 1
Formatted: Left, Space After: 0 pt, Line spacing: single
Formatted: Font color: Text 1
Formatted: Left, Space After: 0 pt, Line spacing: single
Formatted: Font color: Text 1
Formatted: Left, Space After: 0 pt, Line spacing: single
Formatted: Font color: Text 1
Formatted: Left, Space After: 0 pt, Line spacing: single
Formatted: Font: Font color: Text 1
Formatted: Left, Space After: 0 pt, Line spacing: single
Formatted: Font: Font color: Text 1
Formatted: Left, Space After: 0 pt, Line spacing: single
Formatted: Font: Not Italic, Highlight
Formatted: Font color: Text 1, Highlight
Formatted: Left, Space After: 0 pt, Line spacing: single
Formatted: Font: Font color: Text 1
Formatted: Left, Space After: 0 pt, Line spacing: single
Formatted: Font: Font color: Text 1
Formatted: Left, Space After: 0 pt, Line spacing: single
Formatted: Font: Font color: Text 1
Formatted: Left, Space After: 0 pt, Line spacing: single
Formatted: Font: Font color: Text 1
Formatted: Left, Space After: 0 pt, Line spacing: single

Asteraceae	*Centaurea* type
	Cirsium arvense
	Crepis sp.
	Doronicum sp.
	Helichrysum sp.
	Hieracium sp.
	Inula sp.
	Senecio sp.
**Bignoniaceae**	*Bignoniaceae* type 1
	Bignoniaceae type 2
**Brassicaceae**	*Brassicaceae* type
**Caryophyllaceae**	*Caryophyllaceae* type
**Cistaceae**	*Cistus* sp.
**Convolvulaceae**	*Convolvulus arvensis*
**Cornaceae**	*Cornus sanguinea*
**Cucurbitaceae**	*Cucurbit~~a~~* sp.
**Cyperaceae**	*Carex* sp.
~~**Fabaceae**~~	*Astragalus* type
	Robinia pseudacacia
**Fabaceae**	*Hedysarum* sp.
	Onobrychis cornuta
	Vicia sp.
**Hydrangeaceae**	*Philadelphus* sp.

**Myrtaceae**	*Eucalyptus calmadonensis*
**Oleaceae**	*Olea europaea*
~~**Polygonaceae**~~	*Fagopyrum* sp.
**Polygonaceae**	*Polygonum* sp.
	Rumex sp.
~~**Ranunculaceae**~~	*Adonis* sp.
**Ranunculaceae**	*Clematis* sp.
	Ranunculus sp.
~~**Rosaceae**~~	*Alchemilla* sp.
	Cotoneaster sp.
	Sorbaria sp.
**Rosaceae**	*Prunus domestica*
	Rosa cania
	Sorbaria sp.
	Spiraea sp.
**Rutaceae**	*Rutaceae* type
	Poncirus sp.
**Salicaceae**	*Populus nigra*
**Solanaceae**	*Solanum* spp.
**Urticaceae**	*Parietaria judaica*
	Urtica dioica

Our results showed that all the studied samples are classified as polyfloral honey and based on the palynological criteria, about 30% of the studied honey samples were not of good quality, the rest good quality.

Pollen grains extracted from honey samples were identified based on pollen references. A quantitative assessment ~~Quantitative evaluation~~ of pollen grains in each ~~of the~~ honey ~~samples~~ ~~was~~sample was performed according to the Louveaux standard. ~~The collected s~~Samples were collected ~~in~~ at ~~the~~ altitudes ~~ranges~~ ranging ~~of~~ from 12 to 3062 meters above sea level. ~~The s~~Samples were taken at seven altitudes~~collected in 7 altitude intervals~~, considering the variation of vegetation type and the location of beekeepers depending on the season.~~which in terms of vegetation type changes and the location of beekeepers in accordance with the season, more~~ More than 73% of the samples were taken at altitudes between ~~in the altitude range of~~ 1000 ~~to~~ and 3000 meters above sea level (Fig 3).

Formatted: Highlight
Formatted: Highlight
Formatted: Highlight
Formatted: Highlight
Formatted: Highlight
Formatted: Highlight
Formatted: Highlight
Formatted: Highlight
Formatted: Highlight
Formatted: Highlight
Formatted: Font color: Red
Formatted: Font color: Red, Highlight
Formatted: Highlight
Formatted: Font color: Red

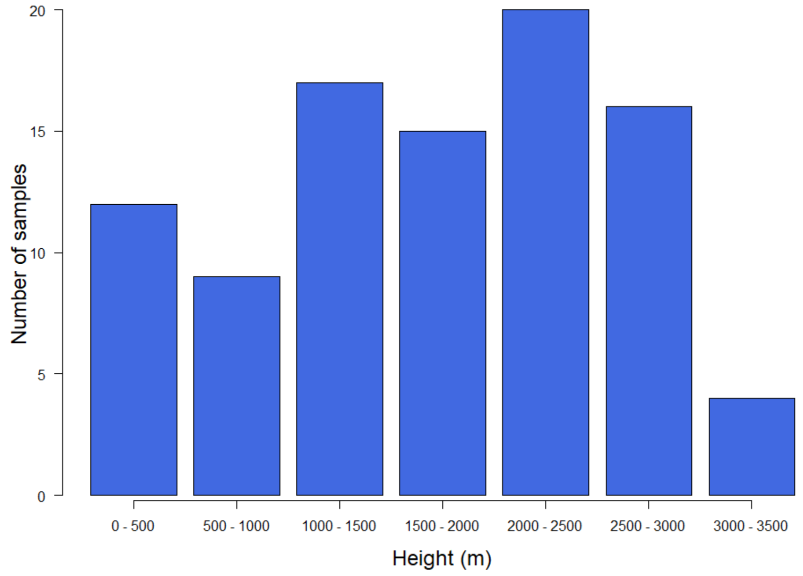

Fig 3. Number of sampling performed at different altitude intervals

Physicochemical analysis of honeys

According to the results obtained fromof the chemical analysis of honey samples, the amount of antioxidant activities in the samples was as low as the lowest of 19% and the highest of as 98%. According to (Fig 4), more than 36% of the total samples had antioxidant activities between 40% and 60%.

Formatted: Highlight

Formatted: Font color: Red

Formatted: Highlight

Formatted: Highlight

Formatted: Highlight

Formatted: Font color: Red

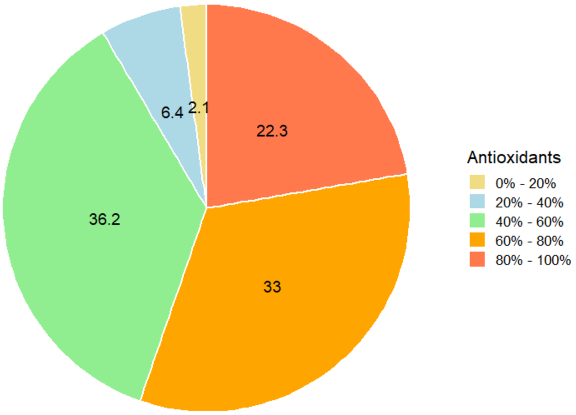

Fig 4. **The** Antioxidant antioxidant activity abundance ratio of honey samples

Formatted: Highlight

Formatted: Font color: Red, Highlight

Formatted: Font color: Red

Formatted: Font color: Red, Highlight

Formatted: Font color: Red

The chemical, physical, antioxidant and health-promoting properties of honey vary depending on the flower source, soil type, geological root, and climatic conditions CACS (2001). Ancient and modern civilizations used honey as medicine for various ailments (Berhanu et al. 2022). It is also a delicious food composed mainly of various sugars and compounds such as proteins, organic acids, amino acids, nutrients, minerals, carotenes and aromatic substances. It is rich in flavonoids and phenolic acids, exhibits many biological effects and acts as natural antioxidant activity (karabagias, et al. 2014). The results of our chemical analysis showed that the chemical composition of honey samples collected from different ecological regions was different from each other (Kuldasheva et al.2022). Chemical experiments also showed that about 3/5 of the samples showed more than 60% of the effect of antioxidant activity. We compared the results of antioxidant activities with increasing elevation, which seems to have a positive response to increasing height, and up to 3000 meters the trendline showed an upward trend, but after 3000

meters, the antioxidant activities followed the exact opposite trend. The highest efficiency of antioxidant activity was observed at altitudes between 2500 and 3000 meters, with an average of 88.71% (Figure 10). The potential variation of the antioxidant activity of honey depends on topographical and botanical differences (Martos et al. 2000), (Yao et al. 2003). The antioxidant activity of honey is mainly due to phenolic and flavonoid compounds. Thus, a significant difference in antioxidant activity is observed between different types of honey worldwide (Beretta et al., 2005; Aljadi and Kamaruddin, 2004; Nayik and Nanda, 2016).

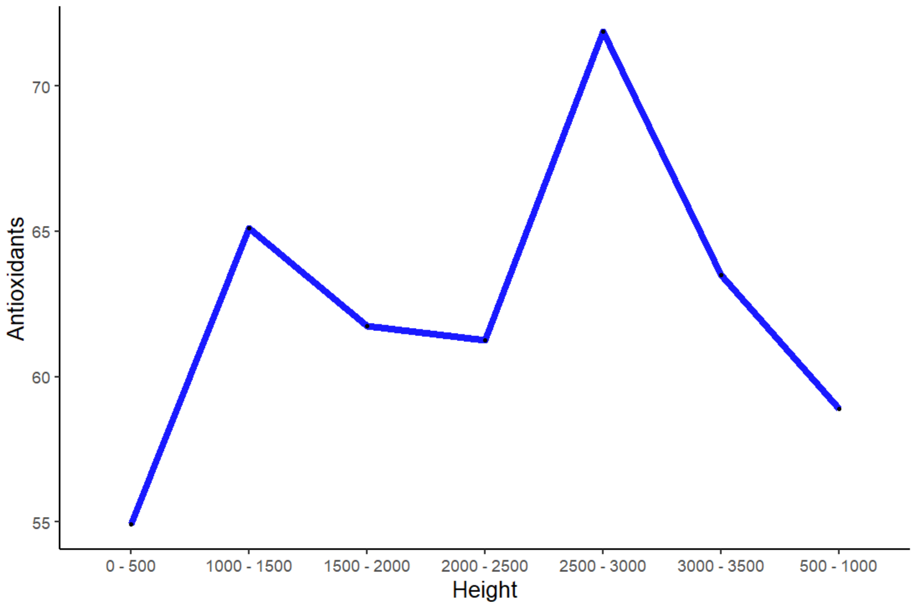

Fig 10. Comparison of antioxidant activities with altitude

The total phenolic content ~~of~~ in the honey samples was between 0.08 ~~to~~ and 0.59 micrograms. Differences in total phenolic content in honey samples may be due to different geographical origins or other factors such as collection season and storage conditions. An important group of compounds affecting the appearance and functional properties of honey is phenolic acid, which has nutritional value and medicinal properties (Alvarez-Suarez et al., 2010). The type and

Formatted: Font: (Default) +Headings CS (Times New Roman), 12 pt

Formatted: Font color: Red

Formatted: Highlight

Formatted: Highlight

Formatted: Font color: Red

Formatted: Font color: Red, Highlight

Formatted: Highlight

Formatted: Font color: Red, Highlight

Formatted: Font color: Red

Formatted: Highlight

concentration of total phenolic content in different kinds of honey are different from each other (Saxena et al. 2010). The amount of total phenolic content is influenced by various factors such as plant type, geographical origin, and climatic conditions of the honey production site (Escuredo et al. 2012). Determination of total phenolic content in honey is a suitable factor to evaluate the quality and therapeutic potential of honey (Ouchemoukh et al. 2007).

Also in this section, the pH of each honey sample was tested~~examined~~.

The lowest pH was 1.9 and the highest pH was 5.2, and 48% of the total samples were in the pH range from 2.5 to 3.5. The global standard for the pH of honey is 3.9 (3.2 to 4.5). The pH values between 3.4 and 6.1 indicate the freshness of honey samples (Khalil et al. 2012; Selvaraju et al. 2019). Honey is recognized as a therapeutic option for the treatment of acute and chronic wounds, and a variety of in vitro and in vivo research studies have been performed in this area (Subrahmanyam, 1998; Cooper et al. 2002; Johnson et al. 2005). The low pH of honey (0.4 to 5.3) promotes wound healing (Efem, 1988; Molan, 1999), which is accomplished by reducing the highly alkaline environment in non-healing wounds to a more acidic healing environment.

Formatted: Highlight

Formatted: Highlight

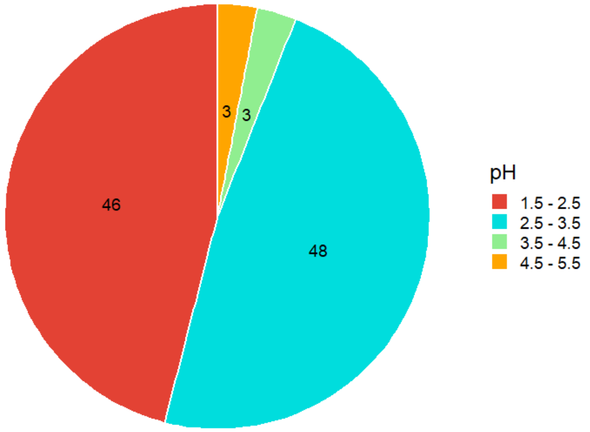

Fig 11. pH abundance ratio of the samples

In this study, all honey samples showed a low moisture content (13% to 18.80%), which corresponded to the values (< 20 <) set by the Codex standard for honey (Almentarius Codex, 1987). Moisture content is considered as an important criterion to evaluate the quality of honey. The less moisture in honey, the longer the shelf life (Fredes and Montenegro, 2006). The moisture content of honey depends on various factors such as the time of harvest, the climatic factors of the geographical area, and the storage temperature (Nanda et al., 2003; Finola et al., 2007). The parameter of quality and uniqueness of honey has been studied by many researchers, and the quality and reliability of honey is very important to both buyers and producers.

In the information obtained from measuring the moisture content of each honey sample, it was determined that the maximum moisture content was 18.8% and the minimum was 13%. Using minitab software, we made a regression between the mean annual relative humidity of the honey

Formatted: Font: (Default) +Headings CS (Times New Roman), 12 pt

Formatted: Centered, Space After: 10 pt, Tab stops: Not at 1.93"

Formatted: Highlight

Formatted: Highlight

Formatted: Highlight

Formatted: Highlight

Formatted: Highlight

Formatted: Font color: Red

Formatted: Font color: Red, Highlight

Formatted: Font color: Red

collection sites and the moistury of the honey samples using the fitted line plots statistical model.

RThe regression results between the average annual relative humidity of the region area (independent variable) and the moistury humidity –of the honey sample (dependent variable) showed that there is a significant relationship between them (p-value 0.000p value 0.000) between them, so that the moisture contenthumidity of the honey samples is a function of the humidity of the environmental humidity –(Fig 5).

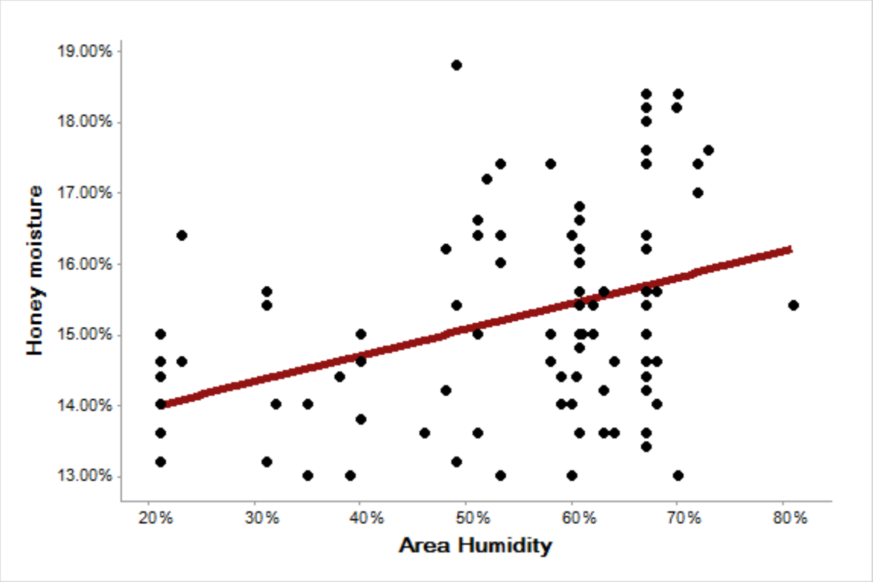

Fig 5. Regression related to the effect of environmental humidity on the moisture of honey samples

Formatted: Highlight
Formatted: Font color: Red, Highlight
Formatted: Font color: Red
Formatted: Font color: Red, Highlight
Formatted: Highlight
Formatted: Font color: Red
Formatted: Font color: Red, Highlight
Formatted: Highlight
Formatted: Font color: Red
Formatted: Font color: Red, Highlight
Formatted: Highlight
Formatted: Font color: Red
Formatted: Highlight
Formatted: Font color: Red
Formatted: Highlight
Formatted: Font color: Red
Formatted: Font color: Red, Highlight
Formatted: Highlight
Formatted: Font color: Red, Highlight
Formatted: Highlight
Formatted: Font color: Red
Formatted: Font color: Red, Highlight
Formatted: Highlight
Formatted: Font color: Red, Highlight
Formatted: Font color: Red
Formatted: Font color: Red
Formatted: Justified

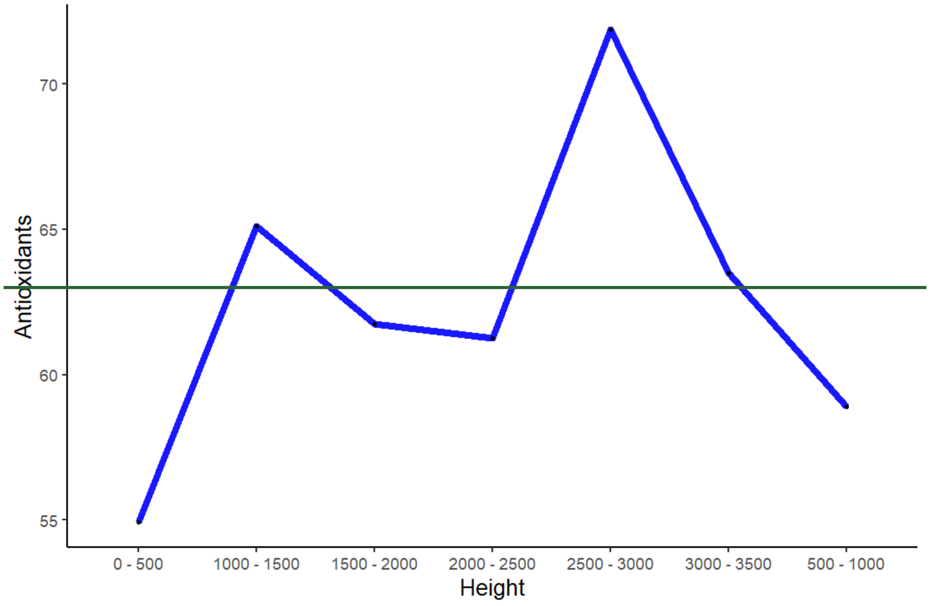

Fig 10. Comparison of antioxidant activities with altitude

Principal component analysis (PCA)

For the PCA analysis, both the first and second axes accounted for 57.19% of the total variance (Table 2). The first principal component explained 29.8% of the variance, the main chemical variables of which were: total phenol content and pH (Table 2). The second axis explained 27.39% of the variance and antioxidant activities and pH were the most significant variables (Table 2).

Table 2. Relative eigenvalue, percentage of variance, and vector values of principal component analysis (PCA) using total phenolic content, antioxidant activity, pH, and moisture content of the studied honey samples.

Name of variables	PC 1	PC 2	PC 3	PC 4
Eigenvalue	1.19215	1.09555	0.932069	0.780231
% variance	29.804	27.389	23.302	19.506
Antioxidant activities	-0.15449	**0.79953**	-0.2348	-0.53081

Formatted: Font: (Default) +Headings CS (Times New Roman), 12 pt
Formatted: Highlight
Formatted: Highlight

Total phenol content	**0.59267**	0.056052	0.69854	-0.39705
pH	**0.58965**	**0.47616**	-0.1825	0.62632
Moisture content	-0.52648	0.36179	0.65085	0.41026

The PCA did not result in any significant differences between all honey samples studied (Fig. 6). Considering that the honey samples studied were collected from different geographical areas, samples from the same area were not placed next to each other in the plot. This shows that honey samples from different locations in the same area have different chemical compositions.

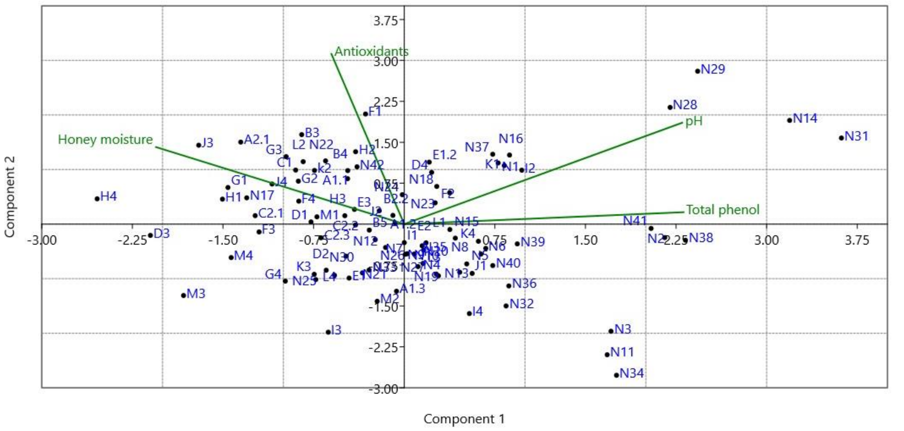

Fig 6. Principal component analysis (PCA) scatter plot of the studied honey samples based on total phenolic content, antioxidant activity, pH, and moisture content. Sample codes in Supplementary 1.

Cluster analysis

According to the cluster analysis of the plant family composition, the honey samples collected from a geographical location were located far from each other in the dendrogram and were not located in the same cluster, as shown in Fig 7.

Formatted: Highlight

Formatted: Font: (Default) +Headings CS (Times New Roman), 12 pt

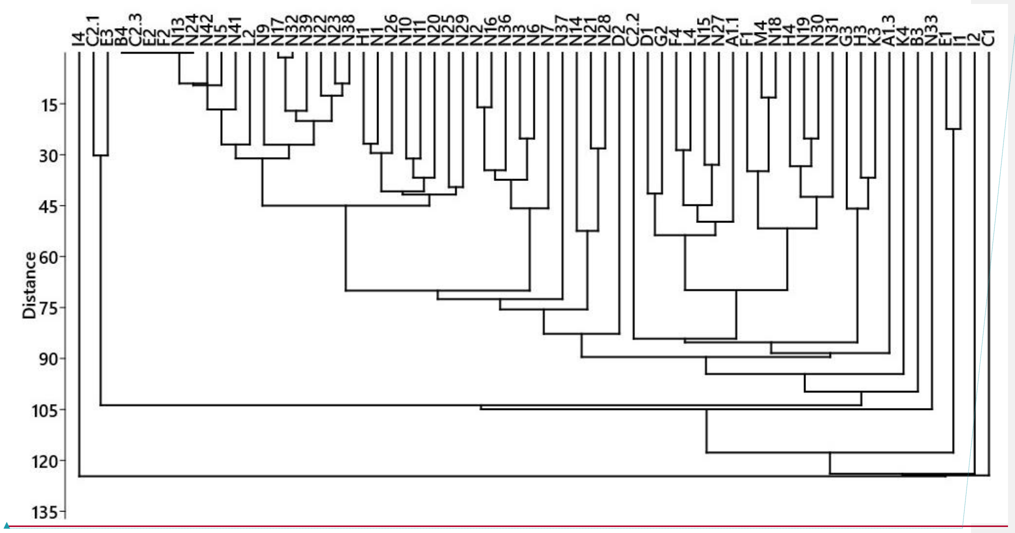

Fig 7. Dendrogram of the studied honey samples according to their botanical family composition and clustered by UPGMA (unweighted pair group method using Euclidean distance). Cophenetic correlation coefficient = 0.8565. Sample codes in Supplementary 1.

~~All honey samples were grouped with chemical factors obtained from them, which our plot (Fig 6) shows that the factors, pH and total phenolic content were positively correlated for grouping and factors, humidity and antioxidant activities for grouping were negatively correlated they had.~~

Formatted: Font: (Default) +Headings CS (Times New Roman), 12 pt

Formatted: Centered

Formatted: Highlight

Formatted: Highlight

Formatted: Font color: Red

Formatted: Font color: Red

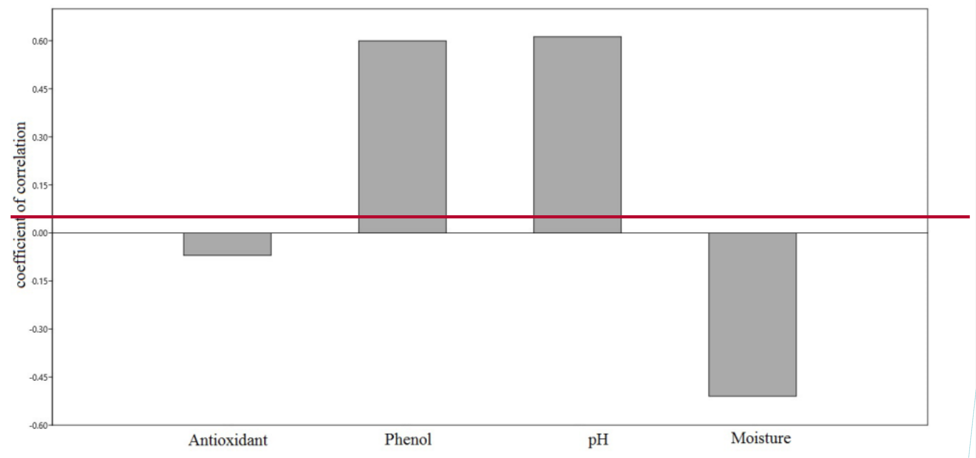

~~**Fig 6**. Plot related to the grouping of total honey samples using chemical factors~~

~~The honey samples collected in this study were from different geographical regions, based on the number of beekeepers present in each region, different honey samples were collected. Two honey samples were collected from some areas and more than two honey samples from some areas. The obtained plot (Figure 5) shows that in order to group the samples using their chemical compositions, even the samples collected from the same geographical area do not have the same chemical composition.~~

Formatted: Font: (Default) +Headings CS (Times New Roman), 12 pt

Formatted: Font color: Red

Formatted: Left

Formatted: Font color: Red

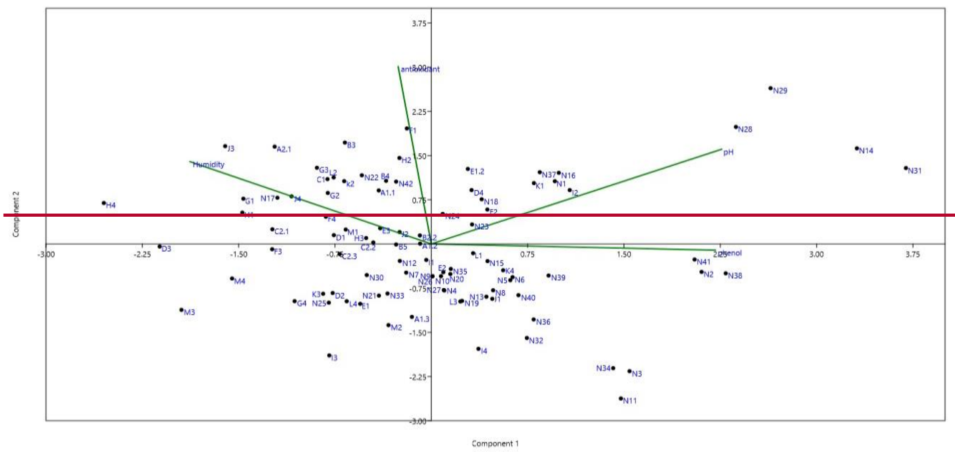

Fig 7. Grouping the samples with their chemical compounds to show the difference in the amount of chemical compounds between the samples according to their geographical areas

Based on the plant families obtained from each honey sample, we performed a cluster analysis (Fig 8) that shows how close or distant the honey samples were in terms of the composition of plant taxa .

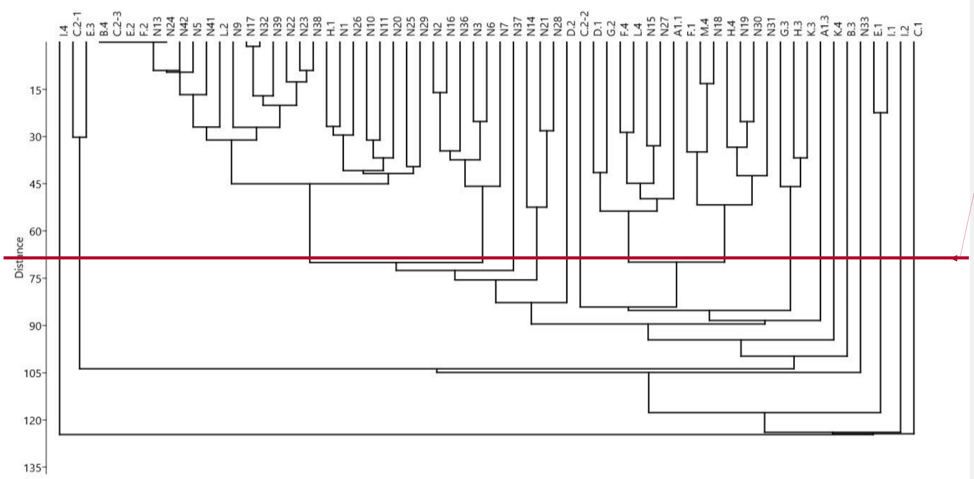

~~**Fig 8.** Cluster analysis to find out how close or distant honey samples were in terms of plant taxon composition~~

~~In this study, 94 samples of honey collected belong to the main honey producing regions in Iran. In the results of this study, 42 plant families and 57 genera of these families were identified. Due to the difficulty of identifying pollen grains extracted from honey at the genus or species level (Von Der Ohe et al.2004), we were able to identify up to the genus level in our studies.~~

~~The highest density of pollen grains in honey samples belong to the Asteraceae, Fabaceae, Rosaceae, Apocynaceae and Apiaceae (Fig 9A,B,C,D). The mentioned frequency showing high similararity to the following studies: Karimi et al. (2016) in Fars province and Razzaqi et al. (2006) in Mazandaran province. However the Asteraceae, Fabaceae, Rosaceae, Apiaceae, Brassicaceae, Papilionaceae, Lamiaceae, showed the highest presence respectiveley, In Rašić et al. (2018). In addition, in the studies of Puusepp and Koff in Estonia, Asteraceae, Fabaceae and Rosaceae plant species showed the highest presence.~~

Formatted: Justified, Space Before: 12 pt, After: 0 pt, Tab stops: 0.88", Left

Formatted: Space Before: 12 pt, Tab stops: 0.88", Left + Not at 1.93"

Formatted: Font color: Red

Formatted: Font color: Red

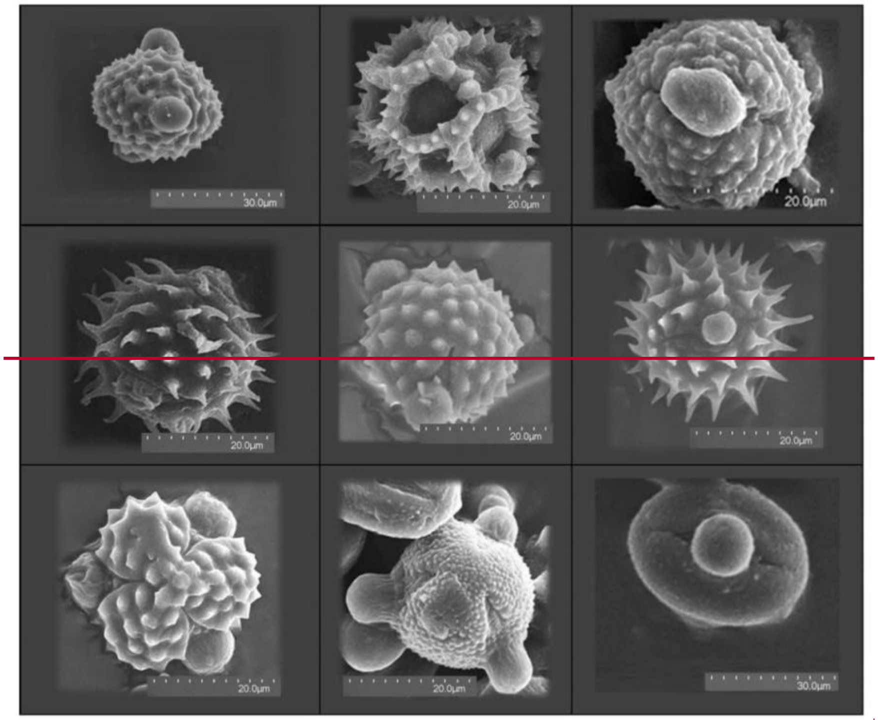

~~**Fig 9A.** Some pollen images of the Asteraceae family~~

Formatted: Justified, Space Before: 12 pt, After: 0 pt, Line spacing: Double, Tab stops: 0.88", Left

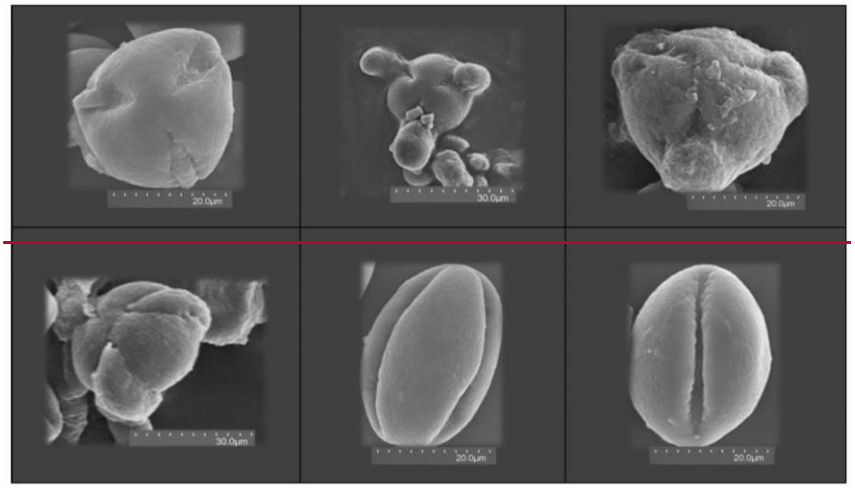

~~**Fig 9B.** Some pollen images of the Rosaceae family~~

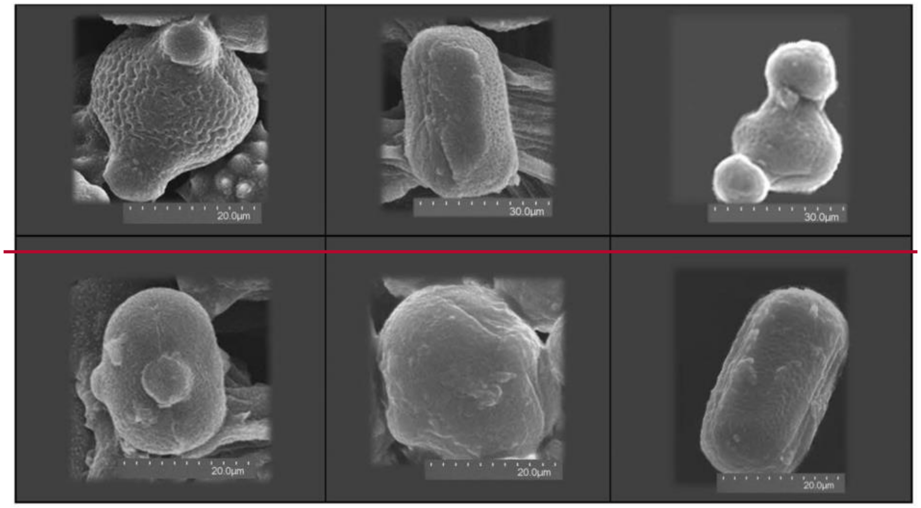

~~**Fig 9C.** Some pollen images of the Fabaceae family~~

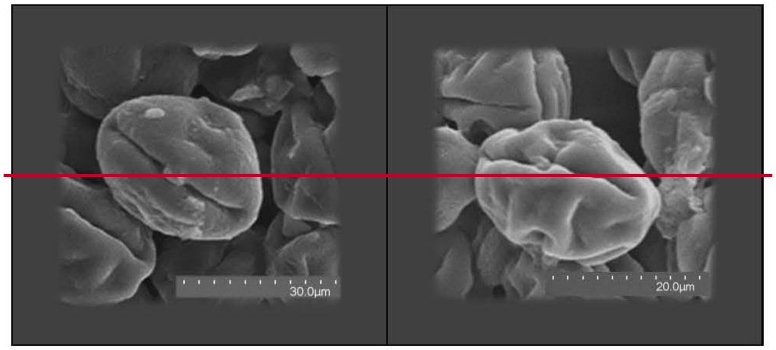

~~**Fig 9D.** some pollen images of the Apocynaceae family~~

~~Moreover The Asteraceae, Fabaceae and Rosaceae were the most present, in the Stonia (Puusepp and Koff 2014) Croatia Our results show that all the studied samples are classified as polyfloral honey and based on palynological criteria, about 30% of the studied honey samples are not of sufficient quality, but the rest of the honeys are of good quality.~~

~~The chemical, physical, antioxidant activity, and healthy composition of honey varies depending on the flower source, soil type, geological roots, and climatic conditions CACS (2001). Both ancient and modern civilizations use honey as a medicine for various diseases (Berhanu et al.2022). It is also a delicious food that is mostly made of different sugars and compounds such as proteins, organic acids, amino acids, nutrients, minerals, carotenoids and aromatic substances. It is rich in flavonoids and phenolic acids, which show a wide range of biological effects and act as a natural antioxidant activity (karabagias, et al. 2014; Manyi-Loh 2011). The results of our chemical analysis showed that the chemical composition of honey samples collected from different ecological areas are different from each other (Kuldasheva et al.2022). Chemical experiments also showed that about 3/5 of the samples showed more than 60% of the effect of antioxidant activity.~~

Formatted: Font color: Red

Formatted: Font color: Red

Formatted: Font color: Red

Formatted: Centered, Indent: Left: -0.13", Space Before: 12 pt

Formatted: Font color: Red

We compared the results of antioxidant activities with increasing height, which seems to have a positive response to increasing height and up to 3000 meters, the trend line shows an upward trend, but after 3000 meters, antioxidant activities follow a completely opposite trend. The highest effect of antioxidant activity was recorded in the altitude range of 2500 to 3000 meters, which showed an average of 88.71% (Fig 10).

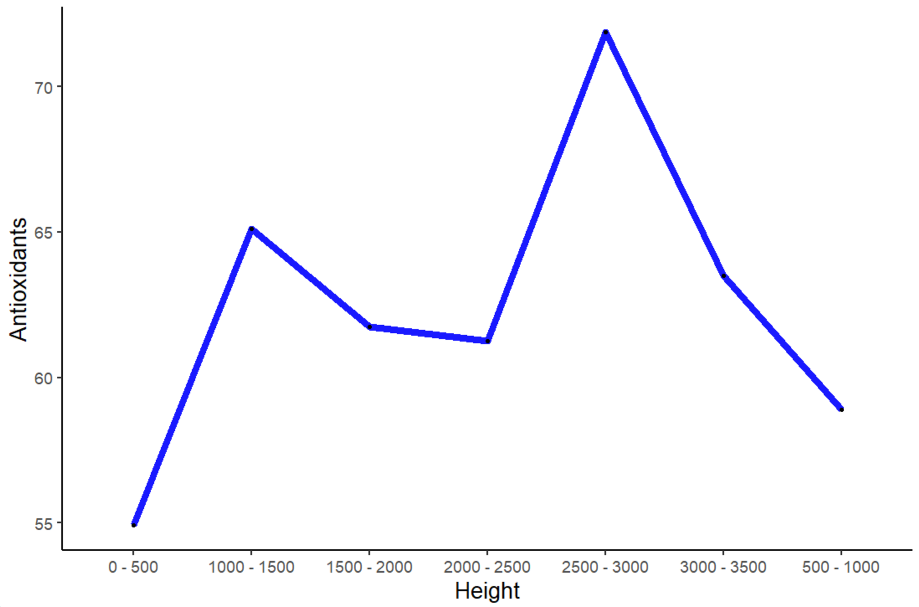

Fig 10. Comparison of antioxidant activities with altitude

Variation in the antioxidant activity potential of honey depends on topographic and botanical differences (Martos et al. 2000), (Yao et al. 2003). The antioxidant activity of honey is mainly due to phenolic and flavonoid compounds. Thus, a significant variation in antioxidant activity is

Formatted: Font color: Red

Formatted: Font color: Red

Formatted: Font color: Red

Formatted: Font: (Default) +Headings CS (Times New Roman), 12 pt

Formatted: Centered, Indent: Left: -0.13", Space Before: 12 pt, Line spacing: Double

Formatted: Font color: Red

Formatted: Font color: Red

found among different types of honey worldwide (Beretta et al., 2005; Aljadi and Kamaruddin, 2004; Nayik and Nanda, 2016). An important group of compounds that affect the appearance and functional properties of honey are phenolic acids, which have nutritional value and therapeutic properties (Alvarez-Suarez et al., 2010). The type and concentration of total phenolic content in different honeys are different from each other (Saxena et al. 2010).

The amount of total phenolic content is influenced by various factors such as plant type, geographical origin and climatic conditions of the honey production site (Escuredo et al. 2012). Determining the amount of total phenolic content in honey is a suitable factor to evaluate the quality and therapeutic potential of honey (Ouchemoukh et al. 2007). In the results of our analysis, the samples showed total phenolic content of 0.08 to 0.51 μg. Honey is recognized as a treatment option for the treatment of acute and chronic wounds, and a wide range of in vitro and in vivo research studies have been performed in this field (Subrahmanyam, 1998; Cooper et al. 2002; Johnson et al. 2005). Low pH of honey (0.4 to 5.3) helps wound healing (Efem, 1988; Molan, 1999), which is done by reducing the high alkaline environment in non healing wounds to a more acidic healing environment. The global standard for pH of honey is 3.9 (3.2 to 4.5). Based on the results of our studies, honey samples showed a pH 1.90 to 5.21 and 48% of the total samples are in the pH range of 2.5 to 3.5 (Fig 11).

Formatted: Font color: Red

Formatted: Font color: Red

Formatted: Font color: Red

Formatted: Font color: Red

Formatted: Font color: Red

Formatted: Font color: Red

Formatted: Font color: Red

Formatted: Font color: Red

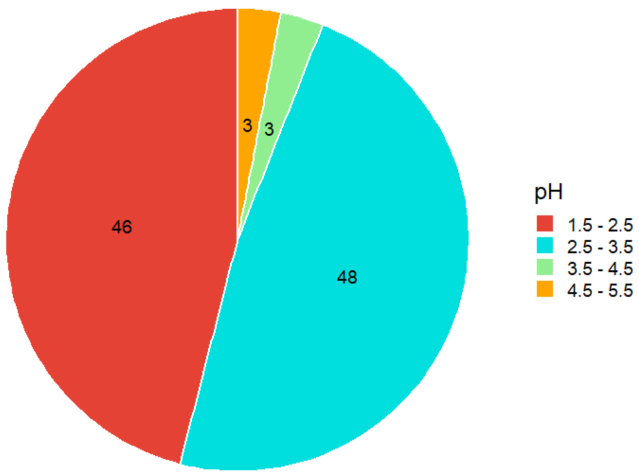

~~**Fig 11.** pH abundance ratio of the samples~~

~~And pH values between 3.4 and 6.1 indicate the freshness of honey samples (Khalil et al. 2012; Selvaraju et al. 2019). Moisture content is considered as an important criterion for measuring the quality of honey. Honey with less moisture has a longer shelf life (Fredes and Montenegro, 2006). The moisture content of honey depends on various factors such as harvest time, climatic factors of the geographical area and storage temperature (Nanda et al., 2003; Finola et al., 2007). In this study, all honey samples showed a low moisture content (13% to 18.80%), which corresponded to the values (< 20 <) set by the Codex standard for honey (Almentarius Codex, 1987). The parameter of quality and uniqueness of honey has been studied by various researchers, this quality and credibility of honey is important for both buyers and producers.~~

One of the important concerns of honey quality is to guarantee the authenticity of honey according to legal requirements. This authenticity is achieved by recognizing the plant's ~~plant~~

Formatted: Font: (Default) +Headings CS (Times New Roman), 12 pt

Formatted: Font color: Red

Formatted: Highlight

origin, geography, and chemical analysis (da Silva et al.2016), (Machado et al.2018) Nutritional and healing properties of honey from the very beginning make it an important component of traditional medicine (Bogdanov 1997; Subrahmanyam 1998). The natural antioxidant activities in honey are valuable against heart disease, cancer, inflammation, coronary heart disease, aging, tumors, gastrointestinal tract, and wound healing (de Rodríguez et al. 2004~~), (~~; Kečkeš et al.2013~~) .~~). In relation to health, honey, bee pollen, propolis, royal jelly, beeswax, and bee venom have all been used in traditional and modern medicine (Easton-Calabria et al. 2019; Kocot et al. 2018). Researchers have identified the bioactive properties of honey, propolis, and royal jelly, indicating compounds with antimicrobial, anti-inflammatory, antioxidant activity, anti-tumor and anti-cancer activities (Pasupuleti et al. 2017; Kocot et al. 2018; Easton Calabria et al. 2019). Honey is used to treat stomach ulcers and injuries, increase oral health, fight stomach disorders, and liver and pancreatic diseases, as well as to promote cardiovascular health (Pasupuleti et al. 2017; Easton-Calabria et al. 2019~~) .~~).

Conclusion

Melissopalynology is a useful and complementary tool along with chemical assays to measure the authenticity, fraud, and differentiation between different types of honey. In addition to being a useful tool in the food industry and therapeutic debates, melissopalynology is even more important in the conservation debate. Conducting more extensive studies in the field of melissopalynology in the future to identify the plant species used by bees is of mutual importance in the conservation debate, plant species conservation protects bees and bee conservation leads to plant species conservation. In the field of conservation, more and more

Formatted: Highlight

Formatted: Font color: Red

Formatted: Highlight

Formatted: Highlight

Formatted: Highlight

Formatted: Font color: Red

Formatted: Highlight

ongoing research is needed to better understand the behavior and role of bees and plants. Bee populations are threatened by human activities. Climate change, invasive species, monocultures that have less variety in flower resources, and the use of pesticides have a negative effect on bees. Considering that climate changes affect the feeding behavior of mammals, these changes can affect insects more (Salmanpour et al. 2023). On the other hand, seasonal changes (including changes in temperature, natural light, and precipitation) and changes in vegetation type can not only have a significant impact on the behavior of bees but also on the behavior of other animals (Salmanpour et al. 2021). It is recommended that future studies look at the impact of climate change on honey bees and on the pollen of honey produced by farmed bees be compared to honey produced by wild bees to help better understand the role of melissopalynology in the protection of bees and plant species and the protection of ecosystems.

~~Melissopalynology is a useful and complementary tool along with chemical assays to measure the authenticity, fraud and differentiation between different types of honey. In addition to being a useful tool in the food industry and therapeutic debates, melissopalynology is even more important in the conservation debate. Conducting more extensive studies in the field of melissopalynology in the future to identify the plant species used by bees is of mutual importance in the conservation debate, plant species conservation protects bees and bee conservation leads to plant species conservation. In the field of conservation, more and more ongoing research is needed to better understand the behavior and role of bees and plants. On the other hand, seasonal changes (including changes in temperature, natural light, precipitation) and changes in vegetation type can not only have a significant impact on the behavior of bees but also the behavior of other animals (Salmanpour et al.2021).~~

Formatted: Highlight

~~Recommendation~~

It is recommended that future studies on pollen of honey produced by farmed bees be compared to honey produced by wild bees to help better understand the role of melissopalynology in the protection of bees and plant species and the protection of ecosystems. Bee populations are threatened by human activities. Climate change, invasive species, monocultures that have less variety in flower resources, and the use of pesticides have a negative effect on bees. On the other hand, seasonal changes (including changes in temperature, natural light, precipitation) and changes in vegetation type can not only have a significant impact on the behavior of bees but also the behavior of other animals (Salmanpour et al. 2021). An understanding of ecosystem processes and the importance of the presence of bees and plants to preserve the planet raises the concern that biodiversity loss is directly affecting ecosystem performance.

Funding

This research received no external funding.

Acknowledgments

The authors thank anonymous reviewers for their valuable comments on an earlier draft. We are grateful to Mr. Saeed Javadi Anaghizi is thanked for his assistance in SEM work. We are profoundly grateful to Mr. Mehdi Kia Hairati, Mr. Mohsen Yasari, Mr. Erfan Rajabi, and Mr. Mohammad Reza Kashfi for their valuable help during the fieldwork study.Many thanks to Mr. Saeed Javadi

Formatted: Highlight

Many thanks to Mr. Mehdi Kia Hairati

Many thanks to Mr. Mohsen Yasari

Many thanks to Mr. Erfan Rajabi

~~Many thanks to Mr. Mohammad Reza Kashfi~~

CRediT authorship contribution statement

Writing - Original Draft: Zahra Shakoori

Writing – review & editing: Zahra Shakoori, Ahmadreza Mehrabian, Dariush Minai, Farid Salmanpour, Farzaneh Khajoei Nasab

Conceptualization: Zahra Shakoori, Dariush Minai, Ahmadreza Mehrabian, Farid Salmanpour, Farzaneh Khajoei Nasab

Principal Investigation: Zahra Shakoori

Formal analysis: Zahra Shakoori, Dariush Minai, Ahmadreza Mehrabian, Farid Salmanpour

Sample collection: Zahra Shakoori, Farid Salmanpour

Software: Zahra Shakoori, Farid Salmanpour, Farzaneh Khajoei Nasab

Supervision: Ahmadreza Mehrabian

Methodology: Zahra Shakoori, Dariush Minai, Ahmadreza Mehrabian, Farid Salmanpour

Data curation: Zahra Shakoori

Conflicts of Interest

The authors confirm that they have no conflicts of interest with respect to the work described in this manuscript.

DATA AVAILABILITY STATEMENT

DOI: 10.6084/m9.figshare.22586863

https://figshare.com/s/949e02f3163f39991d4e

Formatted: Line spacing: Double

Formatted: Default Paragraph Font, Font: (Default) +Body (Calibri), 11 pt, Font color: Auto

Formatted: Font color: Red

Statement

In the above study, the beekeepers provided us with honey samples voluntarily and with full consent, and we did not need to obtain a permit due to not entering protected areas or national parks.

Formatted: Font color: Red

References

1. Alijani, B., O'Brien, J. and Yarnal, B., 2008. Spatial analysis of precipitation intensity and concentration in Iran. *Theoretical and Applied Climatology*, *94*(1), pp.107-124. https://doi.org/10.1007/s00704-007-0344-y
2. Alijani, B. and Harman, J.R., 1985. Synoptic climatology of precipitation in Iran. *Annals of the Association of American Geographers*, *75*(3), pp.404-416. https://doi.org/10.1111/j.1467-8306.1985.tb00075.x
3. Aljadi, A.M. and Kamaruddin, M.Y., 2004. Evaluation of the phenolic contents and antioxidant capacities of two Malaysian floral honeys. *Food chemistry*, *85*(4), pp.513-518. https://doi.org/10.1016/S0308-8146(02)00596-4
4. Al-Kiyumi, E.H., Al-Rashdi, B.S., Al-Alawi, A.R., Al-Balushi, A.A., Al-Hooti, S.N., Al-Hosni, S.I., Dhanalekshmi, U.M. and Khan, S.A., 2021. Quantification of bioactive components and evaluation of antioxidative potential of different floral origin honey from arid regions of Oman. *Biocatalysis and Agricultural Biotechnology*, *33*, p.102007.
5. Al-Mamary, M., Al-Meeri, A. and Al-Habori, M., 2002. Antioxidant activities and total phenolics of different types of honey. *Nutrition research*, *22*(9), pp.1041-1047.
6. Al-Naggar, Y. and El-Sofany, A., 2018. Characterization of Apis mellifera Honey of Different Botanical and Geographical Origins in Egypt.

Formatted: Font color: Red, Strikethrough

http://dx.doi.org/10.5455/egysebz.20180523104927

7. Alnaqdy, A., Al-Jabri, A., Al Mahrooqi, Z., Nzeako, B. and Nsanze, H., 2005. Inhibition effect of honey on the adherence of Salmonella to intestinal epithelial cells in vitro. *International journal of food microbiology*, *103*(3), pp.347-351. https://doi.org/10.1016/j.ijfoodmicro.2004.11.042

8. Alvarez-Suarez, J.M. ed., 2017. *Bee products-chemical and biological properties*. Springer. https://doi.org/10.1007/978-3-319-59689-1

9. Alvarez-Suarez, M., J., Giampieri, F., & Battino, M.(2013). Honey as a source of dietary antioxidants: structures, bioavailability and evidence of protective effects against human chronic diseases. Current medicinal chemistry, 20(5), pp.621-638. https://doi.org/10.2174/092986713804999358

10. Alvarez-Suarez, J.M., Tulipani, S., Romandini, S., Bertoli, E. and Battino, M., 2010. Contribution of honey in nutrition and human health: a review. *Mediterranean Journal of Nutrition and Metabolism*, *3*(1), pp.15-23. https://doi.org/10.1007/s12349-009-0051-6

11. Amiri, M.J. and Eslamian, S.S., 2010. Investigation of climate change in Iran. *Journal of Environmental Science and Technology*, *3*(4), pp.208-216.

12. Andrade, P.B., Amaral, M.T., Isabel, P., Carvalho, J.C., Seabra, R.M. and da Cunha, A.P., 1999. Physicochemical attributes and pollen spectrum of Portuguese heather honeys. *Food Chemistry*, *66*(4), pp.503-510. https://doi.org/10.1016/S0308-8146(99)00100-4

13. Barthlott, W., Biedinger, N., Braun, G., Feig, F., Kier, G. and Mutke, J., 1999. Terminological and methodological aspects of the mapping and analysis of global biodiversity. *Acta botanica fennica*, *162*(0), pp.103-110.

14. Barthlott, W., Neinhuis, C., Jetter, R., Bourauel, T. and Riederer, M., 1996. Waterlily, poppy, or sycamore: on the systematic position of Nelumbo. *Flora*, *191*(2), pp.169-174. https://doi.org/10.1016/S0367-2530(17)30709-0

15. Basualdo, C., Sgroy, V., Finola, M.S. and Marioli, J.M., 2007. Comparison of the antibacterial activity of honey from different provenance against bacteria usually isolated from skin wounds. *Veterinary microbiology*, *124*(3-4), pp.375-381. https://doi.org/10.1016/j.vetmic.2007.04.039

16. Behm, F., von der Ohe, K. and Henrich, W., 1996. Zuverlässigkeit der Pollen analyse von Honig. Bestimmung Der Pollenhäufigkeit, Dtsch. Lebensm, 92: 183-187.

17. Belay, A., Haki, G.D., Birringer, M., Borck, H., Addi, A., Baye, K. and Melaku, S., 2017. Rheology and botanical origin of Ethiopian monofloral honey. *LWT*, *75*, pp.393-401. https://doi.org/10.1016/j.lwt.2016.09.021

18. Berberian, M. (1983). The southern Caspian: a compressional depression floored by a trapped, modified oceanic crust. *Canadian Journal of Earth Sciences*, *20*(2), 163-183. https://doi.org/10.1139/e83-015

19. Berberian, M. and King, G.C.P., 1981. Towards a paleogeography and tectonic evolution of Iran. *Canadian journal of earth sciences*, *18*(2), pp.210-265. https://doi.org/10.1139/e81-019

20. Beretta, G., Granata, P., Ferrero, M., Orioli, M. and Facino, R.M., 2005. Standardization of antioxidant properties of honey by a combination of spectrophotometric/fluorimetric assays and chemometrics. *Analytica Chimica Acta*, *533*(2), pp.185-191. https://doi.org/10.1016/j.aca.2004.11.010

21. Berhanu, S., Tadesse, D.M. and Jorge, A., 2022. Physico-chemical properties of Ethiopian Apis mellifera Honey: Review. *J Agric Sc Food Technol*, *8*(1), pp.038-044. https://dx.doi.org/10.17352/2455-815X.000143

22. Bogdanov, S., Jurendic, T., Sieber, R. and Gallmann, P., 2008. Honey for nutrition and health: a review. *Journal of the American college of Nutrition*, *27*(6), pp.677-689. https://doi.org/10.1080/07315724.2008.10719745

23. Bogdanov, S. and Martin, P., 2002. Honey authenticity. *Mitteilungen aus Lebensmitteluntersuchung und Hygiene*, *93*(3), pp.232-254.

24. Bogdanov, S., 1997. Nature and origin of the antibacterial substances in honey. *LWT-Food Science and Technology*, *30*(7), pp.748-753. https://doi.org/10.1006/fstl.1997.0259

25. Bryant Jr, V.M. and Jones, G.D., 2001. The r-values of honey: Pollen coefficients. *Palynology*, *25*(1), pp.11-28. https://doi.org/10.1080/01916122.2001.9989554

26. Buba, F., Gidado, A. and Shugaba, A., 2013. Analysis of biochemical composition of honey samples from North-East Nigeria. *Biochem Anal Biochem*, *2*(3), p.139.

27. Cantarelli, M.A., Pellerano, R.G., Marchevsky, E.J. and Camiña, J.M., 2008. Quality of honey from Argentina: Study of chemical composittion and trace elements. *The Journal of Argentine Chemical Society*, *96*(1-2), pp.33-41.

28. Christy, E.M.L. and Anna, M.C., 2011. An overview of honey: Therapeutic properties and contribution in nutrition and human health. *African Journal of Microbiology Research*, *5*(8), pp.844-852. https://doi.org/10.5897/AJMR10.008

29. Chua, L. S., Rahaman, N. L., Adnan, N. A., & Eddie Tan, T. T. (2013). Antioxidant Activity of Three Honey Samples in relation with Their Biochemical Components. *Journal of analytical methods in chemistry*, *2013*, 313798. https://doi.org/10.1155/2013/313798

30. Cianciosi, D., Forbes-Hernández, T.Y., Afrin, S., Gasparrini, M., Reboredo-Rodriguez, P., Manna, P.P., Zhang, J., Bravo Lamas, L., Martínez Flórez, S., Agudo Toyos, P. and Quiles, J.L., 2018. Phenolic compounds in honey and their associated health benefits: A review. *Molecules*, *23*(9), p.2322. https://doi.org/10.3390/molecules23092322

31. Cooper, R.A., Molan, P.C. and Harding, K.G., 2002. The sensitivity to honey of Gram-positive cocci of clinical significance isolated from wounds. *Journal of Applied microbiology*, *93*(5), pp.857-863. https://doi.org/10.1046/j.1365-2672.2002.01761.x

32. Corvucci, F., Nobili, L., Melucci, D. and Grillenzoni, F.V., 2015. The discrimination of honey origin using melissopalynology and Raman spectroscopy techniques coupled with multivariate analysis. *Food chemistry*, *169*, pp.297-304. https://doi.org/10.1016/j.foodchem.2014.07.122

33. da Silva, P.M., Gauche, C., Gonzaga, L.V., Costa, A.C.O. and Fett, R., 2016. Honey: Chemical composition, stability and authenticity. *Food chemistry*, *196*, pp.309-323. https://doi.org/10.1016/j.foodchem.2015.09.051

34. de Rodríguez, G.O., de Ferrer, B.S., Ferrer, A. and Rodríguez, B., 2004. Characterization of honey produced in Venezuela. *Food Chemistry*, *84*(4), pp.499-502. https://doi.org/10.1016/S0308-8146(02)00517-4

35. Djamali, M., Akhani, H., Khoshravesh, R., Andrieu-Ponel, V., Ponel, P. and Brewer, S., 2011. Application of the global bioclimatic classification to Iran: implications for

understanding the modern vegetation and biogeography. *Ecologia mediterranea*, *37*(1), pp.91-114. https://www.persee.fr/doc/ecmed_0153-8756_2011_num_37_1_1350

36. Easton-Calabria, A., Demary, K.C. and Oner, N.J., 2019. Beyond pollination: honey bees (Apis mellifera) as zootherapy keystone species. *Frontiers in Ecology and Evolution*, *6*, p.161. https://doi.org/10.3389/fevo.2018.00161

37. Efem, S.E.E., 1988. Clinical observations on the wound healing properties of honey. *British journal of Surgery*, *75*(7), pp.679-681.
https://doi.org/10.1002/bjs.1800750718

38. El-Senduny, F.F., Hegazi, N.M., Abd Elghani, G.E. and Farag, M.A., 2021. Manuka honey, a unique mono-floral honey. A comprehensive review of its bioactives, metabolism, action mechanisms, and therapeutic merits. *Food Bioscience*, *42*, p.101038.
https://doi.org/10.1016/j.fbio.2021.101038

39. Escuredo, O., Silva, L.R., Valentão, P., Seijo, M.C. and Andrade, P.B., 2012. Assessing Rubus honey value: Pollen and phenolic compounds content and antibacterial capacity. *Food Chemistry*, *130*(3), pp.671-678.
https://doi.org/10.1016/j.foodchem.2011.07.107

40. Fagih, A.R., Ebadi, R. & Nazarian, H. 2004. Pollen study of flowering plants used by bees in the regions Khansar and Faridan of Isfahan province. Journal of Iran Agriculture Science 35(2): 265-283.
https://eurekamag.com/research/004/024/004024250.php

41. Farhadinia, M.S., Soofi, M., Rosen, T., Moghadas, P., Hobeali, K., Behnoud, P., Amanov, A., Dieterich, T., Hojamuradov, H., Hudaikuliev, N., Karryeva, s., Kaczensky, P., Mengliev, s., Muhashov, A., Nurmuhambetov, Z., Pestov, M., Potaeva, A., Terentyev, V., Salmanpour,

F., Tizrouyan, H., Alinezhad, H., Jafari, B., Ommatmohammadi, M., Rabie, K., Veyisov, A., Linnell, J., 2022. Status of Persian leopards in northern Iran and Central Asia. CATnews Special. https://www.researchgate.net/publication/362889852

42. Finola, M.S., Lasagno, M.C. and Marioli, J.M., 2007. Microbiological and chemical characterization of honeys from central Argentina. *Food chemistry*, *100*(4), pp.1649-1653. https://doi.org/10.1016/j.foodchem.2005.12.046

43. Fredes, C. and Montenegro, G., 2006. Heavy metal and other trace elements contents in honey bee in Chile. Cien. Inv. Agr.(in English) 33 (1): 50-58. *International Journal of Agriculture and Natural Resources*, *33*(1), pp.50-58. https://revistaschilenas.uchile.cl/handle/2250/10.7764/rcia.v33i1.328

44. García, N.L., 2018. The current situation on the international honey market. *Bee World*, *95*(3), pp.89-94. https://doi.org/10.1080/0005772X.2018.1483814

45. Hailu, D. and Belay, A., 2020. Melissopalynology and antioxidant properties used to differentiate Schefflera abyssinica and polyfloral honey. *Plos one*, *15*(10), p.e0240868. https://doi.org/10.1371/journal.pone.0240868

46. Hedge, I. C., & Wendelbo, P. (1978). Patterns of distribution and endemism in Iran. *Notes Roy. Bot. Gard. Edinburgh*, *36*(2), 441-464. https://www.scopus.com/record/display.uri?eid=2-s2.0-0006003564&origin=inward&txGid=08c1677fbb653bcda02974e2f9bd74ed

47. Noroozi J, Körner C. A bioclimatic characterization of high elevation habitats in the Alborz mountains of Iran. Alpine botany. 2018 Apr;128:1-1. https://doi.org/10.1007/s00035-018-0202-9

48. Jones, G.D. and Bryant Jr, V.M., 1996. New Frontiers in Palynology. *Palynology: Principles and Applications, edited by J. Jansonius and DC McGregor*, pp.933-38. https://scholar.google.com/scholar?hl=en&as_sdt=0%2C5&q=New+Frontiers+in+Palynology.+Palynology%3A+Principles+and+Applications%2C+edited+by+J.+Jansonius+and+DC+McGregor.&btnG=

49. Johnson, D.W., van Eps, C., Mudge, D.W., Wiggins, K.J., Armstrong, K., Hawley, C.M., Campbell, S.B., Isbel, N.M., Nimmo, G.R. and Gibbs, H., 2005. Randomized, controlled trial of topical exit-site application of honey (Medihoney) versus mupirocin for the prevention of catheter-associated infections in hemodialysis patients. *Journal of the American Society of Nephrology*, *16*(5), pp.1456-1462. https://doi.org/10.1681/ASN.2004110997

50. Karabagias, I.K., Badeka, A., Kontakos, S., Karabournioti, S. and Kontominas, M.G., 2014. Characterisation and classification of Greek pine honeys according to their geographical origin based on volatiles, physicochemical parameters and chemometrics. *Food chemistry*, *146*, pp.548-557. https://doi.org/10.1016/j.foodchem.2013.09.105

51. Karimi, A., Nazarian, H., Jafari, E. and Hatami, A., 2016. Identification and determine the distribution of plants used by bees in Galehdar watershed (Fars province). *Natural Ecosystems of Iran*, *7*(3), pp.13-27. https://neijournal.nour.iau.ir/mobile/article_527674.html?lang=en

52. Kaškonienė, V. and Venskutonis, P.R., 2010. Floral markers in honey of various botanical and geographic origins: a review. *Comprehensive reviews in food science and food safety*, *9*(6), pp.620-634. https://doi.org/10.1111/j.1541-4337.2010.00130.x

53. Kečkeš, S., Gašić, U., Veličković, T.Ć., Milojković-Opsenica, D., Natić, M. and Tešić, Ž., 2013. The determination of phenolic profiles of Serbian unifloral honeys using ultra-high-performance liquid chromatography/high resolution accurate mass spectrometry. *Food Chemistry*, *138*(1), pp.32-40. https://doi.org/10.1016/j.foodchem.2012.10.025

54. Kędzierska-Matysek, M., Stryjecka, M., Teter, A., Skałecki, P., Domaradzki, P. and Florek, M., 2021. Relationships between the content of phenolic compounds and the antioxidant activity of polish honey varieties as a tool for botanical discrimination. *Molecules*, *26*(6), p.1810. https://doi.org/10.3390/molecules26061810

55. Khalil, M.I., Moniruzzaman, M., Boukraâ, L., Benhanifia, M., Islam, M.A., Islam, M.N., Sulaiman, S.A. and Gan, S.H., 2012. Physicochemical and antioxidant properties of Algerian honey. *Molecules*, *17*(9), pp.11199-11215. https://doi.org/10.3390/molecules170911199

56. Khansaritoreh, E., Salmaki, Y., Azirani, T.A., Henareh, F., Alizadeh, K., Ramezani, E., Zarre, S., Beckh, G. and Behling, H., 2021. The sources and quality of Iranian honey. *Heliyon*, *7*(4), p.e06651. https://doi.org/10.1016/j.heliyon.2021.e06651

57. Kier, G., Mutke, J., Dinerstein, E., Ricketts, T.H., Küper, W., Kreft, H. and Barthlott, W., 2005. Global patterns of plant diversity and floristic knowledge. *Journal of Biogeography*, *32*(7), pp.1107-1116. https://doi.org/10.1111/j.1365-2699.2005.01272.x

58. Klein, J.C. and Lacoste, A., 1989. The oak groves of *Quercus macranthera* F. and M. in the Alborz mountain (Iran) and the eastern extremities of the Great and Little Caucasus. *Ecologia Mediterranea (France)*.

https://scholar.google.com/scholar?hl=en&as_sdt=0%2C5&q=%5BThe+oak+groves+of+Quercus+macranthera+F.+and+M.+in+the+Alborz+mountain+%28Iran%29+and+the+eastern+extremities+of+the+Great+and+Little+Caucasus&btnG=

59. Klein, B. and Leffler, K.B., 1981. The role of market forces in assuring contractual performance. *Journal of political Economy*, *89*(4), pp.615-641. https://doi.org/10.1086/260996

60. Kocot, J., Kiełczykowska, M., Luchowska-Kocot, D., Kurzepa, J. and Musik, I., 2018. Antioxidant potential of propolis, bee pollen, and royal jelly: possible medical application. *Oxidative medicine and cellular longevity*, *2018*. https://doi.org/10.1155/2018/7074209

61. Kortesniemi, M., Rosenvald, S., Laaksonen, O., Vanag, A., Ollikka, T., Vene, K. and Yang, B., 2018. Sensory and chemical profiles of Finnish honeys of different botanical origins and consumer preferences. *Food Chemistry*, *246*, pp.351-359. https://doi.org/10.1016/j.foodchem.2017.10.069

62. Kuldasheva, F., Madartov, B., Nurmurodova, N. and Eshdavlatov, O., 2022. STUDY OF ORGANOLEPTIC, PHYSICAL AND CHEMICAL INDICATORS OF HONEY IN TASHKENT REGION. *Agro Inform*, (1), pp.43-51. https://cyberleninka.ru/article/n/study-of-organoleptic-physical-and-chemical-indicators-of-honey-in-tashkent-region

63. Louveaux, J., Maurizio, A., & Vorwohl, G. (1978). Methods of melissopalynology. Bee world, 59(4), 139-157. https://doi.org/10.1080/0005772X.1978.11097714

64. Louveaux, J. and Maurizio, A., G. vorwohl. 1970. Methods of melissopalynology. *Bee world*, *51*, pp.25-138. https://doi.org/10.1080/0005772X.1970.11097312

65. Machado De-Melo, A.A., Almeida-Muradian, L.B.D., Sancho, M.T. and Pascual-Maté, A., 2018. Composition and properties of Apis mellifera honey: A review. *Journal of apicultural research*, *57*(1), pp.5-37. https://doi.org/10.1080/00218839.2017.1338444

66. Manzanares, A.B., García, Z.H., Galdón, B.R., Rodríguez-Rodríguez, E.M. and Romero, C.D., 2017. Physicochemical characteristics and pollen spectrum of monofloral honeys from Tenerife, Spain. *Food chemistry*, *228*, pp.441-446. https://doi.org/10.1016/j.foodchem.2017.01.150

67. Martos, I., Ferreres, F., Yao, L., D'Arcy, B., Caffin, N. and Tomás-Barberán, F.A., 2000. Flavonoids in monospecific Eucalyptus honeys from Australia. *Journal of Agricultural and Food Chemistry*, *48*(10), pp.4744-4748. https://doi.org/10.1021/jf000277i

68. Maurizio, A., 1975. Microscopy of honey. *Honey: A Comprehensive Survey. E. Crane, ed*. https://scholar.google.com/scholar?hl=en&as_sdt=0%2C5&q=Maurizio%2C+A.+1975.+Microscopy+of+honey.+%E2%80%93+In%3A+Honey%3A+&btnG=

69. Mehrabian, A. and Rad, M.A., 2021. Distribution patterns and priorities for conservation of Iranian Endemic Monocots: determining the Areas of Endemism (AOEs). *Journal of Wildlife and Biodiversity*, *5*(2), pp.69-87. https://doi.org/10.22120/jwb.2020.136616.1188

70. Memariani, F. & Ghoraishi Alhossein, J. 2000. Pollen study of some samples of honey in Khorasan province. Pajohesh va Sazandegi 49: 76-83.

71. Menad, H., Ben-Naoum, F. and Amine, A., 2020. A Hybrid Grey Wolves Optimizer and Convolutional Neural Network for Pollen Grain Recognition. *International Journal of Swarm Intelligence Research (IJSIR)*, *11*(3), pp.49-71.

https://doi.org/10.4018/IJSIR.2020070104

72. Molan, P., 2001. Why honey is effective as a medicine: 2. The scientific explanation of its effects. *Bee world*, *82*(1), pp.22-40. https://doi.org/10.1080/0005772X.2001.11099498

73. Mureșan, C.I., Cornea-Cipcigan, M., Suharoschi, R., Erler, S. and Mărgăoan, R., 2022. Honey botanical origin and honey-specific protein pattern: Characterization of some European honeys. *LWT*, *154*, p.112883. https://doi.org/10.1016/j.lwt.2021.112883

74. Naqinezhad, A. and Esmailpoor, A., 2017. Flora and vegetation of rocky outcrops/cliffs near the Hyrcanian forest timberline in the Mazandaran mountains, northern Iran. *Nordic Journal of Botany*, *35*(4), pp.449-466. https://doi.org/10.1111/njb.01384

75. Nanda, V., Sarkar, B.C., Sharma, H.K. and Bawa, A.S., 2003. Physico-chemical properties and estimation of mineral content in honey produced from different plants in Northern India. *Journal of Food Composition and Analysis*, *16*(5), pp.613-619. https://doi.org/10.1016/S0889-1575(03)00062-0

76. Nayik, G.A. and Nanda, V., 2016. Application of response surface methodology to study the combined effect of temperature, time and pH on antioxidant activity of cherry (Prunus avium) honey. *Polish Journal of Food and Nutrition Sciences*, *66*(4), pp.287-293. https://doi.org/10.1515/pjfns-2015-0055

77. Nazarian, H., Sanei Shariat Panahi, M. & tahmasebi, G.H. 1997. Determination of Identification of plants used by bees in Tehran province. The second Conference on Honey bee. Iran Animal Science Research Institute.

78. Noroozi, J. ed., 2020. *Plant biogeography and vegetation of high mountains of Central and South-West Asia*. Springer International Publishing. https://doi.org/10.1007/978-3-030-45212-4

79. Noroozi, J., Pauli, H., Grabherr, G. and Breckle, S.W., 2011. The subnival–nival vascular plant species of Iran: a unique high-mountain flora and its threat from climate warming. *Biodiversity and Conservation*, *20*(6), pp.1319-1338. https://doi.org/10.1007/s10531-011-0029-9

80. Olivieri, C., Marota, I., Rollo, F. and Luciani, S., 2012. Tracking plant, fungal, and bacterial DNA in honey specimens. *Journal of Forensic Sciences*, *57*(1), pp.222-227. https://doi.org/10.1111/j.1556-4029.2011.01964.x

81. Ouchemoukh, S., Louaileche, H. and Schweitzer, P., 2007. Physicochemical characteristics and pollen spectrum of some Algerian honeys. *Food control*, *18*(1), pp.52-58. https://doi.org/10.1016/j.foodcont.2005.08.007

82. Ozturk, F.; Erkan, C. Bee plants of Van Lake Basin (Turkey). *Int. J. Bot.* **2010**, *6*, 101–106. https://www.cabdirect.org/cabdirect/abstract/20103306252

83. Pasupuleti, V.R., Sammugam, L., Ramesh, N. and Gan, S.H., 2017. Honey, propolis, and royal jelly: a comprehensive review of their biological actions and health benefits. *Oxidative medicine and cellular longevity*, *2017*. https://doi.org/10.1155/2017/1259510

84. Ponnuchamy, R., Bonhomme, V., Prasad, S., Das, L., Patel, P., Gaucherel, C., Pragasam, A. and Anupama, K., 2014. Honey pollen: using melissopalynology to understand foraging preferences of bees in tropical South India. *PLoS One*, *9*(7), p.e101618. https://doi.org/10.1371/journal.pone.0101618

85. Puusepp, L. and Koff, T., 2014. Pollen analysis of honey from the Baltic region, Estonia. *Grana*, *53*(1), pp.54-61. https://doi.org/10.1080/00173134.2013.850532

86. Rao, P.V., Krishnan, K.T., Salleh, N. and Gan, S.H., 2016. Biological and therapeutic effects of honey produced by honey bees and stingless bees: a comparative review. *Revista Brasileira de Farmacognosia*, *26*, pp.657-664. https://doi.org/10.1016/j.bjp.2016.01.012

87. Rašić, S., Štefanić, E., Antunović, S., Jović, J. and Kristek, S., 2018. Pollen analysis of honey from north-eastern Croatia. *Poljoprivreda*, *24*(2), pp.43-49. https://doi.org/10.18047/poljo.24.2.6

88. Razzaghy Kamroudi Mehdi, Sanei Shariat Panahi Mohammad, Nazarian Hassan, Qelichnia Hassan. Identification of pollens in honeys of Nourrud watershed of Mazandaran. Research and construction. 2006 [cited 2022May14]; 19 (3 (p. 72) on livestock and aquaculture): 74-83.

89. Ronsisvalle, S., Lissandrello, E., Fuochi, V., Petronio Petronio, G., Straquadanio, C., Crascì, L., Panico, A., Milito, M., Cova, A.M., Tempera, G. and Furneri, P.M., 2019. Antioxidant and antimicrobial properties of Casteanea sativa Miller chestnut honey produced on Mount Etna (Sicily). *Natural product research*, *33*(6), pp.843-850. https://doi.org/10.1080/14786419.2017.1413568

90. Safi Ahmad Abad, M., Shirvany, A., Panahi, P. (2017). 'Comparison of pollen grain composition and quality of honey obtained from forest and rangeland sites in Arasbaran region', *Iranian Journal of Forest and Poplar Research*, 25(2), pp. 342-353. doi: 10.22092/ijfpr.2017.111783 https://dx.doi.org/10.22092/ijfpr.2017.111783

91. Salmanpour, F., Shakoori, Z., Kia, M. and Ghomi, S., 2021. Investigating the Factors Affecting Leopard (Panthera pardus tulliana) Conflict with Domestic Livestock Compared to other Large Carnivores in Nowshahr Hyrcanian Forests. *Journal of Animal Environment*, *13*(2), pp.1-8. doi: 10.22034/AEJ.2021.135105 https://www.aejournal.ir/article_135105.html?lang=en

92. Sanei Shariat Panahi, M. & Said Abadi, V. 1974. Nectriferous plants in Karaj based on palynology. Journal of Agricultural Faculty, 2-3: 35-47.

93. Saxena, S., Gautam, S. and Sharma, A., 2010. Physical, biochemical and antioxidant properties of some Indian honeys. *Food chemistry*, *118*(2), pp.391-397. https://doi.org/10.1016/j.foodchem.2009.05.001

94. Selvaraju, K., Vikram, P., Soon, J.M., Krishnan, K.T. and Mohammed, A., 2019. Melissopalynological, physicochemical and antioxidant properties of honey from West Coast of Malaysia. *Journal of food science and technology*, *56*(5), pp.2508-2521. https://doi.org/10.1007/s13197-019-03728-3

95. Shakoori, Z., Mehrabian, A., Salmanpour, F., 2022. Evaluation of plant origin of honey based on pollen evidence in some prominent honey-producing zones in Iran, 47th Apimondia Congress (0355), pp-124Ref.0355. https://www.researchgate.net/publication/363134174

96. Stocklin, J., 1977. Structural correlation of the Alpine ranges between Iran and Central Asia.https://pascal-francis.inist.fr/vibad/index.php?action=getRecordDetail&idt=PASCALGEODEBRGM7720435256

97. Stöcklin, J., 1974. Northern iran: Alborz mountains. *Geological Society, London, Special Publications*, *4*(1), pp.213-234. https://doi.org/10.1144/GSL.SP.2005.004.01.12

98. Subrahmanyam, M., 1998. A prospective randomised clinical and histological study of superficial burn wound healing with honey and silver sulfadiazine. *Burns*, *24*(2), pp.157-161. https://doi.org/10.1016/S0305-4179(97)00113-7

99. Tucak, Z.; Tucak, A.; Puškadija, Z.; Tucak, M. Nutritious healing composition of some kinds of honey in Eastern Croatia. *Agriculture* **2000**, *6*, 129–132.

100. Vaghefi, S.A., Keykhai, M., Jahanbakhshi, F., Sheikholeslami, J., Ahmadi, A., Yang, H. and Abbaspour, K.C., 2019. The future of extreme climate in Iran. *Scientific reports*, *9*(1), pp.1-11. https://doi.org/10.1038/s41598-018-38071-8

101. Von Der Ohe, W., Oddo, L.P., Piana, M.L., Morlot, M. and Martin, P., 2004. Harmonized methods of melissopalynology. *Apidologie*, *35*(Suppl. 1), pp.S18-S25. DOI: 10.1051/apido:2004050 https://www.apidologie.org/articles/apido/pdf/2004/06/MHS07.pdf

102. Yao, L., Datta, N., Tomás-Barberán, F.A., Ferreres, F., Martos, I. and Singanusong, R., 2003. Flavonoids, phenolic acids and abscisic acid in Australian and New Zealand Leptospermum honeys. *Food Chemistry*, *81*(2), pp.159-168. https://doi.org/10.1016/S0308-8146(02)00388-6

Books:

102. Davis SD, Heywood VH, Hamilton AC. Centres of plant diversity. Natural History. 1994;111(1):1-0.

103. Dewan, M.L. and Famouri, J., 1964. *The soils of Iran*. Food and Agriculture Organization of the United Nations.

104. Ghelich Nia, H. 2017. Vegetation types of Mazandaran. Research Institute of Forests and Rangelands.

105. Fayaz, M. 2015. Vegetation types of Tehran. Research Institute of Forests and Rangelands.

106. Stöcklin, J., 1968. Salt deposits of the Middle East.

107. Takhtajan, A. 1986: Floristic regions of the world. - Berkeley: University of California Press.Volume 7, Issue 4, April 2021, e06651.

108. Zahran, M.A., 2010. *Climate-Vegetation:: Afro-Asian Mediterranean and Red Sea Coastal Lands* (Vol. 4). Springer Science & Business Media.

109. Zimmerman, M.I.C.H.A.E.L., 1988. Nectar production, flowering phenology, and strategies for pollination. *Plant reproductive ecology: patterns and strategies*, *41*, pp.157-178.

110. Zohary, M., 1973. *Geobotanical foundations of the Middle East*. Fischer.

Thesis:

111. Dewan ML, Famouri J. *The Soils of Iran, FAO, Rome* (Doctoral dissertation, Thesis presented to the)1963.

112. Klein R. *The new politics of the National Health Service* (Doctoral dissertation, Texas Tech University),1972.

Website:

113. UNESCO (United Nations Educational Scientific and Cultural Organization). 2019. https://www.whc.unesco. org/en/list/1584. Hyrcanian Forests.

114. FAOSTAT, 2018. Production of Natural Honey in World. http://www.fao.org/faostat/en/#data/QL/visualize

1. Aljadi AM, Kamaruddin MY. Evaluation of the phenolic contents and antioxidant capacities of two Malaysian floral honeys. Food chemistry. 2004 May 1;85(4):513-8. https://doi.org/10.1016/S0308-8146(02)00596-4
2. Al Kiyumi EH, Al Rashdi BS, Al Alawi AR, Al Balushi AA, Al Hooti SN, Al Hosni SI, Dhanalekshmi UM, Khan SA. Quantification of bioactive components and evaluation of antioxidative potential of different floral origin honey from arid regions of Oman. Biocatalysis and Agricultural Biotechnology. 2021 May 1;33:102007.
3. Al-Mamary M, Al-Meeri A, Al-Habori M. Antioxidant activities and total phenolics of different types of honey. Nutrition research. 2002 Sep 1;22(9):1041-7. https://doi.org/10.1016/S0271-5317(02)00406-2
4. Al Naggar Y, El-Sofany A, Naiem E, Seif A. Characterization of Apis mellifera honey of different botanical and geographical origins in Egypt. The Egyptian Journal of Experimental Biology (Zoology). 2018;14(1):75-84. http://dx.doi.org/10.5455/egysebz.20180523104927
5. Alnaqdy A, Al-Jabri A, Al Mahrooqi Z, Nzeako B, Nsanze H. Inhibition effect of honey on the adherence of Salmonella to intestinal epithelial cells in vitro. International journal of food microbiology. 2005 Sep 15;103(3):347-51. https://doi.org/10.1016/j.ijfoodmicro.2004.11.042
6. Alvarez-Suarez JM, editor. Bee products-chemical and biological properties. Springer; 2017 Sep 5. https://doi.org/10.1007/978-3-319-59689-1
7. M Alvarez-Suarez J, Giampieri F, Battino M. Honey as a source of dietary antioxidants: structures, bioavailability and evidence of protective effects against human chronic diseases. Current medicinal chemistry. 2013 Feb 1;20(5):621-38. https://doi.org/10.2174/092986713804999358
8. Alvarez-Suarez JM, Tulipani S, Romandini S, Bertoli E, Battino M. Contribution of honey in nutrition and human health: a review. Mediterranean Journal of Nutrition and Metabolism. 2010 Jan 1;3(1):15-23. https://doi.org/10.1007/s12349-009-0051-6
9. Athipornchai A, Klangmanee K. A rapid determination of the effective antioxidant agents using their Fe (III) complexes. Arabian Journal of Chemistry. 2021 Mar 1;14(3):102986. https://doi.org/10.1016/j.arabjc.2021.102986
10. Barthlott W, Biedinger N, Braun G, Feig F, Kier G, Mutke J. Terminological and methodological aspects of the mapping and analysis of global biodiversity. Acta botanica fennica. 1999;162(0):103-10. https://www.researchgate.net/profile/Jens-Mutke/publication/215672839_Terminological_and_methodological_aspects_of_the_mapping_and_analysis_of_global_biodiversity/links/57c5338208aecd4514157c0b/Terminological-and-methodological-aspects-of-the-mapping-and-analysis-of-global-biodiversity.pdf
11. Barthlott W, Neinhuis C, Jetter R, Bourauel T, Riederer M. Waterlily, poppy, or sycamore: on the systematic position of Nelumbo. Flora. 1996 Apr 1;191(2):169-74. https://doi.org/10.1016/S0367-2530(17)30709-0

12. Basualdo C, Sgroy V, Finola MS, Marioli JM. Comparison of the antibacterial activity of honey from different provenance against bacteria usually isolated from skin wounds. Veterinary microbiology. 2007 Oct 6;124(3-4):375-81. https://doi.org/10.1016/j.vetmic.2007.04.039
13. Behm F, Von Der Ohe K, Henrich W. Zuverlässigkeit der Pollenanalyse von Honig: bestimmung der Pollenhäufigkeit. Deutsche Lebensmittel-Rundschau. 1996;92(6):183-8. https://pascal-francis.inist.fr/vibad/index.php?action=getRecordDetail&idt=3123198
14. Berberian M, King GC. Towards a paleogeography and tectonic evolution of Iran: Reply. Canadian Journal of Earth Sciences. 1981 Nov 1;18(11):1764-6. https://doi.org/10.1139/e81-019
15. Beretta G, Granata P, Ferrero M, Orioli M, Facino RM. Standardization of antioxidant properties of honey by a combination of spectrophotometric/fluorimetric assays and chemometrics. Analytica Chimica Acta. 2005 Mar 28;533(2):185-91. https://doi.org/10.1016/j.aca.2004.11.010
16. Berhanu S, Tadesse DM, Jorge A. Physico-chemical properties of Ethiopian Apis mellifera Honey: Review. J Agric Sc Food Technol. 2022;8(1):038-44. https://dx.doi.org/10.17352/2455-815X.000143
17. Bogdanov S, Jurendic T, Sieber R, Gallmann P. Honey for nutrition and health: a review. Journal of the American college of Nutrition. 2008 Dec 1;27(6):677-89. https://doi.org/10.1080/07315724.2008.10719745
18. Bogdanov S. Nature and origin of the antibacterial substances in honey. LWT-Food Science and Technology. 1997 Nov 1;30(7):748-53. https://doi.org/10.1006/fstl.1997.0259
19. Bryant Jr VM, Jones GD. The R-values of honey: pollen coefficients. Palynology. 2001 Jan 1;25(1):11-28. https://doi.org/10.1080/01916122.2001.9989554
20. Buba F, Gidado A, Shugaba A. Analysis of biochemical composition of honey samples from North-East Nigeria. Biochem Anal Biochem. 2013;2(3):139. DOI: 10.4172/2161-1009.1000139
21. Cantarelli MA, Pellerano RG, Marchevsky EJ, Camiña JM. Quality of honey from Argentina: Study of chemical composittion and trace elements. The Journal of Argentine Chemical Society. 2008 Dec;96(1-2):33-41. https://doi.org/10.5897/AJMR10.008
22. Chua LS, Rahaman NL, Adnan NA, Eddie Tan TT. Antioxidant activity of three honey samples in relation with their biochemical components. Journal of analytical methods in chemistry. 2013 Jan 1;2013. https://doi.org/10.1155/2013/313798
23. Cianciosi D, Forbes-Hernández TY, Afrin S, Gasparrini M, Reboredo-Rodriguez P, Manna PP, Zhang J, Bravo Lamas L, Martínez Flórez S, Agudo Toyos P, Quiles JL. Phenolic compounds in honey and their associated health benefits: A review. Molecules. 2018 Sep 11;23(9):2322. https://doi.org/10.3390/molecules23092322
24. Cooper RA, Molan PC, Harding KG. The sensitivity to honey of Gram-positive cocci of clinical significance isolated from wounds. Journal of Applied microbiology. 2002 Nov 1;93(5):857-63. https://doi.org/10.1046/j.1365-2672.2002.01761.x

25. Corvucci F, Nobili L, Melucci D, Grillenzoni FV. The discrimination of honey origin using melissopalynology and Raman spectroscopy techniques coupled with multivariate analysis. Food chemistry. 2015 Feb 15;169:297-304. https://doi.org/10.1016/j.foodchem.2014.07.122
26. da Silva PM, Gauche C, Gonzaga LV, Costa AC, Fett R. Honey: Chemical composition, stability and authenticity. Food chemistry. 2016 Apr 1;196:309-23. https://doi.org/10.1016/j.foodchem.2015.09.051
27. Davis SD, Heywood VH, Hamilton AC. Centres of plant diversity. Natural History. 1994;111(1):1-0. http://www.nativefishlab.net/library/textpdf/18340.pdf
28. de Rodríguez GO, de Ferrer BS, Ferrer A, Rodríguez B. Characterization of honey produced in Venezuela. Food Chemistry. 2004 Mar 1;84(4):499-502. https://doi.org/10.1016/S0308-8146(02)00517-4
29. Dewan ML, Famouri J. The soils of Iran. 1964. FAO, Rome.
30. Easton-Calabria A, Demary KC, Oner NJ. Beyond pollination: honey bees (Apis mellifera) as zootherapy keystone species. Frontiers in Ecology and Evolution. 2019 Feb 1;6:161. https://doi.org/10.3389/fevo.2018.00161
31. Efem SE. Clinical observations on the wound healing properties of honey. British journal of Surgery. 1988 Jul;75(7):679-81. https://doi.org/10.1002/bjs.1800750718
32. Escuredo O, Silva LR, Valentão P, Seijo MC, Andrade PB. Assessing Rubus honey value: Pollen and phenolic compounds content and antibacterial capacity. Food Chemistry. 2012 Feb 1;130(3):671-8.https://doi.org/10.1016/j.foodchem.2011.07.107
33. Faghihi AR, Ebadi R, Nazarian H. Study of pollen plants used by honey bees (Apis mellifera L.) in Khansar and Faridan regions of Isfahan province with sub stepic climate. Iranian Journal of Agricultural Sciences. 2004;35(2):265-83.https://eurekamag.com/research/004/024/004024250.php
34. Farhadinia, M.S., Soofi, M., Rosen, T., Moghadas, P., Hobeali, K., Behnoud, P., Amanov, A., Dieterich, T., Hojamuradov, H., Hudaikuliev, N., Karryeva, s., Kaczensky, P., Mengliev, s., Muhashov, A., Nurmuhambetov, Z., Pestov, M., Potaeva, A., Terentyev, V., Salmanpour, F., Tizrouyan, H., Alinezhad, H., Jafari, B., Ommatmohammadi, M., Rabie, K., Veyisov, A., Linnell, J., 2022. Status of Persian leopards in northern Iran and Central Asia. CATnews Special. https://www.researchgate.net/publication/362889852
35. FAOSTAT, 2018. Production of Natural Honey in World. http://www.fao.org/faostat/en/#data/QL/visualize
36. Fayaz, M. 2015. Vegetation types of Tehran. Research Institute of Forests and Rangelands.
37. Finola MS, Lasagno MC, Marioli JM. Microbiological and chemical characterization of honeys from central Argentina. Food chemistry. 2007 Jan 1;100(4):1649-53. https://doi.org/10.1016/j.foodchem.2005.12.046
38. Fredes C, Montenegro G. Heavy metal and other trace elements contents in honey bee in Chile. Cien. Inv. Agr.(in English) 33 (1): 50-58. International Journal of Agriculture and Natural Resources. 2006 Jan 1;33(1):50-8.

https://revistaschilenas.uchile.cl/handle/2250/10.7764/rcia.v33i1.328

39. Ghelich Nia, H. 2017. Vegetation types of Mazandaran. Research Institute of Forests and Rangelands.

40. Hammer Ø, Harper DA, Ryan PD. PAST: Paleontological statistics software package for education and data analysis. Palaeontologia electronica. 2001 Jun 22;4(1):9. https://paleo.carleton.ca/2001_1/past/past.pdf
41. Hedge IC, Wendelbo P. Patterns of distribution and endemism in Iran. 1978. https://www.scopus.com/record/display.uri?eid=2-s2.0-0006003564&origin=inward&txGid=08c1677fbb653bcda02974e2f9bd74ed
42. Jones GD, Bryant Jr VM. New frontiers in Palynology. Palynology: Principles and Applications, edited by J. Jansonius and DC McGregor. 1996:933-38. https://scholar.google.com/scholar?hl=en&as_sdt=0%2C5&q=New+Frontiers+in+Palynology.+Palynology%3A+Principles+and+Applications%2C+edited+by+J.+Jansonius+and+DC+McGregor.&btnG=
43. Johnson DW, van Eps C, Mudge DW, Wiggins KJ, Armstrong K, Hawley CM, Campbell SB, Isbel NM, Nimmo GR, Gibbs H. Randomized, controlled trial of topical exit-site application of honey (Medihoney) versus mupirocin for the prevention of catheter-associated infections in hemodialysis patients. Journal of the American Society of Nephrology. 2005 May 1;16(5):1456-62. https://doi.org/10.1681/ASN.2004110997
44. Karabagias IK, Badeka A, Kontakos S, Karabournioti S, Kontominas MG. Characterisation and classification of Greek pine honeys according to their geographical origin based on volatiles, physicochemical parameters and chemometrics. Food chemistry. 2014 Mar 1;146:548-57. https://doi.org/10.1016/j.foodchem.2013.09.105
45. Karimi A, Nazarian H, Jafari E, Hatami A. Identification and determine the distribution of plants used by bees in Galehdar watershed (Fars province). Natural Ecosystems of Iran. 2016 Nov 21;7(3):13-27. https://neijournal.nour.iau.ir/mobile/article_527674.html?lang=en
46. Kaškonienė V, Venskutonis PR. Floral markers in honey of various botanical and geographic origins: a review. Comprehensive reviews in food science and food safety. 2010 Nov;9(6):620-34. https://doi.org/10.1111/j.1541-4337.2010.00130.x
47. Kečkeš S, Gašić U, Veličković TĆ, Milojković-Opsenica D, Natić M, Tešić Ž. The determination of phenolic profiles of Serbian unifloral honeys using ultra-high-performance liquid chromatography/high resolution accurate mass spectrometry. Food Chemistry. 2013 May 1;138(1):32-40. https://doi.org/10.1016/j.foodchem.2012.10.025
48. Kędzierska-Matysek M, Stryjecka M, Teter A, Skałecki P, Domaradzki P, Florek M. Relationships between the content of phenolic compounds and the antioxidant activity of Polish honey varieties as a tool for botanical discrimination. Molecules. 2021 Mar 23;26(6):1810. https://doi.org/10.3390/molecules26061810

49. Khalil MI, Moniruzzaman M, Boukraâ L, Benhanifia M, Islam MA, Islam MN, Sulaiman SA, Gan SH. Physicochemical and antioxidant properties of Algerian honey. Molecules. 2012 Sep 20;17(9):11199-215. https://doi.org/10.3390/molecules170911199
50. Khansaritoreh E, Salmaki Y, Azirani TA, Henareh F, Alizadeh K, Ramezani E, Zarre S, Beckh G, Behling H. The sources and quality of Iranian honey. Heliyon. 2021 Apr 1;7(4):e06651. https://doi.org/10.1016/j.heliyon.2021.e06651
51. Kier G, Mutke J, Dinerstein E, Ricketts TH, Küper W, Kreft H, Barthlott W. Global patterns of plant diversity and floristic knowledge. Journal of Biogeography. 2005 Jul;32(7):1107-16. https://doi.org/10.1111/j.1365-2699.2005.01272.x
52. Klein JC, Lacoste A. The oak groves of Quercus macranthera F. and M. in the Alborz mountain (Iran) and the eastern extremities of the Great and Little Caucasus. Ecologia Mediterranea (France). 1989. https://scholar.google.com/scholar?hl=en&as_sdt=0%2C5&q=%5BThe+oak+groves+of+Quercus+macranthera+F.+and+M.+in+the+Alborz+mountain+%28Iran%29+and+the+eastern+extremities+of+the+Great+and+Little+Caucasus&btnG=
53. Klein B, Leffler KB. The role of market forces in assuring contractual performance. Journal of political Economy. 1981 Aug 1;89(4):615-41. https://doi.org/10.1086/260996
54. Klein R. The new politics of the National Health Service (Doctoral dissertation, Texas Tech University).
55. Kocot J, Kiełczykowska M, Luchowska-Kocot D, Kurzepa J, Musik I. Antioxidant potential of propolis, bee pollen, and royal jelly: possible medical application. Oxidative medicine and cellular longevity. 2018 May 2;2018. https://doi.org/10.1155/2018/7074209
56. Kortesniemi M, Rosenvald S, Laaksonen O, Vanag A, Ollikka T, Vene K, Yang B. Sensory and chemical profiles of Finnish honeys of different botanical origins and consumer preferences. Food Chemistry. 2018 Apr 25;246:351-9. https://doi.org/10.1016/j.foodchem.2017.10.069
57. Kuldasheva F, Madartov B, Nurmurodova N, Eshdavlatov O. STUDY OF ORGANOLEPTIC, PHYSICAL AND CHEMICAL INDICATORS OF HONEY IN TASHKENT REGION. Agro Inform. 2022(1):43-51.https://cyberleninka.ru/article/n/study-of-organoleptic-physical-and-chemical-indicators-of-honey-in-tashkent-region
58. Louveaux J, Maurizio A, Vorwohl G. Methods of melissopalynology. Bee world. 1978 Jan 1;59(4):139-57.https://doi.org/10.1080/0005772X.1978.11097714
59. Louveaux J, Maurizio A, Vorwohl G. Methods of melissopa-lynology. Bee World. 1970 51:25–138https://doi.org/10.1080/0005772X.1970.11097312
60. Machado De-Melo AA, Almeida-Muradian LB, Sancho MT, Pascual-Maté A. Composition and properties of Apis mellifera honey: A review. Journal of apicultural research. 2018 Jan 1;57(1):5-37.https://doi.org/10.1080/00218839.2017.1338444

61. Martos I, Ferreres F, Tomás-Barberán FA. Identification of flavonoid markers for the botanical origin of Eucalyptus honey. Journal of Agricultural and Food Chemistry. 2000 May 15;48(5):1498-502.https://doi.org/10.1021/jf000277i
62. Maurizio A. Microscopy of honey. Honey: A Comprehensive Survey. E. Crane, ed. 1975.https://scholar.google.com/scholar?hl=en&as_sdt=0%2C5&q=Maurizio%2C+A.+1975.+Microscopy+of+honey.+%E2%80%93+In%3A+Honey%3A+&btnG=
63. Mehrabian A, Rad MA. Distribution patterns and priorities for conservation of Iranian Endemic Monocots: determining the Areas of Endemism (AOEs). Journal of Wildlife and Biodiversity. 2021 Mar 30;5(2):69-87.https://doi.org/10.22120/jwb.2020.136616.1188
64. Memariani F. A pollen study of some honey samples in Khorasan province. Journal of Research and Construction. 2000;17(4):49-83.
65. Menad H, Ben-Naoum F, Amine A. A hybrid grey wolves optimizer and convolutional neural network for pollen grain recognition. International Journal of Swarm Intelligence Research (IJSIR). 2020 Jul 1;11(3):49-71.https://doi.org/10.4018/IJSIR.2020070104
66. Molan P. Why honey is effective as a medicine: 2. The scientific explanation of its effects. Bee world. 2001 Jan 1;82(1):22-40.https://doi.org/10.1080/0005772X.2001.11099498
67. Nanda V, Sarkar BC, Sharma HK, Bawa AS. Physico-chemical properties and estimation of mineral content in honey produced from different plants in Northern India. Journal of Food Composition and Analysis. 2003 Oct 1;16(5):613-9. https://doi.org/10.1016/S0889-1575(03)00062-0
68. Nayik GA, Nanda V. Application of response surface methodology to study the combined effect of temperature, time and pH on antioxidant activity of cherry (Prunus avium) honey. Polish Journal of Food and Nutrition Sciences. 2016 Dec 31;66(4):287-93. https://doi.org/10.1515/pjfns-2015-0055
69. Nazarian, H., Sanei Shariat Panahi, M. & tahmasebi, G.H. 1997. Determination of Identification of plants used by bees in Tehran province. The second Conference on Honey bee. Iran Animal Science Research Institute.
70. Noroozi J, Körner C. A bioclimatic characterization of high elevation habitats in the Alborz mountains of Iran. Alpine botany. 2018 Apr;128:1-1. https://doi.org/10.1007/s00035-018-0202-9
71. Noroozi J, Pauli H, Grabherr G, Breckle SW. The subnival–nival vascular plant species of Iran: a unique high-mountain flora and its threat from climate warming. Biodiversity and Conservation. 2011 Jun;20:1319-38.https://doi.org/10.1007/s10531-011-0029-9
72. Ouchemoukh S, Louaileche H, Schweitzer P. Physicochemical characteristics and pollen spectrum of some Algerian honeys. Food control. 2007 Jan 1;18(1):52-8. https://doi.org/10.1016/j.foodcont.2005.08.007
73. Ozturk F, Erkan C. Bee plants of Van Lake Basin (Turkey). International Journal of Botany. 2010;6(2):101-6. https://www.cabdirect.org/cabdirect/abstract/20103306252

74. Pasupuleti VR, Sammugam L, Ramesh N, Gan SH. Honey, propolis, and royal jelly: a comprehensive review of their biological actions and health benefits. Oxidative medicine and cellular longevity. 2017 Oct;2017. https://doi.org/10.1155/2017/1259510
75. Ponnuchamy R, Bonhomme V, Prasad S, Das L, Patel P, Gaucherel C, Pragasam A, Anupama K. Honey pollen: using melissopalynology to understand foraging preferences of bees in tropical South India. PLoS One. 2014 Jul 8;9(7):e101618. https://doi.org/10.1371/journal.pone.0101618
76. Puusepp L, Koff T. Pollen analysis of honey from the Baltic region, Estonia. Grana. 2014 Jan 2;53(1):54-61. https://doi.org/10.1080/00173134.2013.850532
77. Rao PV, Krishnan KT, Salleh N, Gan SH. Biological and therapeutic effects of honey produced by honey bees and stingless bees: a comparative review. Revista Brasileira de Farmacognosia. 2016 Sep;26:657-64.https://doi.org/10.1016/j.bjp.2016.01.012
78. Rašić S, Štefanić E, Antunović S, Jović J, Kristek S. Pollen analysis of honey from north-eastern Croatia. Poljoprivreda. 2018 Dec 19;24(2):43-9.https://doi.org/10.18047/poljo.24.2.6
79. Razzaghy Kamroudi M, Sanei Shariat Panahi M, Nazarian H, Qelichnia Hassan. Identification of pollens in honeys of Nourrud watershed of Mazandaran. Research and construction. 2006 [cited 2022May14]; 19 (3 (p. 72) on livestock and aquaculture): 74-83.
80. Safi Ahmad Abad M, Shirvany A, Panahi P. Comparison of pollen grain composition and quality of honey obtained from forest and rangeland sites in Arasbaran region. Iranian Journal of Forest and Poplar Research. 2017 Jun 22;25(2):342-53. https://dx.doi.org/10.22092/ijfpr.2017.111783
81. Salmanpour F, Shakoori Z, Kia M, Eshaghi R, Ghaderi M, Ghomi S, Kaveh R, Rabie K, Kiabi BH, Farhadinia MS. Mineral lick use by a community of large herbivores in northern Iran. Ecology and Evolution. 2023 Jan;13(1):e9731. https://doi.org/10.1002/ece3.9731
82. Salmanpour F, Shakoori Z, Kia M, Ghomi S. Investigating the Factors Affecting Leopard (Panthera pardus tulliana) Conflict with Domestic Livestock Compared to other Large Carnivores in Nowshahr Hyrcanian Forests. Journal of Animal Environment. 2021 Jul 23;13(2):1-8. https://www.aejournal.ir/article_135105.html?lang=en
83. Sanei Shariat Panahi, M, & Said Abadi, V. Nectriferous plants in Karaj based on palynology. Journal of Agricultural Faculty. 1974; 2-3: 35-47.
84. Saxena S, Gautam S, Sharma A. Physical, biochemical and antioxidant properties of some Indian honeys. Food chemistry. 2010 Jan 15;118(2):391-7. https://doi.org/10.1016/j.foodchem.2009.05.001
85. Selvaraju K, Vikram P, Soon JM, Krishnan KT, Mohammed A. Melissopalynological, physicochemical and antioxidant properties of honey from West Coast of Malaysia. Journal of food science and technology. 2019 May 1;56:2508-21. https://doi.org/10.1007/s13197-019-03728-3
86. Shakoori Z, Mehrabian A, Salmanpour F. Evaluation of plant origin of honey based on pollen evidence in some prominent honey-producing zones in Iran, 47th Apimondia Congress (0355). 2022; pp-124Ref.0355.

https://www.researchgate.net/publication/363134174

87. Stöcklin J. Salt deposits of the Middle East. Geological Society of America; 1968 Jan. https://books.google.com/books?hl=en&lr=&id=uwGmwnjjM5YC&oi=fnd&pg=PA157&dq=87.%09St%C3%B6cklin,+J.,+1968.+Salt+deposits+of+the+Middle+East.+&ots=QRqRrTvKhb&sig=BDdWzNrkq8YwaQG9lrqFcse05No#v=onepage&q&f=false
88. Subrahmanyam M. A prospective randomised clinical and histological study of superficial burn wound healing with honey and silver sulfadiazine. Burns. 1998 Mar 1;24(2):157-61. https://doi.org/10.1016/S0305-4179(97)00113-7
89. Takhtajan AL. Floristic regions of the world. Berkeley University of California Press. 54 B. Djavadi & S. M Ghaffari. 1986.
90. Tucak Z, Tucak A, Puskadija Z, Tucak M. Nutritions-healing structure of some kinds Honey in eastern Croatia. In8 th International Symposium Animal science days, Osijek 2000 (pp. 129-131). https://www.researchgate.net/profile/Martin-Verstegen/publication/40140722_Ileal_digestibility_of_amino_acids_in_pig_feeds_and_its_use_in_diet_formulations/links/53db7c780cf2631430cb5153/Ileal-digestibility-of-amino-acids-in-pig-feeds-and-its-use-in-diet-formulations.pdf?_sg%5B0%5D=started_experiment_milestone&origin=journalDetail&_rtd=e30%3D#page=129
91. Von Der Ohe W, Oddo LP, Piana ML, Morlot M, Martin P. Harmonized methods of melissopalynology. Apidologie. 2004;35(Suppl. 1):S18-25. DOI: 10.1051/apido:2004050 https://www.apidologie.org/articles/apido/pdf/2004/06/MHS07.pdf
92. Yao L, Datta N, Tomás-Barberán FA, Ferreres F, Martos I, Singanusong R. Flavonoids, phenolic acids and abscisic acid in Australian and New Zealand Leptospermum honeys. Food Chemistry. 2003 May 1;81(2):159-68. https://doi.org/10.1016/S0308-8146(02)00388-6
93. Zahran MA. Climate-Vegetation:: Afro-Asian Mediterranean and Red Sea Coastal Lands. Springer Science & Business Media; 2010 Mar 10. https://books.google.com/books?id=rRIBe5OyF5MC&printsec=frontcover#v=onepage&q&f=false
94. Zimmerman MI. Nectar production, flowering phenology, and strategies for pollination. Plant reproductive ecology: patterns and strategies. 1988 Jul 7;41:157-78. https://books.google.com/books?id=Yjv1woP0QtYC&printsec=frontcover#v=onepage&q&f=false
95. Zohary M. Geobotanical foundations of the Middle East. Fischer; 1973. https://ixtheo.de/Record/1154164403

Dear Dr. Daniel de Paiva Silva

Thank you for providing us with the opportunity to submit a revised draft of the manuscript PONE-D-22-31247 entitled " Assessing the Quality of Bee Honey on the basis of melissopalynology as well chemical analysis ". We appreciate the time and effort that you and the reviewers have dedicated to providing valuable feedback on our manuscript. We are grateful to the reviewers for their insightful comments on the paper. We have been able to incorporate changes to reflect all the suggestions provided and all changes are highlighted in the manuscript text file.

Please see below a point-by-point response to the reviewers' comments and concerns.

Best wishes

Ahmadreza Mehrabian

Dear Editor

- **Comments to the Author:**

After this first review round, all three reviewers raised significant issues that prevent the publication of your manuscript at this time. Still, the reviewers indicated that the manuscript has a potential to be published in PLoS One after improvements are considered.

Response: We appreciate the positive comments of the reviewers and have done our best to incorporate all comments, with specific responses below.

If applicable, we recommend that you deposit your laboratory protocols in protocols.io to enhance the reproducibility of your results. Protocols.io assigns your protocol its own identifier (DOI) so that it can be cited independently in the future.

Response: We saved our data in the figshare repository and placed the link in the text of the manuscript.

DATA AVAILABILITY STATEMENT
DOI: 10.6084/m9.figshare.22586863
https://figshare.com/s/949e02f3163f39991d4e

- **Journal requirements:**

1. Please ensure that your manuscript meets PLOS ONE's style requirements, including those for file naming. The PLOS ONE style templates can be found at
https://journals.plos.org/plosone/s/file?id=wjVg/PLOSOne_formatting_sample_main_body.pdf
and https://journals.plos.org/plosone/s/file?id=ba62/PLOSOne_formatting_sample_title_authors_affiliations.pdf.

Response: We prepared the manuscript with PLOS ONE style requirements, including file naming.

2. We noticed you have some minor occurrence of overlapping text with the following previous publication(s), which needs to be addressed:

Plant Biogeography and Vegetation of High Mountains of Central and South-West Asia - https://link.springer.com/book/10.1007/978-3-030-45212-4, and

A bioclimatic characterization of high elevation habitats in the Alborz mountains of Iran - https://doi.org/10.1007/s00035-018-0202-9.

In your revision ensure you cite all your sources (including your own works), and quote or rephrase any duplicated text outside the methods section. Further consideration is dependent on these concerns being addressed.

Response: We added "A bioclimatic characterization of high elevation habitats in the Alborz mountains of Iran - https://doi.org/10.1007/s00035-018-0202-9" in our study area section:

The Alborz mountain range is located at 36° N from the Caspian Sea up to a height of 5671 meters (Noroozi et al.2018).

3. In your Methods section, please provide additional information regarding the permits you obtained for the work. Please ensure you have included the full name of the authority that approved the field site access and, if no permits were required, a brief statement explaining why.

Response: In the above study, we did not need a permit because the honey samples were provided to us voluntarily by the beekeepers, and because there was no need to enter protected areas or national parks, we did not need to obtain a permit.

Statement: In the above study, the beekeepers provided us with honey samples voluntarily and with full consent, and we did not need to obtain a permit due to not entering protected areas or national parks.

4. Please update your submission to use the PLOS LaTeX template. The template and more information on our requirements for LaTeX submissions can be found at http://journals.plos.org/plosone/s/latex.

Response: Corrections were made.

5. We note that you have stated that you will provide repository information for your data at acceptance. Should your manuscript be accepted for publication, we will hold it until you provide the relevant accession numbers or DOIs necessary to access your data. If you wish to make changes to your Data Availability statement, please describe these changes in your cover letter and we will update your Data Availability statement to reflect the information you provide.

Response: We saved our laboratory protocols and data in the figshare repository and placed the link in the text of the manuscript.

DATA AVAILABILITY STATEMENT

DOI: 10.6084/m9.figshare.22586863

https://figshare.com/s/949e02f3163f39991d4e

6. PLOS requires an ORCID iD for the corresponding author in Editorial Manager on papers submitted after December 6th, 2016. Please ensure that you have an ORCID iD and that it is validated in Editorial Manager. To do this, go to 'Update my Information' (in the upper left-hand corner of the main menu), and click on the Fetch/Validate link next to the ORCID field. This will take you to the ORCID site and allow you to create a new iD or authenticate a pre-existing iD in Editorial Manager. Please see the following video for instructions on linking an ORCID iD to your Editorial Manager account: https://www.youtube.com/watch?v=_xcclfuvtxQ.

Response: The ORCID of each author was declared.

Zahra Shakoori: https://orcid.org/0000-0003-3057-8981

Ahmadreza Mehrabian: https://orcid.org/0000-0001-6633-3092

Dariush Minai: https://orcid.org/0000-0003-3589-7324

Farid Salmanpour: https://orcid.org/0000-0002-1517-9061

Farzaneh Khajoei Nasab: https://orcid.org/0000-0002-2325-9555

- **Dear Prof. Ing. Jaromir Lachman, Reviewer #1:**

To the article, I have next comments and recommendations:

• In the article, there are many typing errors, which should be carefully checked and corrected, e.g., in Abstract: you should use 0.08 to 0.05 ppm or 0.08-0.05 and so on throughout the text. Like wise L. 47:…climate of the region…; L. 61:…botanical…;, Table 1: Bignoniaceae L. 98: instead antioxidative should be used antioxidant activity or antioxidant capacity and similar in the all article.

Response: We tried to carefully check and correct typographical errors. For example, we used 0.08 to 0.05 pp throughout the text. Instead of antioxidant, we used antioxidant activity in the article. Other mentioned points such as amendment in L. 46:...climate of the region...; L. 58:…botanical…;, Table 1: Bignoniaceae done.

• Latin names of plants should be in Italics through all text and References, e.g., L. 140: Quercus macranthera, etc.

Response: The Latin names of the plants were written in italics in all texts and references.

• All applied equipment and chemicals should be carefully completed with the name of the producer, town and country (state) – check and complete the missing date especially in section Laboratory studies.

Response: The name of the manufacturer, city and country (state) of all equipment and chemicals used were carefully entered with the name of the manufacturer, especially in the laboratory studies section.

- L.212: Hot plate model HP100 made by Mtops company in South Korea
- L.216: Centrifuge model TDL-4 made by Jenius China
- L.247: Merck Chemical Company in Germany.
- L.248: 2,2-Diphenyl-1-picrylhydrazyl (DPPH) was obtained from Sigma American Chemical.
- 258: JENUS V-1100 spectrophotometer Made by DLAB China

- In the centrifugation convert rpm to g values and correct centrifugation instead centrifuge(s), where it is appropriate.

Response: In the centrifuge, we converted revolutions per minute to g values and corrected centrifugation instead of centrifuge where appropriate.

- L. 637: 1% ferric chloride…

  Response: The item mentioned in L. 637 was not found, but it was found in line 264 and corrected.

- Unify Apocynaceae & Apiaceae or Apocynaceae and Apiaceae, similarly throughout the text.

  Response: Apocynaceae and Apiaceae are similarly united throughout the text.

- L. 294 and elsewhere: total phenolic content or total phenolics.

  Response: Throughout the text, the phenol compounds and total phenol were changed to total phenolic content.

- Some sentences are completely confusing and should be corrected, e.g. L. 336-337:…in the Estonia (Puusepp and Koff 2014). Croatia (?). Our results show…

  Response: Confusing sentences were completely corrected throughout the text.

- The main problem is that the citation system should be substantially redone according to system used in PLOS One, i.e., using brackets like [1□, [2□,[3□, etc. and hence the references they will not be in alphabetical order.

  Response: In this version, we have adjusted the entire manuscript according to PLOS One guidelines.

- According to this system you should change the order of references. In References you should apply Italics in Latin names of plants (Prunus avium, Schefflera abyssinica) and bees

(Apis mellifera), use the full titles of Journals with capitals of nouns and adjectives, like Food Chemistry, Nutrition Research, International Journal of Food Microbiology, Current Medicinal Chemistry, LWT-Food Science and Technology, etc., do not use Italics in titles of journals (not mixture). Do not use capitals in the name of article – should be e.g., Study of organoleptic, physical, and chemical indicators of honey in Tashkent region. Do not use pp. and p. in references, by the way some articles in new volumes use an article number (one number) instead of pages.

Response: We modified and prepared all the references according to the instructions of the journal and all the mentioned points were applied.

- Books, Theses and Website should be implemented along journals referenced with their citation number.

Here you are some examples:

1. Hailu D, Belay A. Melissopalynology and antioxidant properties used to differentiate Schefflera abyssinica and polyfloral honey. PLOS One. 2020;15(10): e0240868.https://doi.org/10.1371/journal.pone.0240868

2. Alvarez-Suarez MJ., Giampieri F, Battino M. Honey as a source of dietary antioxidants: structures, bioavailability and evidence of protective effects against human chronic diseases. Current Medicinal Chemistry. 2013; 20(5), 621-638. https://doi.org/10.2174/092986713804999358

3. Berberian M.The southern Caspian: a compressional depression floored by a trapped, modified oceanic crust. Canadian Journal of Earth Sciences. 1983; 20(2): 163-183. https://doi.org/10.1139/e83-015

4. Chua LS, Rahaman NL, Adnan NA, Eddie Tan, TT. Antioxidant activity of three honey samples in relation with their biochemical components. Journal of Analytical Methods in Chemistry. 2013: 313798. https://doi.org/10.1155/2013/313798

5. El-Senduny FF, Hegazi NM, Abd Elghani GE, Farag MA. Manuka honey, a unique mono-floral honey. A comprehensive review of its bioactives. metabolism, action mechanisms, and therapeutic merits. Food Bioscience. 2021; 42:101038. https://doi.org/10.1016/j.fbio.2021.101038

6. Hedge IC, Wendelbo P. Patterns of distribution and endemism in Iran. Notes from the Royal Botanic Garden, Edinburgh. 1978; 36(2): 441-464. https://www.scopus.com/record/display.uri?eid=2-s2.0-0006003564&origin=inward&txGid=08c1677fbb653bcda02974e2f9bd74ed

7. Kędzierska-Matysek M, Stryjecka M, Teter A, Skałecki P, Domaradzki P, Florek M. 2021. Relationships between the content of phenolic compounds and the antioxidant activity of Polish honey varieties as a tool for botanical discrimination. Molecules. 2021; 26(6): 1810. https://doi.org/10.3390/molecules26061

Response: We have made the mentioned corrections completely.

- **Dear Reviewer #2:**

1. Authors stated in line 105 that “the study tries to analyze the mentioned data in relation to geographical factors.” and they try to analyze this aspect in the section 5. Discussion. However, for drawing conclusions if chemical parameters of honey depend on geographical factors, the proper statistical analysis is needed.

   Response: This section has been completely revised.

2. The sentence “We compared the humidity of honey samples with the humidity of the areas from which the samples were collected, which shows that the humidity of the environment affected the humidity of honey samples” must be proven by the statistical analysis.

   Response: We removed the map related to the effect of ambient humidity on the humidity of honey samples and using the minitab software, we got regression fitted line plots statistical model.

   The regression result between the average annual relative humidity of the region (independent variable) and the humidity of the honey sample (dependent variable) showed that there is a significant relationship (p value 0.000) between them, so that the humidity of honey samples is a function of the humidity of the environment.

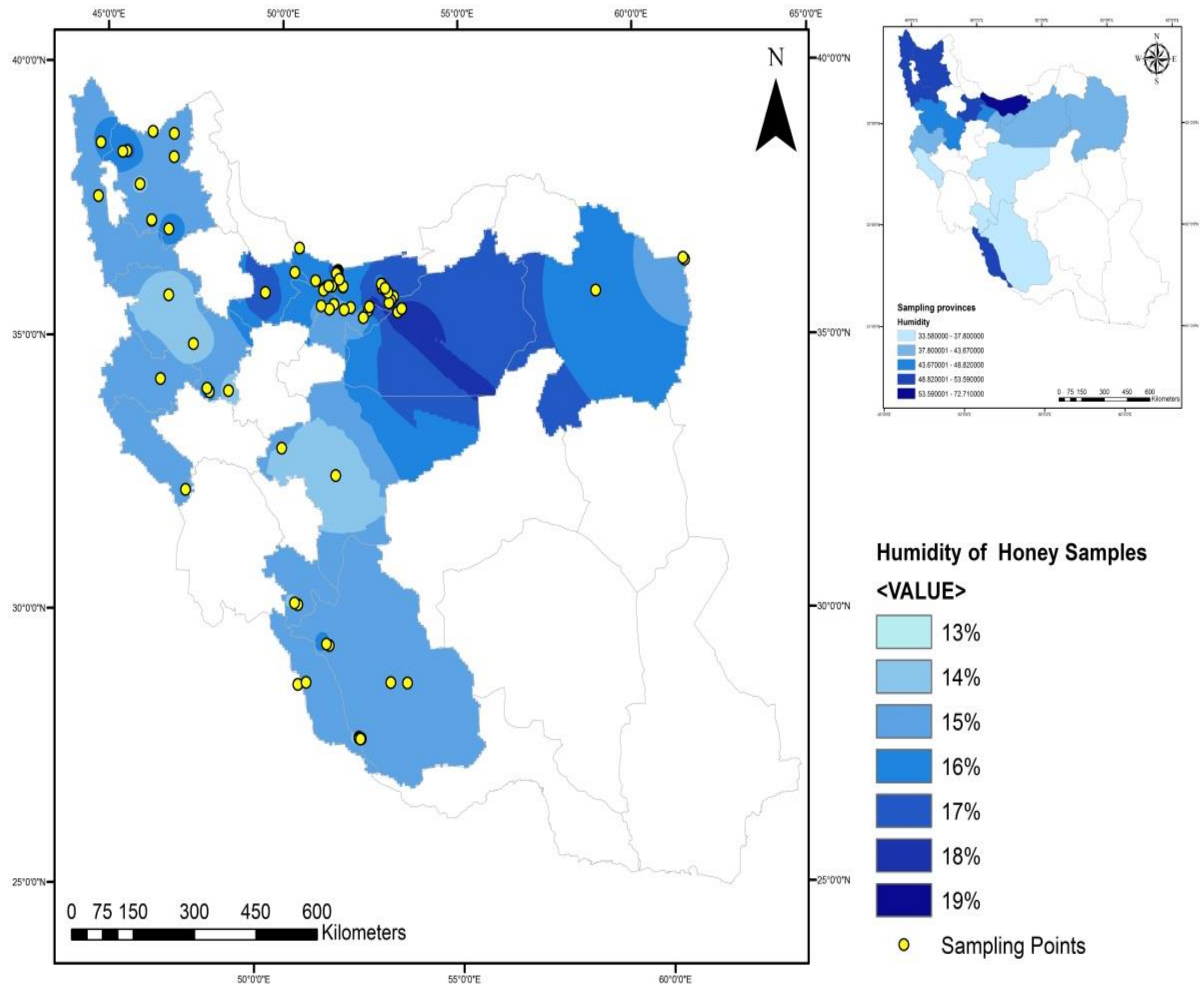

Fig 5. Comparison of moisture content of honey samples with moisture content of the collection site

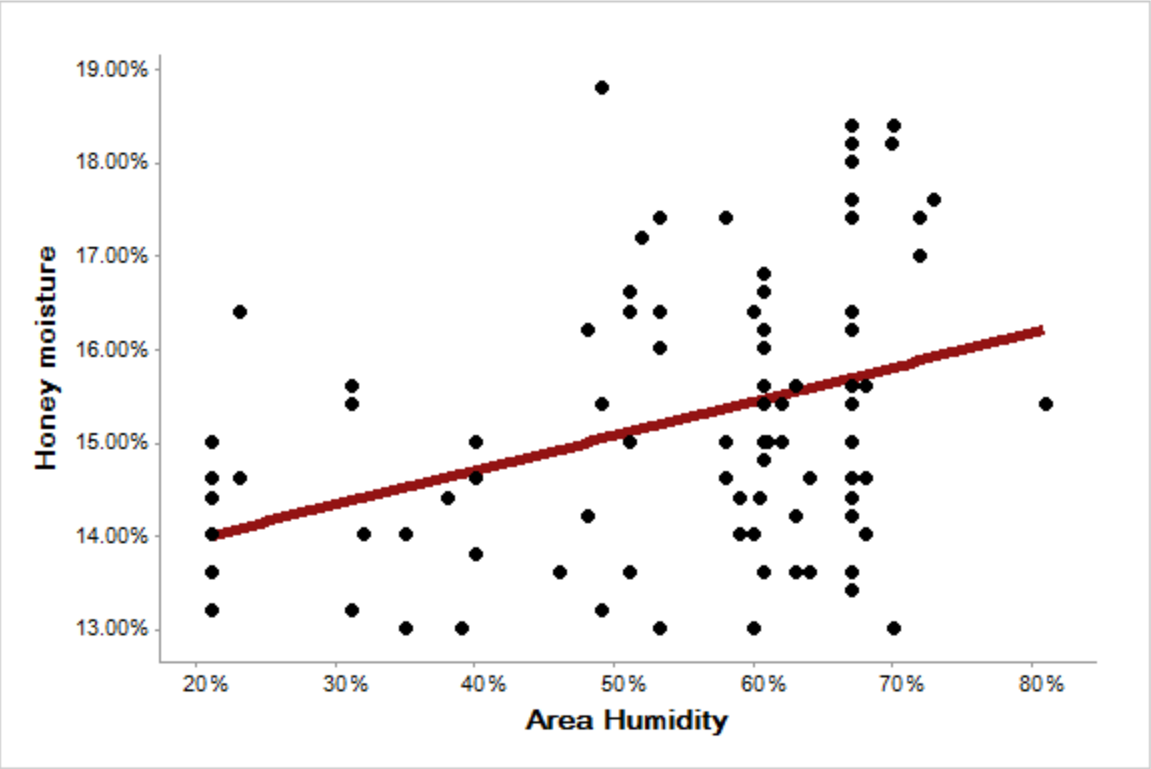

Fig 5. Regression related to the effect of environmental humidity on the moisture of honey samples

3. On Figure. 6 the font size on the axis descriptions must be increased for greater readability. It is also no clear for me, what is the Loading. Do Authors mean coefficient of correlation?
   Response: In this section, more detailed reforms were needed, and these reforms were carried out.
   Yes, loading means the same coefficient of correlation that was modified in the figure.

4. The sentence in lines 299-302 should be corrected. It is completely incomprehensible.

   Response: In this section, more detailed reforms were needed, and these reforms were carried out.

5. Figure 8 – Authors should inform where the reader can find the explanation of codes of samples. On this picture Authors depict results of cluster analysis but I can't find in the text how these results are used, or which conclusions were drawn base on them.
   Response: We saved our data in the figshare repository and placed the link in the text of the manuscript, codes can be found in it.
   DOI: 10.6084/m9.figshare.22586863

https://figshare.com/s/949e02f3163f39991d4e
In addition, this section was modified in the text of the manuscript and some information was included in the text of the manuscript.

6. Text must be linguistically and editorially corrected.
   Response: The text was linguistically and editorially corrected.

Dear Dr. Haya i. Aljohar, Reviewer #3

- Introduction
- The introduction consists of many staccato sentences, forcing the author to use an infinite number of linking phrases, such as: as well as , Furthermore, in addition, Therefore etc (Rewrite the entire introduction in proper English).
  Response: We rewrote the entire introduction in proper English.

  - Line 49 remove(the)
    Response: It was corrected.

  - Line 51 use counterfeit instead of fake
    Response: Counterfeit replaced fake.

  - Line 54 use and instead of as well as and change has to have.
    Response: It was corrected

  - Line 56 (Louveaux (1970, 1978)) Add reference
    Response: Line59: Reference added (Louveaux et al. 1970; 1978)

  - Line 59- 65 Rewrite this paragraph (There are many separate sentences that must be linked together and rewritten in proper English).
    Response: We have rewritten this paragraph in proper English.

  - Line 73 (SW Asia) It was mentioned here for the first time, so it must be written without abbreviation.
    Response: Line 78: Southwest Asia is written instead of SW Asia

  - Rewrite the paragraph from line 69-78.
    Response: The text of the manuscript was completely rewritten and revised

- Study area
- Rewrite in a proper English

  Response: All the text of the manuscript was rewritten in proper English.

- Field studies Change to sample collection

  Response: Field studies Changed to sample collection

  - Line 179 What is the meaning of amount of hight.

    Response: We mean the same height.

    Line 197: The amount of hight was changed to height

- Laboratory studies

  - It is better to put a paragraph for the chemicals and reagents used and another paragraph for the devices and tools used in the experiment separately.

    Response: We have completely revised this paragraph and tried to separate the content as much as possible in the same paragraph to make it clearer.

  - Rewrite in a proper English.

    Response: All the text of the manuscript was rewritten in proper English.

  - Line 192 Then, remove large …. By passed through

    Response: corrections were made

  - Line 223 DPPH abbreviation for what? It was mentioned here for the first time, so it must be written without abbreviation.

    Response: Line 248: DPPH was written in full.

    2,2-Diphenyl-1-picrylhydrazyl (DPPH) was obtained from Sigma American Chemical Company.

- Line 237(1%) Repeated twice in the sentence why?

  Response: $FeCl_3$ 1% is the abbreviation of 1% ferric chloride solution, that's why 1% is repeated twice in the sentence.

  Line 264: The sentence was modified in this way: In this experiment, 1% ferric chloride solution ($FeCl_3$ 1%)...

- Results

- Rewrite scientifically in a proper English

  Response: It was scientifically rewritten in appropriate English

- Add a table containing honey samples names, symbols, and geographical areas

  Response: We have added the requested information and stored it in the figshare repository as backup information.

  DATA AVAILABILITY STATEMENT
  DOI: 10.6084/m9.figshare.22586863
  https://figshare.com/s/949e02f3163f39991d4e

- Line 273 Was in the range instead of was the lowest.

  Response: Line 339: It was corrected.

- Figure 6 Not clear

  Response: Response: In this section, more detailed reforms were needed, and these reforms were carried out.

- Line 299-304 This part is incomprehensible and needs reformulation

  Response: Line 424 – 427: The paragraph has been completely revised.
  The PCA did not result in any significant differences between all honey samples studied (Fig. 9). Considering that the honey samples studied were collected from different geographical areas, samples from the same area were not placed next to each other in the plot. This shows that honey samples from different locations in the same area have different chemical compositions.

- Unify the method of writing references within the article

  Response: Unify the references in the article were prepared as based on the journal's guidelines.

- Line 348 Rephrasing

  Response: Rewriting done
