# Supplementary material for: Assessing the quality of bee honey on the basis of melissopalynology as well as chemical analysis
Source: PLoS One. 2023 Aug 8;18(8):e0289702. doi: 10.1371/journal.pone.0289702 (PMC10409281; doi:10.1371/journal.pone.0289702)
Supplement: Attachment — Submitted filename: Review of PONE-D-22-31247.docx [file pone.0289702.s001.docx]

**Review of PONE-D-22-31247**

In the article, melissopalynology and chemical analysis to evaluate 22 different honey samples from two main Iranian hubs of honey. Sampling was carried out at two 23 important poles of Iranian honey production, the central Alborz region, and the mountain ecosystems of Zagros in the years 2020-2021. results showed that all honey samples were classified as polyfloral honey. Based on their findings, 57 honey samples (61%) contained the standard amount of pollen. A total of 42 plant families and 55 genera were identified in the studied samples, with 32 the highest presence of Asteraceae, Fabaceae, Rosaceae, Apiaceae, and Apocynaceae.

To the article, I have next comments and recommendations:

- In the article, there are many typing errors, which should be carefully checked and corrected, e.g., in Abstract: you should use 0.08 to 0.05 ppm or 0.08-0.05 and so on throughout the text. Like wise L. 47:…climate of the region…; L. 61:…botanical…;, Table 1: Bignoniaceae L. 98: instead antioxidative should be used antioxidant activity or antioxidant capacity and similar in the all article.
- Latin names of plants should be in Italics through all text and References, e.g., L. 140: *Quercus macranthera*, etc.
- All applied equipment and chemicals should be carefully completed with the name of the producer, town and country (state) – check and complete the missing date especially in section Laboratory studies.
- In the centrifugation convert rpm to g values and correct centrifugation instead centrifuge(s), where it is appropriate.
- L. 637: 1% ferric chloride…
- Unify Apocynaceae & Apiaceae or Apocynaceae and Apiaceae, similarly throughout the text.
- L. 294 and elsewhere: total phenolic content or total phenolics.
- Figure 6: should be modified, the data on axis x are nearly not readable.
- Some sentences are completely confusing and should be corrected, e.g. L. 336-337:…in the Estonia (Puusepp and Koff 2014). Croatia (?). Our results show…
- The main problem is that the citation system should be substantially redone according to system used in PLOS One, i.e., using brackets like [1], [2],[3], etc. and hence the references they will not be in alphabetical order.
- According to this system you should change the order of references. In References you should apply Italics in Latin names of plants (*Prunus avium, Schefflera abyssinica*) and bees (*Apis mellifera*), use the full titles of Journals with capitals of nouns and adjectives, like Food Chemistry, Nutrition Research, International Journal of Food Microbiology, Current Medicinal Chemistry, LWT-Food Science and Technology, etc., do not use Italics in titles of journals (not mixture). Do not use capitals in the name of article – should be e.g., Study of organoleptic, physical, and chemical indicators of honey in Tashkent region. Do not use pp. and p. in references, by the way some articles in new volumes use an article number (one number) instead of pages.
- Books, Theses and Website should be implemented along journals referenced with their citation number.

**Here you are some examples:**

1. Hailu D, Belay A. Melissopalynology and antioxidant properties used to differentiate *Schefflera abyssinica* and polyfloral honey. PLOS One. 2020;15(10): e0240868.<https://doi.org/10.1371/journal.pone.0240868>
2. Alvarez-Suarez MJ., Giampieri F, Battino M. Honey as a source of dietary antioxidants: structures, bioavailability and evidence of protective effects against human chronic diseases. Current Medicinal Chemistry. 2013; 20(5), 621-638. <https://doi.org/10.2174/092986713804999358>
3. Berberian M.The southern Caspian: a compressional depression floored by a trapped, modified oceanic crust. Canadian Journal of Earth Sciences. 1983; 20(2): 163-183. <https://doi.org/10.1139/e83-015>
4. Chua LS, Rahaman NL, Adnan NA, Eddie Tan, TT. Antioxidant activity of three honey samples in relation with their biochemical components. Journal of Analytical Methods in Chemistry. 2013: 313798. <https://doi.org/10.1155/2013/313798>
5. El-Senduny FF, Hegazi NM, Abd Elghani GE, Farag MA. Manuka honey, a unique mono-floral honey. A comprehensive review of its bioactives. metabolism, action mechanisms, and therapeutic merits. Food Bioscience. 2021; 42:101038. <https://doi.org/10.1016/j.fbio.2021.101038>
6. Hedge IC, Wendelbo P. Patterns of distribution and endemism in Iran. Notes from the Royal Botanic Garden, Edinburgh. 1978; 36(2): 441-464. <https://www.scopus.com/record/display.uri?eid=2-s2.0-0006003564&origin=inward&txGid=08c1677fbb653bcda02974e2f9bd74ed>
7. Kędzierska-Matysek M, Stryjecka M, Teter A, Skałecki P, Domaradzki P, Florek M. 2021. Relationships between the content of phenolic compounds and the antioxidant activity of Polish honey varieties as a tool for botanical discrimination. Molecules. 2021; 26(6): 1810. <https://doi.org/10.3390/molecules26061810>
