# Supplementary material for: Assessing the quality of bee honey on the basis of melissopalynology as well as chemical analysis
Source: PLoS One. 2023 Aug 8;18(8):e0289702. doi: 10.1371/journal.pone.0289702 (PMC10409281; doi:10.1371/journal.pone.0289702)
Supplement: Attachment — Submitted filename: Response to Reviewers.docx [file pone.0289702.s003.docx]

Dear Dr. Daniel de Paiva Silva

Thank you for providing us with the opportunity to submit a revised draft of the manuscript PONE-D-22-31247 entitled " Assessing the Quality of Bee Honey on the basis of melissopalynology as well chemical analysis ". We appreciate the time and effort that you and the reviewers have dedicated to providing valuable feedback on our manuscript. We are grateful to the reviewers for their insightful comments on the paper. We have been able to incorporate changes to reflect all the suggestions provided and all changes are highlighted in the manuscript text file.

Please see below a point-by-point response to the reviewers’ comments and concerns.

Best wishes

Ahmadreza Mehrabian

Dear Editor

- **Comments to the Author:**

After this first review round, all three reviewers raised significant issues that prevent the publication of your manuscript at this time. Still, the reviewers indicated that the manuscript has a potential to be published in PLoS One after improvements are considered.

Response: We appreciate the positive comments of the reviewers and have done our best to incorporate all comments, with specific responses below.

If applicable, we recommend that you deposit your laboratory protocols in protocols.io to enhance the reproducibility of your results. Protocols.io assigns your protocol its own identifier (DOI) so that it can be cited independently in the future.

Response: We saved our data in the figshare repository and placed the link in the text of the manuscript.

DATA AVAILABILITY STATEMENT

DOI: 10.6084/m9.figshare.22586863

https://figshare.com/s/949e02f3163f39991d4e

- **Journal requirements:**

1. Please ensure that your manuscript meets PLOS ONE's style requirements, including those for file naming. The PLOS ONE style templates can be found at

https://journals.plos.org/plosone/s/file?id=wjVg/PLOSOne_formatting_sample_main_body.pdf and <https://journals.plos.org/plosone/s/file?id=ba62/PLOSOne_formatting_sample_title_authors_affiliations.pdf>.

Response: We prepared the manuscript with PLOS ONE style requirements, including file naming.

2. We noticed you have some minor occurrence of overlapping text with the following previous publication(s), which needs to be addressed:

Plant Biogeography and Vegetation of High Mountains of Central and South-West Asia - https://link.springer.com/book/10.1007/978-3-030-45212-4, and

A bioclimatic characterization of high elevation habitats in the Alborz mountains of Iran - https://doi.org/10.1007/s00035-018-0202-9.

In your revision ensure you cite all your sources (including your own works), and quote or rephrase any duplicated text outside the methods section. Further consideration is dependent on these concerns being addressed.

Response: We added "A bioclimatic characterization of high elevation habitats in the Alborz mountains of Iran - https://doi.org/10.1007/s00035-018-0202-9" in our study area section:

The Alborz mountain range is located at 36° N from the Caspian Sea up to a height of 5671 meters (Noroozi et al.2018).

3. In your Methods section, please provide additional information regarding the permits you obtained for the work. Please ensure you have included the full name of the authority that approved the field site access and, if no permits were required, a brief statement explaining why.

Response: In the above study, we did not need a permit because the honey samples were provided to us voluntarily by the beekeepers, and because there was no need to enter protected areas or national parks, we did not need to obtain a permit.

Statement: In the above study, the beekeepers provided us with honey samples voluntarily and with full consent, and we did not need to obtain a permit due to not entering protected areas or national parks.

4. Please update your submission to use the PLOS LaTeX template. The template and more information on our requirements for LaTeX submissions can be found at <http://journals.plos.org/plosone/s/latex>.

Response: Corrections were made.

5. We note that you have stated that you will provide repository information for your data at acceptance. Should your manuscript be accepted for publication, we will hold it until you provide the relevant accession numbers or DOIs necessary to access your data. If you wish to make changes to your Data Availability statement, please describe these changes in your cover letter and we will update your Data Availability statement to reflect the information you provide.

Response: We saved our laboratory protocols and data in the figshare repository and placed the link in the text of the manuscript.

DATA AVAILABILITY STATEMENT

DOI: 10.6084/m9.figshare.22586863

https://figshare.com/s/949e02f3163f39991d4e

6. PLOS requires an ORCID iD for the corresponding author in Editorial Manager on papers submitted after December 6th, 2016. Please ensure that you have an ORCID iD and that it is validated in Editorial Manager. To do this, go to ‘Update my Information’ (in the upper left-hand corner of the main menu), and click on the Fetch/Validate link next to the ORCID field. This will take you to the ORCID site and allow you to create a new iD or authenticate a pre-existing iD in Editorial Manager. Please see the following video for instructions on linking an ORCID iD to your Editorial Manager account: <https://www.youtube.com/watch?v=_xcclfuvtxQ>.

Response: The ORCID of each author was declared.

Zahra Shakoori: <https://orcid.org/0000-0003-3057-8981>

Ahmadreza Mehrabian: <https://orcid.org/0000-0001-6633-3092>

Dariush Minai: <https://orcid.org/0000-0003-3589-7324>

Farid Salmanpour: <https://orcid.org/0000-0002-1517-9061>

Farzaneh Khajoei Nasab: https://orcid.org/0000-0002-2325-9555

- **Dear Prof. Ing. Jaromir Lachman, Reviewer #1:**

To the article, I have next comments and recommendations:

• In the article, there are many typing errors, which should be carefully checked and corrected, e.g., in Abstract: you should use 0.08 to 0.05 ppm or 0.08-0.05 and so on throughout the text. Like wise L. 47:…climate of the region…; L. 61:…botanical…;, Table 1: Bignoniaceae L. 98: instead antioxidative should be used antioxidant activity or antioxidant capacity and similar in the all article.

Response: We tried to carefully check and correct typographical errors. For example, we used 0.08 to 0.05 pp throughout the text. Instead of antioxidant, we used antioxidant activity in the article. Other mentioned points such as amendment in L. 46:...climate of the region...; L. 58:…botanical…;, Table 1: Bignoniaceae done.

• Latin names of plants should be in Italics through all text and References, e.g., L. 140: Quercus macranthera, etc.

Response: The Latin names of the plants were written in italics in all texts and references.

• All applied equipment and chemicals should be carefully completed with the name of the producer, town and country (state) – check and complete the missing date especially in section Laboratory studies.

Response: The name of the manufacturer, city and country (state) of all equipment and chemicals used were carefully entered with the name of the manufacturer, especially in the laboratory studies section.

- L.212: Hot plate model HP100 made by Mtops company in South Korea
- L.216: Centrifuge model TDL-4 made by Jenius China
- L.247: Merck Chemical Company in Germany.
- L.248: 2,2-Diphenyl-1-picrylhydrazyl (DPPH) was obtained from Sigma American Chemical.
- 258: JENUS V-1100 spectrophotometer Made by DLAB China
- In the centrifugation convert rpm to g values and correct centrifugation instead centrifuge(s), where it is appropriate.

Response: In the centrifuge, we converted revolutions per minute to g values and corrected centrifugation instead of centrifuge where appropriate.

- L. 637: 1% ferric chloride…

Response: The item mentioned in L. 637 was not found, but it was found in line 264 and corrected.

- Unify Apocynaceae & Apiaceae or Apocynaceae and Apiaceae, similarly throughout the text.

Response: Apocynaceae and Apiaceae are similarly united throughout the text.

- L. 294 and elsewhere: total phenolic content or total phenolics.

Response: Throughout the text, the phenol compounds and total phenol were changed to total phenolic content.

- Some sentences are completely confusing and should be corrected, e.g. L. 336-337:…in the Estonia (Puusepp and Koff 2014). Croatia (?). Our results show…

Response: Confusing sentences were completely corrected throughout the text.

- The main problem is that the citation system should be substantially redone according to system used in PLOS One, i.e., using brackets like [1, [2,[3, etc. and hence the references they will not be in alphabetical order.

Response: In this version, we have adjusted the entire manuscript according to PLOS One guidelines.

- According to this system you should change the order of references. In References you should apply Italics in Latin names of plants (Prunus avium, Schefflera abyssinica) and bees (Apis mellifera), use the full titles of Journals with capitals of nouns and adjectives, like Food Chemistry, Nutrition Research, International Journal of Food Microbiology, Current Medicinal Chemistry, LWT-Food Science and Technology, etc., do not use Italics in titles of journals (not mixture). Do not use capitals in the name of article – should be e.g., Study of organoleptic, physical, and chemical indicators of honey in Tashkent region. Do not use pp. and p. in references, by the way some articles in new volumes use an article number (one number) instead of pages.

Response: We modified and prepared all the references according to the instructions of the journal and all the mentioned points were applied.

- Books, Theses and Website should be implemented along journals referenced with their citation number.

Here you are some examples:

1. Hailu D, Belay A. Melissopalynology and antioxidant properties used to differentiate Schefflera abyssinica and polyfloral honey. PLOS One. 2020;15(10): e0240868.https://doi.org/10.1371/journal.pone.0240868

2. Alvarez-Suarez MJ., Giampieri F, Battino M. Honey as a source of dietary antioxidants: structures, bioavailability and evidence of protective effects against human chronic diseases. Current Medicinal Chemistry. 2013; 20(5), 621-638. https://doi.org/10.2174/092986713804999358

3. Berberian M.The southern Caspian: a compressional depression floored by a trapped, modified oceanic crust. Canadian Journal of Earth Sciences. 1983; 20(2): 163-183. https://doi.org/10.1139/e83-015

4. Chua LS, Rahaman NL, Adnan NA, Eddie Tan, TT. Antioxidant activity of three honey samples in relation with their biochemical components. Journal of Analytical Methods in Chemistry. 2013: 313798. https://doi.org/10.1155/2013/313798

5. El-Senduny FF, Hegazi NM, Abd Elghani GE, Farag MA. Manuka honey, a unique mono-floral honey. A comprehensive review of its bioactives. metabolism, action mechanisms, and therapeutic merits. Food Bioscience. 2021; 42:101038. https://doi.org/10.1016/j.fbio.2021.101038

6. Hedge IC, Wendelbo P. Patterns of distribution and endemism in Iran. Notes from the Royal Botanic Garden, Edinburgh. 1978; 36(2): 441-464. https://www.scopus.com/record/display.uri?eid=2-s2.0-0006003564&origin=inward&txGid=08c1677fbb653bcda02974e2f9bd74ed

7. Kędzierska-Matysek M, Stryjecka M, Teter A, Skałecki P, Domaradzki P, Florek M. 2021. Relationships between the content of phenolic compounds and the antioxidant activity of Polish honey varieties as a tool for botanical discrimination. Molecules. 2021; 26(6): 1810. <https://doi.org/10.3390/molecules26061>

Response: We have made the mentioned corrections completely.

- **Dear Reviewer #2:**

1. Authors stated in line 105 that “the study tries to analyze the mentioned data in relation to geographical factors.” and they try to analyze this aspect in the section 5. Discussion. However, for drawing conclusions if chemical parameters of honey depend on geographical factors, the proper statistical analysis is needed.

Response: This section has been completely revised.

2. The sentence “We compared the humidity of honey samples with the humidity of the areas from which the samples were collected, which shows that the humidity of the environment affected the humidity of honey samples” must be proven by the statistical analysis.

Response: We removed the map related to the effect of ambient humidity on the humidity of honey samples and using the minitab software, we got regression fitted line plots statistical model.

The regression result between the average annual relative humidity of the region (independent variable) and the humidity of the honey sample (dependent variable) showed that there is a significant relationship (p value 0.000) between them, so that the humidity of honey samples is a function of the humidity of the environment.

**Fig 5.** Comparison of moisture content of honey samples with moisture content of the collection site

**Fig 5.** Regression related to the effect of environmental humidity on the moisture of honey samples

3. On Figure. 6 the font size on the axis descriptions must be increased for greater readability. It is also no clear for me, what is the Loading. Do Authors mean coefficient of correlation?

Response: In this section, more detailed reforms were needed, and these reforms were carried out.

Yes, loading means the same coefficient of correlation that was modified in the figure.

4. The sentence in lines 299-302 should be corrected. It is completely incomprehensible.

Response: In this section, more detailed reforms were needed, and these reforms were carried out.

5. Figure 8 – Authors should inform where the reader can find the explanation of codes of samples. On this picture Authors depict results of cluster analysis but I can’t find in the text how these results are used, or which conclusions were drawn base on them.

Response: We saved our data in the figshare repository and placed the link in the text of the manuscript, codes can be found in it.

DOI: 10.6084/m9.figshare.22586863

<https://figshare.com/s/949e02f3163f39991d4e>

In addition, this section was modified in the text of the manuscript and some information was included in the text of the manuscript.

6. Text must be linguistically and editorially corrected.

Response: The text was linguistically and editorially corrected.

**Dear Dr. Haya i. Aljohar, Reviewer #3**

- Introduction
- The introduction consists of many staccato sentences, forcing the author to use an infinite number of linking phrases, such as: as well as , Furthermore, in addition, Therefore etc (Rewrite the entire introduction in proper English).

Response: We rewrote the entire introduction in proper English.

- Line 49 remove( the)

Response: It was corrected.

- Line 51 use counterfeit instead of fake

Response: Counterfeit replaced fake.

- Line 54 use and instead of as well as and change has to have.

Response: It was corrected

- Line 56 (Louveaux (1970, 1978)) Add reference

Response: Line59: Reference added (Louveaux et al. 1970; 1978)

- Line 59- 65 Rewrite this paragraph (There are many separate sentences that must be linked together and rewritten in proper English).

Response: We have rewritten this paragraph in proper English.

- Line 73 (SW Asia) It was mentioned here for the first time, so it must be written without abbreviation.

Response: Line 78: Southwest Asia is written instead of SW Asia

- Rewrite the paragraph from line 69-78.

Response: The text of the manuscript was completely rewritten and revised

- Study area
- Rewrite in a proper English

Response: All the text of the manuscript was rewritten in proper English.

- Field studies Change to sample collection

Response: Field studies Changed to sample collection

- Line 179 What is the meaning of amount of hight.

Response: We mean the same height.

Line 197: The amount of hight was changed to height

- Laboratory studies
- It is better to put a paragraph for the chemicals and reagents used and another paragraph for the devices and tools used in the experiment separately.

Response: We have completely revised this paragraph and tried to separate the content as much as possible in the same paragraph to make it clearer.

- Rewrite in a proper English.

Response: All the text of the manuscript was rewritten in proper English.

- Line 192 Then, remove large …. By passed through

Response: corrections were made

- Line 223 DPPH abbreviation for what? It was mentioned here for the first time, so it must be written without abbreviation.

Response: Line 248: DPPH was written in full.

2,2-Diphenyl-1-picrylhydrazyl (DPPH) was obtained from Sigma American Chemical Company.

- Line 237( 1% ) Repeated twice in the sentence why?

Response: FeCl3 1% is the abbreviation of 1% ferric chloride solution, that's why 1% is repeated twice in the sentence.

Line 264: The sentence was modified in this way: In this experiment, 1% ferric chloride solution (FeCl3 1%)...

- Results
- Rewrite scientifically in a proper English

Response: It was scientifically rewritten in appropriate English

- Add a table containing honey samples names, symbols, and geographical areas

Response: We have added the requested information and stored it in the figshare repository as backup information.

DATA AVAILABILITY STATEMENT

DOI: 10.6084/m9.figshare.22586863

https://figshare.com/s/949e02f3163f39991d4e

- Line 273 Was in the range instead of was the lowest.

Response: Line 339: It was corrected.

- Figure 6 Not clear

Response: Response: In this section, more detailed reforms were needed, and these reforms were carried out.

- Line 299-304 This part is incomprehensible and needs reformulation

Response: Line 424 – 427: The paragraph has been completely revised.

The PCA did not result in any significant differences between all honey samples studied (Fig. 9). Considering that the honey samples studied were collected from different geographical areas, samples from the same area were not placed next to each other in the plot. This shows that honey samples from different locations in the same area have different chemical compositions.

- Unify the method of writing references within the article

Response: Unify the references in the article were prepared as based on the journal's guidelines.

- Line 348 Rephrasing

Response: Rewriting done
