# Supplementary material for: Assessing the quality of bee honey on the basis of melissopalynology as well as chemical analysis
Source: PLoS One. 2023 Aug 8;18(8):e0289702. doi: 10.1371/journal.pone.0289702 (PMC10409281; doi:10.1371/journal.pone.0289702)
Supplement: Attachment — Submitted filename: Review of PONE-D-22-31247_R1.docx [file pone.0289702.s004.docx]

**Review of PONE-D-22-31247R1**

The article has been in part improved; however I still have some little and larger reminders.

- L. 63:..dependent on…
- In the article you used both units: mL and μL or ml and μl. Please unify to one expression of the liter unit – l or L. I prefer mL and μL throughout the article, correct it.
- Table 1: **Acanthaceae** should be in Bold in the column Family.
- L. 263:..(Fig. 6).
- L. 290:..(Alimentarius Codex)…
- My main comments are about the citations and references, which need to be corrected. Citations of number in brackets: they should be from the start of the article text, i.e., from 1 to 95 and subsequently renumbered in References 1-95. So, References will not be arranged alphabetically, but from 1 to 95; so [1] will be Zimmermann MI ….; [2] Kečkeš S, Gašić U, Veličković TĆ, Milojković-Opsenica D, Natić M, Tešić Ž….; [3] Mehrabian A, Rad MA….; [4] Alvarez-Suarez JM…; [5] Louveaux J, Maurizio A, Vorwohl G…; [6] Louveaux J, Maurizio A, Vorwohl G. Methods of melissopalynology, etc. This way you can continue editing further.
- Another important item is the title of referenced journals. You can choose two different ways - either the full title of the journal or its abbreviation. However, the style should be unique and not a mixture.
- Here you are some examples: Food Chemistry (full) or Food Chem. (abbreviation); Nutrition Research (full) or Nutr Res. (abbreviation); International Journal of Food Microbiology (full) or Int J Food Microbiol. (abbreviation); Current Medicinal Chemistry (full) or Curr Med Chem. (abbreviation); Acta Botanica Fennica (full) or Acta Bot Fenn. (abbreviation); Veterinary Microbiology (full) or Vet Microbiol. (abbreviation). You should follow these corrections in References and choose only one method – either the full title or its abbreviation.
