# Supplementary material for: Assessing the quality of bee honey on the basis of melissopalynology as well as chemical analysis
Source: PLoS One. 2023 Aug 8;18(8):e0289702. doi: 10.1371/journal.pone.0289702 (PMC10409281; doi:10.1371/journal.pone.0289702)
Supplement: Attachment — Submitted filename: honey article .docx [file pone.0289702.s006.docx]

- **The article need proofreading, especially the shaded parts**
- **The numbering of reference uncorrected**
- **Put all instruments and equipment under instrumentations.**
- **Put all chemicals and reagent under chemical and reagents**.
- **Delete everything marked as deleted**.
- **Line 21:** Melissopalynological and chemical analysis of honey provide us useful
- Line 57: Comma instead of fullstop
- Line 113: fig. 1 Write complete caption, it must identify what’s the red spots.
- Line 17: in the period from
- Line 122: One sample
- Line 123: Remove comma.
- Line 123-124: Delete this part.
- Line 126: Start with - material or chemical and reagents - instrumentations.
- Line132-133: Then, the honey samples were filtrate.
- Line 135-140: Rewrite this paragraph. What is the speed and how many round per minutes?
- Line 151: from instead of by
- Line 156: Start the statement with For pollen identification we used …..
- Line 161: Put it under chemical and reagents.
- Line 171: add Place
- Line 177-180: 0.1 ml of ferric acid (FeCl3) was mixed with 0.1 ml of diluted honey and the absorbance was measured at 590 nm., against Phenol solution (0.09%) which was used as standard. []
- Line 185: Add information about this instrument under instrumentation.
- Line 190-193: proofreading
- Fig.3 alb, c,: Is it actual figure for your sample.
- Table 1: Add column for samples name or number.
