# Supplementary material for: Assessing the quality of bee honey on the basis of melissopalynology as well as chemical analysis
Source: PLoS One. 2023 Aug 8;18(8):e0289702. doi: 10.1371/journal.pone.0289702 (PMC10409281; doi:10.1371/journal.pone.0289702)
Supplement: Attachment — Submitted filename: Response to Reviewers.docx [file pone.0289702.s007.docx]

Dear Dr. Daniel de Paiva Silva

Thank you for providing us with the opportunity to submit a revised draft of the manuscript PONE-D-22-31247 entitled " Assessing the Quality of Bee Honey on the basis of melissopalynology as well chemical analysis ". We appreciate the time and effort that you and the reviewers have dedicated to providing valuable feedback on our manuscript. We are grateful to the reviewers for their insightful comments on the paper. We have been able to incorporate changes to reflect all the suggestions provided and all changes are highlighted in the manuscript text file.

Please see below a point-by-point response to the reviewers’ comments and concerns.

Best wishes

Ahmadreza Mehrabian

Dear Editor

- **Comments to the Author:**

After this new review round, one of the reviewers still believes your manuscripts deserves a major review. Among several issues, the reviewer believes (and I agree) that you manuscript needs an in-deep review and proofreading. Therefore, considering the issues raised by this reviewer (Reviewer #3), I will grant you a two-month period to deliver the new review for the appreciation of the reviewer.

Response: We appreciate the positive comments of the reviewers and have done our best to incorporate all comments, with specific responses below.

If applicable, we recommend that you deposit your laboratory protocols in protocols.io to enhance the reproducibility of your results. Protocols.io assigns your protocol its own identifier (DOI) so that it can be cited independently in the future.

Response: We saved our data in the figshare repository and placed the link in the text of the manuscript.

DATA AVAILABILITY STATEMENT

DOI: 10.6084/m9.figshare.22586863

https://figshare.com/s/949e02f3163f39991d4e

- **Dear Prof. Ing. Jaromir Lachman, Reviewer #1:**
- L. 63: dependent on…

Response: L. 63 it was corrected.

- In the article you used both units: mL and uL or ml and ul. Please unify to one expression of the liter unit – l or L. I prefer mL and uL throughout the article, correct it.

Response: All µl were changed to µL, and ml to mL according to the reviewer's opinion.

- Table 1: Acanthaceae should be in Bold in the column Family.

Response: It was corrected.

- L. 263: (Fig. 6).

Response: It was corrected.

- L. 290:(Alimentarius Codex)…

Response: L.294: It was corrected.

- My main comments are about the citations and references, which need to be corrected. Citations of number in brackets: they should be from the start of the article text, i.e., from 1 to 95 and subsequently renumbered in References 1-95. So, References will not be arranged alphabetically, but from 1 to 95; so [1) will be Zimmermann MI ….; [2) Kečkeš S, Gašić U, Veličković TĆ, Milojković-Opsenica D, Natić M, Tešić Ž….; [3) Mehrabian A, Rad MA….; [4) Alvarez-Suarez JM…; [5) Louveaux J, Maurizio A, Vorwohl G…; [6) Louveaux J, Maurizio A, Vorwohl G. Methods of melissopalynology, etc. This way you can continue editing further.

Response: All references were edited according to the opinion of the respected referee.

- Another important item is the title of referenced journals. You can choose two different ways - either the full title of the journal or its abbreviation. However, the style should be unique and not a mixture.

Here you are some examples: Food Chemistry (full) or Food Chem. (abbreviation); Nutrition Research (full) or Nutr Res. (abbreviation); International Journal of Food Microbiology (full) or Int J Food Microbiol. (abbreviation); Current Medicinal Chemistry (full) or Curr Med Chem. (abbreviation); Acta Botanica Fennica (full) or Acta Bot Fenn. (abbreviation); Veterinary Microbiology (full) or Vet Microbiol. (abbreviation). You should follow these corrections in References and choose only one method – either the full title or its abbreviation.

Response: It was corrected.

- **Dear Reviewer #2:**

Thank you for your constructive and valuable comments and we are glad that we were able to implement your comments correctly and completely.

**Dear Dr. Haya i. Aljohar, Reviewer #3:**

- The numbering of reference uncorrected

Response: It was corrected.

- Put all instruments and equipment under instrumentations.

Response: L. 135-142 we put all the tools and equipment under the instrumentations.

- Put all chemicals and reagent under chemical and reagents.

Response: L.131-134 we placed all chemicals and reagents under chemicals and reagents.

- Delete everything marked as deleted.

Response: We removed everything marked as removed.

- Line 21: Melissopalynological and chemical analysis of honey provide us useful.

Response: It was corrected.

- Line 57: Comma instead of fullstop.

Response: It was corrected.

- Line 113: fig. 1 Write complete caption, it must identify what’s the red spots.

Response: L.117 All the sampling locations are marked using red dots.

In the legend of Figure 1, it is also explained about the red dots that indicate the sampling points.

- Line 117: in the period from

Response: L.121 it was corrected.

- Line 122: One sample

Response: L.126 it was corrected.

- Line 123: Remove comma.

Response: L.127 the comma was removed

- Line 123-124: Delete this part.

Response: L.127 – 128 this part was deleted

- Line 126: Start with - material or chemical and reagents - instrumentations.

Response: L.131 it was corrected.

Material or chemical and reagents

- Line132-133: Then, the honey samples were filtrate.

Response: L.152 it was corrected.

- Line 135-140: Rewrite this paragraph. What is the speed and how many round per minutes?

Response: L.154-159 it was corrected.

- Line 151: from instead of by

Response: L.171 From was used instead of by

- Line 156: Start the statement with For pollen identification we used …..

Response: L.176 it was corrected.

- Line 161: Put it under chemical and reagents.

Response: L.132 – 134 we put the sentence under chemicals and reagents

- Line 171: add Place

Response: It was corrected.

- Line 177-180: 0.1 ml of ferric acid (FeCl3) was mixed with 0.1 ml of diluted honey and the absorbance was measured at 590 nm., against Phenol solution (0.09%) which was used as standard. []

Response: L.197 – 198 it was corrected.

- Line 185: Add information about this instrument under instrumentation.

Response: Information about the refractometer was added in the instrumentation section.

- Line 190-193: proofreading

Response: L. 205-210 it was corrected.

Principal component analysis (PCA) was used to determine the relationship between the geographical origin of honey samples and the amount of chemical compounds they contained. The main objective of this analysis was to determine whether honey samples in a geographic area contained the same amount of chemical compounds. Chemical parameters (pH), chemical compounds (total phenolic content and antioxidant activity) and moisture content were used for PCA analysis. A variance-covariance matrix was generated for all honey samples and four chemical parameters. Data were analyzed using PAST software version 3.21[60].

- Fig.3 alb, c,: Is it actual figure for your sample.

Response: Yes, all the photos in Figure 3(a,b,c,d) are related to the pollen extracted from the honey samples in this study, which were imaged using a scanning electron microscope.

Table 1: Add column for samples name or number.

Response: In Table 1, a column was added for the number of samples.

- Is it actual figure for your sample if it is not please use the actual figure?

Response: Yes, all the Figures are related to the pollen extracted from the honey samples in this study, which were imaged using a scanning electron microscope.
