# Supplementary material for: Assessing the quality of bee honey on the basis of melissopalynology as well as chemical analysis
Source: PLoS One. 2023 Aug 8;18(8):e0289702. doi: 10.1371/journal.pone.0289702 (PMC10409281; doi:10.1371/journal.pone.0289702)
Supplement: Attachment — Submitted filename: Review of PONE-D-22-31247_R2.docx [file pone.0289702.s008.docx]

**Review of PONE-D-22-31247_R2**

The article has been substantially improved according to reviewers´ remarks and recommendations. To the article, I have some remarks, regarding journal titles and names of articles in References, which can be easily corrected and then the article can be ready for acceptance:

- L. 422: Journal of Apicultural Research; L. 441-442: L. 445: Veterinary Microbiology; L. 450-452: Study of organoleptic, physical and chemical indicators of honey in Tashkent region. L. 551: Current Medicinal Chemistry; L. 554: Nutrition Research; L. 570: Alpine Botany; International Journal of Food Microbiology; L. 593: Journal of Analytical Methods in Chemistry; L. 598: Palaeontologia Electronica; L. 601: Comprehensive Reviews in Food Science and Food Safety; L. 613: Bee world; L. 626, 632, 655, 672, 675: Food Chemistry; L. 638: Food Control; L. 644-645: Journal of Food Science and Technology; L. 651: Journal of Applied Microbiology; L. 657-658: British Journal of Surgery; L. 698-699: Oxidative Medicine and Cellular Longevity.
