# Supplementary material for: Assessing the quality of bee honey on the basis of melissopalynology as well as chemical analysis
Source: PLoS One. 2023 Aug 8;18(8):e0289702. doi: 10.1371/journal.pone.0289702 (PMC10409281; doi:10.1371/journal.pone.0289702)
Supplement: Attachment — Submitted filename: Response to Reviewers.docx [file pone.0289702.s009.docx]

Dear Dr. Daniel de Paiva Silva

Thank you for providing us with the opportunity to submit a revised draft of the manuscript PONE-D-22-31247 entitled "Assessing the Quality of Bee Honey on the basis of melissopalynology as well chemical analysis ". We appreciate the time and effort that you and the reviewers have dedicated to providing valuable feedback on our manuscript. We are grateful to the reviewer for his insightful comments on the paper. We have been able to incorporate changes to reflect all the suggestions provided and all changes are highlighted in the manuscript text file.

Please see below a point-by-point response to the reviewers’ comments and concerns.

Best wishes

Ahmadreza Mehrabian

- **Dear Prof. Ing. Jaromir Lachman, Reviewer #1:**

The article has been substantially improved according to reviewers´ remarks and recommendations. To the article, I have some remarks, regarding journal titles and names of articles in References, which can be easily corrected and then the article can be ready for acceptance:
• L. 422: Journal of Apicultural Research; L. 441-442: L. 445: Veterinary Microbiology; L. 450-452: Study of organoleptic, physical and chemical indicators of honey in Tashkent region. L. 551: Current Medicinal Chemistry; L. 554: Nutrition Research; L. 570: Alpine Botany; International Journal of Food Microbiology; L. 593: Journal of Analytical Methods in Chemistry; L. 598: Palaeontologia Electronica; L. 601: Comprehensive Reviews in Food Science and Food Safety; L. 613: Bee world; L. 626, 632, 655, 672, 675: Food Chemistry; L. 638: Food Control; L. 644-645: Journal of Food Science and Technology; L. 651: Journal of Applied Microbiology; L. 657-658: British Journal of Surgery; L. 698-699: Oxidative Medicine and Cellular Longevity.

**Response:** All the references were corrected and according to the opinion of the respected referee, the first letter of the names of the journals in the reference was also capitaled.
